# Auditing farm animal refuge: management and welfare of livestock in disaster situations

**DOI:** 10.3389/fvets.2025.1681087

**Published:** 2025-10-24

**Authors:** Sillas M. S. Silva, Cássia C. V. Del Valle, Vanessa M. Reis, Carolina R. Bonatto, Gislene F. S. R. Fournier, Felipe Bertelli, Alex Castro

**Affiliations:** ^1^Fauna Team, AECOM Brazil, Belo Horizonte, Brazil; ^2^Independent Researcher, Belo Horizonte, Brazil

**Keywords:** animal welfare, cattle refuge, farm animal refuge, livestock disaster response, emergency management, veterinary auditing, animal science auditing, governance

## Abstract

The collapse of mining dams in Brazil exposed significant deficiencies in emergency planning for livestock, resulting in prolonged vulnerability of cattle in disaster-affected areas. This study presents a pioneering initiative that implemented a temporary refuge for approximately 575 cattle, incorporating veterinary oversight, nutritional and reproductive management, and behavioral monitoring. The initiative was developed within the framework of the Fauna Term of Covenant, an institutional agreement that ensured independent technical auditing and legal accountability. This experience offers a replicable model for protecting livestock in socio-environmental crises. It also illustrates how veterinary and animal sciences intersect with governance, ethics, and environmental justice, emphasizing that effective animal welfare in disaster contexts requires more than technical solutions. It demands sustained institutional commitment and interdisciplinary collaboration. The findings contribute to the development of future frameworks for integrating production animals into emergency response and public policy.

## 1 Introduction

In Brazil, uncontrolled anomalies and the collapse of mining dams (rupture, over-flow, or imminent risk of rupture) have caused significant social, environmental, and economic impacts in several cities in Minas Gerais, resulting in legislative and legal responses aimed at minimizing the damage and preventing new occurrences.

In this context, several public institutions, especially the Public Prosecutor’s Office of Minas Gerais (MPMG), have adopted the Term of Covenant (ToC) as an effective alternative to the traditional judicial process, especially in cases collective or diffuse interests, such as environmental rights. Based on the clauses and conditions of the ToC, the post-disaster environmental audit was instituted (1), with the aim of assessing the impacts and potential for recurrence (2, 3).

Supported by current Brazilian legislation, the ToC enables companies to be held directly liable for socio-environmental damages. In the case of the ToC Fauna, the agreement established specific obligations for the mining company to ensure the complete protection of all animals affected by ruptures or emergency evacuations, including domestic, wild, and production animals. This initiative establishes a legal framework aimed at safeguarding animal life in disaster situations, reinforcing the role of the State in defending animal welfare and repairing environmental damage (4, 5).

The agreement implemented by the MPMG, a pioneering agreement, established the monitoring of independent technical environmental audits in specific actions to safeguard the affected animals, such as adapting shelters, maintaining their wellbeing, locating guardians, and promoting adoption. In this context, AECOM Brazil became the auditing company, responsible for examining the execution of activities implemented by the responsible party in a technical-scientific and independent manner, encouraging continuous review of processes and, consequently, promoting the proper functioning of the actions. This process involved identifying and correcting possible weaknesses.

It is important to highlight that the impact of anthropogenic technological disasters (6), which occurred in the primary sector, permeated physical or social vulnerabilities (7, 8), in addition to damage to nature with serious consequences that must be mitigated based on lessons learned around the world (9, 10). In this scenario, production systems involving animals were exposed to the devastation of dam ruptures or new threats of structural failures according to the emergency level (11).

Thus, structural changes in the way animals perceive disasters are initiated. The rescue or evacuation of farm animals requires prior planning, whether through logistics, which includes selecting a suitable vehicle for the species to be transported and establishing a predetermined destination, or through mapping and mobilizing a team specialized in managing the species.

The evacuated or rescued animals were sent to structures similar to Animal Control Centers (ACCs), whose guidelines were originally developed for the sheltering of wildlife but were predominantly applied to companion animals such as dogs and cats (12, 13). Given this context, it became necessary to reinterpret the shelter model to address the specific needs of production animals, which required adapting livestock systems to emergency conditions (14, 15).

In this scenario, the term Farm Animal Refuge (FAR) was established, referring to the temporary sheltering of livestock species, with an initial focus on cattle. This concept aligns with international discussions on the need for species-specific sheltering strategies, as emphasized in studies on the behavioral and welfare needs of farm animals in emergency contexts (16).

The ACCs for domestic animals generally offer a temporary stay, with the primary objective of recovering, resocializing, and reintroducing the animals into society through responsible adoptions. In the case of farm animals, such as cattle, the process is more complex, involving the resolution of issues related to compensation or returns to their owners. This procedure can be lengthy, due to the need for an agreement between the parties, which can delay the final destination of the animals. In this context, this paper aims to report on the experience of the independent technical audit team, highlighting how ToC Fauna allowed monitoring and recommending preventive and, in some cases, corrective actions for the responsible party, aiming to promote the welfare of the cattle during their stay at the farm animal refuge.

## 2 Methods

This study presents the technical auditing process conducted by AECOM Brazil between 2019 and 2024, under the legal framework of the Fauna Term of Covenant (ToC Fauna). The audit focused on the welfare and management of approximately 575 cattle rescued or evacuated from areas at risk of mining dam collapse in the metropolitan mesoregion of Belo Horizonte, Minas Gerais.

The methodology integrated three complementary strategies: Field inspections at the Farm Animal Refuge, a commercial farm adapted to temporarily shelter cattle; Review of operational and veterinary documentation, including health records and management protocols; Institutional coordination with public agencies to ensure alignment with regulatory and welfare standards.

**Figure 1:**
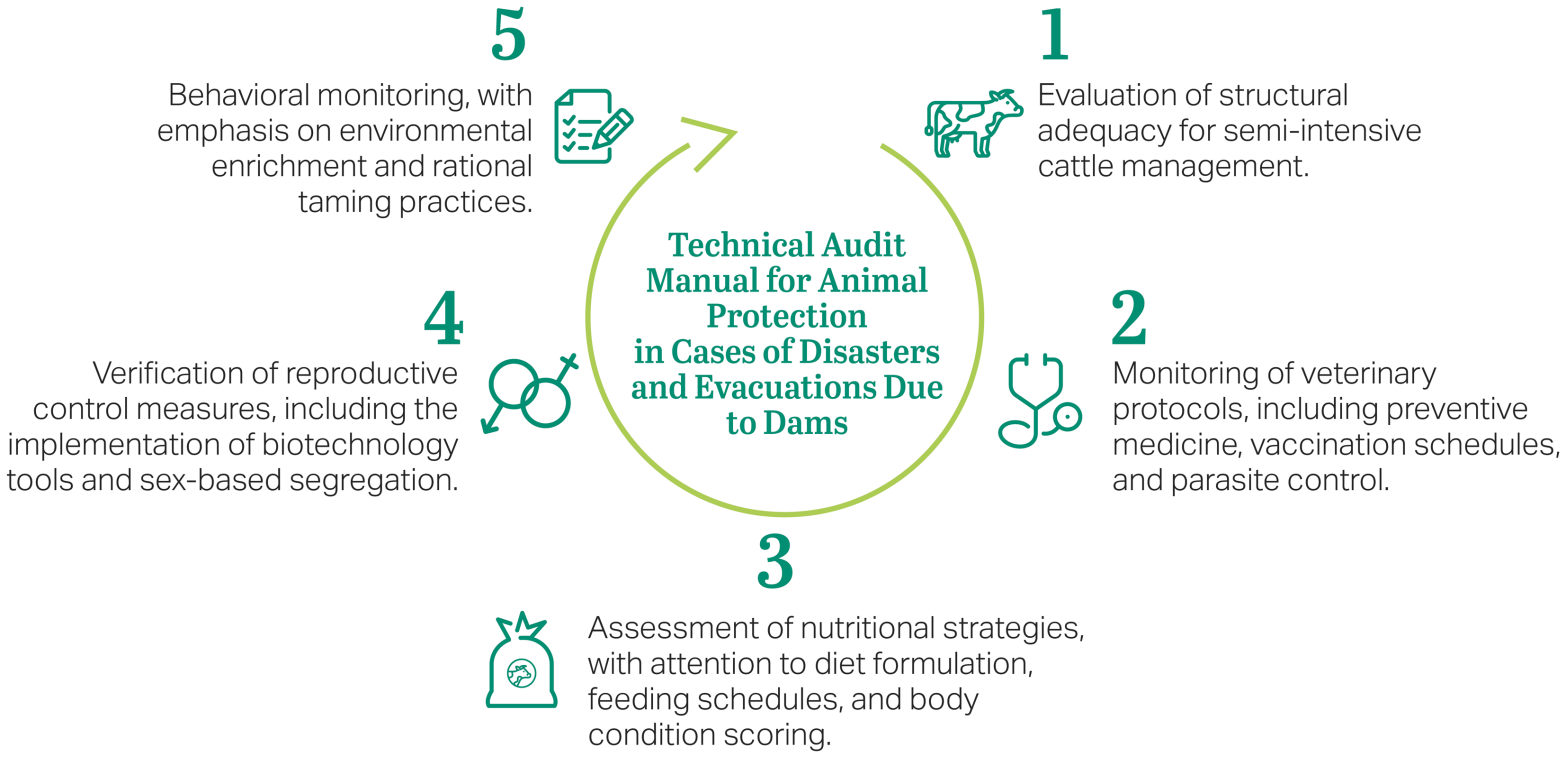
Circular diagram illustrating the five pillars of the technical auditing process conducted at the farm animal refuge. Adapted from the AECOM technical audit manual for animal protection in cases of disasters and evacuations due to dams.

Auditing activities were guided by a standardized protocol developed specifically for animals in disaster contexts. This protocol incorporates principles from animal science, shelter veterinary medicine, and environmental governance. The evaluation framework was organized into five core pillars (Figure 1): behavioral monitoring, reproductive control, structural adequacy, veterinary protocols, and nutritional strategies. Recommendations were issued to the responsible party and monitored through iterative assessments, ensuring continuous improvement in shelter conditions and animal welfare. Further methodological details, including the full protocol and operational guidelines, are available in the Technical Audit Manual provided in the Supplementary Material.

It is important to note that, as this was an independent technical audit conducted under specific legal clauses, certain operational data are subject to confidentiality restrictions and therefore cannot be fully disclosed. Accordingly, the results are presented in an aggregated and illustrative manner to ensure reader comprehension without compromising the integrity of sensitive information or institutional confidentiality.

### 2.1 Auditing governance and protocol standardization

The ToC Fauna was established in 2019, with AECOM Brazil designated as a technical intervener in the process. Within its scope of responsibilities, AECOM Brazil serves as a formal interlocutor between the audited company and the competent public agencies, specifically the State Coordination for Animal Defense (CEDA) and the Operational Support Center for Environmental Defense (CAOMA).

The purpose of the ToC Fauna is to implement measures aimed at protecting animals present in areas of direct influence of geotechnical structures classified as having “very high” or “extreme” Consequence Classification, according to the similar criteria established by the Brazilian National Mining Agency (ANM). The planned actions must be carried out according to the Alert Level or Emergency Levels, as outlined in ANM Resolution No. 175/2024 (17).

Among the clauses of the ToC Fauna aimed at protecting and maintaining the wellbeing of affected production animals, the following stand out: (1) immediate rescue of isolated animals; (2) identification of animals; (3) implementation of preventive veterinary medicine; (4) adequate and good-quality feed and water; (5) favorable wellbeing conditions for all rescued animals, minimizing stress and fear; (6) veterinary care for all rescued species; (7) birth control; (8) reintegration with their original guardians; (9) adoption and post-adoption monitoring program; (10) creation of an animal database (individual traceability); (11) monitoring of actions by an independent technical audit (MPMG – ToC Fauna) (18, 19). Additionally, the slaughter of any affected animal species is prohibited (20), except in cases of health emergency.

Based on this aspect, the independent technical audit team from AECOM Brazil developed a method to standardize procedures in animals auditing (21). This method is specific, replicable, and adaptable to different objects related to animal issues, reducing subjectivity during the evaluation process. The analysis of the audit process is supported by technical and legislative knowledge, especially that recommended by: CEDA/MPMG; World Organization for Animal Health – WOAH; Federal and Regional Councils of Veterinary Medicine – CFMV/CRMV; Ministry of Agriculture and Livestock – MAPA; State Secretariat for the Environment and Sustainable Development of Minas Gerais – SEMAD; State Institute of Forests of Minas Gerais – IEF, Minas Gerais Institute of Agriculture – IMA, and Institute of Veterinary Medicine of the Collective – IMVC.

## 3 Results

The cattle were kept in a farm animal refuge (FAR) specifically designed to house the animals on a farm located in the metropolitan mesoregion of Belo Horizonte, Minas Gerais. The environment corresponds to a commercial farm, with adequate structure for keeping the animals in a semi-intensive system. Approximately 575 cattle, originating from the areas downstream of different geotechnical structures, were rescued or evacuated. The animals were grouped according to the categories of interest (sex, age, weight) to optimize management and care in the shelter.

As part of ongoing adjustments under the ToC Fauna framework, the FAR underwent managerial restructuring. Initially occupying 877 hectares, including 488 hectares of pasture, the facility was reduced to 213 hectares by October 2023, with 193 hectares remaining as pasture. To mitigate the impact of reduced grazing capacity, trough lines were installed to support twice-daily corn silage supplementation, ensuring nutritional adequacy for the remaining cattle. The operational planning adopted during this transition is detailed in Table 1, which outlines the management strategies implemented to maintain animal welfare and optimize resource use under the revised spatial conditions.

**Table 1:** Operational planning and management strategies implemented at the farm animal refuge.

Category	Details
Management type	Semi-intensive
Feeding management adjustments	Daily supplementation with corn silage at a rate of 20–30 kg/animal/day, adjusted according to age group and live weight (LW), equivalent to 2% LW, administered twice daily to meet maintenance requirements.
	Feeding lines were constructed across 12 paddocks to support nutritional supplementation. A total of 30 troughs were distributed among paddocks 01, 02, 03, 05, 06, 07, 09, 11, 12, 13, and 14, while paddock 04 received 100 troughs due to its larger capacity and strategic role in feed distribution.
	Pre-round inspection of troughs to assess leftovers and ensure proper disposal; evaluation of batch-specific quantity adjustments.
Continuous measures	Provision of protein-enriched salt limited to 400 g/day/animal.
	Geriatric cattle feed provided at 0.5% of live weight.
	Rational cattle handling employing pressure and release techniques.
Paddock area	Ranging from 10 to 24 hectares per paddock.
Animal welfare program	Monitoring of hierarchical disputes during silage distribution; observation of sodium intake behavior in non-castrated males; installation of shade nets and mobile structures; use of tires as enrichment tools; implementation of efficiency assessments for environmental enrichment in animals not housed in nursing facilities.

Throughout the ToC Fauna, challenges related to the welfare of sheltered animals, and the management of FAR emerged. Initially, the audit focused on actions related to maintaining the welfare of sheltered animals, implementing preventive veterinary medicine protocols, and birth control.

The responsible party then mobilized a multidisciplinary team specialized in animal production, facilitating theoretical and practical discussions and the implementation of the actions recommended by the audit. The improvement process at the farm animal refuge was ongoing, respecting the specific needs of each stage of the cattle’s life. Based on the actions proposed by the audit, the responsible party implemented significant changes, including the construction of corrals adapted for rational training, the creation of a clinical area with space for hospitalizations, a well-equipped pharmacy, and a laboratory for low-complexity tests. In addition, the team of independent technical auditors monitored adherence to the health protocol, which included parasite control practices and the application of vaccines according to the categories of animals (22), intending to maintain collective health.

Operational changes at the FAR led to the development of standard operating procedures for various fronts of daily management, including enhancements in the sanitation of drinkers and the provision of higher-quality water for cattle. Nutritional management underwent significant improvements, with established schedules for providing diets adapted to the herd categories, and technical supervision of the quantity supplied, contributing to adequate body score (23, 24).

Considering the main population flow observed at the FAR between 2019 and January 2024, Figure 2 presents detailed data on cattle rescues, births, devolution owner, adoptions, and deaths. It also outlines the thematic axes addressed by the audit’s recommendations, offering a concise overview of the key issues identified throughout the ToC Fauna period. The most prominent recommendations pertain to animal welfare, followed by governance-related concerns linked to farm management decisions. Additional focus areas include human-animal interactions, particularly regarding the relationship between the cattle and both previous owners and potential adopters, and One Health considerations, emphasizing disease prevention and vaccination protocols within the herd.

**Figure 2:**
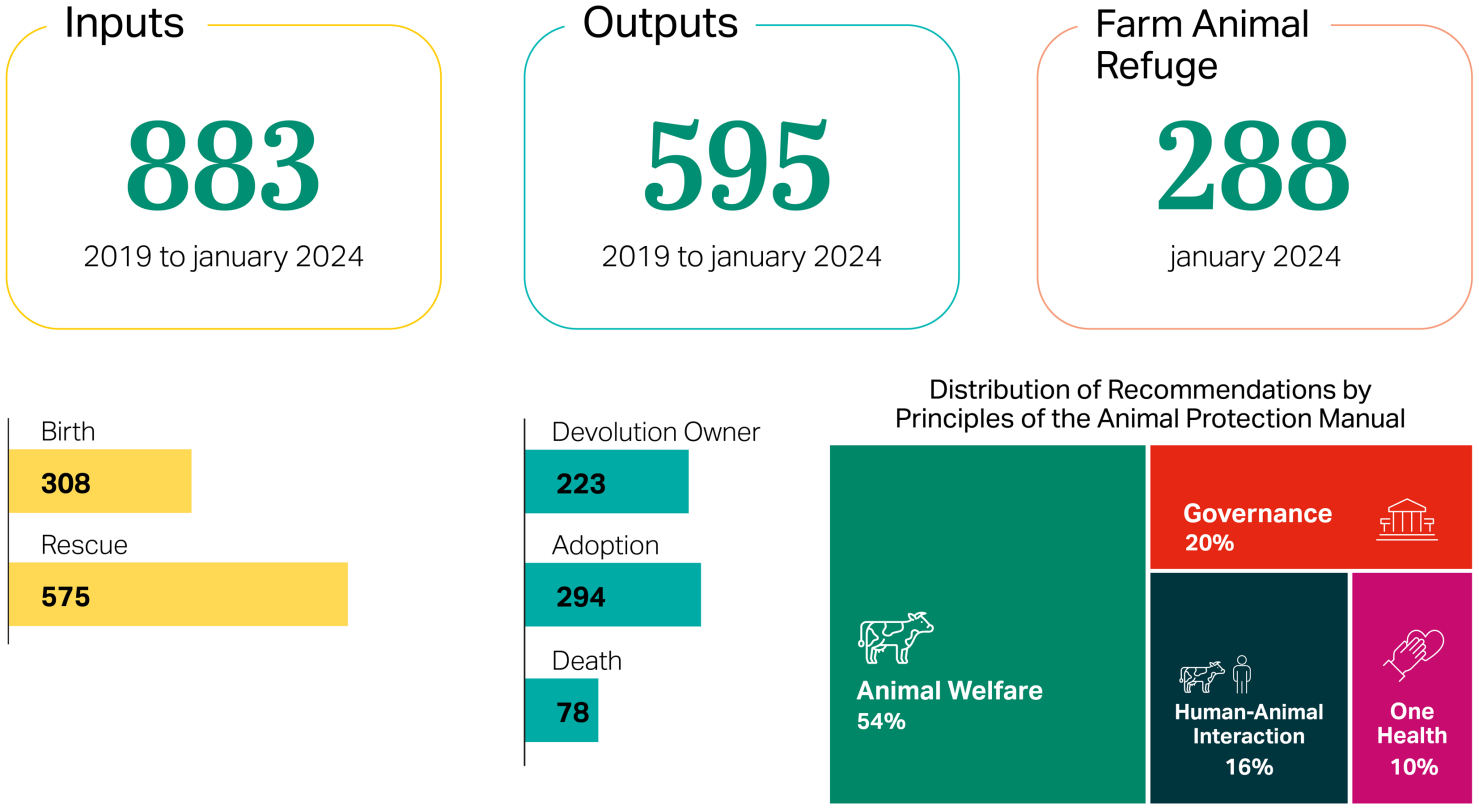
Consolidated population flow and thematic axes of the audit recommendations at the farm animal refuge between 2019 and 2024.

For this scenario, there was a paradigm shift in the formulation of diets for cattle with productive aptitude for milk and beef. Thus, the biggest challenge faced was adapting diets conventionally used in production systems to the particularities of keeping cattle on the FAR for an indefinite period (25, 26).

For dairy cattle, management was directed toward the animal’s ceasing production. Furthermore, another highlight was the birth control of sheltered animals, based on one of the clauses of the ToC Fauna that prohibits reproduction in the FAR. After 10 months of rescue and evacuation of animals, the birth rate on the FAR continued to increase. For this scenario, the audit recommended presenting a protocol to pursue the total absence of births. In this sense, the team of experts from the responsible party proposed the protocol based on reproductive biotechnology tools (27, 28), associated with field management practices (29), in addition to the total separation of males and females, resulting in the absence of new pregnancies after its implementation. It should be noted that during the evacuation and rescue process, some females were already in the gestational stage, and their pregnancy was assured.

Considering the premises of ToC Fauna, the welfare of sheltered animals has always been the pillar for all management actions carried out at the FAR. In these circumstances, the concept of behavioral medicine was first adopted by the predominant ACC model, originally rooted in shelter veterinary medicine and primarily intended for companion animals. In this article, we explore how this concept was later adapted for use with farm animals, particularly cattle. The implementation of this concept has contributed to significant advances in various areas of management, especially in recognizing animal behavior as a preventive medicine practice in shelters. Consequently, the responsible party implemented welfare monitoring for sheltered cattle, with attention to both individual and collective behavior.

Furthermore, the behavioral medicine protocol, which included environmental enrichment actions, was recommended, challenging the routine of the responsible party. This is a necessary condition to ensure the quality and psychological wellbeing of the animals. To achieve this purpose, the audit highlighted the extreme importance of maintaining monitoring of behavioral assessments and the application of environmental enrichment throughout the period the animals remain on the farm animal refuge.

**Figure 3:**
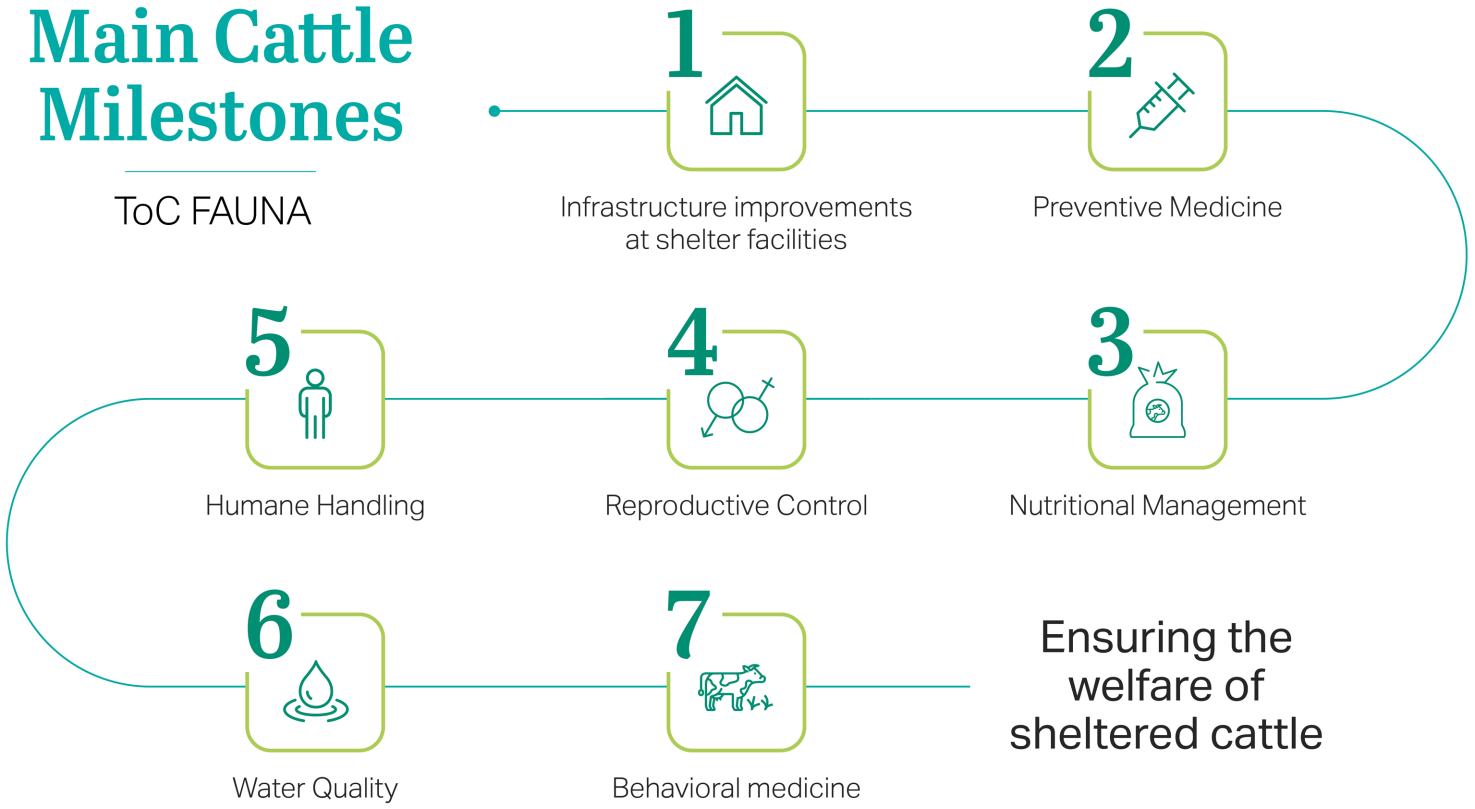
Flowchart of the main technical and operational milestones adopted at the farm animal refuge to ensure the welfare of sheltered cattle, based on an independent audit conducted under the scope of the fauna term of covenant.

Furthermore, the operational and technical milestones implemented at the FAR are summarized in Figure 3. To ensure the welfare of the sheltered cattle, specific protocols were developed and applied across key areas, including preventive medicine, nutritional management, reproductive control, humane handling, water quality, and behavioral medicine.

The implementation of environmental enrichment practices for cattle in semi-intensive systems presented significant challenges, primarily due to the structural limitations of the unit and the behavioral characteristics of the animals in this type of management. Unlike cattle in extensive systems, animals in semi-intensive systems can exhibit distinct behavioral patterns, requiring adapted environmental enrichment strategies that ensure both efficacy and safety for the animals and the handlers. In this context, behavioral assessment proved to be an indispensable tool for supporting decision-making and adapting enrichment practices in the shelter farm environment.

In response, the independent audit encouraged the adaptation of existing behavioral analysis methodologies and tools, aiming at adapting management practices for cattle in sheltered conditions. Throughout the validity of the ToC Fauna, the responsible party presented results of rational taming actions to correct behavioral deviations in bulls and other sheltered categories, making them suitable for adoption.

## 4 Discussion

Regarding companion animals, the reality of shelters in Brazil highlights the lack of specialized monitoring in shelter veterinary medicine, as well as the limited implementation of best practices in animal welfare and population management strategies. Moreover, deficiencies in the planning and management of these facilities are evident. These challenges arise from several factors, including limited knowledge of shelter medicine protocols, financial constraints, insufficient public subsidies, and inadequate management, all of which hinder the provision of appropriate care and the efficient allocation of available resources (13, 30). A similar situation is observed in countries such as Australia, where disaster planning has historically prioritized human interests, underestimating the needs and intrinsic value of companion animals (31).

For production animals, such as cattle, these arrangements are even more incipient, with the actions developed by the responsible party and the monitoring of the independent technical audit, through ToC Fauna, being a pioneering action in Brazil. The FAR for cattle overcame challenges by hiring specialized teams and maintaining animals at the farm animal refuge, while respecting the species’ characteristics and ensuring appropriate management practices.

Over the four-year duration of the ToC Fauna, a series of technical and administrative activities were carried out to enhance the management of the FAR and strengthen institutional governance. In total, 44 technical reports were issued, 24 field audits were conducted focusing on animal welfare and structural compliance, 34 technical sessions were held with specialists and responsible party, and 17 formal presentations were delivered to the MPMG. This set of activities demonstrates the continuous and systematic nature of the independent auditing process, reinforcing its role as a tool for transparency, social control, and improvement of disaster response protocols involving production animals.

In this scenario, the ToC Fauna became a positive example by fostering that animal rights were effectively protected and that protection measures were implemented rigorously and effectively. The independent technical audit played a crucial role in promoting wildlife protection actions, as it provided an impartial and specialized assessment of the practices used in the new system in which cattle are inserted. By reviewing the methods and procedures in detail, the parties involved in the ToC identified opportunities for improvement, gaps, or failures that could compromise the effectiveness of protection measures, and encouraged actions aligned with best practices and current standards.

The audit of a FAR aimed at the shelter of cattle rescued or evacuated from risk areas, involving multiple guardians, represented an unprecedented scenario in Brazil that required adapting existing theoretical frameworks. For this purpose, it was necessary to integrate animal production guidelines with the fundamentals of shelter veterinary medicine.

Although previous works have provided initial theoretical support for adapting shelter practices to cattle, including contributions on livestock behavior and handling by Grandin (32–34), technical guidelines from the Regional Council of Veterinary Medicine of Minas Gerais (35), and recent recommendations for sheltering animals in disaster contexts (13, 15), the direct observation conducted during technical field inspections proved to be indispensable for tailoring strategies to the specific realities of the audited environment. This practice enabled an understanding of the specificities of the audited environment and the proposal of strategies consistent with local reality.

The experience gained through the actions carried out by the responsible party is particularly noteworthy and provides a valuable opportunity to develop a field of specialized knowledge that can be applied in similar situations in the future. The practices and protocols developed in this context can serve as a reference for veterinary medicine in shelters and disasters, adapting to the specific needs of cattle and the shelter conditions, in addition to the possibility of replicating them in other production animal species in similar situations.

It is essential to note that, although specific publications on temporary refuge for cattle are limited, they are crucial for understanding the challenges and best practices in this field. These publications tend to address issues such as management, animal health, biosecurity, and environmental impact, all of which are essential to ensuring cattle welfare and the sustainability of animal shelters.

In summary, the experience of the responsible party at the farm animal refuge can be seen as a milestone in the animals shelter literature, providing valuable insights for the scientific community and professionals involved in disaster management, animal science, and shelter veterinary medicine.

## 5 Conclusion

The experience of implementing a Farm Animal Refuge in response to mining dam disasters in Brazil demonstrates that it is possible to adapt conventional cattle management practices to emergency contexts. Through the integration of veterinary protocols, nutritional strategies, behavioral monitoring, and reproductive control, the initiative ensured the health and welfare of rescued cattle over a four-year period. The role of independent technical auditing was essential in guiding and validating these actions, reinforcing the importance of science-based, transparent, and accountable approaches in disaster response. Importantly, all efforts were focused on maintaining the animals in non-productive systems, respecting species-specific characteristics and ensuring appropriate management practices.

This study contributes to the emerging field of disaster management for production animals, offering a replicable model that bridges gaps between animal science, shelter medicine, and environmental governance. The findings highlight the need for institutional frameworks that include livestock in emergency preparedness plans and recognize their welfare as a component of environmental justice. Future research should explore the scalability of the FAR model, its application to other species, and its integration into national and international disaster response protocols.

## Data Availability

The original contributions presented in the study are included in the article/Supplementary material, further inquiries can be directed to the corresponding author.
