# Supplementary material for: Auditing farm animal refuge: management and welfare of livestock in disaster situations
Source: Front Vet Sci. 2025 Oct 24;12:1681087. doi: 10.3389/fvets.2025.1681087 (PMC12591868; doi:10.3389/fvets.2025.1681087)
Supplement: Supplementary file 1 [file Data_Sheet_1.pdf]

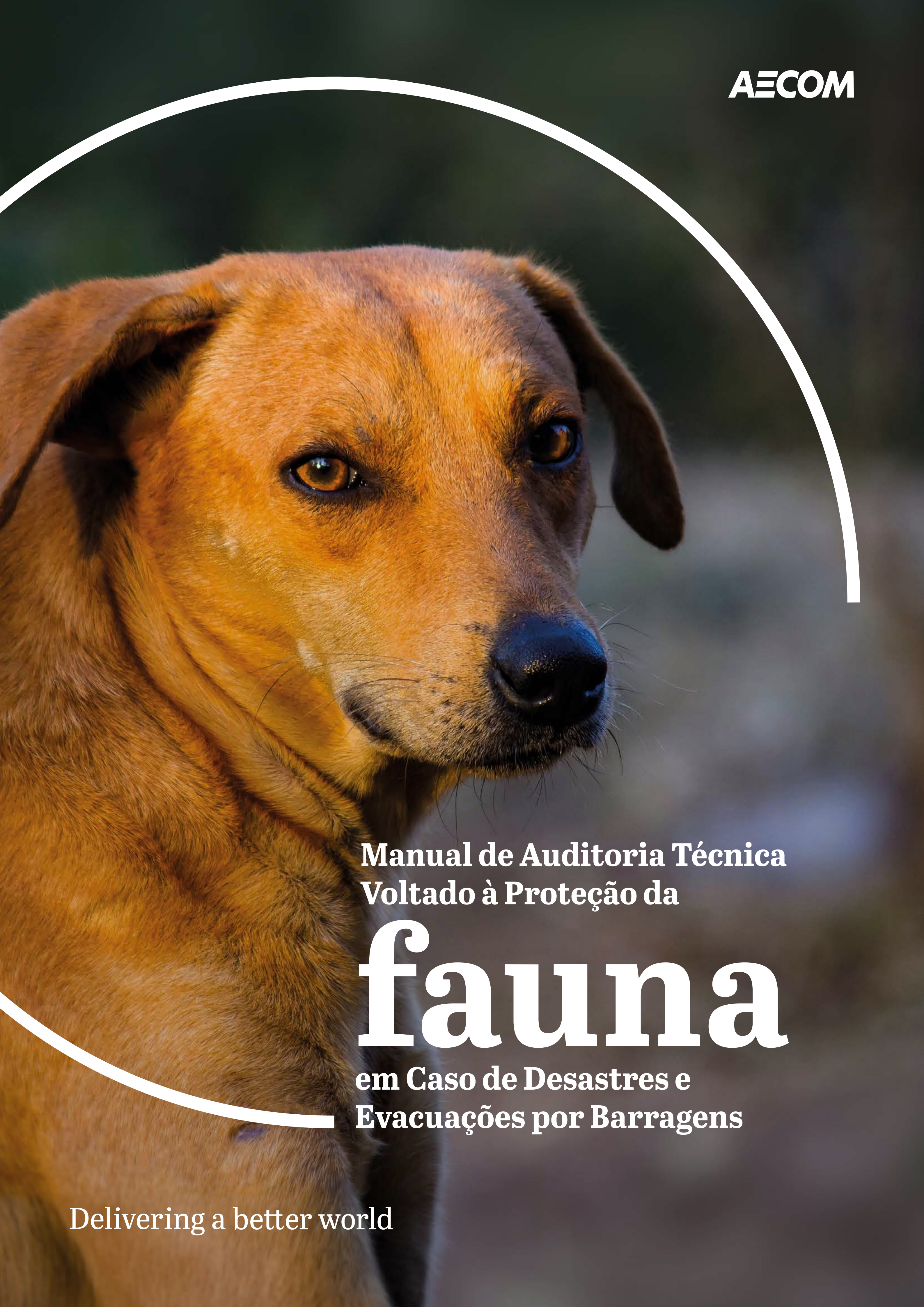

Manual de Auditoria Técnica Voltado à Proteção da fauna em Caso de Desastres e Evacuações por Barragens

Delivering a better world

AECOM

Manual de Auditoria Técnica Voltado à Proteção da fauna em Caso de Desastres e Evacuações por Barragens

Delivering a better world

Créditos

Presidente AECOM do Brasil
Vicente Mello

Diretoria Técnica
Alex Castro

Gerência Técnica
Felipe Bertelli

Coordenação Técnica
Gislene Fournier

Autores dos Capítulos
Carolina de Rezende Bonatto
Cássia Christina Vitorino Del Valle
Débora Bernardes de Andrade
Diego Garcia Ramirez
Felipe Bertelli
Gabriel Guerra Ferraz
Gislene Fournier
Graziela Virgínia Tolesano Pascoli
Manuella Folly
Roberta Ferreira Miranda
Sillas Silva
Tiago Carvalho de Morais
Vanessa Miranda Reis

Revisão de Texto
Graziela Virgínia Tolesano Pascoli
Gislene Fournier
Felipe Bertelli
Galpão33

Contribuição Técnica:
Gustavo de Morais Donancio Rodrigues Xaulim
Clarice Gomes Marotta

Diagramação:
Galpão33

Foto Capa
Robbie Ross

Catalogação na Publicação (CIP)

M294 Manual de auditoria técnica voltado à proteção da fauna em casos de desastres e evacuações por barragens / Carolina de Rezende Bonatto... [*et al.*] ; coordenação técnica Gislene Fournier. – Belo Horizonte : AECOM do Brasil, 2024.
127 p. : il. foto. color.

Vários autores.
Inclui bibliografia.
ISBN 978-65-985342-0-2

1. Animais – Proteção 2. Auditoria – Manuais, guias, etc. I. Bonatto, Carolina de Rezende II. Fournier, Gislene III. Título.

CDD: 591 (21ª ed.)

Bibliotecária responsável: Fernanda Gomes de Souza CRB-6/2472

"A todas as pessoas que dedicam suas vidas aos animais: vocês são uma fonte inesgotável de inspiração e sabedoria. Seu trabalho é fundamental para a construção de um legado de cuidado e respeito pelos nossos companheiros de jornada."

Agradecimento

A equipe de fauna da AECOM expressa a mais profunda gratidão a todos que participaram de alguma forma no desenvolvimento deste manual. A colaboração e dedicação de cada um, ao longo desses anos, consolidou uma ferramenta inédita que permitiu avaliar o bem-estar animal no contexto de desastres.

Agradecemos o apoio do Ministério Público de Minas Gerais (MPMG), sem o qual este manual não seria possível, em especial à Dra. Luciana Imaculada de Paula, por atuar em defesa da fauna e inspirar nossos esforços na busca pela proteção dos animais domésticos e silvestres. Também agradecemos ao Dr. Gustavo de Morais Donancio Rodrigues Xaulim e à Dra. Clarice Gomes Marotta, que compartilharam seus conhecimentos e experiências, contribuindo com sugestões que enriqueceram o conteúdo deste manual.

Gostaríamos de agradecer às equipes técnicas, aos coordenadores e gestores da VALE e demais empresas envolvidas, parabenizando-os por compreenderem e colaborarem com o processo pioneiro de auditoria para proteção da fauna no Brasil.

Agradecemos também aos nossos colegas da AECOM das diferentes áreas envolvidas pelo apoio ao longo do processo e, adicionalmente, pela informatização do manual em prol de sua aplicação.

Por fim, continuaremos trabalhando para melhorar e atualizar este manual para que as informações nele contidas possam ser aplicáveis em diferentes contextos da proteção animal.

Prefácio

Recebi, honrada, o convite para apresentar este manual subscrito pela admirável equipe da AECOM, que generosamente compartilha seu conhecimento e experiência adquiridos durante a auditoria de acordos tomados pelo MPMG e que estabelecem ações de proteção aos animais atingidos pelo rompimento de barragens em Brumadinho e por evacuações de comunidades em áreas de risco de diferentes barragens de mineração em Minas Gerais.

Rompimentos de barragens não são exatamente uma novidade em um lugar em que a mineração está presente, inclusive, em seu nome. Mesmo antes de sua constituição como unidade federativa, o território das Minas Gerais já era palco de intensa exploração de seu minério e o rescaldo dessa atividade continua, ainda nos dias de hoje, a reverberar na economia, na cultura, na paisagem e em cada cidadão mineiro, pois, nas palavras do poeta itabirano Carlos Drummond de Andrade, *cada um de nós tem seu pedaço no Pico do Cauê*.

No entanto, foi a partir dos desastres em Mariana e em Brumadinho que animais não humanos, de modo inédito e ostensivo, também foram considerados atingidos e mereceram ampla consideração nas ações de resposta pelas empresas responsáveis e pelos órgãos oficiais. Destaca-se que as ações de resgate, cuidado e destinação de animais foi objeto de termos de compromissos tomados pelo MPMG com a empresa responsável, sem realizar acepção entre nativos, exóticos, silvestres e domésticos, em superação ao paradigma especista dominante.

Entrementes, a Lei Mar de Lama Nunca Mais, como ficou conhecida a Lei Estadual nº 23.291/2019, de Minas Gerais, e a Resolução Conjunta SEMAD/FEAM/IEF/IGAM nº 3.181/2022, que a regulamenta, consolidaram a proteção dos animais na política estadual de segurança de barragens, prevendo que o resgate de animais deve compor o conteúdo mínimo dos Planos de Ação de Emergência para Barragens de Mineração (PAEBM).

O cumprimento dos acordos anteriormente mencionados resultou no resgate e abrigamento de milhares de animais, deflagrando-se o desafio premente de assegurar níveis satisfatório de bem-estar a todos os espécimes acautelados. Nesse contexto, a auditoria independente pela AECOM mostrou-se fundamental na construção, em conjunto com os órgãos públicos e a empresa compromissária, de procedimentos de manejo para espécies diversas, além da elaboração de indicadores para monitoramento dos resultados, sempre sob os postulados da Saúde Única e sem olvidar da dignidade intrínseca que cada animal carrega em si.

Nestas páginas, portanto, encontram-se consolidadas não apenas a legislação e a literatura científica de referência, mas também a experiência de fôlego, de mais de cinco anos, vivenciada pela equipe multidisciplinar da empresa auditora, a partir das intervenções bem-sucedidas em prol dos animais no contexto de emergência e resposta a desastres de mineração em Minas Gerais.

Embora desenvolvido com o escopo de atender as demandas específicas do TAC Fauna, o presente manual tem amplo potencial de replicabilidade, uma vez que seus postulados podem servir de referência para o manejo de animais abrigados em razão de emergência ou desastres, mas também em situação de normalidade, pelo serviço público ou por organismos da sociedade civil, com eventuais adaptações que se mostrarem convenientes.

O seu conteúdo, tendo em vista seu ineditismo e apuro técnico, certamente se inserirá na literatura de referência sobre o tema e muito contribuirá para a disseminação de boas-práticas em bem-estar animal.

Luciana Imaculada de Paula

Lista de figuras

Figura 1 | Os domínios do bem-estar animal (adaptado de Mellor e Beausoleil, 2015) 10

Figura 2 | Diferença entre perícia, consultoria e auditoria técnica 17

Figura 3 | Princípios, Critérios e Indicadores 19

Figura 4 | Definição dos princípios elencados para o Manual de Auditoria Técnica voltado à Proteção da Fauna em Caso de Desastres e Evacuações por Barragens 20

Figura 5 | Princípios e Critérios estabelecidos para o Manual de Auditoria Técnica voltado à Proteção da Fauna em Caso de Desastres e Evacuações por Barragens 21

Figura 6 | Nota do indicador considerando o método aplicado à auditoria técnica voltada à proteção da fauna 24

Figura 7 | Fases da operacionalização do método de auditoria técnica voltada à proteção da fauna 25

Figura 8 | Exemplo de Painel Gerencial aplicado a um abrigo de fauna 27

Figura 9 | Critérios avaliativos do Princípio Governança 30

Figura 10 | Critérios avaliativos do Princípio Do Preparo à Resposta 40

Figura 11 | Critérios avaliativos do Princípio Interação Humano e Animal 61

Figura 12 | Capacidade de Prover Cuidados em abrigos de fauna 72

Figura 13 | Critérios avaliativos do Princípio Bem-estar do Animal Abrigado 73

Figura 14 | Critérios avaliativos do Princípio Saúde Única 87

Figura 15 | Ocorrência de desastres no Brasil, 1993 – 2023 99

Lista de abreviaturas e siglas

ADA
Área Diretamente Afetada

AI
Área de Influência

AMR
Antimicrobial Resistance (Resistência Antimicrobiana)

ANM
Agência Nacional de Mineração

ANVISA
Agência Nacional de Vigilância Sanitária

APP
Área de Preservação Permanente

AR
Área de Referência

CETAS
Centro de Triagem de Animais Silvestres

CFMV
Conselho Federal de Medicina Veterinária

CONAMA
Conselho Nacional do Meio Ambiente

COPAM
Conselho de Proteção Ambiental

CPC
Capacidade de Prover Cuidados

CRMV
Conselho Regional de Medicina Veterinária

EPC
Equipamento de Proteção Coletiva

EPI
Equipamento de Proteção Individual

FEAM
Fundação Estadual do Meio Ambiente de Minas Gerais

FIOCRUZ
Fundação Oswaldo Cruz

FUNCAP
Fundo Nacional para Calamidades Públicas

GTA
Guia de Transporte Animal

IBAMA
Instituto Brasileiro do Meio Ambiente e dos Recursos Naturais Renováveis

IEF
Instituto Estadual de Florestas de Minas Gerais

IGAM
Instituto Mineiro de Gestão das Águas

MAPA
Ministério da Agricultura e Pecuária e Abastecimento

MCTIC
Ministério da Ciência, Tecnologia, Inovações e Comunicações

MMA
Ministério do Meio Ambiente e Mudança do Clima

MPMG
Ministério Público de Minas Gerais

ODS
Objetivos de Desenvolvimento Sustentável

OIE
Organização Mundial da Saúde Animal

ONU
Organização das Nações Unidas

PAE
Plano de Ação de Emergência

PAEBM
Planos de Ação de Emergência para Barragens de Mineração

PGRS
Plano de Gerenciamento de Resíduos Sólidos

PNSB
Política Nacional de Segurança de Barragens

POP
Procedimento Operacional Padrão

PTE
Plano de Tratamento de Efluentes

SEMAD
Secretaria de Estado de Meio Ambiente e Desenvolvimento Sustentável de Minas Gerais

SGBD
Sistema de Gerenciamento de Banco de Dados

TAC
Termo de Ajustamento de Conduta

TC
Termo de Compromisso

UC
Unidades de Conservação

Sumário

pág.

1. Apresentação 1

2. Introdução 3

2.1 Desastres: cenários e perspectivas sobre a fauna

2.2 Termo de Compromisso – TAC Fauna

3. Premissas da auditoria voltada à fauna 9

3.1 Senciência animal

3.2 Bem-estar animal

3.3 *Zooeyia* e família multiespécie

3.4 Direito e dignidade animal

4. Auditoria técnica voltada à proteção da fauna 17

4.1 A conduta do auditor técnico de fauna

4.2 Método de auditoria técnica voltado à proteção da fauna

4.2.1 Princípios

4.2.2 Critérios

4.2.3 Indicadores

4.2.4 Operacionalização

5. Princípio 1 – Governança 29

5.1 Critério I – Gestão

5.2 Critério II – Gestão de Informações e Banco de Dados

5.3 Critério III – Ações de Treinamentos e Aperfeiçoamento Profissional

6. Princípio 2 – Do Preparo à Resposta 39

6.1 Critério IV – Diagnósticos Ambientais e Fauna Silvestre

6.2 Critério V – Inventário da Fauna Doméstica, Exótica e Silvestre Cativa

6.3 Critério VI – Ações Preparatórias para Desastre

6.4 Critério VII – Ações de Resposta Emergencial

6.5 Critério VIII – Avaliação e Mitigação de Impacto à Fauna Atingida

6.6 Critério IX – Reabilitação e Reintrodução de Animais Silvestres

7. Princípio 3 – Interação Humano e Animal 61

7.1 Critério X – Ações Voltadas para Assegurar a Relação Humanos e Animais

7.2 Critério XI – Reintegração ao Tutor

7.3 Critério XII – Ações de Adoção e Pós-adoção

8. Princípio 4 – Bem-estar do Animal Abrigado 71

8.1 Critério XIII – Domínio Nutricional

8.2 Critério XIV – Domínio Ambiental

8.3 Critério XV – Domínio Sanitário

8.4 Critério XVI – Domínio Comportamental

8.5 Critério XVII – Domínio Mental

9. Princípio 5 – Saúde Única (*One Health* / Uma Só Saúde) 85

9.1 Critério XVIII – Natureza Biológica

9.2 Critério XIX – Natureza Física

9.3 Critério XX – Natureza Química

10. Perspectivas Futuras 99

11. Considerações Finais 103

12. Bibliografia 105

13. Glossário 119

Apresentação

A AECOM apresenta o Manual de Auditoria Técnica voltado à Proteção da Fauna em Caso de Desastres e Evacuações por Barragens baseado nas experiências de cinco anos de auditoria voltada ao bem-estar da fauna impactada pelo rompimento de barragens e por evacuações de comunidades em áreas de risco em função da mineração em Minas Gerais. Este manual foi elaborado por uma equipe multidisciplinar e oferece uma abordagem prática e informativa, combinando teoria (boas práticas e legislações) e exemplos reais de intervenções bem-sucedidas com os animais que foram atendidos no âmbito do TAC Fauna.

AECOM

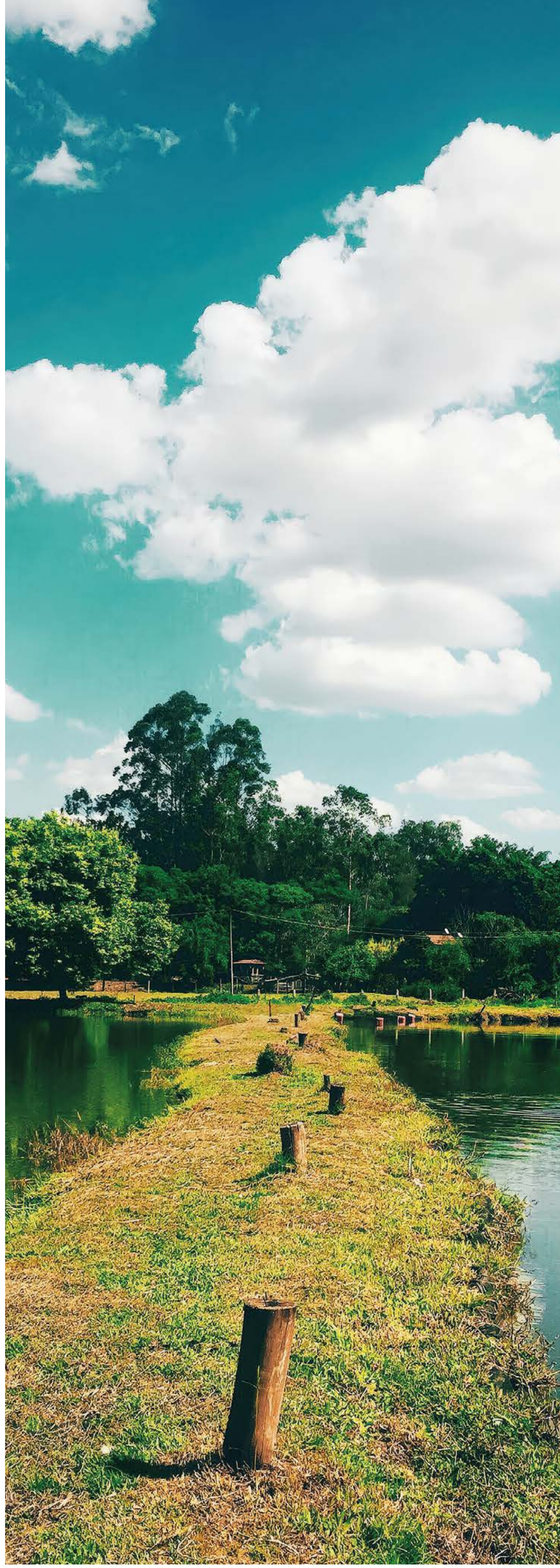

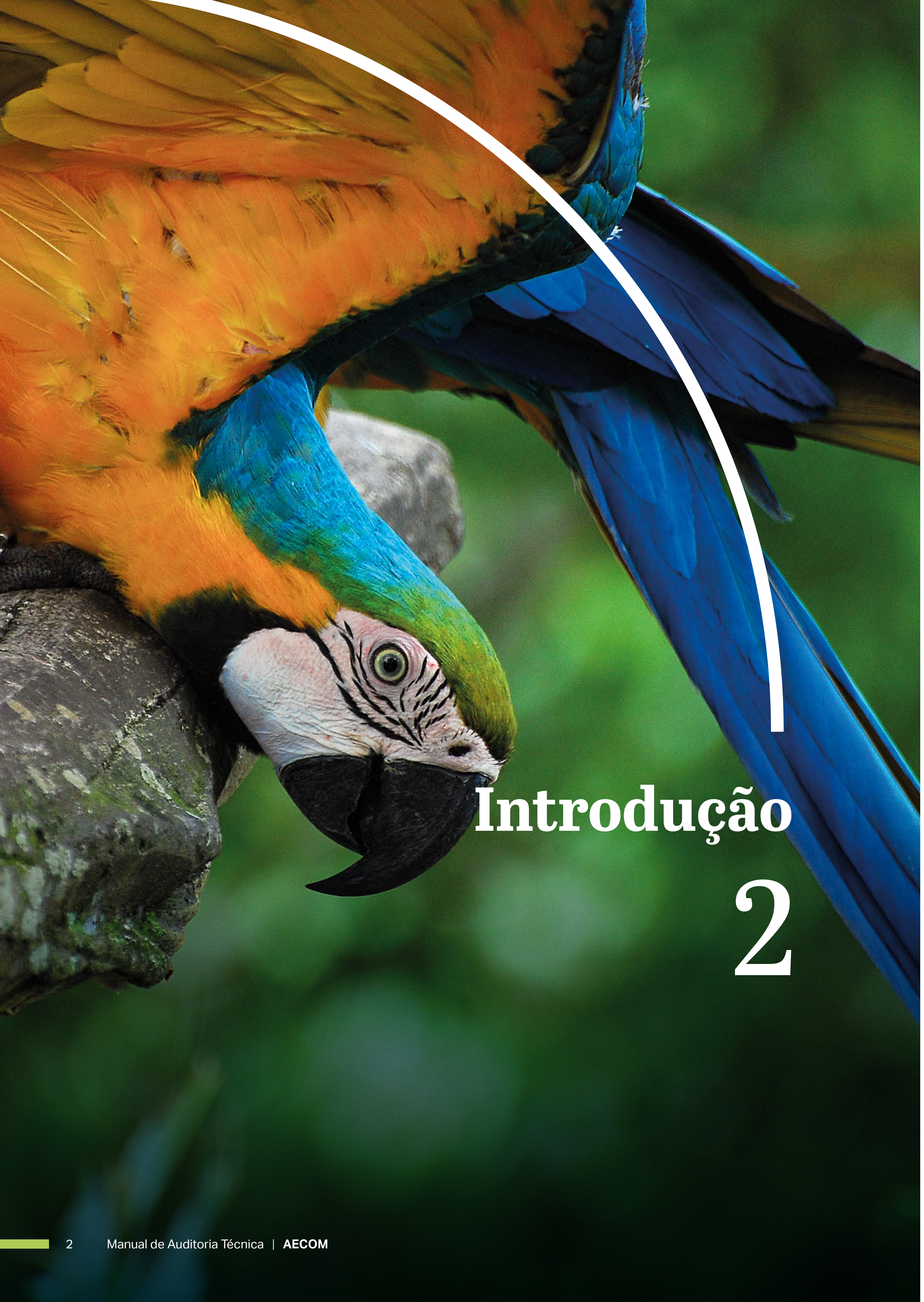

2. Introdução

2.1 Desastres: cenários e perspectivas sobre a fauna

O desastre é a consequência de um evento adverso, que pode resultar em danos e perdas humanas, materiais e ambientais, expondo a sociedade a uma situação de grande fragilidade. Para ser considerada desastre, a ocorrência do evento deve interromper o cotidiano e funcionamento de determinada comunidade, sociedade ou nação (UNISDR, 2016). Existem diferentes critérios para classificar um desastre, resumidamente, pela sua magnitude (acidentes, desastre de pequeno, médio e grande porte), tipo de evolução (súbito, crônico e de somação), frequência de ocorrência (recorrente e intermitente) e origem (natural, humano e/ou misto).

O desastre natural é provocado por fenômenos e desequilíbrios da natureza, independentemente da ação humana. Como exemplos, citam-se as secas, ciclones, terremotos, erupções vulcânicas, entre outros. Os desastres antropogênicos, por sua vez, ocorrem em função das ações humanas e podem ser subdivididos em três formas: os tecnológicos – gerados pelo uso inadequado de normas e princípios no âmbito da tecnologia dentro de um equilíbrio com o meio ambiente, repercutindo na forma de incêndios, colapsos estruturais, explosões – os sociais – que ocorrem pela falta de respeito aos princípios da liberdade, igualdade e fraternidade, sendo exemplos as guerras, violência, fome – e os biológicos – caracterizados pelo desequilíbrio nas ações humanas que impactam negativamente o ambiente e os seres vivos, como as epidemias (Araújo, 2021).

As leis e programas brasileiros definem a proteção das pessoas em casos de desastres naturais, como a Lei nº 12.340/2010 (Brasil, 2010), que sistematiza o Fundo Nacional para Calamidades Públicas (FUNCAP), proteção e defesa civil, e a Lei nº 12.608/2012 (Brasil, 2012), que institui a Política Nacional de Proteção e Defesa Civil. Já os órgãos ambientais possuem normativas que protegem os animais silvestres, como a Resolução CONAMA nº 01/1986 (Brasil, 1986) que trata do Estudo de Impacto Ambiental, a Lei nº 9.605/1998 (Brasil, 1998), que tipifica crimes ambientais e a Lei nº 9.966/2000 (Brasil, 2000) que preconiza ações em casos de derramamento de óleo (Gomes *et al.*, 2020).

Embora a Constituição Federal (1988) atribua ao Poder Público o dever de proteger a fauna e de colocar os animais a salvo de práticas cruéis (art. nº 225, §1º, VII), nada menciona sobre o resgate de animais domésticos, tanto os de companhia quanto os utilizados para produção, em caso de desastres. Cada vez mais são necessários esforços para atuar nessa área e elaborar estratégias utilizando uma abordagem multidisciplinar na gestão de riscos de desastres, seja ela de maneira corretiva ou prospectiva em prol dos animais domésticos (PNDEC, 2012).

Os desastres ocasionados pelas mudanças climáticas trazem preocupação para diversas áreas, incluindo a resiliência de barragens. No Brasil, há pelo menos 10 mil barragens de pequeno a grande porte. A maior parte delas foi construída com base em estudos hidrológicos que consideram séries históricas de dados mais antigos ou com poucos registros recentes de eventos extremos. Ao se pesar as incertezas associadas a métodos estatísticos na área da hidrologia, utilizados para o dimensionamento de estruturas hidráulicas de barragens, faz-se necessária uma avaliação quanto ao risco admitido para as estruturas frente a eventos extremos.

Dessa forma, as ações mitigatórias para adaptar as barragens às chuvas mais intensas, considerando seu tamanho e/ou classificação, devem ser priorizadas. Os rompimentos/extravasamentos de barragens que vêm ocorrendo nos últimos anos no país têm atingido dimensões cada vez maiores, com consequências diretas nos âmbitos econômicos e socioambientais, requerendo a incorporação de ações de salvaguarda de animais (Ramos *et al.*, 2021; Souza, 2023; MAB, 2024).

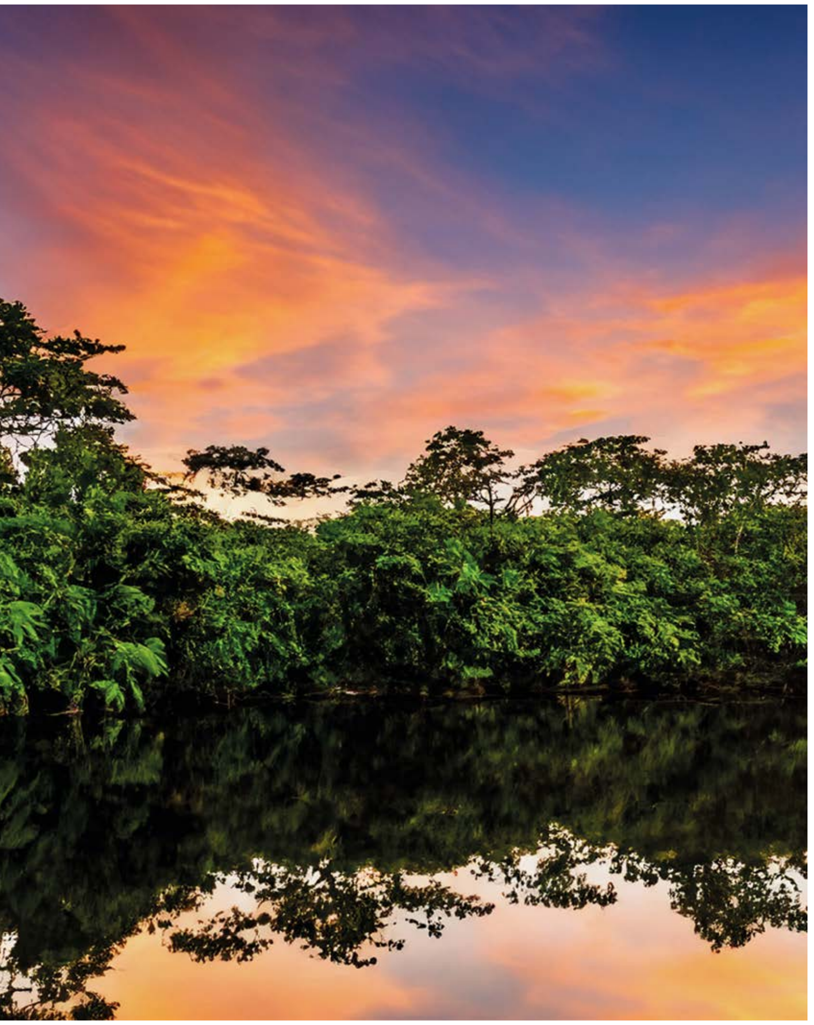

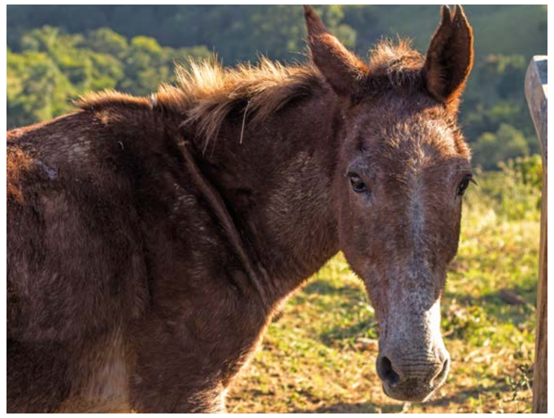

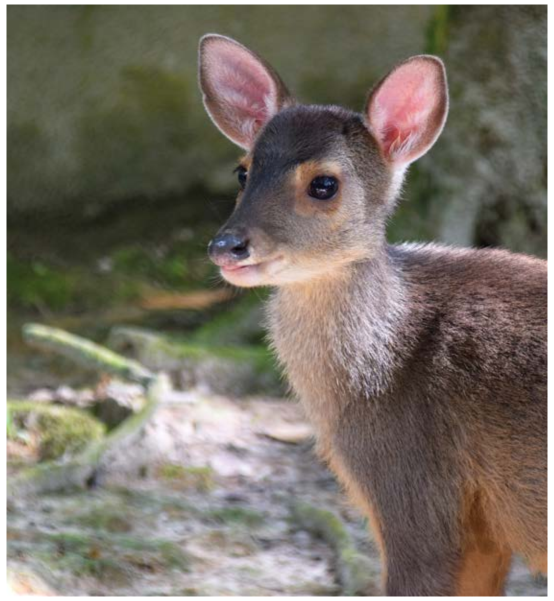

O aumento na ocorrência de desastres e de suas consequências impõe a necessidade de desenvolver mecanismos de proteção dos ecossistemas naturais e da biodiversidade, assim como na aplicação de investimentos para ampliar a resiliência das infraestruturas urbanas e industriais. Para animais domésticos, a elaboração de orientações, diretrizes e ações voltadas para pré e pós-desastres está em expansão no Brasil, principalmente após 2019. Diante dessa nova perspectiva e das crescentes populações de animais domésticos e de produção no território brasileiro, este manual reúne o conhecimento adquirido pela equipe técnica da AECOM, atuando por cinco anos na área de auditoria do bem-estar da fauna oriunda de resgates e evacuações emergenciais.

2.2 Termo de Compromisso – TAC Fauna

Em 2015 e 2019, o Brasil passou por dois grandes desastres por rompimento de barragens de mineração, ambas localizadas no estado de Minas Gerais. O colapso dessas estruturas geotécnicas causou danos ambientais e socioeconômicos imensuráveis, também para animais da fauna doméstica e silvestre.

A partir desses eventos, a possibilidade de novos colapsos levou à evacuação emergencial de diferentes comunidades situadas em áreas de risco de outras barragens mineiras. Evidencia-se, no entanto, que em meio aos desastres de grande repercussão humanitária e econômica, a situação dos animais muitas vezes acaba secundária e, dessa forma, permanece subnotificada.

Nesse contexto de desastres envolvendo a mineração e dos impactos diretos à vida de animais ocorreu uma iniciativa inédita no Brasil (e possivelmente no mundo), imputando a uma grande mineradora, responsável por dezenas de barragens, obrigações legais específicas para a proteção de animais em situação de risco pela presença dessas estruturas. O Ministério Público de Minas Gerais (MPMG) estabeleceu um Termo de Compromisso (TC) para assegurar que o empreendedor assumisse a responsabilidade pelo bem-estar de todos os animais atingidos por barragens, independentemente da espécie, e determinou o acompanhamento de uma auditoria técnica independente para verificar a aderência ao objeto do acordo judicial, nomeado como TAC Fauna.

No âmbito do TAC Fauna, a mineradora comprometeu-se a realizar o resgate, abrigar pelo tempo necessário e fornecer cuidados médicos-veterinários (incluindo castração de cães e gatos) para todos os animais evacuados das áreas de risco por barragens em nível de emergência , e àqueles atingidos pelo rompimento das barragens ocorrido em Brumadinho, Minas Gerais. Além disso, deveria identificar os antigos tutores de cada animal e reintegrá-los, promover programas de adoção para os animais sem tutores identificados, dentre outros compromissos. Ao assumir a função de auditora, a AECOM atuou no acompanhamento das diversas atividades desenvolvidas pelo empreendedor, identificando riscos e direcionando a auditada a antecipar as resoluções de problemas de forma objetiva, além de avaliar os controles e processos da organização como forma de prevenção de outros riscos potenciais.

[1] Os níveis de Emergência e de alerta são convenções utilizadas pela Resolução da Agência Nacional de Mineração (ANM) nº 175/2024 para graduar as situações de emergência em potencial que possam comprometer a segurança da barragem, variando na escala crescente de risco em "Sem Emergência", "Nível de Alerta", "Nível de Emergência 1", "Nível de Emergência 2" e "Nível de Emergência 3".

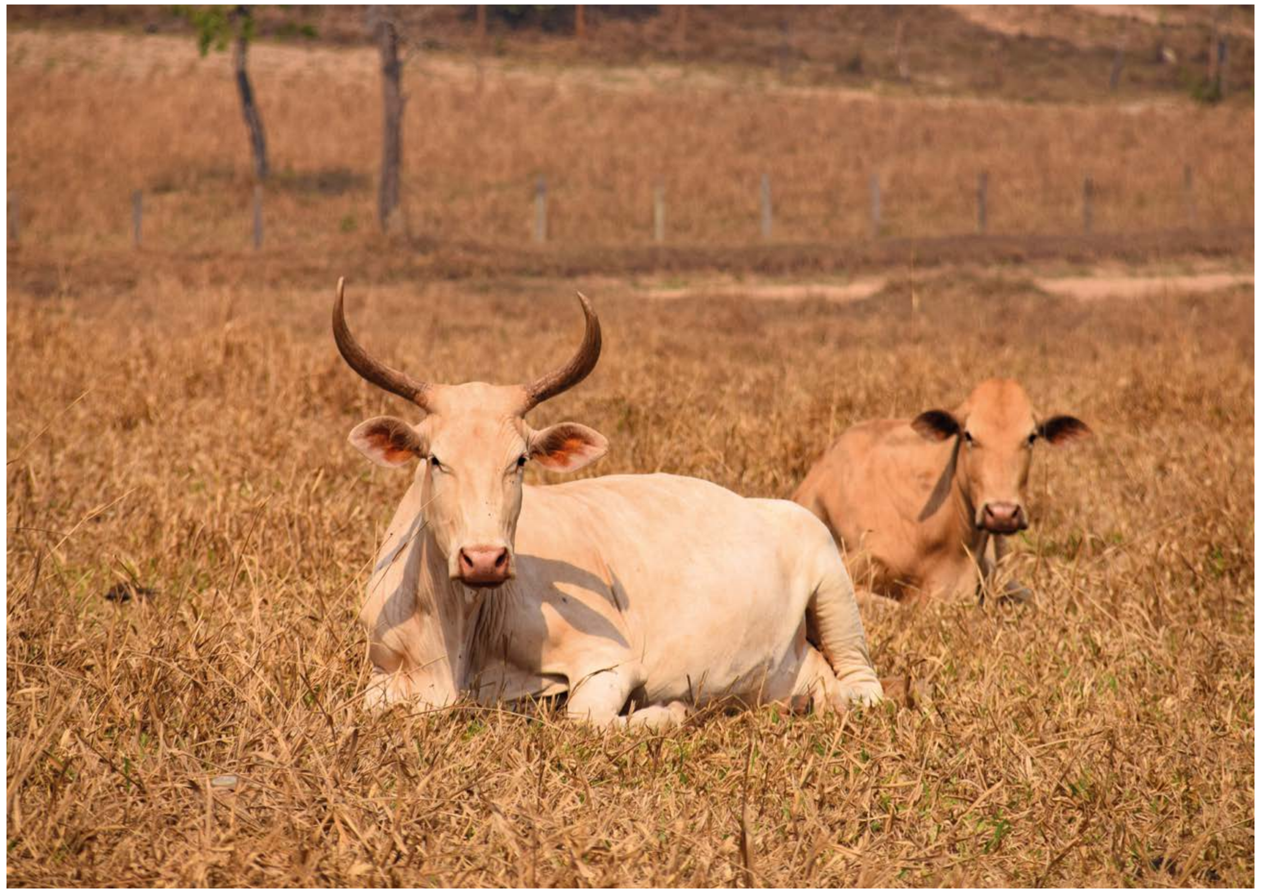

Para tanto, a AECOM desenvolveu um método de auditoria para avaliar as ações voltadas à proteção animal no contexto de desastres oriundos de barragens e inundações, com o objetivo de minimizar a subjetividade na avaliação de aderência ao escopo do TAC Fauna, tendo como premissas fundamentais o bem-estar animal e os princípios da saúde única. Neste contexto, o Manual de Auditoria Técnica voltado à Proteção da Fauna em Caso de Desastres e Evacuações por Barragens foi desenvolvido com diretrizes básicas e procedimentos necessários para a realização de auditoria a partir de evidências documentais geradas pelo empreendedor, e com avaliações realizadas *in loco* pela equipe de auditores. A literatura de referência é embasada nas leis, diretrizes e normativas brasileiras, publicações técnico-científicas nacionais e internacionais que proporcionaram fundamentação para a criação dos Princípios e Critérios avaliativos que compõem este manual.

Considerando a contemporaneidade do objeto de auditoria e visando a aplicabilidade deste método, é pressuposta a revisão periódica dos seus indicadores, sempre que houver necessidade. Assim sendo, é importante salientar que o arcabouço legal envolvendo a proteção dos animais em situação de desastres foi incrementado no Estado de Minas Gerais, por meio da revisão dos instrumentos legais que definem o conteúdo mínimo dos Planos de Ação de Emergência para Barragens de Mineração (PAEBM) e dos novos marcos na legislação que rege o licenciamento e a operação de atividades minerárias, com destaque para a publicação da Resolução Conjunta SEMAD/FEAM/IEF/IGAM nº 3.181/2022 (Minas Gerais, 2022). Desta forma, caso a auditoria técnica voltada à proteção da fauna seja aplicada em outros estados brasileiros, alguns dos itens auditáveis poderão ser adaptados conforme a legislação onde se encontra a organização auditada.

Apesar de desenvolvido inicialmente para atender a necessidade de auditoria do TAC Fauna, a proposta de manual de auditoria técnica voltada à proteção da fauna traz consigo o legado do aprendizado da avaliação das ações de manejo e bem-estar de fauna. Nesse contexto, a possibilidade de replicação da abordagem proposta neste documento em outros cenários de ameaça à segurança ao bem-estar de animais foi sendo desenvolvida paulatinamente à execução das atividades de auditoria. Este manual representa um legado da visão da equipe AECOM para os animais no contexto de emergência e resposta a desastres, construído a partir da experiência prática de uma equipe multidisciplinar de especialistas, da visão de vanguarda do Ministério Público de Minas Gerais, das novas publicações de saúde animal e do objetivo de promover os princípios de dignidade animal em diferentes esferas da sociedade.

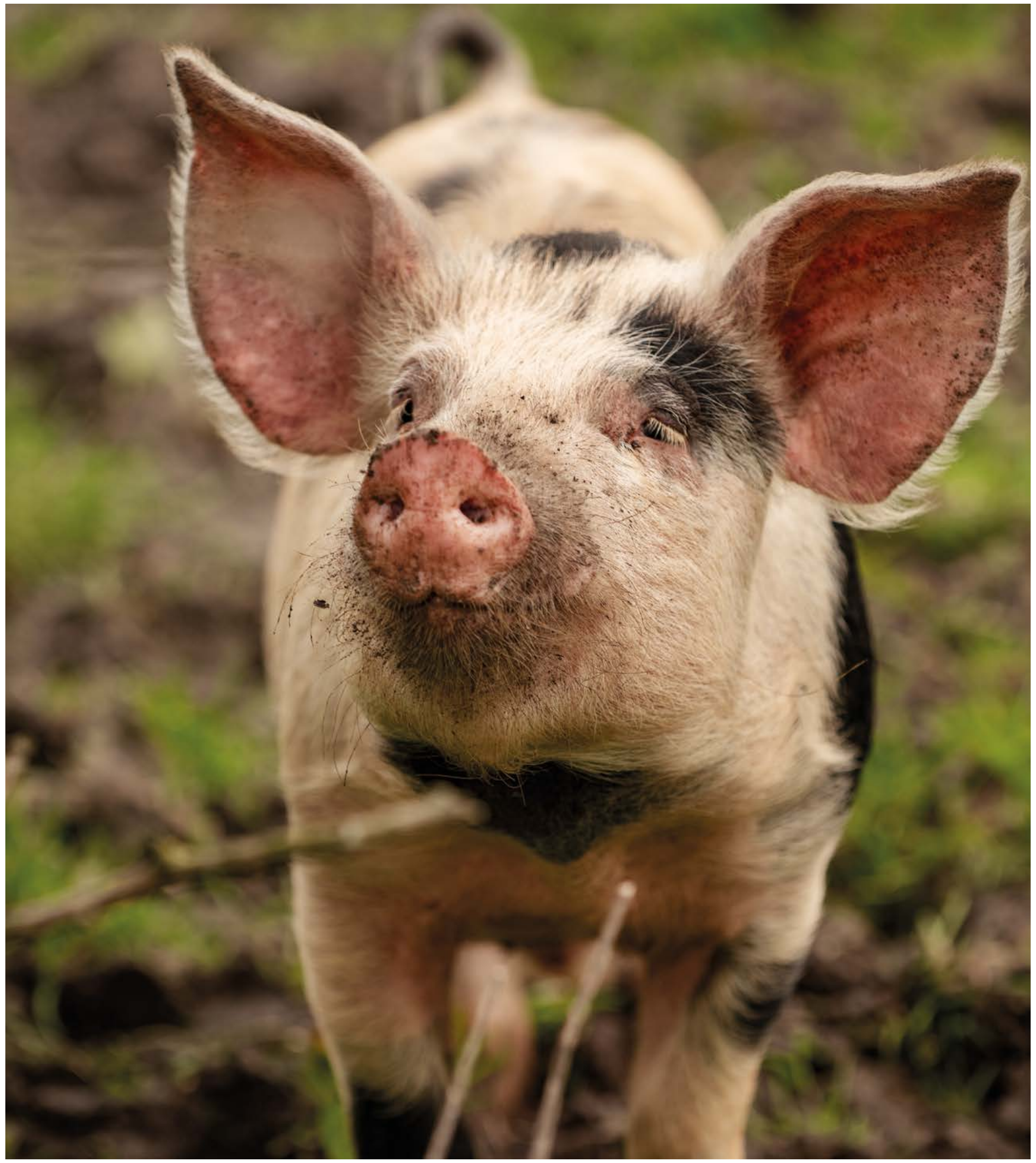

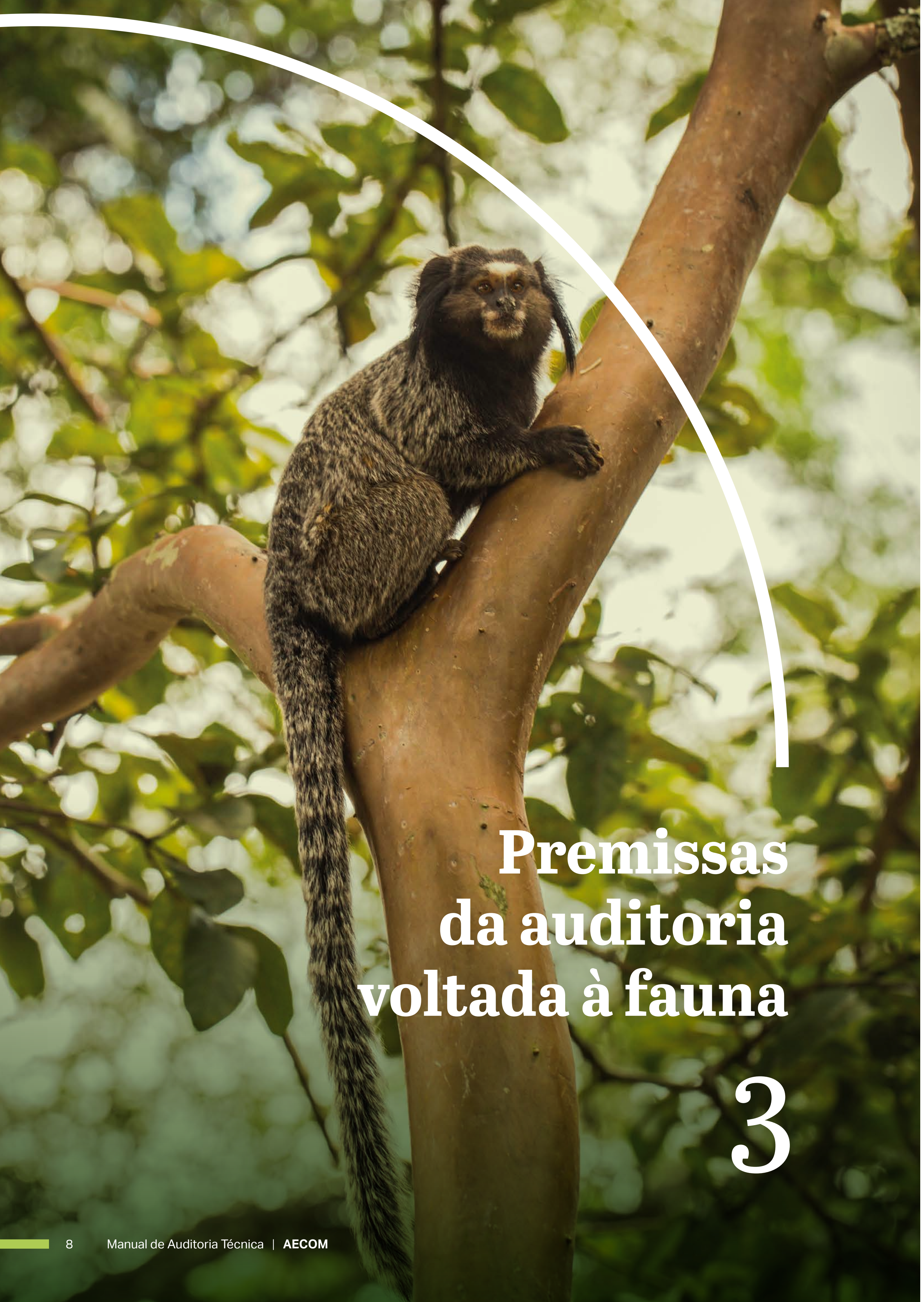

3. Premissas da auditoria voltada à fauna

3.1 Senciência animal

A senciência é o nível mais profundo de consciência, ou seja, é a capacidade de sentir conscientemente as sensações mais básicas em relação ao entorno (Domingos e Souza, 2019). Segundo a Organização *Animal-Ethics* (2024), "*a senciência é a capacidade de ser afetado positiva ou negativamente. É a capacidade de ter experiências*". A ciência demonstra que todos os animais dotados de um sistema nervoso central são potencialmente sencientes e que, para muitas espécies, sua evidência é robusta por razões comportamentais, evolutivas e, em particular, fisiológicas. Atualmente, reconhece-se a senciência em todos os animais vertebrados, em cefalópodes e em alguns crustáceos (Andrews *et al.*, 2024).

Sendo assim, fica claro que todos os seres que podem ter experiências positivas e negativas têm interesse em serem afetados de forma positiva e não de forma negativa. Em outras palavras, almejam ter o seu bem-estar maximizado e o seu sofrimento minimizado. Portanto, assim como os humanos, todas as espécies sencientes têm interesse em não sofrer, são capazes de adquirir experiências e de desfrutar das suas vidas. A capacidade de desfrutar suas vidas implica, entre outras coisas, que eles têm interesse em continuar vivos, para que, assim, tenham a oportunidade de ter experiências positivas (Bonella, 2012).

Diante disso, o reconhecimento de tal capacidade nos animais tem profundas implicações éticas e morais, trazendo à tona discussões importantes sobre bem-estar animal em sistemas produtivos e fins científicos, aprofundamento do papel social dos animais de companhia e dos desafios da conservação ambiental e dos animais silvestres.

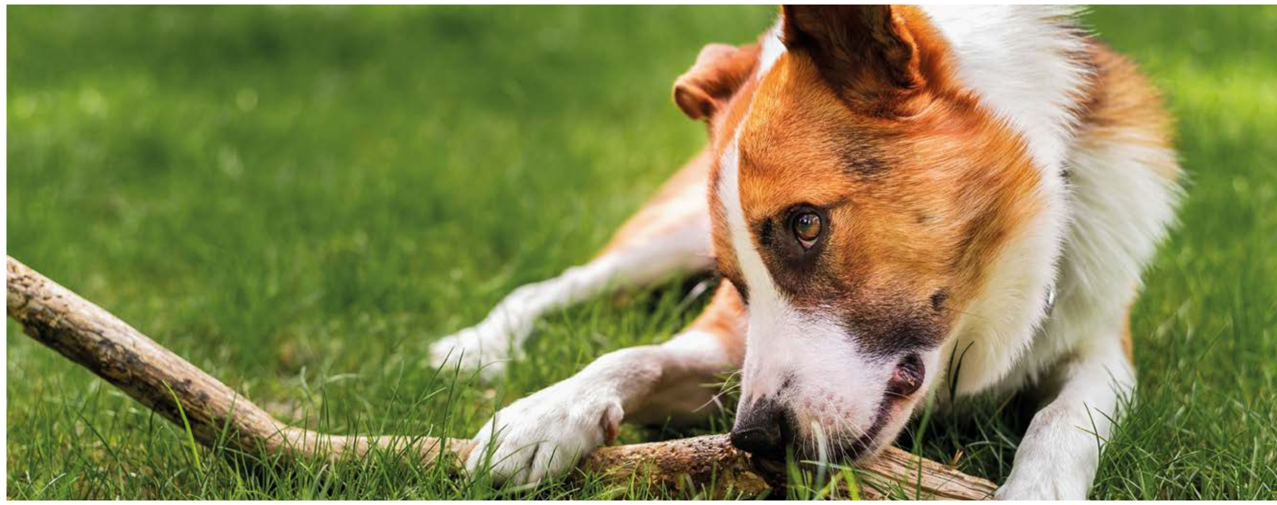

3.2 Bem-estar animal

O bem-estar animal está intimamente relacionado à saúde animal, à saúde e ao bem-estar das pessoas e à sustentabilidade dos sistemas socioeconômicos e ecológicos, sendo prioridade nas principais instituições e órgãos mundiais que são referência em manejo animal, conservação da biodiversidade e saúde única (OIE, 2016, 2017; WHO, 2019). Este é um tema complexo e multifacetado com dimensões científicas, éticas, econômicas, jurídicas, religiosas e culturais, além de albergar importantes implicações em políticas comerciais.

A compreensão da motivação, cognição e complexidade do comportamento social em animais fez surgir a ciência do bem-estar animal, que se desenvolveu amplamente nos últimos 30 anos (Broom, 2011). Atualmente a avaliação sobre nível adequado de bem-estar animal considera uma série de quesitos, que estão associados às condições clínicas (saúde física) e comportamentais (saúde mental) de cada indivíduo. A avaliação completa do *status* de bem-estar animal contempla a observação de evidências de que o indivíduo demonstra estar: saudável, confortável, bem nutrido, seguro, sem lesão aparente ou dor e se é capaz de expressar comportamentos naturais que são importantes para seu estado físico e mental (Blokhuis *et al.*, 2013; Barnard *et al.*, 2016).

Indo além, o modelo dos Cinco Domínios, conceito adaptado por Mellor e Beausoleil (2015) a partir da proposta das Cinco Liberdades, permitiu acomodar os aspectos positivos e negativos dos estados internos e funcionais, como a nutrição, ambiente, saúde e comportamento com os estados mentais (fatores psico afetivos). Os Cinco Domínios são reconhecidos como uma abordagem que utiliza métricas estruturadas para identificar ações específicas necessárias para promover o bem-estar de animais sob tutela humana, e avaliar a sua eficácia (Figura 1).

Domínios do bem-estar animal.

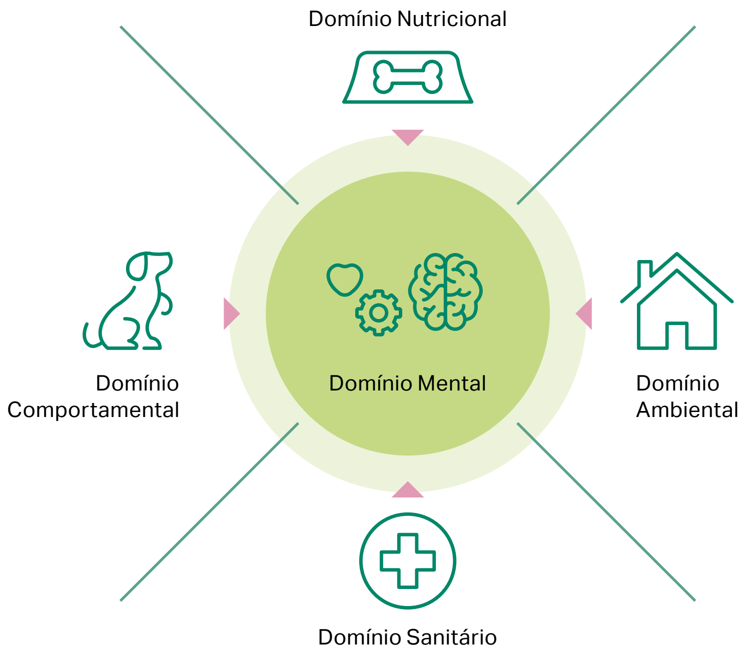

Figura 1 – Os domínios do bem-estar animal (adaptado de Mellor e Beausoleil, 2015).

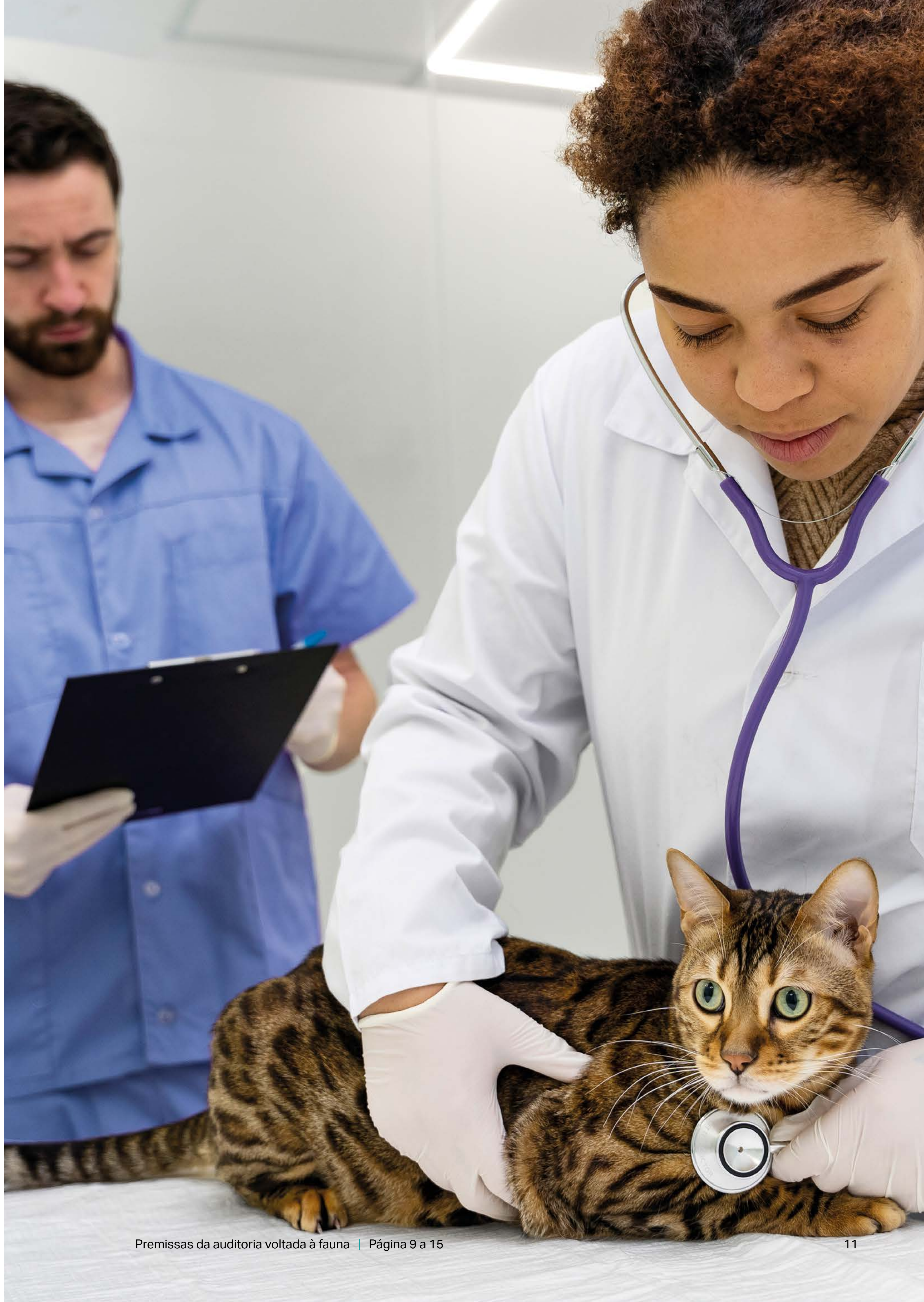

Dessa forma, o bem-estar animal adequado requer prevenção de doenças e cuidados veterinários apropriados, abrigo, manejo e nutrição espécie-específicos, um ambiente estimulante e seguro, manejo humanitário e, quando necessário, acesso à eutanásia de forma ética. Ressalta-se que desses preceitos de bem-estar também devem ser aplicados à fauna aquática, nativa ou exótica, de vida livre ou em sistemas de produção (Gomes *et al.*, 2020; WOAH, 2021).

A auditoria técnica voltada à proteção da fauna preconiza o uso de métodos de manejo voltados às características biológicas de cada espécie, assim como o abrigo em um ambiente adequado para atender suas necessidades e expressão de seu comportamento natural, incluindo ações específicas para estimular experiências positivas durante o período de estadia em abrigo (Faraco *et al.*, 2021). Em situações de desastre ambiental, o bem-estar começa com o manejo adequado para cada espécie durante as atividades de resgate e o atendimento médico-veterinário emergencial (Gomes *et al.*, 2020; Oliveira *et al.*, 2020; Gebara *et al.*, 2024).

3.3 *Zooeyia* e família multiespécie

O termo *zooeyia* (do grego: *zoion* significa animal; e *Hygeia* significa saúde, a antiga deusa da saúde) foi proposto pelas pesquisadoras Hodgson e Darling (2011) para denominar os benefícios que os animais de companhia trazem para suas famílias humanas, sendo posteriormente confirmado que o impacto positivo se expande para as esferas física, emocional e comunitária (Hodgson *et al.*, 2015).

O vínculo humano-animal foi definido como um relacionamento dinâmico entre pessoas e animais, de modo que cada um influencia o estado psicológico e fisiológico do outro. Esse relacionamento é influenciado por comportamentos que são essenciais para a saúde e o bem-estar de ambos, incluindo interações emocionais, psicológicas e físicas entre pessoas, animais e o ambiente em que vivem (Williamson *et al.*, 2022).

As ciências que albergam esta área do conhecimento são a zooantropologia e a bioética animal, que seguem a tese continuísta que considera que seres humanos e não-humanos possuem diferenças de grau e não de natureza, estendendo a noção de pessoa também aos animais. O amadurecimento das pesquisas em zooantropologia e bioética estão revolucionando o entendimento da interação entre pessoas e animais nas esferas filosófica, ética e pedagógica (Leira *et al.*, 2017).

Pesquisas sobre o elo entre pessoas e seus cães são muito recentes, mas confirmaram o conhecimento empírico de muitos tutores, que os cães têm uma influência profunda na vida de sua família, principalmente quando há uma conexão afetiva aos animais de companhia. Por outro lado, o vínculo com humanos provê estado emocional positivo (relaxamento e segurança) e afeta o bem-estar desses animais a longo prazo, pois aumenta sua resiliência ao estresse (Rault *et al.*, 2020).

Compartilhar a vida com um animal de companhia tem sido associado a uma diminuição do risco de doença arterial coronariana, redução dos níveis de estresse e aumento da atividade física (Williamson *et al.*, 2022; Xie *et al.*, 2017). A presença de um animal de companhia durante atividades estressantes já foi demonstrada como fator de diminuição de ansiedade entre casais que participam dessas tarefas (Allen, 1998). A interação humano-animal também pode ser considerada estimulante de interações sociais, nas quais as pessoas que gostam de animais se associam. Portanto, abriu espaço para novas discussões sobre legislações para proteção, posse responsável e bem-estar animal (Carver, 2019).

Além disso, uma nova concepção surgiu no campo do direito da família e direito dos animais, o conceito de família multiespécie, que reconhece legalmente as relações afetivas e convivência entre humanos e animais de estimação como integrantes da unidade familiar. Dessa forma, o direito da família multiespécie assegura que as mesmas questões éticas e legais sejam aplicadas para todos os membros da família, como por exemplo, guarda e custódia, proteção e bem-estar, herança e legado (Rodrigues *et al.*, 2022). Este é um reflexo de um crescente movimento ético e jurídico em direção a uma sociedade mais justa e compassiva, incorporando conceitos como *zooeyia*, família multiespécie e dignidade animal.

Concomitantemente, surgiram os conceitos de dignidade animal e direito animal com significativas discussões para assegurar que todo ser senciente não seja tratado como propriedade humana e sim como sujeitos de direito (Gomes, 2010; Silva *et al.*, 2020).

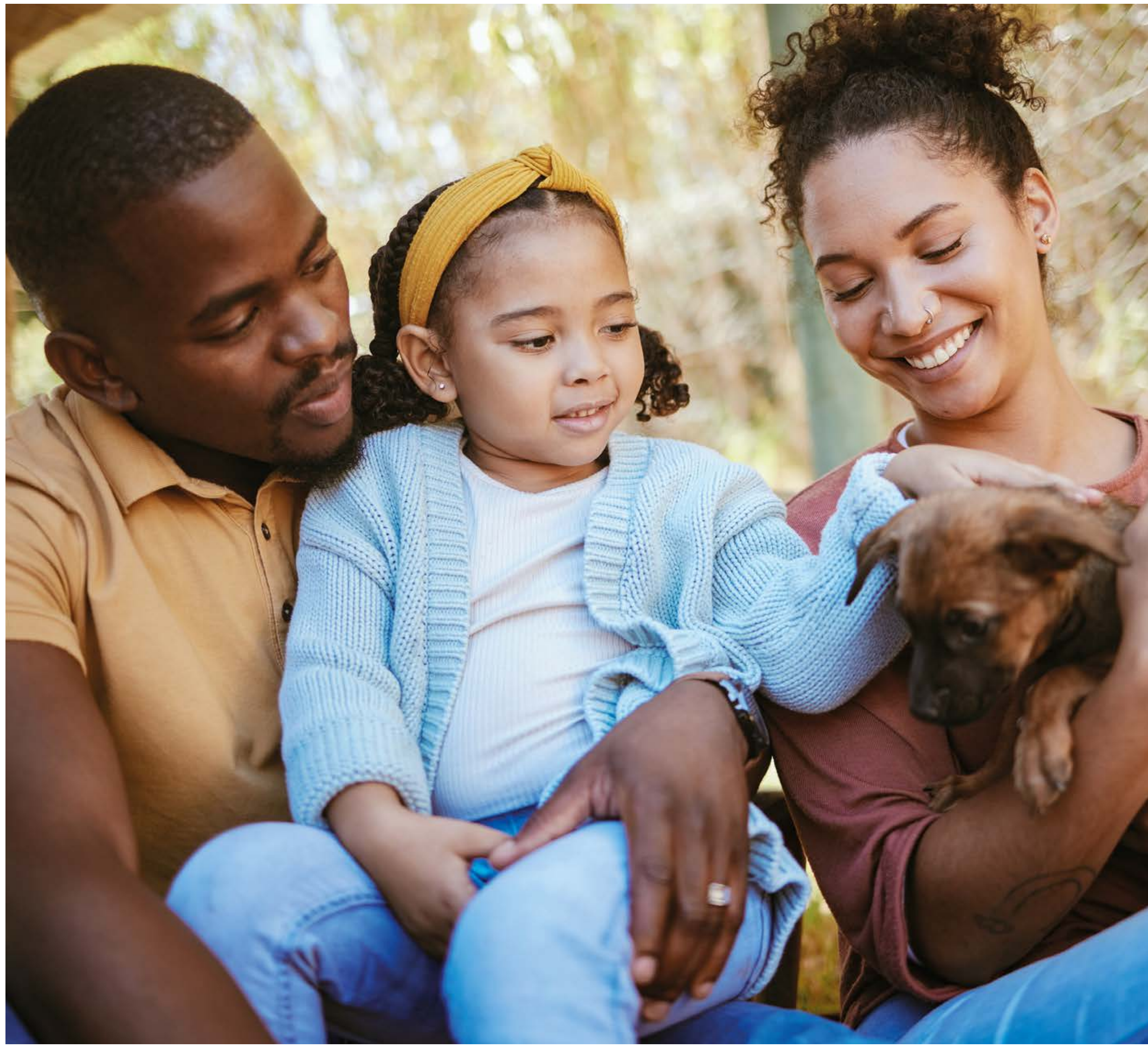

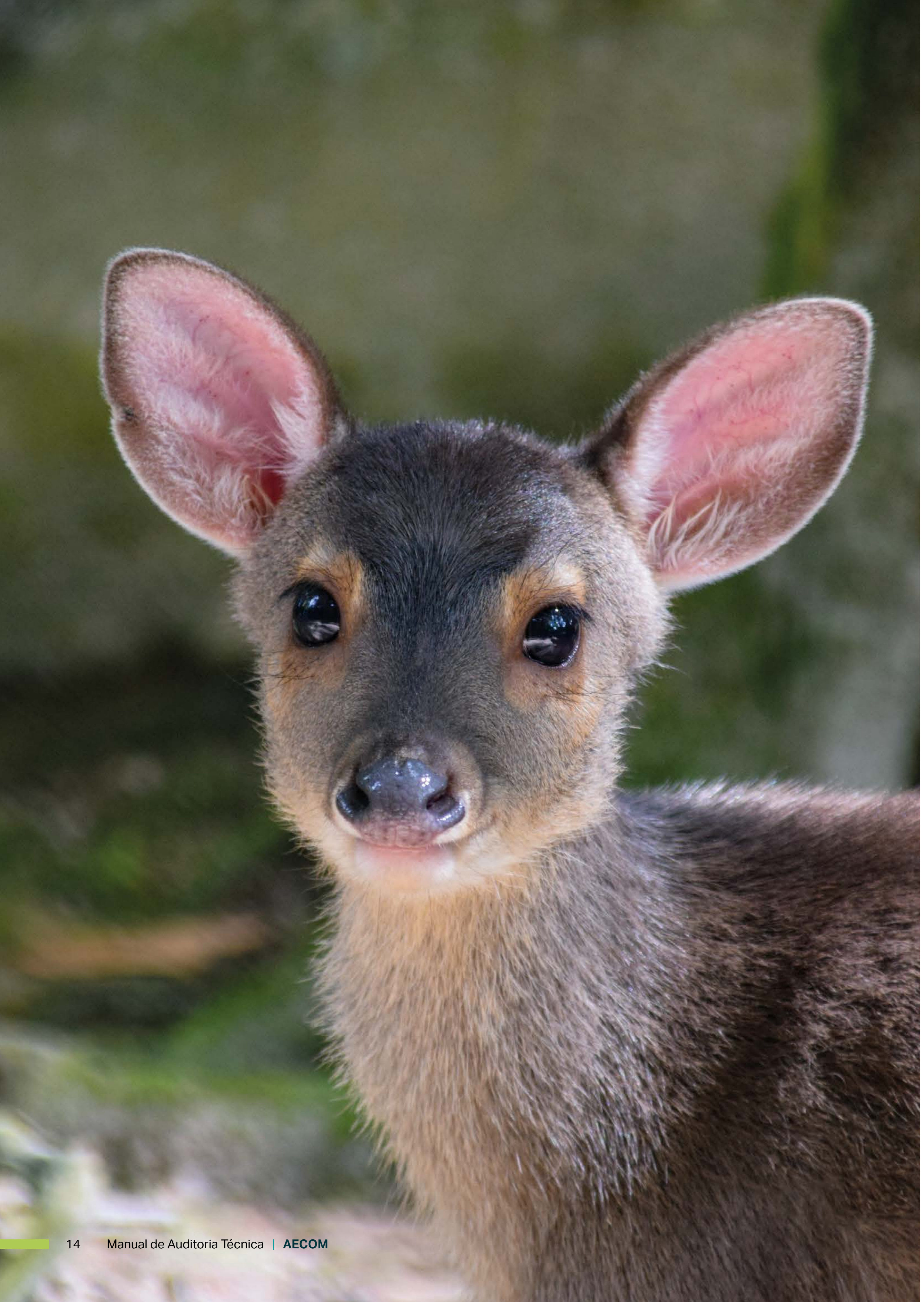

3.4 Direito e dignidade animal

O direito animal pode ser definido como o conjunto de regras e princípios que estabelece os direitos dos animais não humanos, reconhecendo-os por si próprios, independentemente de sua função ecológica, econômica ou científica. Em outras palavras, os animais não são objetos de direitos, mas sim sujeitos de direitos (Ataide Júnior, 2022). Sua base legal compreende o art. nº 32 da Lei nº 9.605/1998 e alterada pela Lei nº 12.305/2010 (Brasil, 2010) e a Resolução CFMV nº 1.236/2018 (Brasil, 2018).

O conceito de animais como sujeitos de direito e o princípio de igual consideração são fundamentados na ética animal e nos direitos naturais, reconhecendo que os animais possuem direitos próprios que devem ser respeitados e protegidos por meio de legislações, levando em conta de forma equitativa os interesses humanos (Gomes, 2010; Souza, 2018; Silva *et al.*, 2020; Carneiro *et al.*, 2023; Ataide Júnior; Lima, 2024). Esse desenvolvimento também exige uma mudança nas percepções culturais e um compromisso contínuo da sociedade com a implementação efetiva dos direitos dos animais (Carneiro *et al.*, 2023).

A ética animal argumenta que os animais são seres sencientes, capazes de sentir dor e prazer, portanto, devem ser tratados com respeito e dignidade. Os direitos naturais, por sua vez, afirmam que os animais têm direitos inerentes, semelhantes aos direitos humanos, derivados de sua própria natureza e capacidade de sofrer (Gomes, 2010; Souza, 2018; Silva *et al.*, 2020).

Assim, surge o conceito de dignidade animal, que reconhece o valor intrínseco dos animais, independentemente de sua utilidade para os seres humanos, assegurando que tenham direito à vida, direito ao bem-estar e o direito à liberdade (Ataide Júnior e Lima, 2024; Carneiro *et al.*, 2023).

No contexto da ética ambiental, a incorporação da dignidade animal está intrinsecamente ligada à preservação dos ecossistemas e à saúde planetária, pois ambientes saudáveis e biodiversos são essenciais para a sobrevivência e ao bem-estar de diversas espécies. Portanto, a degradação ambiental e a destruição da biosfera podem ser interpretadas como violações da dignidade animal (Carneiro *et al.*, 2023).

Corroborando essa teoria, a Declaração de Nova York sobre a Consciência Animal (*The New York Declaration on Animal Consciousness*), assinada em 19 de abril de 2024 (Andrews *et al.*, 2024), é um grande marco na ciência animal, reconhecendo três importantes pontos: **1)** Todos os mamíferos e aves são conscientes; **2)** Todos os vertebrados e diversos invertebrados (moluscos cefalópodes, crustáceos decápodes e insetos) são dotados de consciência; **3)** Todos os seres conscientes têm direito ao bem-estar e a viver uma vida que merece ser vivida.

Como exemplo no Brasil, o governo paraibano ampliou a legislação estadual, permanecendo na vanguarda do tema em relação aos outros estados brasileiros. O Código de Direito e Bem-Estar Animal do Estado da Paraíba foi instituído pela Lei nº 11.140/2018 (Paraíba, 2018), com entrada em vigor no dia 7 de outubro de 2018. Os animais, segundo o art. nº 2 da Lei paraibana, são "*seres sencientes e nascem iguais perante a vida, devendo ser alvos de políticas públicas governamentais garantidoras de suas existências dignas, a fim de que o meio ambiente, bem de uso comum do povo e essencial à sadia qualidade de vida dos seres vivos, mantenha-se ecologicamente equilibrado para as presentes e futuras gerações*". Uma das mudanças mais significativas é o crescente reconhecimento de que os animais não podem mais ser tratados como meros objetos ou coisas. Ao adaptar as leis a essa mudança de paradigma, os legisladores demonstram compromisso com uma ética mais humanitária e com a promoção de uma convivência mais justa e respeitosa entre humanos e animais.

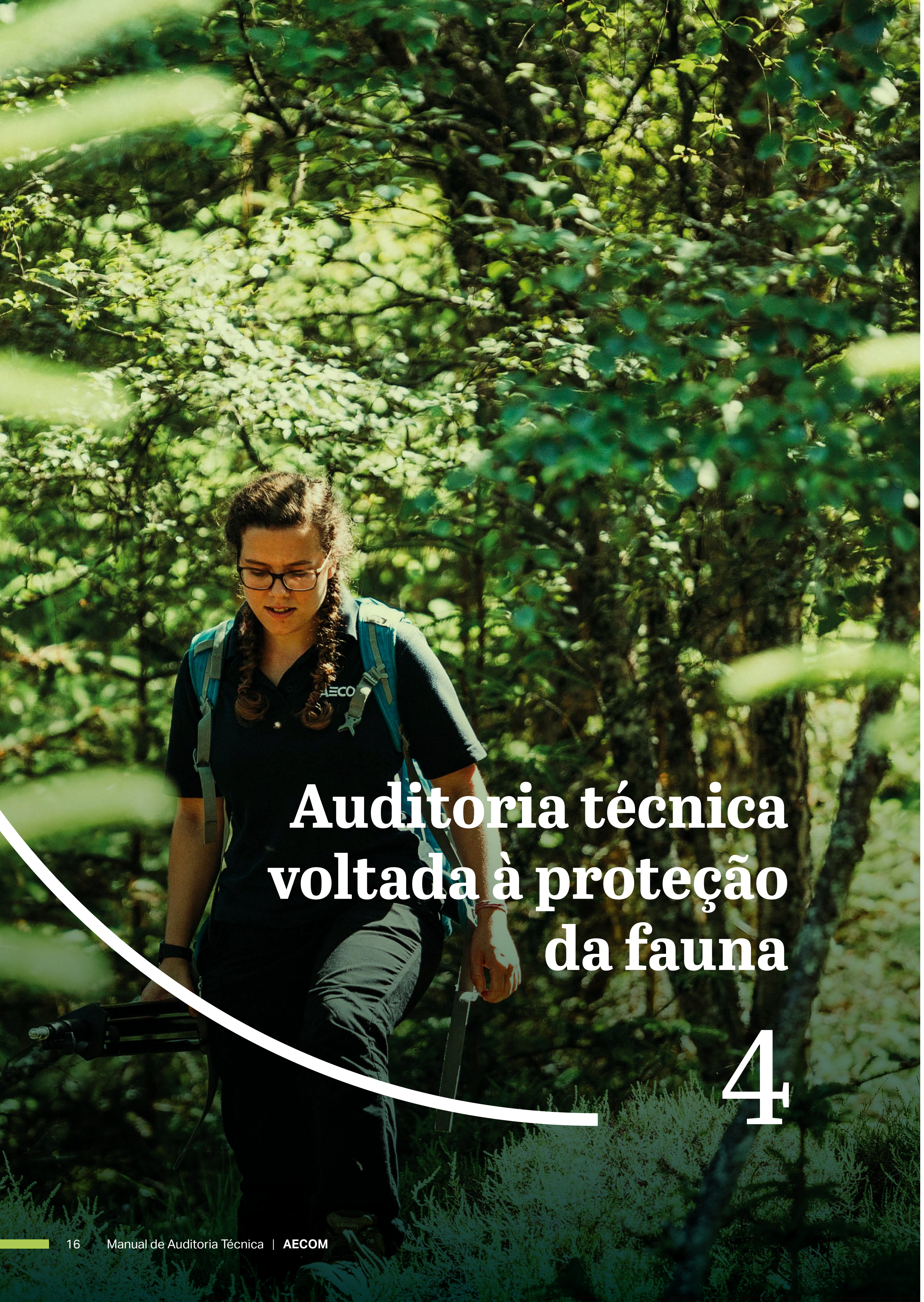

4. Auditoria técnica voltada à proteção da fauna

4.1 A conduta do auditor técnico de fauna

Inicialmente, faz-se necessária a distinção entre as atividades de auditoria técnica, consultoria e perícia ambiental, conforme descrito na Figura 2.

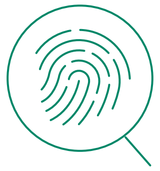

Perícia

se prende ao caráter científico de uma prova com o objetivo de esclarecer controvérsias. É específica, restrita aos quesitos e pontos controvertidos, especificados pelo condutor judicial. Sua análise é irrestrita e abrangente.

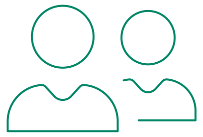

Consultoria

se prende ao caráter de aconselhar. Com a capacidade de interpretar um cenário, diagnosticar uma situação e apontar os caminhos e os métodos mais apropriados para sua correção.

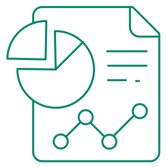

Auditoria Técnica

se prende ao caráter de examinar e atestar a integridade e veracidade das ações. Não tem vínculo direto com a empresa auditada e, por ser independente, seus resultados são isentos de interesse ou influência, portanto, mais fidedignos.

Figura 2 – Diferença entre perícia, consultoria e auditoria técnica.

A auditoria técnica é fundamentada em cuidados e condutas profissionais que devem ser seguidas pelos auditores, de forma a prover eficácia, confiança e isonomia em todo o processo de auditoria, gerando informações concludentes.

A função do auditor é avaliar o atendimento aos padrões da auditoria técnica de maneira objetiva, imparcial e racional, considerando normas técnicas, publicações científicas, instrumentos jurídicos e boas práticas, além de documentar sistematicamente as evidências coletadas através do acompanhamento das atividades e dos processos. A sua conduta deve ser pautada no fundamento do profissionalismo que considera imparcialidade, ética, honestidade e responsabilidade, dentro das premissas de legalidade, impessoalidade, publicidade e integralidade.

O auditor técnico de fauna deve possuir conhecimentos e habilidades específicas focadas no bem-estar e na saúde animal, permitindo avaliar desde a estrutura do local até o comportamento dos indivíduos para detecção de possíveis anormalidades. É imprescindível que o auditor tenha uma visão integralista que permita reconhecer os riscos e vulnerabilidades sociais a que os animais estão sujeitos em cenários de desastres, mas também a resiliência das pessoas e as possibilidades colaborativas que as diversas situações emergenciais demandam.

Além disso, deve atuar de forma objetiva, atentando-se à confidencialidade e à discrição voltadas para a segurança da informação, uma vez que, em função da natureza do trabalho, o auditor pode ter acesso a procedimentos e informações comerciais e estratégicas potencialmente sensíveis ao auditado. Deve ser, também, capaz de fazer julgamentos ponderados, de acordo com a importância de cada situação no processo da auditoria.

Os indicadores devem ser examinados minuciosamente, com base em evidências objetivas e passíveis de verificação, de acordo com os preceitos estabelecidos pela ciência e pelo direito animal, considerando a legislação municipal, estadual e federal. É relevante o conhecimento prévio dos objetivos da auditoria, de questões externas e internas pertinentes, da organização auditada, além das expectativas dos *stakeholders* envolvidos.

É essencial que as auditorias sejam realizadas em dupla ou em equipe de auditores, evitando a possibilidade de individualização da avaliação (pessoalidade). As constatações e conclusões da auditoria, bem como os relatórios emitidos, devem refletir com veracidade e precisão as atividades desenvolvidas durante o período de execução dos trabalhos. Para isso, a comunicação estabelecida deve ser verdadeira, precisa e objetiva, realizada em tempo hábil, de forma clara e completa.

Com o avanço constante das tecnologias e das regulamentações ambientais, sanitárias e de proteção da fauna, é crucial que o desenvolvimento do auditor seja contínuo. Suas competências e aperfeiçoamento profissional devem ser tratados como prioridade, a fim de permitir que desenvolva habilidades críticas para a resolução de problemas complexos. Portanto, investir no aprimoramento técnico dos auditores não só melhora a qualidade das auditorias, mas também contribui para a proteção da fauna, das pessoas, do meio ambiente e o cumprimento das normas legais.

4.2 Método de auditoria técnica voltado à proteção da fauna

Este manual visa padronizar as práticas de auditoria técnica voltada à proteção da fauna, construindo um método replicável e capaz de ser adaptado a diferentes objetos relacionados ao tema. O método proposto visa diminuir a subjetividade durante o processo avaliativo.

Dessa forma, o manual é organizado em Princípios, Critérios e Indicadores, sendo estes assim definidos:

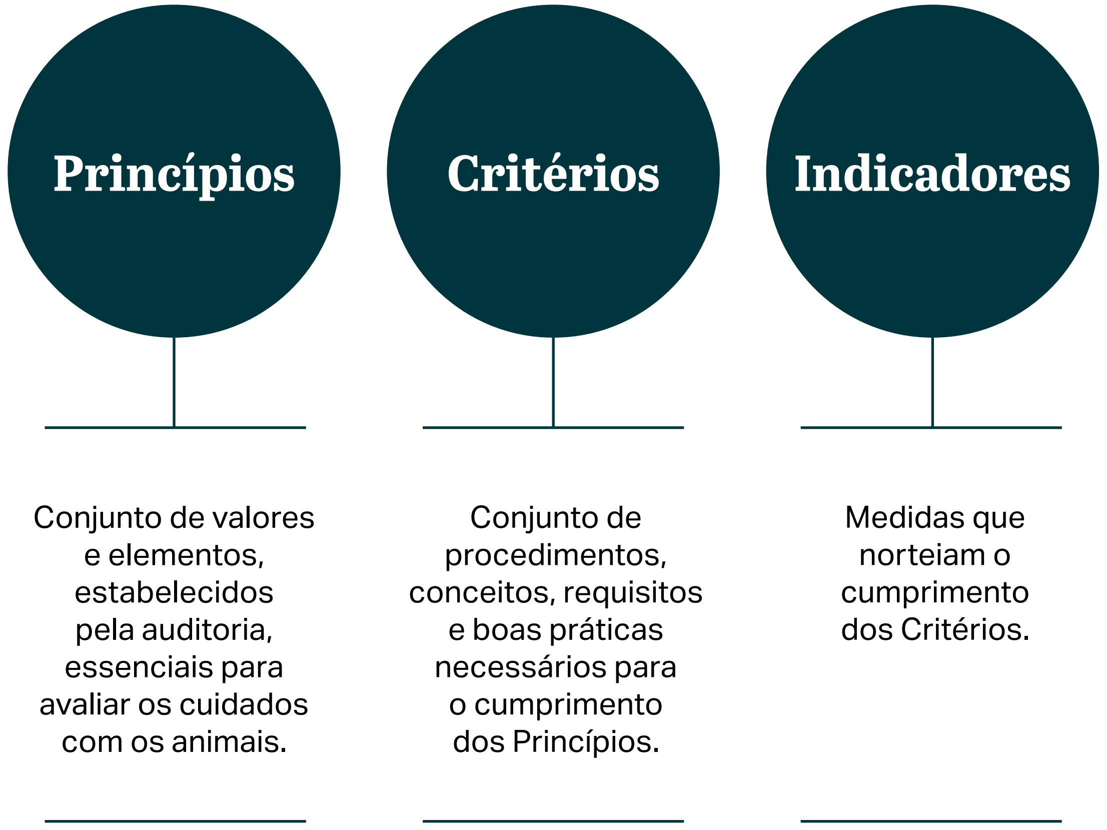

Figura 3 – Princípios, critérios e indicadores.

A partir desses conceitos, foram estabelecidos 5 Princípios, 20 Critérios e 162 Indicadores para a realização da auditoria técnica voltada à proteção da fauna em caso de desastre.

Ressalta-se que a Resolução Conjunta SEMAD/FEAM/IEF/IGAM nº 3.181/2022 (Minas Gerais, 2022) é pioneira no direcionamento de ações voltadas à proteção da fauna no setor de mineração no estado de Minas Gerais. Os indicadores se baseiam nessa legislação, no TAC Fauna e nas lições aprendidas nos rompimentos de barragens. Dessa forma, considerando seu ineditismo, o presente manual representa um potencial de aplicabilidade em diferentes cenários de desastres envolvendo a fauna no Brasil.

4.2.1 Princípios

Os princípios estabelecidos neste documento estão relacionados ao direito à vida animal estabelecidos na Constituição Federal (1988). Esses princípios visam a proteção da fauna e do bem-estar dos animais, considerando a inseparabilidade das relações entre humanos, animais e ambiente (Figura 4).

Princípios

1 GOVERNANÇA

A governança deve seguir padrões e protocolos de modo a definir responsabilidades e processos para tomadas de decisões, além de estabelecer diretrizes para o cumprimento da legislação, normas e acordos.

2 DO PREPARO À RESPOSTA

Estas ações devem seguir os preceitos estabelecidos em instrumentos abarcados em diversas legislações que se relacionam às obrigatoriedades atribuídas às organizações auditadas voltadas à proteção da fauna em situações emergenciais.

3 INTERAÇÃO HUMANO E ANIMAL

A interação humano e animal é o reconhecimento de que o ser humano e os outros animais compartilham um longo e histórico relacionamento que traz benefícios para todas as espécies envolvidas quando em equilíbrio.

4 BEM-ESTAR DO ANIMAL ABRIGADO

Os Cinco Domínios do bem-estar são base técnica para o manejo ético dos animais abrigados, devendo ser aplicados considerando-se as particularidades cada espécie.

5 SAÚDE ÚNICA

Gestão interativa e multidisciplinar para avaliação combinada de riscos à saúde nas quatro áreas ecológicas (humano, animal, plantas e do ambiente físico) para implementação de estratégias de intervenção e prevenção.

Figura 4 – Definição dos princípios elencados para o Manual de Auditoria Técnica voltado à Proteção da Fauna em Caso de Desastres e Evacuações por Barragens.

4.2.2 Critérios

Os Critérios foram elaborados como um conjunto de procedimentos, conceitos, requisitos e boas práticas que resultam da aderência a um Princípio, sendo que sua verificação se dá através dos Indicadores (Figura 5).

Manual para Auditoria de Fauna

1 GOVERNANÇA
- **CRITÉRIO I** Gestão
- **CRITÉRIO II** Gestão de Informações e Banco de Dados
- **CRITÉRIO III** Ações de Treinamentos e Aperfeiçoamento Profissional

2 DO PREPARO À RESPOSTA
- **CRITÉRIO IV** Diagnósticos Ambientais e Fauna Silvestre
- **CRITÉRIO V** Inventário da Fauna Doméstica, Exótica e Silvestre Cativa
- **CRITÉRIO VI** Ações Preparatórias para Desastre
- **CRITÉRIO VII** Ações de Resposta Emergencial
- **CRITÉRIO VIII** Avaliação e Mitigação de Impacto à Fauna Atingida
- **CRITÉRIO IX** Reabilitação e Reintroduçao de Animais Silvestres

3 INTERAÇÃO HUMANO E ANIMAL
- **CRITÉRIO X** Ações Voltadas para Assegurar a Relaçao Humanos e Animais
- **CRITÉRIO XI** Reintegração ao Tutor
- **CRITÉRIO XII** Ações de Adoção e Pós-adoção

4 BEM-ESTAR DO ANIMAL ABRIGADO
- **CRITÉRIO XIII** Domínio Nutricional
- **CRITÉRIO XIV** Domínio Ambiental
- **CRITÉRIO XV** Domínio Sanitário
- **CRITÉRIO XVI** Domínio Comportamental
- **CRITÉRIO XVII** Domínio Mental

5 SAÚDE ÚNICA
- **CRITÉRIO XVIII** Natureza Biológica
- **CRITÉRIO XIX** Natureza Física
- **CRITÉRIO XX** Natureza Química

Figura 5 – Princípios e Critérios estabelecidos para o Manual de Auditoria Técnica voltado à Proteção da Fauna em Caso de Desastres e Evacuações por Barragens.

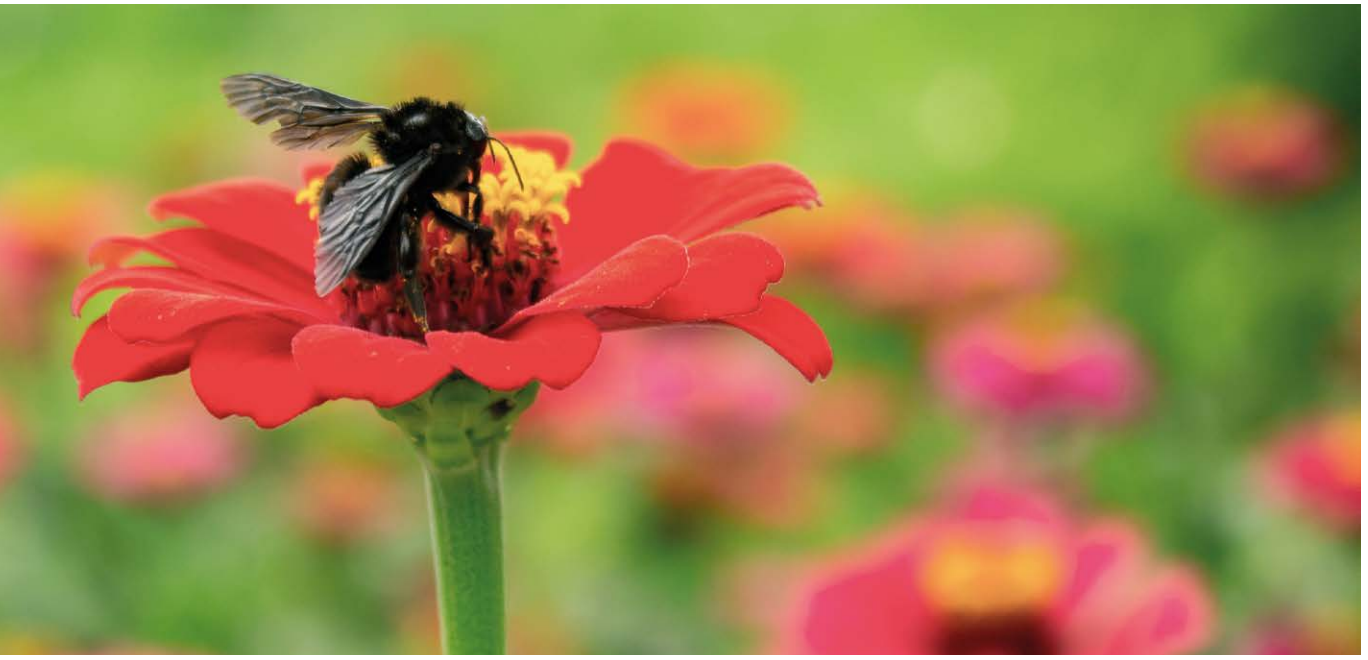

4.2.3 Indicadores

Os Indicadores foram definidos como as medidas que norteiam o cumprimento dos Critérios de auditoria, funcionando como um parâmetro qualitativo. Estes são elaborados para descrever uma característica objetiva e verificável, assim como uma lista de checagem e que, ao serem avaliados periodicamente, denotam a existência ou não de tendências. A listagem completa de indicadores foi desenvolvida para avaliar diferentes conjunturas relacionadas às questões de proteção à fauna. Para avaliação de desempenho do processo de auditoria, foi atribuído para cada indicador um peso de magnitude diferente.

Para calcular o peso de cada Indicador, foram considerados seus distintos níveis de severidade, riscos associados ao seu descumprimento, fundamentos legais, grau de reversibilidade e magnitude das ações corretivas. Para tanto, foi desenvolvida uma matriz de interação que avalia características intrínsecas associadas aos Indicadores, considerando quatro variáveis: referências, riscos associados, tempo sem conformidade e grau de reversibilidade do dano.

A variável **referências** está relacionada à fonte de informação que fundamenta a aplicação do Indicador. Elas podem ser respaldadas em normas internas estabelecidas pela organização auditada, pelas boas práticas de conhecimentos técnicos e científicos ou por leis, resoluções, normativas, dentre outros, que pressupõem a obrigatoriedade de seu cumprimento.

A variável **risco associado** avalia a gravidade do descumprimento do Indicador perante a vida humana ou animal e danos ao meio ambiente. É classificada de acordo com a probabilidade da ocorrência de ferimentos, dor ou sofrimento, incapacidade ou óbito, além da capacidade de gerar danos ambientais.

A variável **tempo sem conformidade** está diretamente associada ao período de resposta necessário para se alcançar a conformidade do Indicador, que pode variar entre as demandas imediatas, de curto ou médio prazo e urgentes, segundo a criticidade do Indicador.

A variável **grau de reversibilidade do dano** se refere ao tempo despendido para reverter os danos causados pelo descumprimento de um Indicador. Os danos podem ser classificados como reversíveis a curto, médio ou longo prazo ou, ainda, como irreversíveis. A matriz de interação com o resumo dos itens e respectivos pesos associados é apresentada abaixo.

	Peso 1	Peso 2	Peso 3	Peso 4
**Referência**	Boas práticas, padrões de excelência e métodos otimizados	Literatura científica com Fator de Impacto (Impact Factor, IF) relevante em áreas de ciências da vida e medicina	Normas e políticas internas da auditadas, associadas aos ODS propostos pela ONU	Resolução de órgãos oficiais e conselhos de Classe, I.N. e acordos judiciais regionais, Leis e decretos
**Risco Associado**	O descumprimento do Indicador não gera ferimentos em humanos ou animais	O descumprimento do Indicador pode gerar ferimentos leves, que necessitam de medidas básicas de primeiros socorros	O descumprimento do Indicador pode gerar ferimentos graves, dor e/ou sofrimento, com necessidade de atendimento médico especializado	O descumprimento do Indicador pode gerar incapacidade ou o óbito / pode gerar dano ambiental (alterações das propriedades físicas, químicas ou biológicas do meio ambiente)
**Tempo Sem Conformidade**	O Indicador não demanda ações imediatas para sua solução	O Indicador demanda ações de médio a longo prazo para sua solução	O Indicador demanda ações de curto prazo para sua solução	O Indicador demanda ações urgentes e imediatas para sua solução
**Grau de Reversibilidade do Dano**	Os danos causados pelo descumprimento do Indicador são reversíveis a curto prazo	Os danos causados pelo descumprimento do Indicador são reversíveis a médio prazo	Os danos causados pelo descumprimento do Indicador são reversíveis a longo prazo	Os danos causados pelo descumprimento do Indicador são irreversíveis

Matriz de interação para avaliação de pesos associados aos Indicadores.

Após a avaliação individual de cada Indicador com a atribuição dos pesos para cada variável analisada, é realizada a soma do resultado das variáveis para gerar um valor final, que estabelece a classificação dos Indicadores como leve, moderado, alto ou crítico, conforme apresentado na Figura 6.

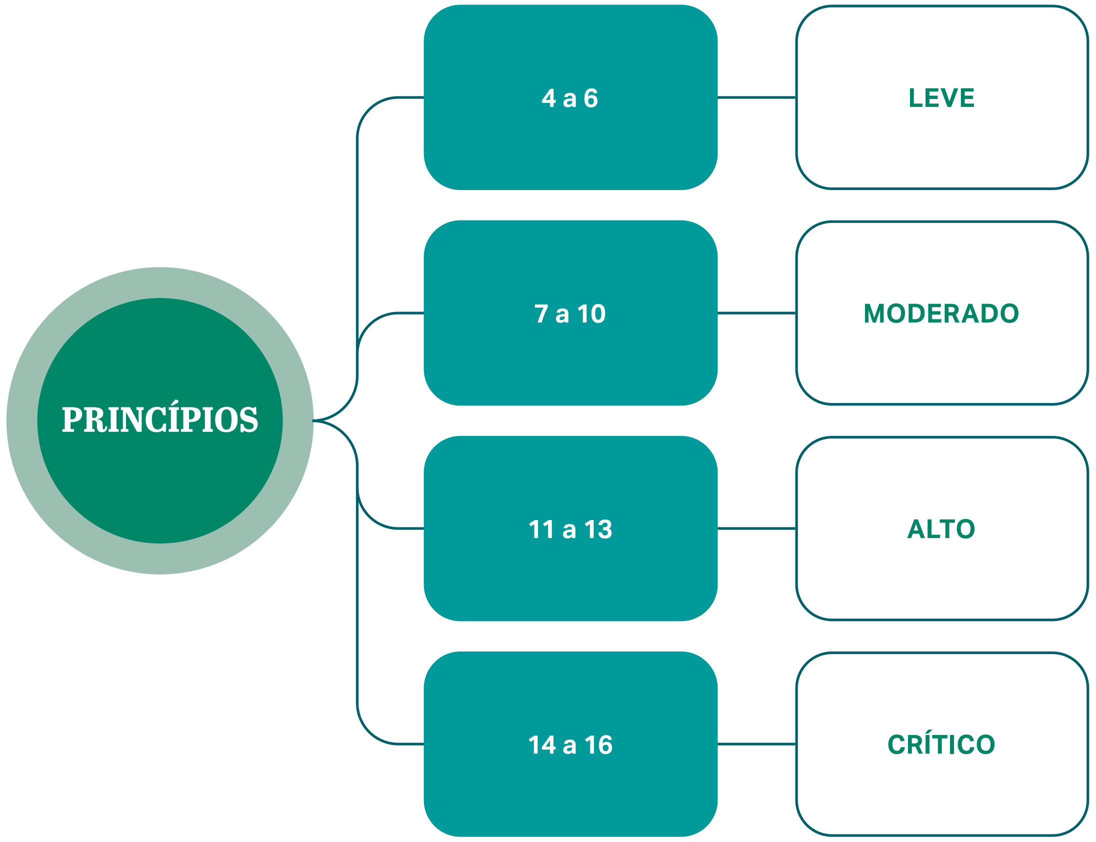

Figura 6 – Nota do indicador considerando o método aplicado à auditoria técnica voltada à proteção da fauna.

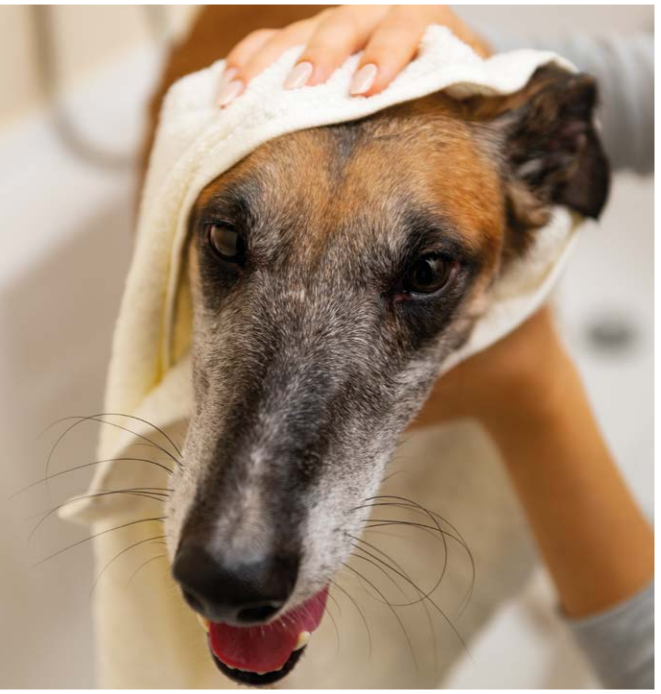

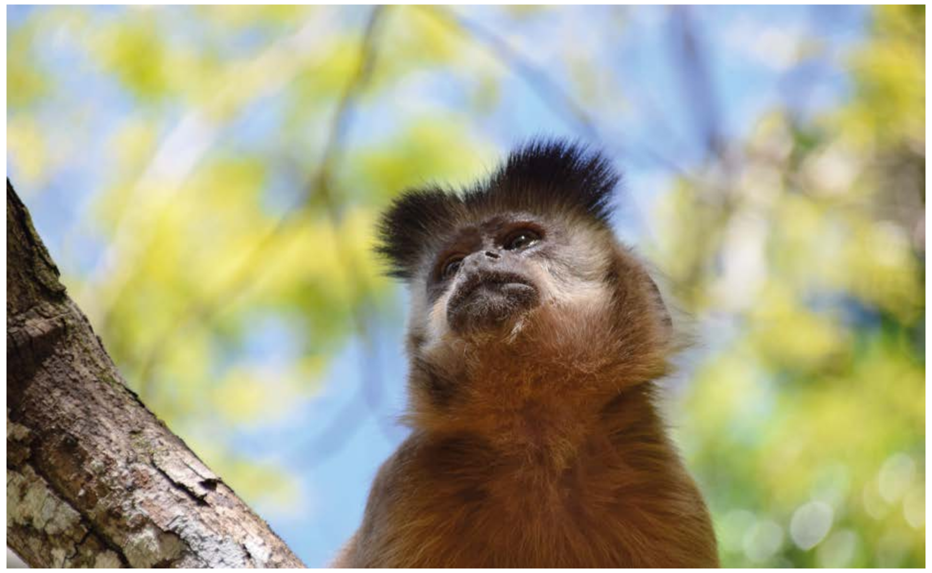

4.2.4 Operacionalização

Este método foi elaborado para ser adaptável a diferentes cenários e deve ser aplicado de acordo com uma ordem previamente estipulada (Figura 7), visando a melhor adequabilidade dos Princípios, Critérios e Indicadores ao cenário a ser analisado.

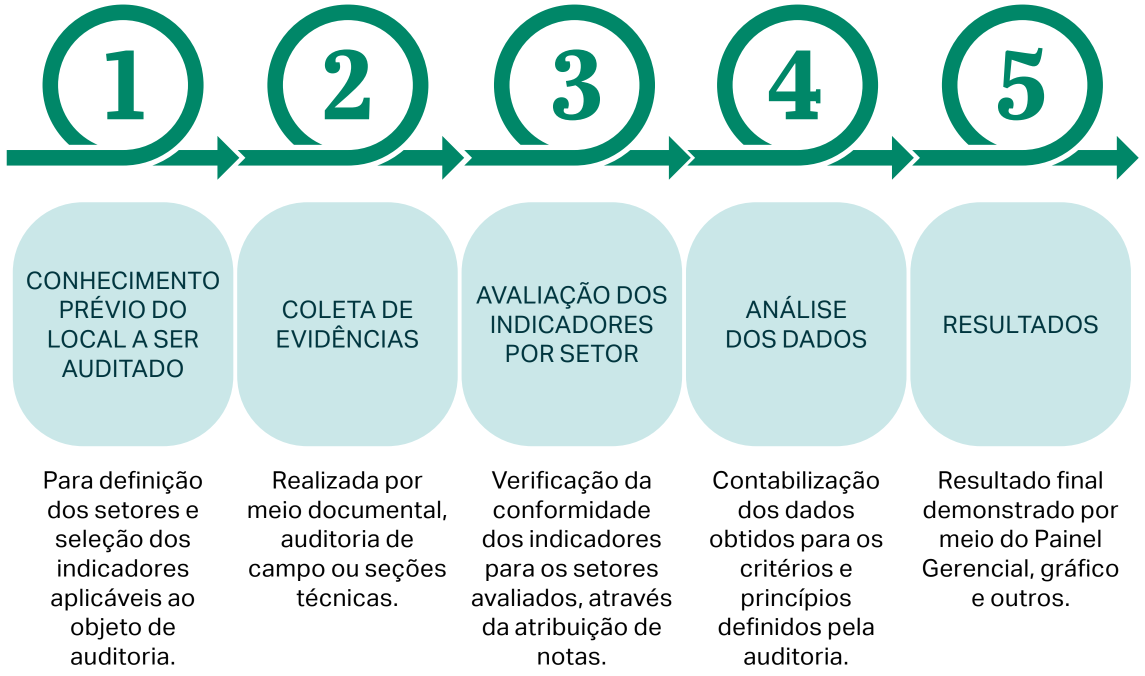

Figura 7 – Fases da operacionalização do método de auditoria técnica voltada à proteção da fauna.

Etapa 1

A adaptabilidade deste método a diferentes cenários se baseia na customização da planilha de métricas, realizada através da seleção dos Indicadores. É essencial o conhecimento prévio das especificidades do cenário auditado. Por exemplo, para a auditoria de uma instalação de fauna com setor para ictiofauna, os indicadores específicos para peixes devem ser selecionados. Portanto, a utilização dos indicadores apropriados é primordial na aplicação deste manual, uma vez que permitirá a obtenção de resultados específicos para diferentes setores, temas ou áreas do cenário auditado.

Etapa 2

Após o planejamento e a seleção dos indicadores, inicia-se a auditoria técnica. A coleta de evidências pode ocorrer de maneira documental, por meio do recebimento de documentação da organização auditada ou *in loco*, através da auditoria técnica de campo e/ou em reuniões de esclarecimento com a organização auditada.

Na auditoria técnica de campo, a equipe de auditores se desloca ao local definido, a fim de evidenciar e acompanhar as ações executadas, conforme os Princípios, Critérios

e Indicadores. É importante que a auditoria técnica de campo seja iniciada por uma reunião de abertura, com o objetivo de confirmar os propósitos das ações a serem auditadas, apresentar a equipe de auditores e assegurar que todas as atividades planejadas possam ser realizadas dentro do tempo e escopo proposto. Cabe ao auditor realizar uma análise crítica das informações verificadas em campo e as documentadas. O resultado destas etapas é a verificação do cumprimento dos Princípios e Critérios pela organização auditada, através da atribuição de notas aos Indicadores.

Etapa 3

Após a auditoria de todas as informações recebidas, uma equipe de auditores determinará de forma conjunta a aferição do grau de conformidade de cada Indicador. Dessa forma, os Indicadores deverão receber uma nota que sintetiza a avaliação do grupo de auditores, sendo estas: 0 para os Indicadores conformes; 0,5 para os parcialmente conformes; 1 para os não conformes. Nota-se que os Indicadores são avaliados baseados na dimensão das inconsistências observadas.

Ressalta-se que todas as avaliações e notas obtidas refletem o momento da auditoria, que considera as evidências de um determinado período de tempo. Portanto, as notas não são definitivas e o esperado é que, através da implementação de ações corretivas pela organização auditada, os resultados de não conformidade ou conformidade parcial possam ser reavaliados em novos ciclos de auditoria.

Etapa 4

A quantificação dos Indicadores classificados como parcialmente conforme ou não conforme, dentro de cada Princípio e Critério avaliado, irá subsidiar a elaboração do resultado da auditoria técnica voltada à proteção da fauna. Nesse ponto, as notas atribuídas na Etapa 3 serão multiplicadas pelo peso de cada Indicador, já que este método considera a relevância dos Indicadores em, por exemplo, assegurar o bem-estar do animal abrigado ou na avaliação de ações emergenciais necessárias em casos de desastres. O resultado do somatório da multiplicação das notas pelo peso de cada Indicador irá gerar uma classificação por Critério e Princípio, sendo estes categorizados em: adequado, requer melhorias e inadequado.

Dessa forma, esse método de auditoria permite a visualização de pontos de atenção e de pontos críticos para determinado setor, dentro de cada Princípio, que podem gerar recomendações emitidas pela auditoria para que a organização auditada realize ações corretivas e ou apresente justificativas, direcionando, assim, as intervenções para maior celeridade de atendimento.

Etapa 5

Os resultados de cada auditoria – ou ciclo de auditoria – são representados de forma gráfica, através de painéis gerenciais (Figura 8) e gráficos, permitindo uma rápida visualização dos princípios e dos setores em relação ao seu grau de adequabilidade. A aplicação desse método permite a obtenção de resultados comparáveis entre diferentes áreas avaliadas e entre os diferentes critérios que compõem o resultado. Ao mesmo tempo, devido à sua padronização, a utilização desse método permite a comparação entre diferentes cenários de aplicação de auditoria, possibilitando a realização de uma análise longitudinal dos resultados, relacionando os diferentes ciclos de auditoria.

Abrigo de Fauna

Princípio / Setor	Gestão e Áreas de Apoio	Setor Quarentena	Setor Gatos	Setor Necropsia	Nota Princípios
**Governança**	✓	±	✓	✗	✗
**Do Preparo à Resposta**	−	−	−	−	−
**Interação Humano e Animal**	✓	✓	✓	−	✓
**Bem-estar do Animal Abrigado**	✓	✓	✓	−	✓
**Saúde Única**	✓	✓	✓	✗	±
**Nota Setores**	✓	✓	✓	✗	±

✓ Em conformidade   ± Requer melhorias   ✗ Não conforme   − Não se aplica

Figura 8 - Exemplo de Painel Gerencial aplicado a um abrigo de fauna.

A seguir, são apresentados de forma detalhada os Princípios, Critérios e Indicadores que formam a base técnica desse método de auditoria voltado à proteção da fauna.

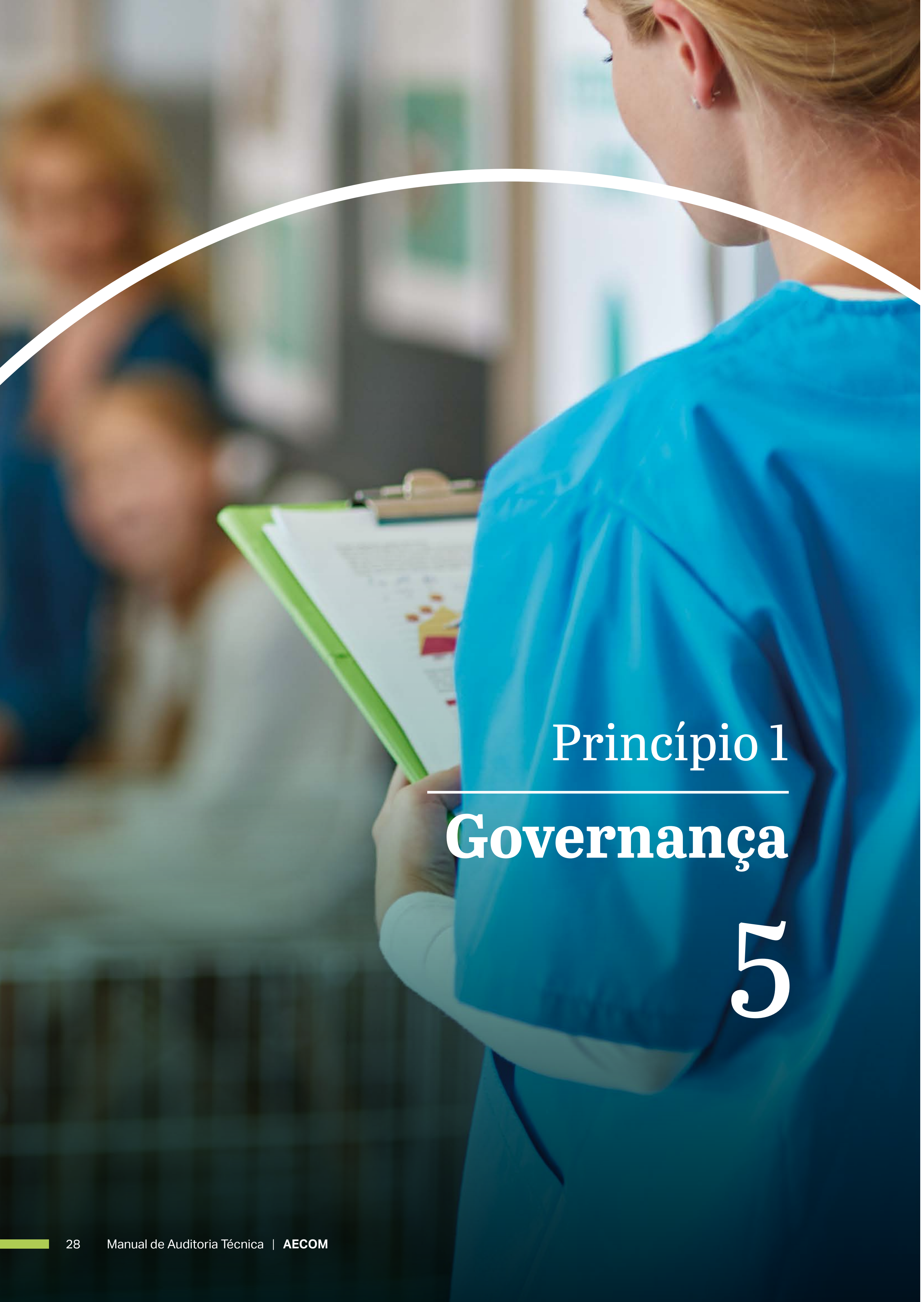

5. Princípio 1 Governança

A governança consiste em um conjunto de ações que definem as responsabilidades e estabelecem os processos para tomada de decisão, além de determinar as diretrizes para o cumprimento da legislação, normas e acordos. É relevante que a governança seja estruturada para que qualquer mudança no corpo técnico ou na estrutura organizacional do empreendedor não afete os outros princípios de sua gestão. Ela deve ser conduzida de forma ética, por meio de políticas e procedimentos padronizados que visem a assegurar a aplicabilidade das leis, acordos, normativas e boas práticas. Para isso, a governança precisa ser organizada e bem estruturada, prezando pela transparência entre os gestores (incluindo das empresas terceirizadas), colaboradores (internos e externos) e outros atores envolvidos, como órgãos públicos e a população atingida.

É necessário versar os três conceitos-chave para esse princípio: liderança, estratégia e controle. Para o conceito de liderança, há a necessidade de definição das responsabilidades de cada um dos envolvidos, criando um cenário onde cada profissional possa executar suas atividades com ética e transparência. Para o conceito de estratégia, é necessário que as ações realizadas pela organização auditada estejam alinhadas aos objetivos previamente definidos e pautadas em princípios éticos e sustentáveis, evitando, assim, os problemas de entendimento entre a equipe que vai executá-la, a população atingida e as autoridades legislativas e governamentais. Já quanto ao conceito de controle, procedimentos deverão ser estabelecidos em conformidade com as estratégias e ações, assegurando a regularidade do método e a frequência das atividades realizadas, gerando confiabilidade dos dados apresentados à população atingida e às autoridades legislativas e governamentais.

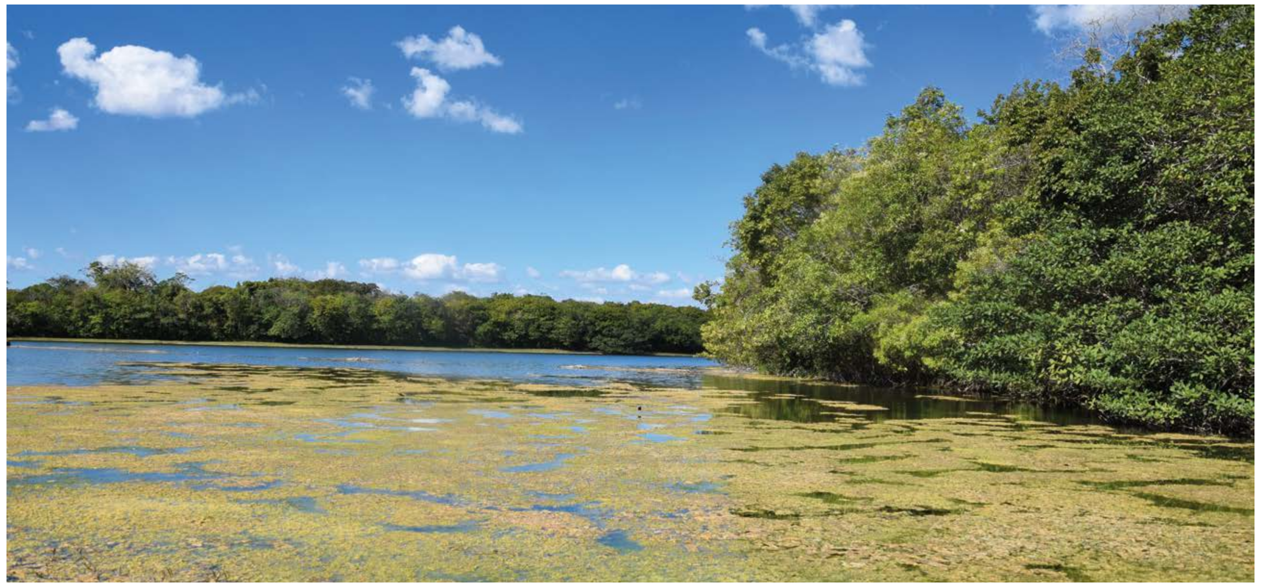

Para o Princípio Governança, no contexto do processo de auditoria técnica voltado à proteção da fauna, são elencados três Critérios avaliativos, conforme a Figura 9.

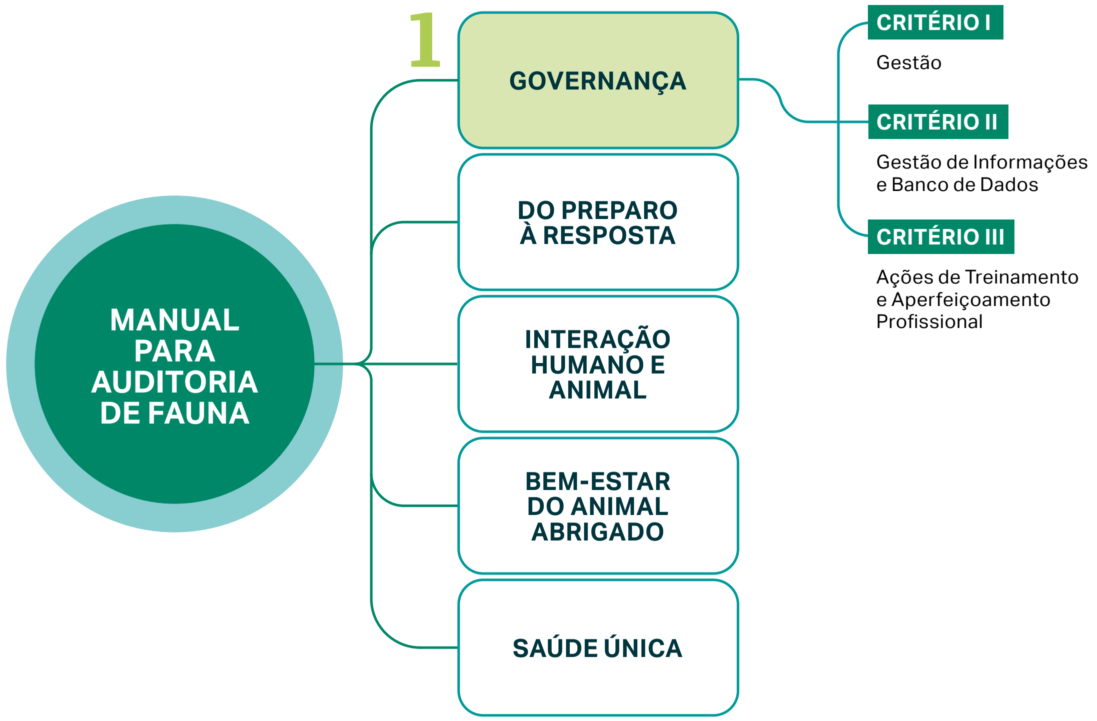

Figura 9 – Critérios avaliativos do Princípio Governança.

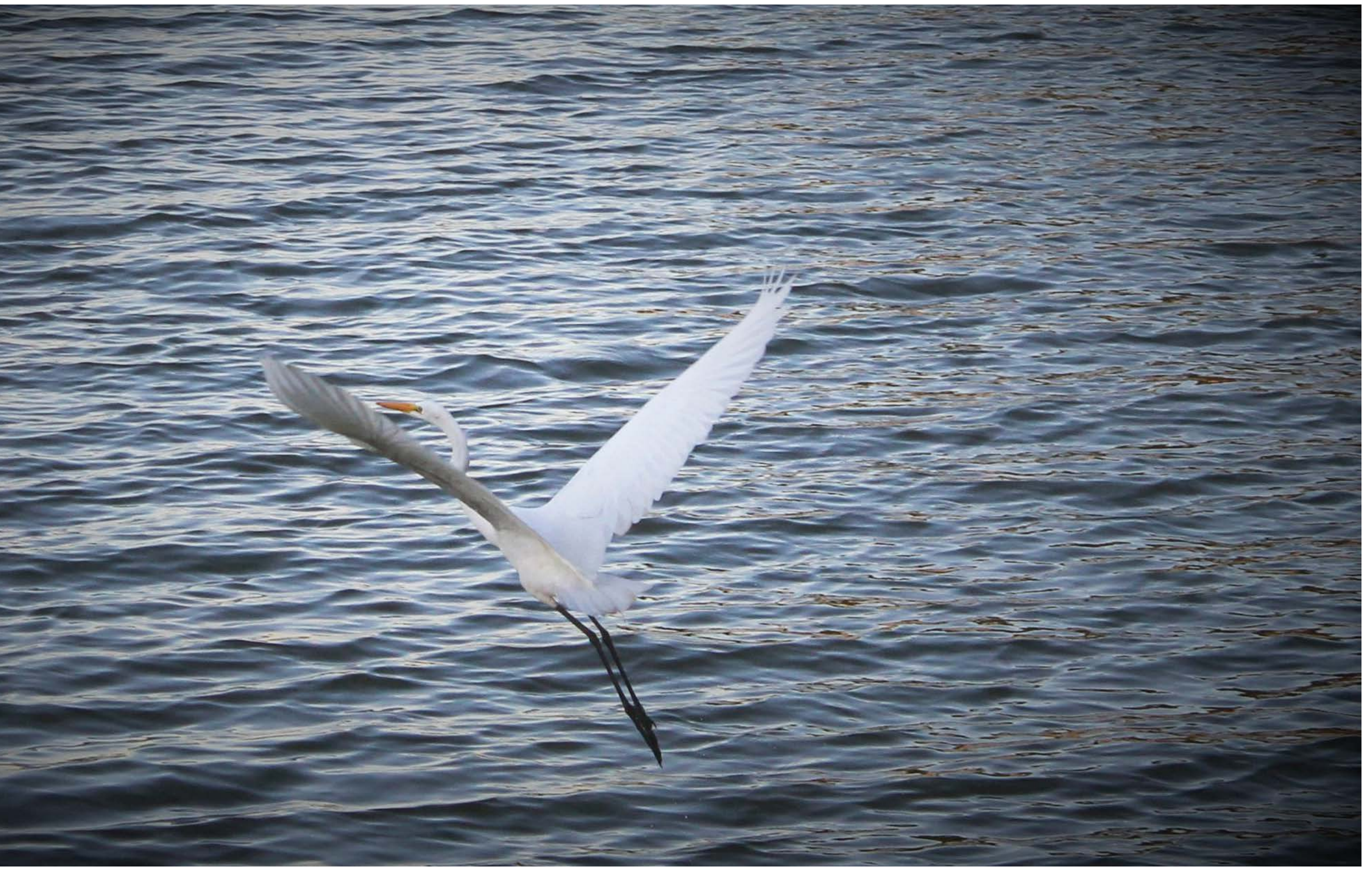

5.1 Critério I – Gestão

DEFINIÇÃO	A Gestão está relacionada ao gerenciamento dos recursos disponíveis para manter a segurança, a rastreabilidade e ao bem-estar do animal abrigado, sejam eles equipes, estoques, orçamento, equipamentos, dentre outros, que devem compor o planejamento das atividades para atendimento aos objetivos do abrigo.<br>Como ferramentas de gestão, destacam-se os procedimentos operacionais, que consistem em descrições detalhadas de todas as medidas necessárias para a realização de uma atividade e a Capacidade de Prover Cuidados (CPC), que se refere à capacidade máxima de atendimento de cada abrigo.
IMPORTÂNCIA	Uma gestão adequada permite que as ações sejam tomadas de maneira assertiva e qualificada, reduzindo as falhas, otimizando as atividades e ajustando as estratégias. Os procedimentos operacionais e as ferramentas de monitoramento são importantes para promover a padronização das atividades realizadas pela organização auditada, além de organizar e facilitar a tomada de decisões.
ATRIBUIÇÕES DA AUDITORIA TÉCNICA	Auditar a gestão do abrigo de fauna por meio de sua estrutura hierárquica nas ações a serem desenvolvidas, dos controles de entrada e saída de indivíduos, da rastreabilidade dos animais, considerando seu local de abrigo e segurança e do quadro de profissionais, próprios e terceiros.<br>Auditar a qualidade e o grau de conformidade dos procedimentos operacionais e das ferramentas de monitoramento com as legislações existentes e boas práticas, quando aplicável, além de verificar a conformidades dos documentos com a finalidade a que se destinam, sua aplicabilidade e execução.

INDICADORES

1. A organização auditada demonstra desenvolver suas atividades de acordo com as orientações e diretrizes propostas pelos órgãos ambientais federal, estadual e municipal, conforme os requisitos para a sua exigibilidade, riscos ambientais, o porte do empreendimento, os estudos ambientais pertinentes ao respectivo processo de licenciamento.

2. A organização auditada demonstra possuir equipe técnica capacitada e especializada, com registro nos Conselhos de Classe e Cadastro Técnico Federal para o desenvolvimento de todas as demandas de manejo dos animais.

> Nota: A equipe de resgate e evacuação de animais deve ser composta por profissionais treinados para avaliar clinicamente e transportar, de maneira segura, diversas espécies animais.

3. A organização auditada dispõe de equipe técnica proporcionalmente dimensionada para a realização de resgate, evacuação e manejo dos animais durante as ações emergenciais.
4. A organização auditada possui organogramas de funções e responsabilidades claramente definidos.
5. A organização auditada fornece identificação (temporária ou permanente) a todos os animais sob sua tutela de maneira adequada a cada espécie.

> Nota: *Microchips* devem ser aplicados por via subcutânea, seguindo as normas internacionais (ISO) e recomendações emitidas pelo Subcomitê para *microchips* da Associação Mundial de Médicos Veterinários de Pequenos Animais (WSAVA, 2020).
>
> Nota: É aceitável que para algumas espécies (por exemplo, coelhos) não seja utilizada identificação visual externa sob o risco de prejudicar o bem-estar do animal. Nesse caso, o *microchip* é indispensável (Gebara, 2019).

6. A organização auditada fornece diagnóstico clínico, tratamento médico-veterinário, hospital de campanha em caso de emergência e internação de animais de acordo com as legislações e normativas dos órgãos competentes em relação aos procedimentos e estruturas necessárias.
7. A organização auditada possui planejamento de continuidade operacional da capacidade de prover cuidados durante emergências, incluindo mapeamento dos recursos que podem ser utilizados durante a resposta, sem prejuízo para a população já abrigada.
8. A organização auditada possui um plano de evacuação emergencial para o abrigo de fauna e realiza treinamentos periódicos com a equipe.
9. A organização auditada evidencia o tombamento de exemplares em coleção biológica de referência, quando for prevista a coleta de espécimes da fauna silvestre ou exótica cativa.
10. A organização auditada possui documentos e licenças para coleta e transporte de material biológico.
11. A organização auditada mantém os estoques em locais distintos e adequados, conforme os tipos de material armazenado e as especificações da legislação vigente.
12. A organização auditada fornece áreas para descanso e alimentação para os colaboradores, voluntários e qualquer outro pessoal envolvido no manejo de animais.

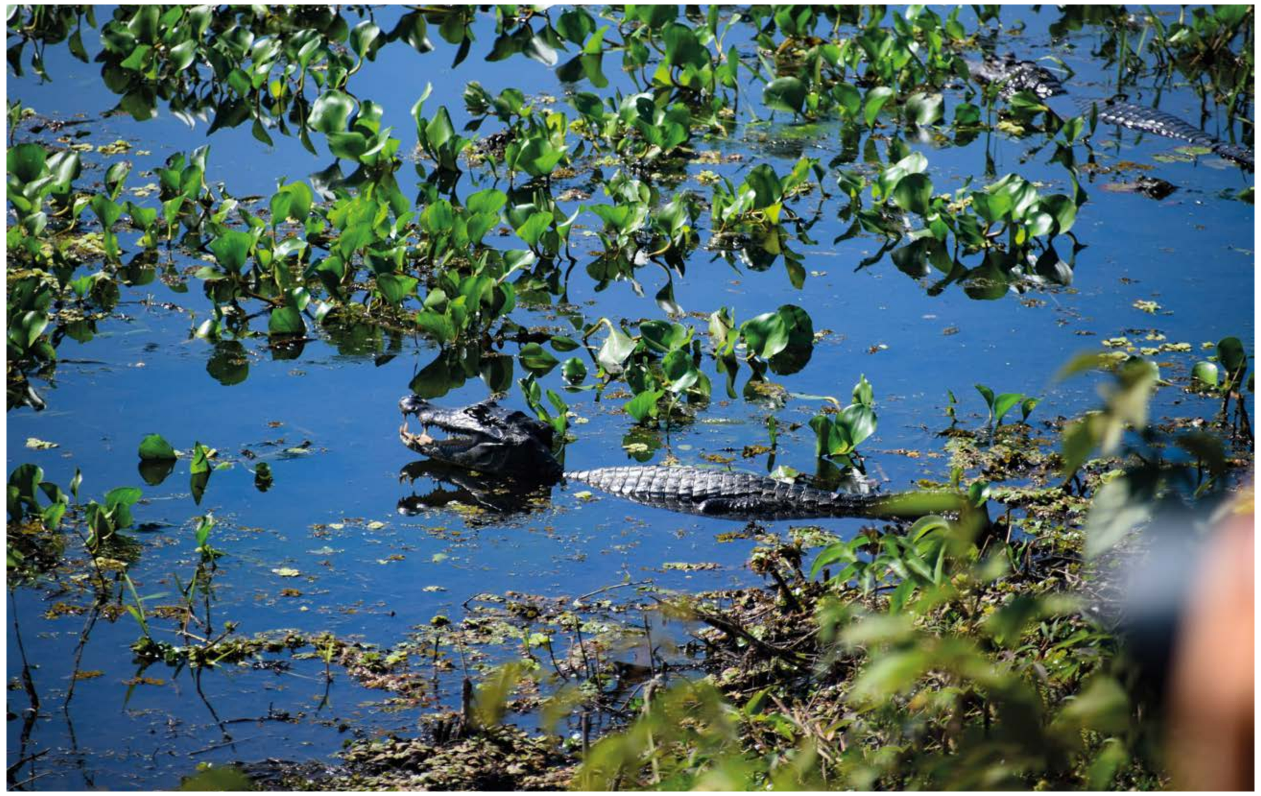

13. A organização auditada possui POPs atualizados e de fácil acesso aos colaboradores.

> Nota: Alguns exemplos de procedimentos operacionais que podem ser implantados relacionados à fauna são: limpeza e organização, controle e organização de estoque, manejo alimentar e bem-estar, resgate e transporte de animais domésticos e silvestres.

14. A organização auditada realiza treinamentos periódicos de seu corpo técnico para a implementação eficaz dos POPs, visando o manejo ético, o bem-estar da fauna atingida, a segurança do meio ambiente, das pessoas e dos animais.

> Nota: A organização auditada deve manter o registro ou evidências dos treinamentos e atualizações realizados com a equipe de colaboradores.
>
> Nota: Podem ser realizados eventos periódicos de treinamento e educação continuada (educação ambiental, por exemplo) sobre saúde coletiva, doenças ocupacionais e zoonoses com a equipe de colaboradores, incluindo informações sobre o reconhecimento de condições potencialmente zoonóticas e os meios de proteger outras pessoas da exposição.

15. A organização auditada monitora a execução de seus POP e realiza revisões periódicas.
16. A organização auditada implementa um plano de comunicação e educação ambiental com a comunidade residente na área atingida.

> Nota: A auditada deve informar a comunidade sobre o resultado das análises toxicológicas da água e/rejeito, se aplicável.

5.2 Critério II – Gestão de Informações e Banco de Dados

DEFINIÇÃO	Banco de dados para fauna é um conjunto de informações organizadas sobre um grupo de animais, estruturadas e armazenadas, podendo ou não serem disponibilizadas ao público externo. Este banco de dados é geralmente controlado por um Sistema de Gerenciamento de Banco de Dados (SGBD) que deve operar com atualização periódica das informações sobre a fauna.
IMPORTÂNCIA	O banco de dados é a forma ideal para se armazenar, editar, rastrear e manter arquivos e registros. É através da rastreabilidade que os dados de recebimento, manutenção e triagem de cada indivíduo registrado podem ser acessados e analisados, permitindo a compreensão do histórico do animal, suas especificidades e condições de saúde, assim como contribuir para o controle de entrada e saída do abrigo e o cálculo da Capacidade de Prover Cuidados. O banco de dados também possibilita a rastreabilidade do histórico do animal sob tutela ou atendido pela organização auditada e retorno aos tutores originais.
ATRIBUIÇÕES DA AUDITORIA TÉCNICA	Auditar o objetivo geral do armazenamento e a consistência dos dados coletados, sua atualização e sua adequação ao objetivo definido.<br>O auditor deve conhecer o SGBD em uso para que possa identificar como podem ser mantidos a integridade, a segurança e o controle dos dados inseridos nas bases. Considerando a possibilidade de rastreamento individual, o auditor pode avaliar de forma amostral os documentos de registro individual dos animais para compreender como ocorreu seu resgate, o estado de saúde, sua identificação física (brinco, *microchip*, anilha, coleira) e a identificação do recinto caso ele se encontre abrigado em instalações coletivas.

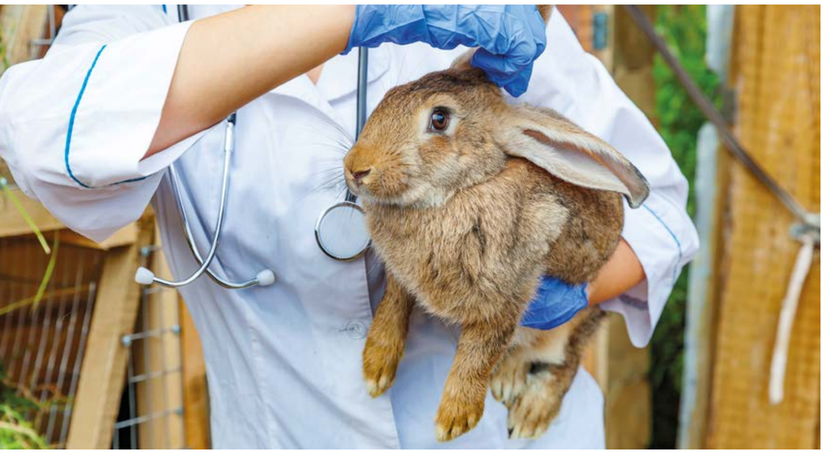

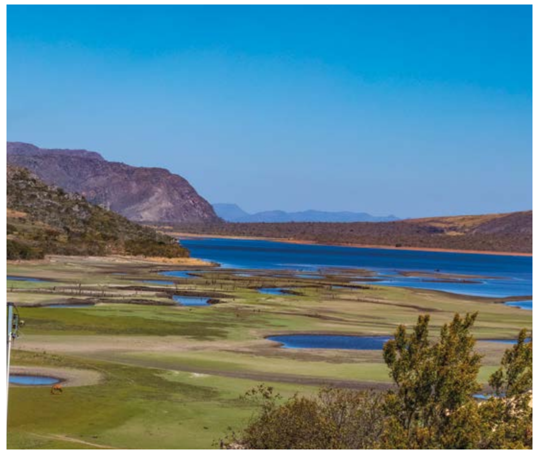

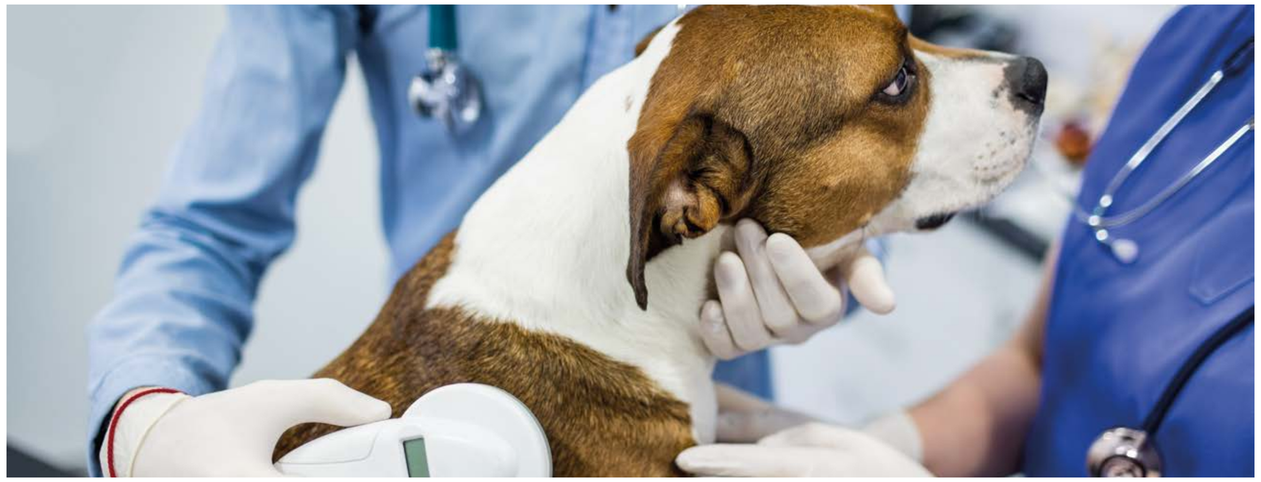

INDICADORES

17. A organização auditada realiza o registro adequado das informações de identificação dos animais resgatados ou evacuados.

    Nota: Em situações de resgate, a ficha de identificação do animal deve incluir local (coordenadas geográficas), data e hora do resgate, espécie, cor, marcas, estado clínico, nome do tutor (se possível), entre outras características únicas daquele indivíduo.

    Nota: No caso do animal permanecer junto ao tutor, é importante que se assegure a identificação permanente, com a permissão do tutor.

18. A organização auditada possui uma base de dados digital sobre a fauna assistida, acessível ao público de interesse.

    Nota: Quando aplicável, os dados de cães e gatos microchipados devem ser inseridos nos bancos de dados oficiais do município ou estado onde ocorrem as ações, respeitando-se o cumprimento da LGPD (Brasil, 2018).

19. A organização auditada possui controle periódico do quantitativo de entradas e saídas de todos os animais sob sua tutela.

    Nota: São consideradas "entradas" informações sobre resgates, nascimentos e transferência entre abrigos. "Saídas" são óbitos, reintegração ao tutor, adoção e soltura/destinação de animais silvestres.

20. A organização auditada possui procedimentos de *backup* e *restore* em seu banco de dados, a fim de promover a segurança dos dados armazenados.

21. A organização auditada apresenta em seu banco de dados o histórico de cada animal sob a sua tutela, desde o resgate até a sua destinação final, incluindo todos os atendimentos clínicos e movimentações realizadas.

22. A organização auditada apresenta em seu banco de dados ferramentas para a seleção de indivíduos ou grupos de animais, organizados de acordo com características do cadastro realizado durante o resgate e de informações detalhadas dos indivíduos.

5.3 Critério III – Ações de Treinamento e Aperfeiçoamento Profissional

DEFINIÇÃO	São ações propostas e/ou executadas para a comunicação de informações e treinamentos dos colaboradores que atuarão com os animais da área afetada. São planejadas e organizadas para desenvolver as competências, habilidades e conhecimentos dos profissionais melhorando seu desempenho na execução do trabalho com segurança.
IMPORTÂNCIA	As ações educativas visam promover a conscientização, desenvolver o conhecimento e a compreensão, motivar ações de melhoria, manutenção e engajamento de todos os colaboradores envolvidos nas etapas e atividades executadas, na responsabilidade sobre a fauna e seu bem-estar e nos riscos à saúde humana e animal. Para os colaboradores, a preparação para ações de proteção à fauna em emergência minimiza os danos e prejuízos consequentes, tendo em vista objetivos claros e/ou cenários específicos.
ATRIBUIÇÕES DA AUDITORIA TÉCNICA	A equipe de auditoria técnica tem o objetivo de analisar a eficiência das ações educativas e preparatórias implementadas pela organização auditada para seus colaboradores, como as campanhas presenciais, material de comunicação impresso e digital, realização de treinamentos, orientações e resposta dos envolvidos nas atividades propostas.

INDICADORES

23. A organização auditada fornece treinamentos para seus colaboradores, focados nas necessidades das espécies abrigadas.

    Nota: São exemplos das necessidades das espécies dieta, comportamento, ambiente, entre outros.

24. A organização auditada fornece, aos colaboradores especialistas, treinamentos sobre primeiros socorros para animais e humanos e medidas de segurança para manuseio de animais e uso de equipamentos.

25. A organização auditada realiza capacitação contínua dos colaboradores, provendo cursos e treinamentos adaptados às necessidades de manejo animal e características do abrigo.

    Nota: Os abrigos devem ser adaptados a cada fase do processo de resposta: emergência e pós-emergência. A partir da finalização da fase de pós-emergência, as instalações devem ser preparadas para oferecer todas as condições para abrigo de longa duração e/ou permanente, considerando que alguns animais poderão ficar abrigados por todo seu período de vida (aqueles animais sob tutela da auditada que não estão aptos para adoção).

26. A organização auditada fornece capacitação e treinamento para as equipes que atuarão em resgate e/ou prospecção embarcado.

    Nota: Há a necessidade de colaboradores com habilitação específica para condução de embarcações em todas as equipes embarcadas.

27. A organização auditada capacita seus colaboradores que atuam em áreas de resgate, para que saibam lidar de forma eficaz e informativa com os diferentes públicos.

    Nota: Considerando que, durante uma evacuação ou após um desastre, as comunidades atingidas estarão abaladas, as equipes de campo devem estar preparadas para comunicação não violenta e assertiva, objetivando sempre a colaboração da população para resgate ou evacuação de seus animais.

    Nota: Para o atendimento de comunidades tradicionais ou em estado de vulnerabilidade, deve-se considerar a atuação conjunta com um Responsável Comunitário (RC).

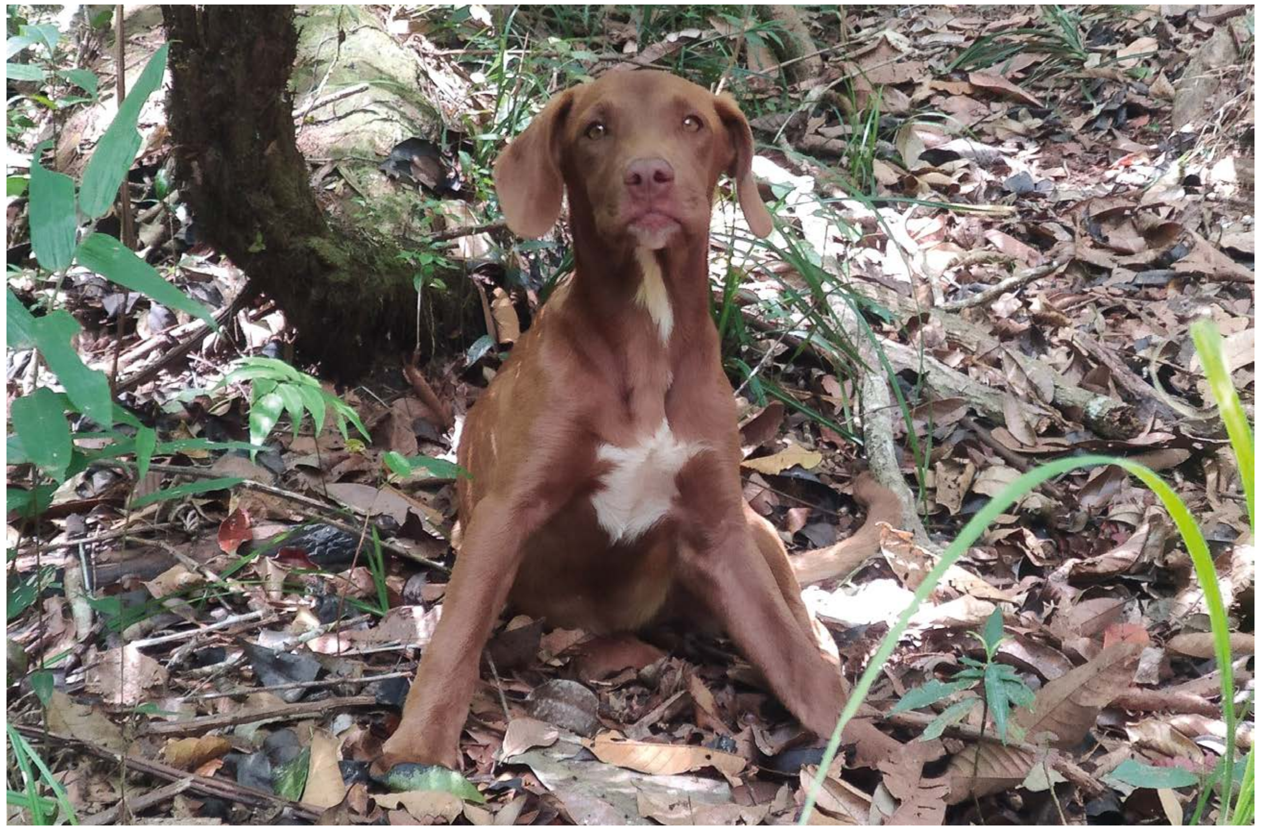

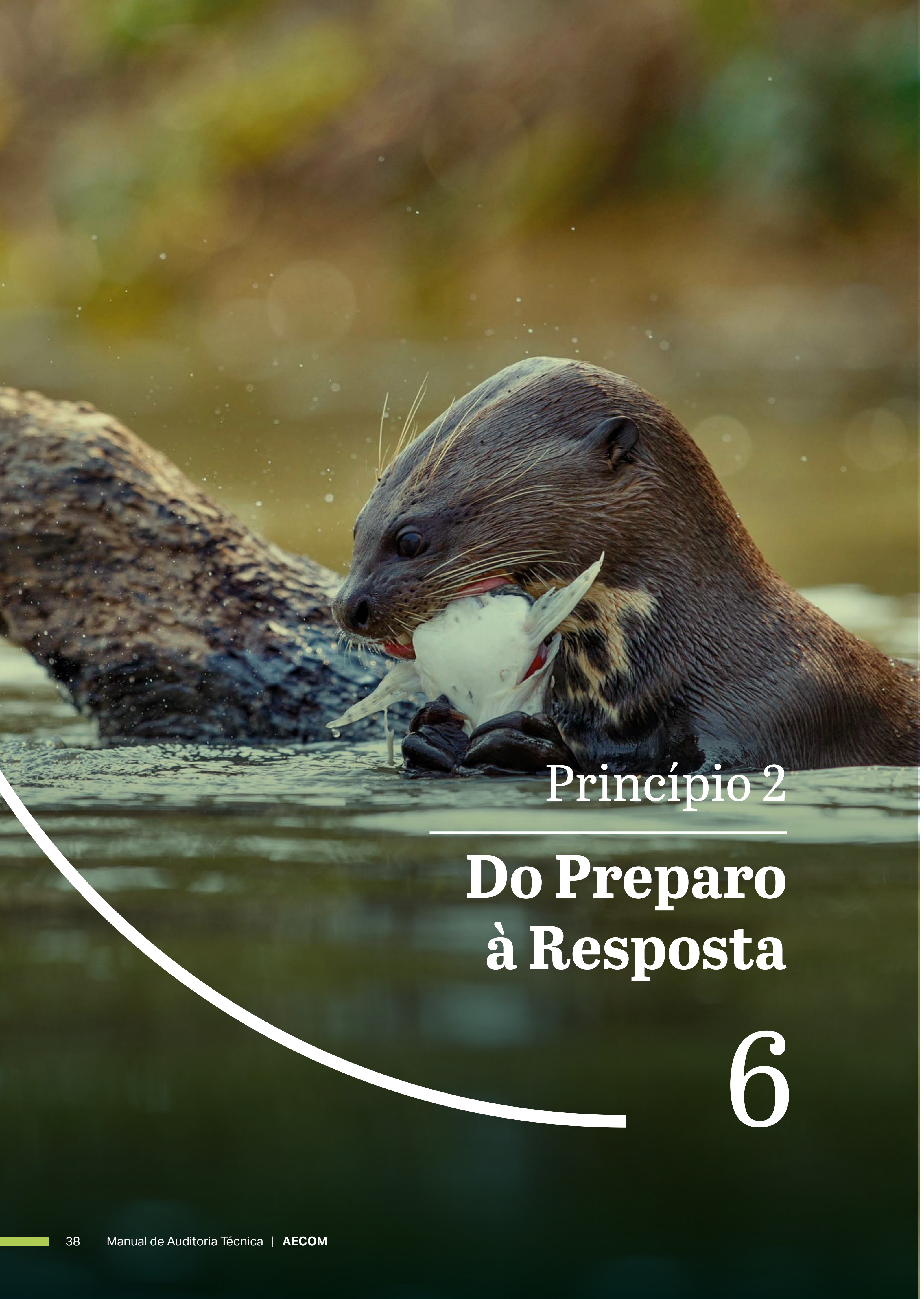

6. Princípio 2
Do Preparo à Resposta

A Constituição Federal (1988), art. 225, dispõe que todos têm direito ao meio ambiente ecologicamente equilibrado, e o poder público e a coletividade têm o dever de defendê-lo e preservá-lo para as presentes e futuras gerações. Para assegurar a efetividade desse direito, incumbe ao poder público, por exemplo: (1) preservar e restaurar os processos ecológicos essenciais e prover o manejo ecológico das espécies e ecossistemas; (2) preservar a diversidade e a integridade do patrimônio genético do país; (3) exigir, na forma da lei, para instalação de obra ou atividade potencialmente causadora de significativa degradação do meio ambiente, estudo prévio de impacto ambiental, a que se dará publicidade; (4) controlar a produção, a comercialização e o emprego de técnicas, métodos e substâncias que comportem risco para a vida, a qualidade de vida e o meio ambiente; (5) promover a educação ambiental em todos os níveis de ensino e a conscientização pública para a preservação do meio ambiente (Deliberação Normativa COPAM nº 214/2017 e alterada pela COPAM nº 238/2020); (6) proteger a fauna e a flora, vedadas, na forma da lei, as práticas que coloquem em risco sua função ecológica, provoquem a extinção de espécies e/ou submetam os animais à crueldade.

No âmbito legal, a preparação para emergências e a temática da segurança de barragens de mineração por parte dos empreendedores e do Estado vêm ganhando cada vez mais relevância no Brasil. Entre as primeiras legislações relacionadas às barragens, a Lei nº 12.334/2010 (Brasil, 2010) estabeleceu a Política Nacional de Segurança de Barragens (PNSB), destinada à acumulação de água para quaisquer usos, à disposição final ou temporária de rejeitos, à acumulação de resíduos industriais, entre outros. Assim, abarca todos os tipos de barragens existentes no território nacional, incluindo aquelas relacionadas à produção energética, acumulação de água e mineração. Outras leis e resoluções estabeleceram critérios e diretrizes para a classificação de risco de barragens e a implementação de políticas de segurança de barragens.

A partir dos eventos de rompimento de barragens em Minas Gerais nos anos de 2015 e 2019, ocorreram a revisão e a publicação de diversas normativas, em âmbito nacional e estadual, com importantes alterações para a proteção da vida e dos bens, baseadas nas experiências de resposta aos desastres. Ainda em Minas Gerais, destaca-se a Lei nº 23.291/2019, que instituiu a Política Estadual de Segurança de Barragens, implementada de forma articulada com a Política Nacional de Segurança de Barragens. Nesse contexto, os Planos de Ação de Emergência de Barragens de Mineração (PAEBM) tornam-se instrumentos centrais dentro do Plano de Segurança de Barragem (PSB). Trata-se do documento que estabelece as orientações para atuação em situações de emergência, possibilitando ações de resposta de forma ordenada. Adicionalmente, também delimita as atribuições do empreendedor e entidades envolvidas no cenário de desastre. A Portaria DNPM nº 70.389/2017 estabelece as diretrizes para a elaboração do Plano

de Ação de Emergência de Barragens de Mineração (PAEBM) de maneira a evitar ou minimizar os riscos à vida nas comunidades localizadas a jusante dessas barragens.

Para tanto, faz-se necessária a operacionalização de forma eficiente dos planos de ação, de maneira que este conceda o suporte necessário, de forma simplificada e elucidativa, para a rápida tomada de decisão em caso de emergência. Nesse sentido, no denominado Princípio do Preparo à Resposta, o processo de auditoria técnica independente é voltado à proteção da fauna em situação de desastres e nele são elencados seis Critérios avaliativos, conforme a Figura 10.

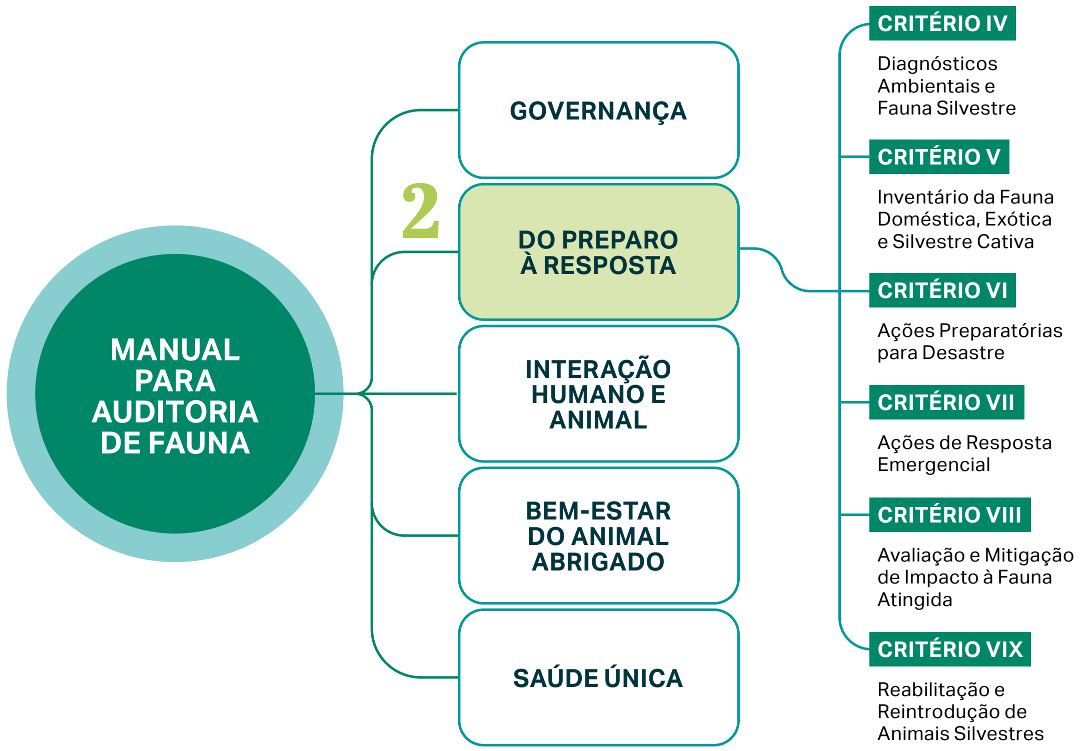

Figura 10 – Critérios avaliativos do Princípio do Preparo à Resposta.

Os indicadores desse Princípio seguiram os preceitos estabelecidos em instrumentos abarcados por diversas legislações. Dentre elas, destaca-se a Resolução Conjunta SEMAD/FEAM/IEF/IGAM nº 3.181/2022 (Minas Gerais, 2022), bem como os Termos de Referência relacionados a esta para o Estado de Minas Gerais. Ainda dentro desse Princípio, alguns indicadores estão relacionados aos estudos ambientais e à avaliação de impactos, bem como aos conceitos determinados pela Resolução CONAMA nº 01/1986 (Brasil, 1986) – que definem as bases dos estudos para avaliação das alterações ambientais decorrentes dos impactos em situações de emergência para barragem de mineração.

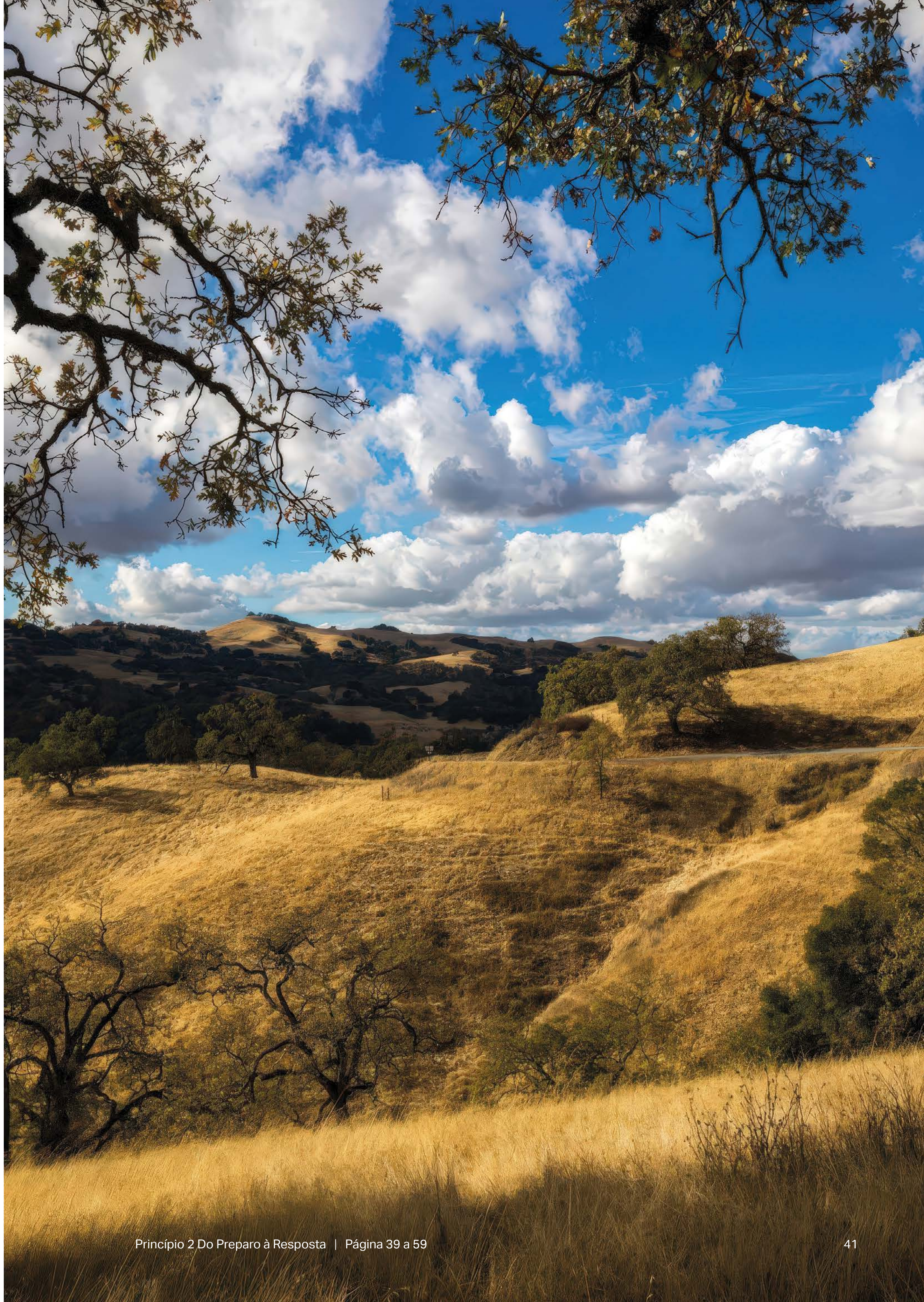

6.1 Critério IV – Diagnósticos Ambientais e Fauna Silvestre

DEFINIÇÃO	O diagnóstico deve caracterizar a fauna silvestre (terrestre e aquática) nas áreas de influência do empreendimento ou áreas afetadas direta e indiretamente em caso de rompimento de barragens. Integra os meios físico e biótico para o conhecimento das espécies da fauna silvestre e a sua distribuição no território. Deve evidenciar os *habitats* e as espécies da fauna silvestre de especial interesse para a conservação, como as ameaçadas de extinção, raras, endêmicas, de distribuição restrita, migratórias, invasoras ou de relevância ecológica e econômica.
IMPORTÂNCIA	Utilizada no planejamento, dimensionamento e direcionamento de ações emergenciais, e na avaliação de impactos ambientais com fins de mitigação, restauração e/ou reparação ambiental pós-desastre.
ATRIBUIÇÕES DA AUDITORIA TÉCNICA	Auditar os documentos que contêm os itens previstos no diagnóstico ambiental que irão subsidiar o planejamento de ações emergenciais e a avaliação de impactos em cenários de desastre. A análise crítica do conteúdo deve avaliar a abrangência, coerência e aplicabilidade da caracterização ambiental, de forma a ser um descritivo dos aspectos físicos e bióticos da área alvo, com foco principal na interação da fauna e dos ecossistemas na área de estudos. A equipe de auditoria técnica independente deve ser composta de especialistas de diferentes áreas considerando a complexidade dos estudos previstos.

INDICADORES

28. A organização auditada apresenta diagnóstico ambiental com a definição da Área Diretamente Afetada (ADA), Área de Influência Direta e Indireta (AI) e Área de Referência (AR) contendo a descrição detalhada dos atributos físicos e bióticos.

29. A organização auditada apresenta análise de ecologia da paisagem, incluindo os aspectos de uso e ocupação do solo e malha hídrica.

30. A organização auditada apresenta, no diagnóstico ambiental, mapa com os pontos amostrais e as metodologias utilizadas nos estudos de campo para a caracterização da fauna terrestre e aquática.

31. A organização auditada apresenta, no diagnóstico ambiental, a caracterização da fauna terrestre e aquática, evidenciando as espécies ameaçadas de extinção, raras, endêmicas e de especial relevância ecológica e sinergética.

32. A organização auditada apresenta, no diagnóstico ambiental, a caracterização da fauna com estrutura populacional, composição, maturação sexual e função das comunidades terrestres e aquáticas, com especial atenção aos táxons bioindicadores.

> Nota: Informações básicas da guilda ecológica comumente utilizadas em listas de espécies da fauna silvestre são o tipo de *habitat* (arborícola, semiaquático, fossorial etc.); endemismo (bioma, bacia hidrográfica, Estado etc.); guilda alimentar (insetívoro, piscívoro, detritívoro etc.), entre outros.

33. A organização auditada apresenta estudos de bens e serviços ecossistêmicos pela fauna silvestre na Área Diretamente Afetada (ADA), Área de Influência (AI) e Área de Referência (AR).

> Nota: Na obtenção de produtos da natureza, é relevante considerar questões de produtividade, água para irrigação, fertilidade do solo, impactos negativos gerados pela atividade.
>
> Nota: Especial atenção deve ser dada às espécies polinizadoras, considerando sua grande importância ecológica na manutenção dos serviços ecossistêmicos.
>
> Nota: Recomenda-se que a organização auditada utilize informações sobre o equilíbrio entre a demanda dos serviços e a oferta, assegurando assim, a sua capacidade de manutenção a longo prazo.

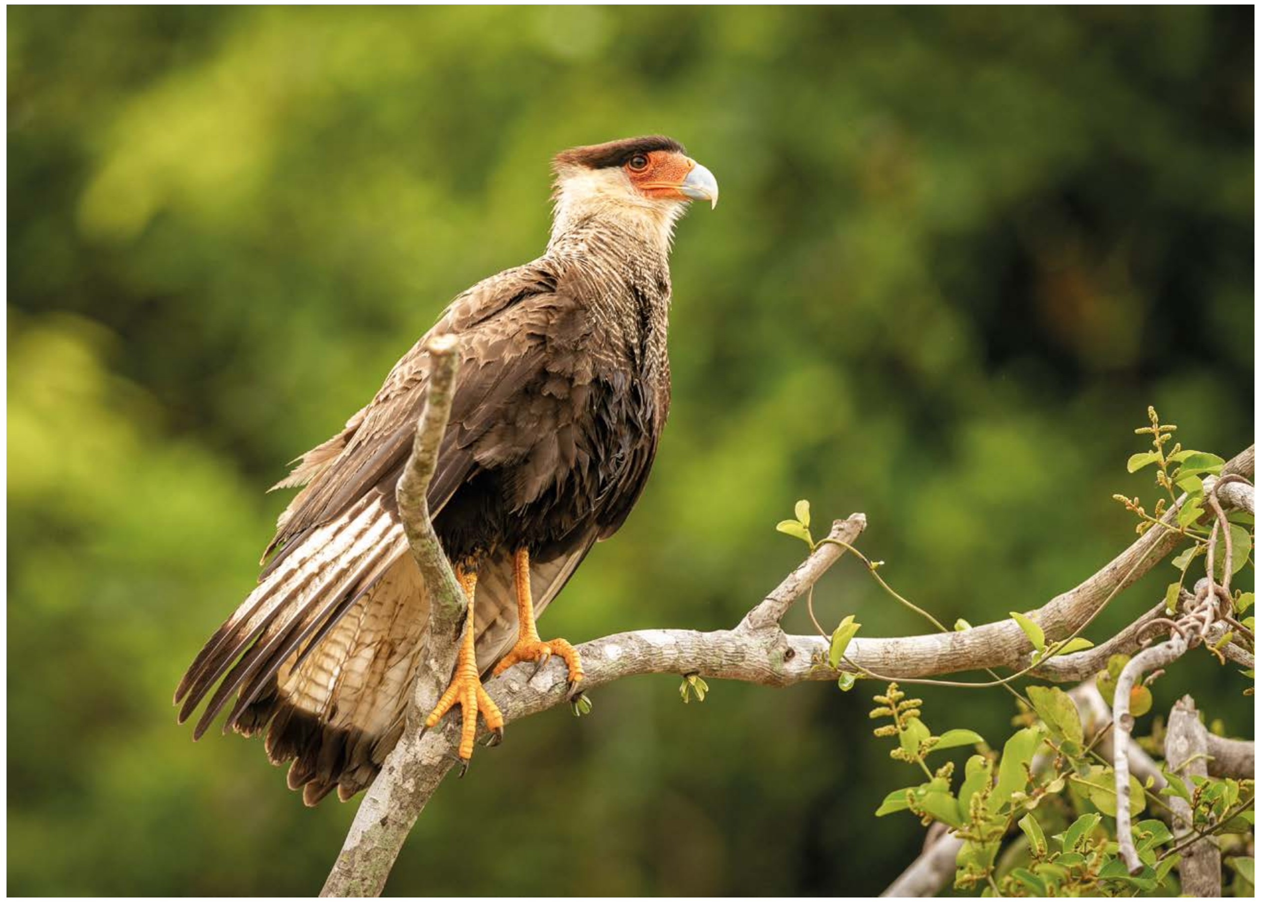

34. A organização auditada deve apresentar relatório com dados toxicológicos e ecotoxicológicos para a fauna terrestre e aquática para fins de futura avaliação de impacto ambiental, em caso de rompimento de barragem.

35. A organização auditada possui mapeamento de *habitats* de especial interesse para a conservação da fauna aquática e terrestre, incluindo rotas migratórias e seus sítios de pouso, de reprodução, nidificação ou desenvolvimento de juvenis e cavidades subterrâneas.

36. A organização auditada possui estudo de análise de conectividade terrestre e aquática para fins de caracterização da área potencialmente atingida.

37. A organização auditada elabora diagnóstico das atividades extrativistas relacionadas à fauna realizadas pelas comunidades tradicionais potencialmente atingidas.

38. A organização auditada apresenta programa de monitoramento dos impactos sobre a fauna silvestre terrestre e aquática, incluindo as informações do diagnóstico e o mapeamento das espécies bioindicadoras e prioritárias para conservação.

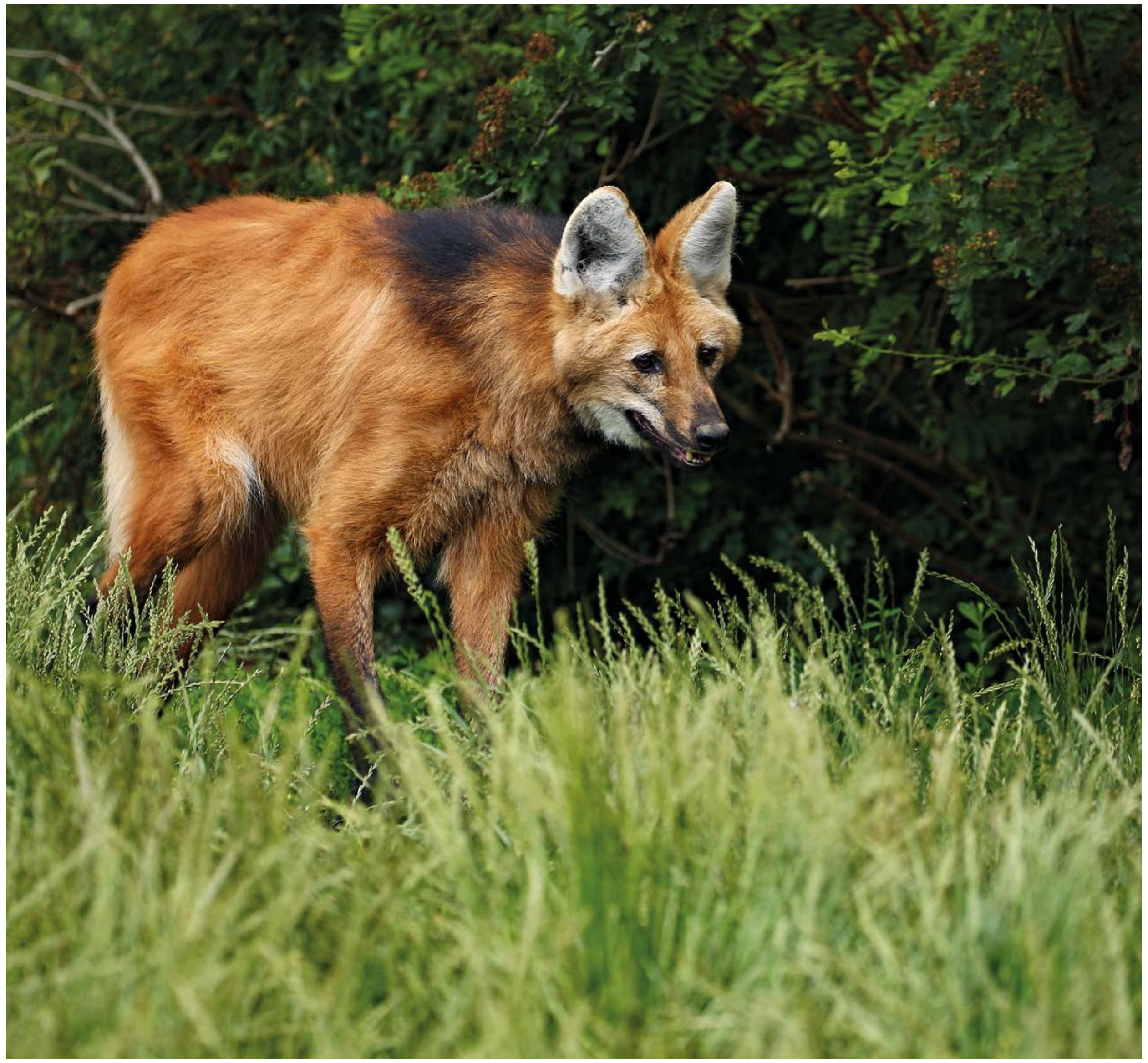

6.2 Critério V – Inventário da Fauna Doméstica, Exótica e Silvestre Cativa

DEFINIÇÃO	Instrumento de coleta de informações para o levantamento em campo de dados qualitativos e quantitativos dos animais domésticos, exóticos, de produção e silvestres cativos, além de animais errantes, classificando-os como companhia ou produção. Através das informações levantadas no inventário, deve ser elaborado um mapa com a distribuição das propriedades georreferenciadas, as espécies e o número de animais presentes no território definido como de risco.
IMPORTÂNCIA	Categorizar locais de maior adensamento animal e a posição geográfica de cada imóvel catalogado, tanto em áreas urbanas quanto em áreas rurais. O resultado do inventário pode ser utilizado para assegurar o bem-estar dos animais em todo o processo de evacuação preventiva e/ou resgate emergencial. Suas informações auxiliam ainda na determinação de medidas estratégicas para acomodação dos animais enquanto permanecem com o tutor ou sob tutela da organização auditada em abrigos. O inventário se apresenta, portanto, como um dos principais fundamentos para a elaboração do plano de ações emergenciais voltado à fauna.
ATRIBUIÇÕES DA AUDITORIA TÉCNICA	É importante que seja auditada a qualidade das informações apresentadas e o método adotado nos estudos. O produto entregue deve conter a distribuição espacial da fauna relacionada com a possível área afetada (aglomerações de animais, áreas ilhadas, acessibilidade das equipes de resgate), frequência e atualização dos dados, além de informações sobre quantidade e estado sanitário dos animais.

INDICADORES

39. A organização auditada possui inventário atualizado dos animais domésticos, em situação de rua, de produção, exóticos e silvestres cativos na área de risco.

Nota: Conforme TR da Resolução Conjunta SEMAD/FEAM/IEF/IGAM nº 3.181/2022 (Minas Gerais, 2022), os grupos de animais em situação de rua são identificados como errantes, e as espécies a serem inventariadas são cães, gatos e equídeos. Para esses animais, é aceitável que haja estimativa na área de risco, desde que documentadas as localidades de avistamento e apresentada a metodologia coerente para o cálculo.

40. A organização auditada apresenta o registro das propriedades sem informações disponíveis (seja por negativa dos proprietários ou outro motivo) no momento da realização do inventário de fauna.

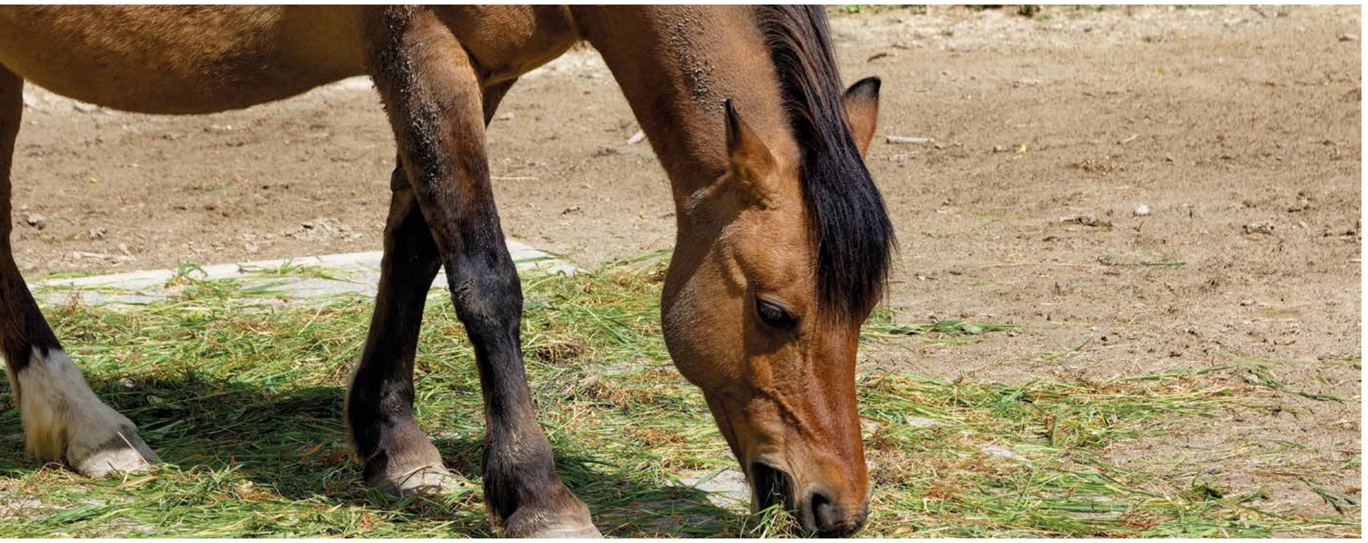

41. A organização auditada possui inventário de fauna com a distribuição das propriedades integralmente inseridas, parcialmente inseridas ou potencialmente isoladas na área de risco.

    Nota: Se possível, os dados do levantamento das propriedades podem ser integrados aos dados oficiais públicos do cadastro ambiental rural.

42. A organização auditada realiza estimativa da fauna, ou apresenta justificativa, para as áreas onde não foi possível realizar o seu inventário.

    Nota: A estimativa de fauna deve incluir informações que orientem as ações de resgate e evacuação, sejam essas executadas de forma preventiva ou emergencial.

    Nota: A estimativa de fauna deve ser capaz de caracterizar o cenário da distribuição da fauna doméstica e de produção ao longo de toda a área potencialmente atingida em caso de rompimento, detalhando valores estimados para os principais pontos de aglomeração de edificações (urbanas e rurais).

    Nota: A estimativa de fauna deverá ser complementada por estudos de caracterização do território, utilizando informações coletadas de outras fontes secundárias e ou por meio de pesquisas primárias.

    Nota: As limitações metodológicas das estimativas devem ser apresentadas de forma clara nos documentos, incluindo o detalhamento do(s) método(s) utilizado(s).

43. A organização auditada identifica e inclui estabelecimentos que abrigam grandes quantidades de animais nas ações preparatórias para emergências.

    Nota: São exemplos de locais que se enquadram neste cenário, estabelecimentos agropecuários voltados à produção animal, como granjas e sistemas pecuários, pisciculturas, abrigos públicos e privados, abrigos informais, além de instalações de tratamento veterinários e hospedagem animal.

    Nota: Locais de aglomeração de animais (clínicas veterinárias, pet hotéis, apiários) e outros grupos que dificilmente se obtém quantitativos exatos e estáveis devem receber atenção especial.

    Nota: Áreas que possam ter a necessidade de manejo de muitos animais devem ser catalogadas com endereço e estimativa de animais possivelmente abrigados.

6.3 Critério VI – Ações Preparatórias para Desastre

DEFINIÇÃO	Ações preparatórias para salvamento de animais em caso de desastre são fundamentais para garantir a segurança e o bem-estar dos animais durante situações de emergência. Essas ações incluem a elaboração de planos específicos de evacuação e resgate, que detalham rotas seguras e pontos de abrigo para os animais. Além disso, a capacitação contínua de equipes de resgate e cuidadores através de treinamentos práticos é essencial.
IMPORTÂNCIA	As ações preparatórias são necessárias no contexto de fomentar ou intensificar a articulação entre diferentes atores sociais e a formação de redes de proteção. As práticas de simulados, por sua vez, também ajudam a mobilização da comunidade e possibilitam identificar e corrigir possíveis falhas. Estas são ações relevantes para minimizar a ocorrência de danos à saúde ou à vida de cada espécie animal em um cenário de desastre.
ATRIBUIÇÕES DA AUDITORIA TÉCNICA	Auditar as ações preparatórias aos desastres consiste em verificar todo o processo, do planejamento à destinação dos animais, objetivando o bem-estar de cada espécie em todas as fases dessas ações. É importante verificar se o fluxo de comunicação em uma ação emergencial está claro, completo e atualizado e se as equipes destinadas à governança estão treinadas para tomar decisões eficazes.

É importante considerar a Portaria IBAMA nº 2.489/2019 (Brasil, 2019) que altera o parágrafo único do art. 1º da Portaria IBAMA nº 93/1998 (Brasil, 1998) e lista os animais considerados domésticos para efeito de operacionalização do IBAMA.

As boas práticas para um plano de resgate de fauna estão estabelecidas na Instrução Normativa IBAMA nº 146/2007 (Brasil, 2007), elaborada em razão de resgates de animais silvestres, e também nos protocolos estabelecidos pelo CFMV (Gomes *et al.*, 2020; Oliveira *et al.*, 2020) e pela literatura técnica (Pinto, 2021), para o resgate da fauna doméstica de companhia e de produção, incluindo ictiofauna. De acordo com a normativa, alguns tópicos devem constar nos planos de resgate, como a descrição da estrutura física da área de risco, suas localizações e vias de acesso; a descrição e quantificação dos equipamentos utilizados; a composição das equipes de resgate; o programa do curso de capacitação pessoal para a equipe de resgate; a destinação pretendida para cada grupo animal da fauna resgatada; o detalhamento da captura, triagem e demais procedimentos para coleta de exemplares vivos ou mortos, informando o tipo de identificação individual, registro e biometria.

INDICADORES

44. A organização auditada considera o diagnóstico das espécies obtido nos estudos de linha de base, para a definição de áreas prioritárias para o planejamento das ações emergenciais voltadas à fauna silvestre terrestre e aquática de vida livre.

45. A organização auditada apresenta plano de resgate adequado para a fauna silvestre terrestre e aquática de vida livre em casos de emergência, com as diretrizes determinadas pelos órgãos ambientais, considerando as etapas de captura, transporte e destinação.

46. A organização auditada apresenta plano de evacuação adequado para a fauna doméstica, silvestre e exótica cativa em casos de emergência, com as diretrizes determinadas pelos órgãos ambientais, considerando as etapas de captura, transporte e destinação.

47. A organização auditada apresenta medidas de mitigação de impactos à fauna silvestre terrestre e aquática.

48. A organização auditada promove a participação social no planejamento de ações educativas e preparatórias para a evacuação de comunidades potencialmente atingidas, considerando o contexto cultural dos tutores com os animais.

Nota: As informações específicas dizem respeito ao cenário de cada local, considerando o modo de vida, a cultura de cada comunidade e o ambiente, incluindo a fauna e a flora local.

Nota: Devem incluir grupos tradicionais como indígenas, quilombolas, agricultores familiares, entre outros.

49. A organização auditada executa simulados de evacuação com as comunidades, contendo orientações sobre as tratativas a serem realizadas com cada espécie animal em caso de emergência, além de fomentar a participação dos animais domésticos de companhia junto aos seus tutores.

Nota: A organização auditada deve incentivar a participação dos animais nos simulados, destacando junto à comunidade as ações a serem realizadas com aqueles que não poderão ser evacuados simultaneamente com os humanos. Por exemplo, não manter os animais presos e garantir que porteiras e gaiolas de animais nativos estejam abertas, ou ainda que aquários e gaiolas de animais exóticos sejam posicionados nos pontos mais altos da residência.

Nota: Nos pontos de encontro, a auditada deve disponibilizar equipes capacitadas para atender os animais, bem como acessórios para contenção e acomodação.

Nota: As orientações sobre os procedimentos de evacuação em caso de emergência devem ser adaptadas à realidade de cada área, incluindo, por exemplo, ações cabíveis para populações localizadas na ZAS tendo em vista o tempo previsto de chegada dos rejeitos às residências e outras edificações.

Nota: As ações de orientação junto à população devem ser articuladas junto à Defesa Civil e às demais instituições públicas de interesse a fim de que as ações de autossalvamento sejam articuladas às ações de salvaguarda da fauna, dentro dos princípios da dignidade animal e da guarda responsável.

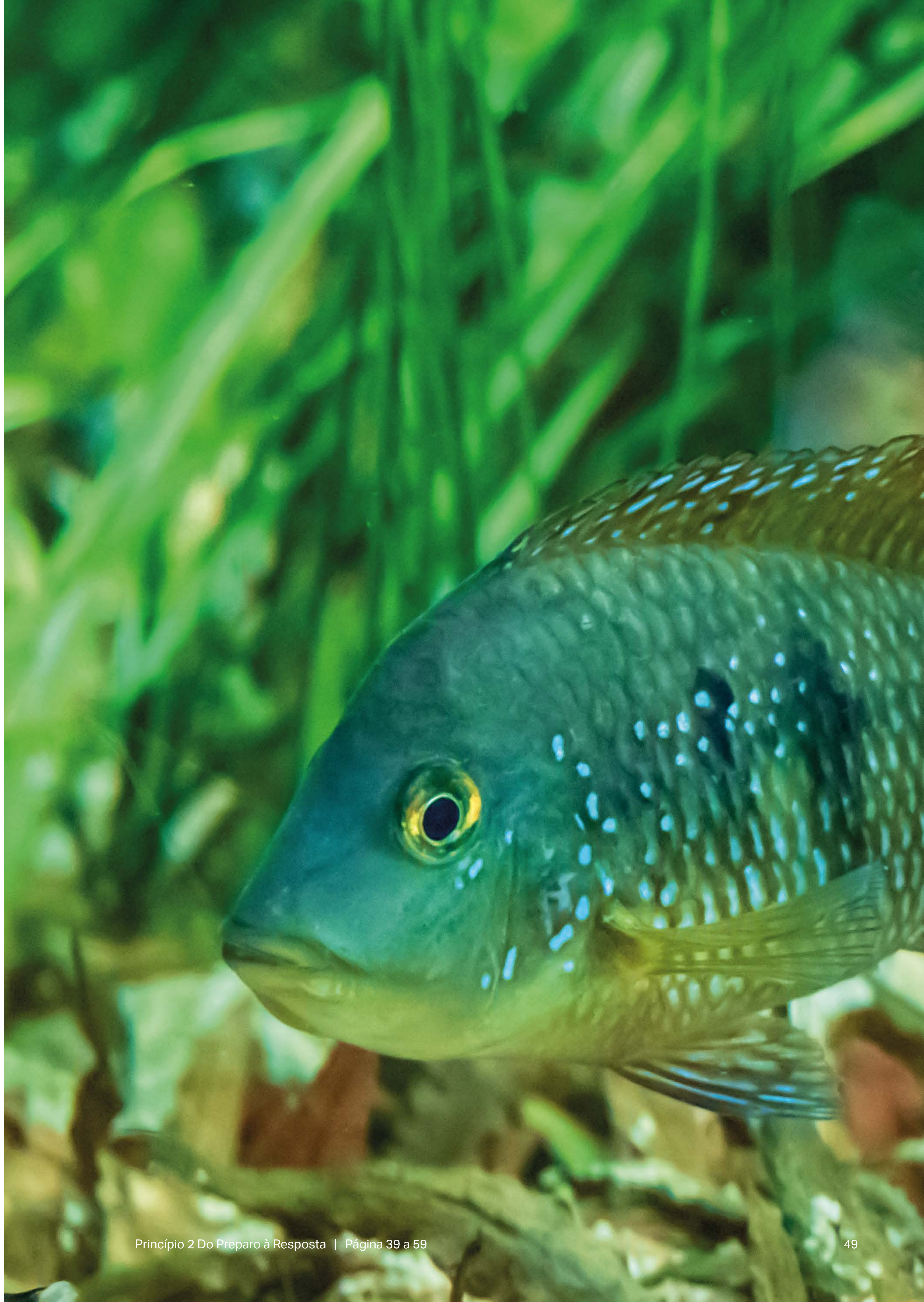

50. A organização auditada disponibiliza cartilhas à comunidade, contendo orientações quanto à guarda responsável, ao manejo alimentar e comportamental básicos de acordo com a espécie, além de orientações sobre as ações a serem realizadas durante uma evacuação emergencial ou programada.

> Nota: A auditada deve fornecer e incentivar as comunidades residentes em áreas de risco o uso de adesivo de segurança do tipo *Pet Alert* em suas residências para facilitar o resgate de animais em caso de emergência.

51. A organização auditada apresenta metodologia de análise de eficiência para as ações preparatórias para a evacuação de comunidades e usa essas informações para as adequações no planejamento dessas atividades.

6.4 Critério VII – Ações de Resposta Emergencial

DEFINIÇÃO	Em um cenário de desastre, as ações de resposta emergencial são as intervenções imediatas realizadas para proteger a vida, minimizar danos e fornecer assistência básica em situações de calamidade. Dentre essas ações, se destacam aquelas voltadas para o resgate, o salvamento e a assistência dos animais atingidos, busca e identificação dos tutores, coordenação e comunicação entre os agentes e as organizações.
IMPORTÂNCIA	Essas ações são fundamentais para mitigar os impactos negativos do desastre e facilitar o processo de recuperação e reconstrução a longo prazo. Resgates rápidos e eficientes, além da provisão de cuidados médicos emergenciais, podem reduzir significativamente o número de fatalidades e lesões graves em animais e humanos. As ações emergenciais contribuem para estabilizar a situação, evitando que o desastre cause danos adicionais.
ATRIBUIÇÕES DA AUDITORIA TÉCNICA	Avaliar as ações de resposta emergencial consiste na revisão sistemática das estratégias e procedimentos planejados para responder a desastres e emergências. As instruções e procedimentos devem ser claros e compreensíveis; cada medida precisa estar em conformidade com normas e regulamentos; a análise de risco deve ser revisada para identificar vulnerabilidades potenciais; os recursos (humanos, materiais, financeiros) devem estar adequadamente identificados e disponíveis; por fim, é necessário identificar pontos de melhoria.

A organização auditada deverá realizar o resgate de animais domésticos terrestres e aquáticos de produção, e silvestres mantidos em cativeiro na área afetada pelo desastre, seguindo as diretrizes e os protocolos já estabelecidos pelo CFMV, a exemplo da Resolução nº 1.511/2023, entre outras (Gomes *et al.*, 2020; Oliveira *et al.*, 2020; Brasil, 2023) e pela literatura técnica (Pinto, 2021), visando o bem-estar de cada indivíduo, seguindo as legislações pertinentes.

INDICADORES

52. A organização auditada implementa meios para o cessamento no menor tempo tecnicamente possível da fonte de impacto após o desastre ambiental.

53. A organização auditada implementa medidas necessárias para o isolamento da Área Diretamente Afetada (ADA) para restringir o acesso de pessoas e animais.

> Nota: Em caso de desastres, deve-se prever ações de afugentamento da fauna, considerando os métodos específicos e eficazes apresentados na literatura para cada espécie, sempre com a anuência do órgão ambiental.
>
> Nota: Para indivíduos e/ou populações onde o afugentamento não é aplicável, deve ser realizado o resgate e a translocação para área segura.

54. A organização auditada implementa e monitora ações de dessedentação e alimentação animal nas áreas atingidas, ilhadas ou de difícil acesso, em caso de desastre.

> Nota: Recomenda-se que a organização auditada possua métricas de efetividade dos pontos de dessedentação.

55. A organização auditada instala medidas para deslocamento autônomo da fauna silvestre de vida livre em áreas atingidas pelo desastre.

> Nota: Entende-se como medidas para deslocamento autônomo, as passagens de fauna aéreas e terrestres, corredores ecológicos, entre outras.

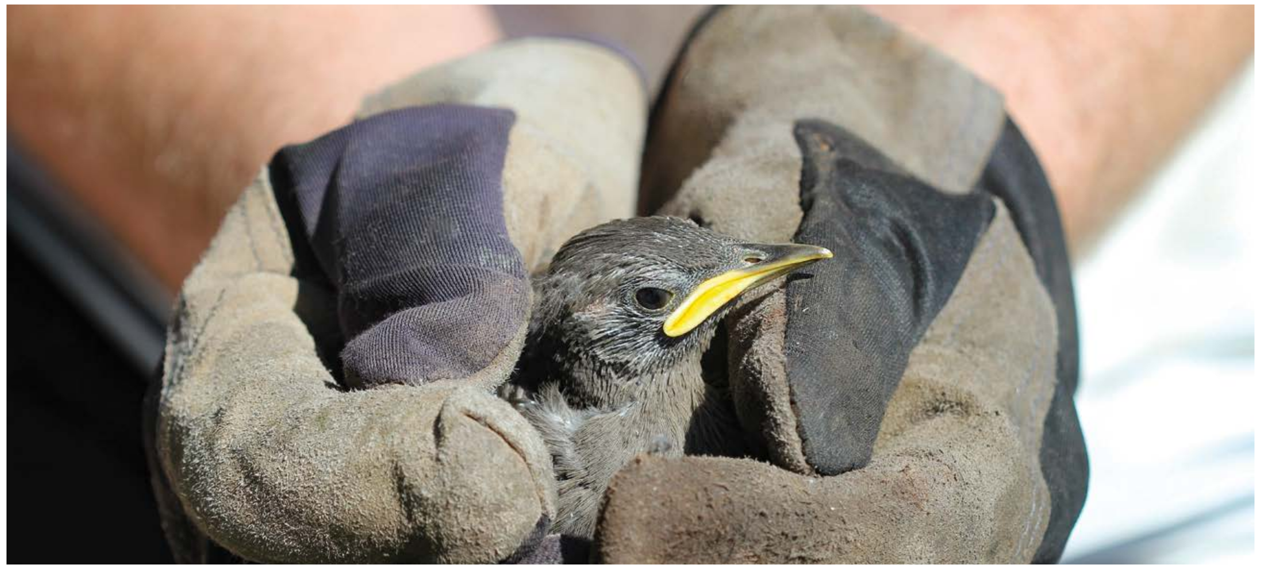

56. A organização auditada possui plano de acessibilidade com rotas rápidas para as equipes de campo executarem as ações emergenciais de salvamento nas áreas prioritárias para a fauna.

57. A organização auditada realiza resgate e evacuação da fauna de vida livre adotando métodos que reduzem o estresse dos animais.

> Nota: Em caso de resgate e soltura imediata de animais silvestres, a área de soltura deve ser a estabelecida de acordo com o órgão ambiental.
>
> Nota: Em caso de resgate de animais silvestres mantidos em cativeiro ilegal, o órgão ambiental competente deverá ser comunicado. Só estejam à vida livre após avaliação de equipe técnica especializada, deve encaminhá-los para Programas de Reabilitação e Reintrodução. Os indivíduos que não estiverem aptos deverão ser destinados para mantenedores definitivos, de acordo com as diretrizes dos órgãos ambientais.
>
> Nota: Em caso de resgate e soltura imediata de animais silvestres, a área de soltura deve ser a estabelecida de acordo com o órgão ambiental.
>
> Nota: No caso de resgate de espécies exóticas invasoras, a destinação adequada deverá ser avaliada junto ao órgão ambiental responsável.
>
> Nota: Os planos de ação emergencial deverão conter a identificação de possíveis áreas de soltura na área de interesse, antecipando e atuando de forma propositiva junto ao órgão ambiental em caso de rompimento.

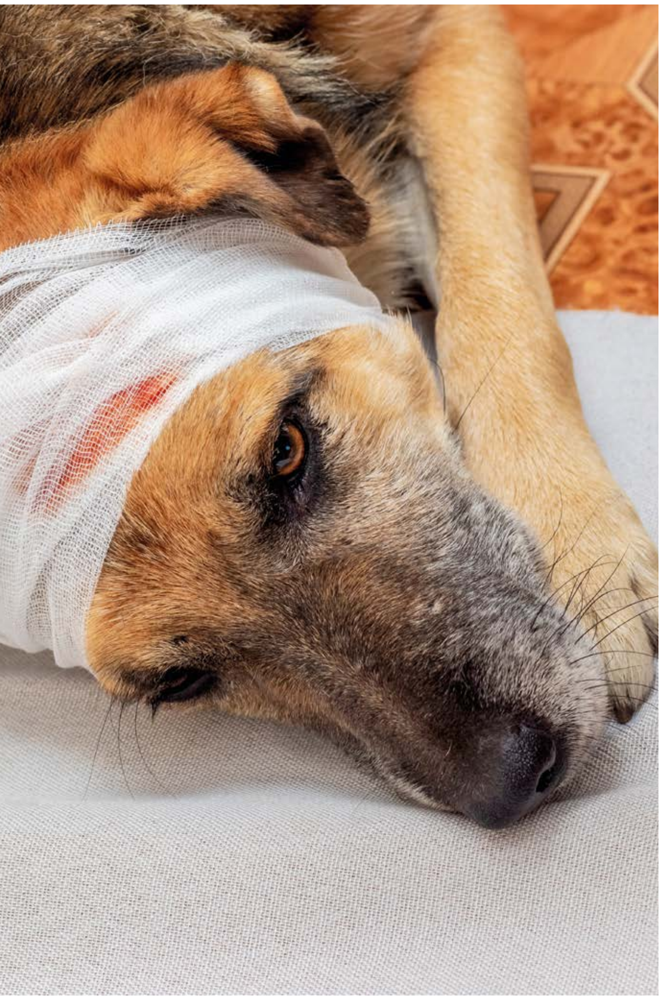

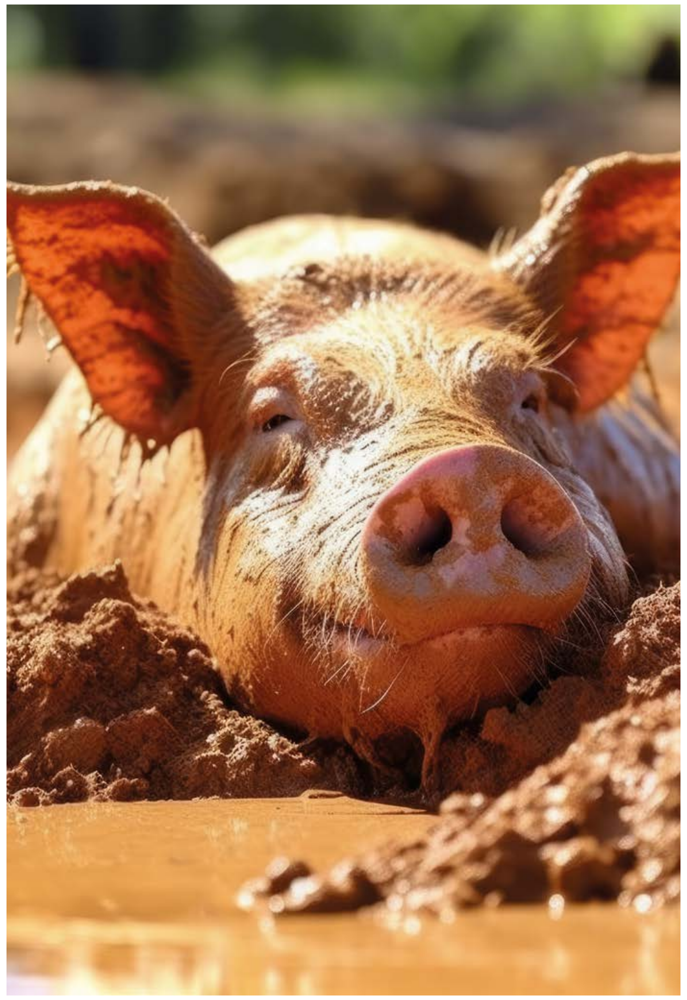

58. A organização auditada apresenta protocolos de triagem e isolamento dos animais doentes ou feridos no momento do resgate ou evacuação.

59. A organização auditada realiza o transporte adequado de todos os animais resgatados/evacuados, de acordo com as necessidades de cada espécie e a sua destinação.

> Nota: O transporte dos animais pode ocorrer de maneira coletiva, desde que seja previamente avaliada a viabilidade sanitária (procedimento padrão sanitário) e a origem dos animais (oriundos do mesmo plantel ou propriedade).

60. A organização auditada realiza resgate e evacuação da fauna aquática com segurança.

61. A organização auditada realiza avaliação de sanidade da ictiofauna no momento da evacuação ou resgate.

> Nota: Em caso de evacuações programadas, o controle sanitário efetivo e rápido deve ocorrer sempre que possível para evitar perdas significativas causadas por enfermidades que podem afetar seriamente as populações de peixes em cativeiro (Pavanelli *et al.*, 2008). Para tanto, deve-se considerar que os grupos devem ser preservados, evitando inserir peixes de outros grupos.

62. A organização auditada implementa o programa de prospecção de fauna no entorno da área impactada pelo desastre.

63. A organização auditada disponibiliza relatórios periódicos dos programas de manejo de fauna realizados durante as ações emergenciais.

64. A organização auditada disponibiliza atendimento assistencialista para a fauna atingida que permanece na área afetada pelo desastre até o restabelecimento total da comunidade.

> Nota: O assistencialismo deve considerar atendimento médico-veterinário, fornecimento de alimentação, água de boa qualidade e insumos.

65. A organização auditada recolhe, registra e aplica protocolo de exame *post mortem* nas carcaças de animais encontrados durante as ações emergenciais, para utilização na avaliação de impacto ambiental.

66. A organização auditada considera áreas com *habitats* adequados às espécies, em caso de translocação ou soltura de animais silvestres.

67. A organização auditada realiza resgate de abelhas (nativas e exóticas), realocação e encaminhamento das colmeias para abrigo apropriados.

6.5 Critério VIII – Avaliação, Mitigação e Compensação do Impacto à Fauna Atingida

DEFINIÇÃO	Avaliar as alterações do meio ambiente ocasionados pelo desastre, estabelecendo os impactos que afetam direta ou indiretamente a fauna doméstica e silvestre, sejam eles positivos ou negativos. Para a avaliação de impacto, é relevante que se tenha um diagnóstico da área, com a caracterização do meio físico, biológico e socioeconômico. Após a avaliação, é necessário definir as medidas mitigatórias para reduzir a gravidade e extensão dos impactos. Posteriormente, são desenvolvidas medidas visando o retorno à situação de normalidade.
IMPORTÂNCIA	Os impactos ocorridos em função de um desastre podem ocasionar desequilíbrio ecológico e redução de populações faunísticas. Em casos de desastres ambientais, as alterações podem implicar em perda direta de serviços ambientais, que são importantes para a funcionalidade dos serviços ecossistêmicos, causando danos à manutenção da biodiversidade e gerando implicações à saúde pública e economia das comunidades locais. Dessa maneira, a definição dos impactos negativos gerados por um rompimento de barragem permite, entre outras coisas, adoção de medidas para corrigi-los ou minimizá-los. Além disso, é possível estabelecer programas de acompanhamento e monitoramento da área afetada para que o ecossistema retorne o mais próximo possível da sua condição original.
ATRIBUIÇÕES DA AUDITORIA TÉCNICA	Auditar os planos de avaliação e mitigação de impacto para a fauna apresentados pela organização auditada. Verificar os limites geográficos dos impactos e identificá-los como diretos e/ou indiretos, imediatos, a médio e longo prazo, temporários e permanentes, reversível ou não, cumulativo ou não. Deve-se avaliar a eficiência das medidas mitigadoras e dos programas propostos, além dos efeitos esperados com sua implementação. Adicionalmente, é relevante que se verifique a avaliação de danos aos serviços ecossistêmicos na área atingida.

INDICADORES

68. A organização auditada possui um programa de avaliação de impactos voltado à fauna doméstica, incluindo animais de produção e errantes.

    Nota: Como impactos à fauna doméstica, destacam-se desabrigo e injúria de indivíduos; morte de indivíduos; perda de áreas de pastagem; restrição do acesso à água a animais utilizados na produção, entre outros.

    Nota: No aspecto sociocultural, é relevante considerar os impactos às festividades tradicionais que incluem animais e ruptura do vínculo humano-animal, especialmente para as famílias multiespécies.

69. A organização auditada possui programa de avaliação de impactos ambientais sobre a fauna silvestre terrestre e aquática.

    Nota: Como impactos sobre fauna silvestre, citam-se: morte de indivíduos; injúria de indivíduos; efeitos de toxicidade e bioacumulação em indivíduos, população e comunidades; perda e redução da qualidade de *habitat*; redução da permeabilidade faunística; alteração da composição e estrutura das comunidades, entre outros.

    Nota: Os estudos devem ser realizados de forma comparativa entre os ambientes (ADA e AR) com método detalhado e os programas propostos elencados a cada um dos impactos identificados para a fauna.

    Nota: No programa de avaliação de impactos, deve estar incluído a análise limnológica integrada à fauna aquática.

    Nota: O programa de avaliação de impactos à biodiversidade deve considerar grupos vetores e fauna sinantrópica.

    Nota: A avaliação dos impactos à biodiversidade faunística deve ser analisada em conjunto com os estudos de impacto à flora.

70. A organização auditada adota métricas para a avaliação dos impactos à provisão de bens e serviços ecossistêmicos pela fauna silvestre na Área Diretamente Afetada (ADA), Área de Influência Direta e Indireta (AI), em caso de desastre.

    Nota: Importante considerar a integração dos impactos, por exemplo, na privação da pesca em comunidades tradicionais (cultural), cabe associar o impacto sobre as espécies de peixes do local (provisão), assim como o impacto sobre a manutenção do ecossistema aquático (regulação).

71. A organização auditada possui estimativa da mortandade total de animais ou biomassa, quando adequado para cada grupo taxonômico.

    Nota: A organização auditada possui uma estimativa de mortandade da fauna (número de indivíduos ou biomassa), caso não seja possível identificar o número de animais que foram a óbito, através da contabilidade das carcaças.

72. A organização auditada adota métricas para a avaliação de impacto aos corpos d'água usualmente utilizados para dessedentação animal.

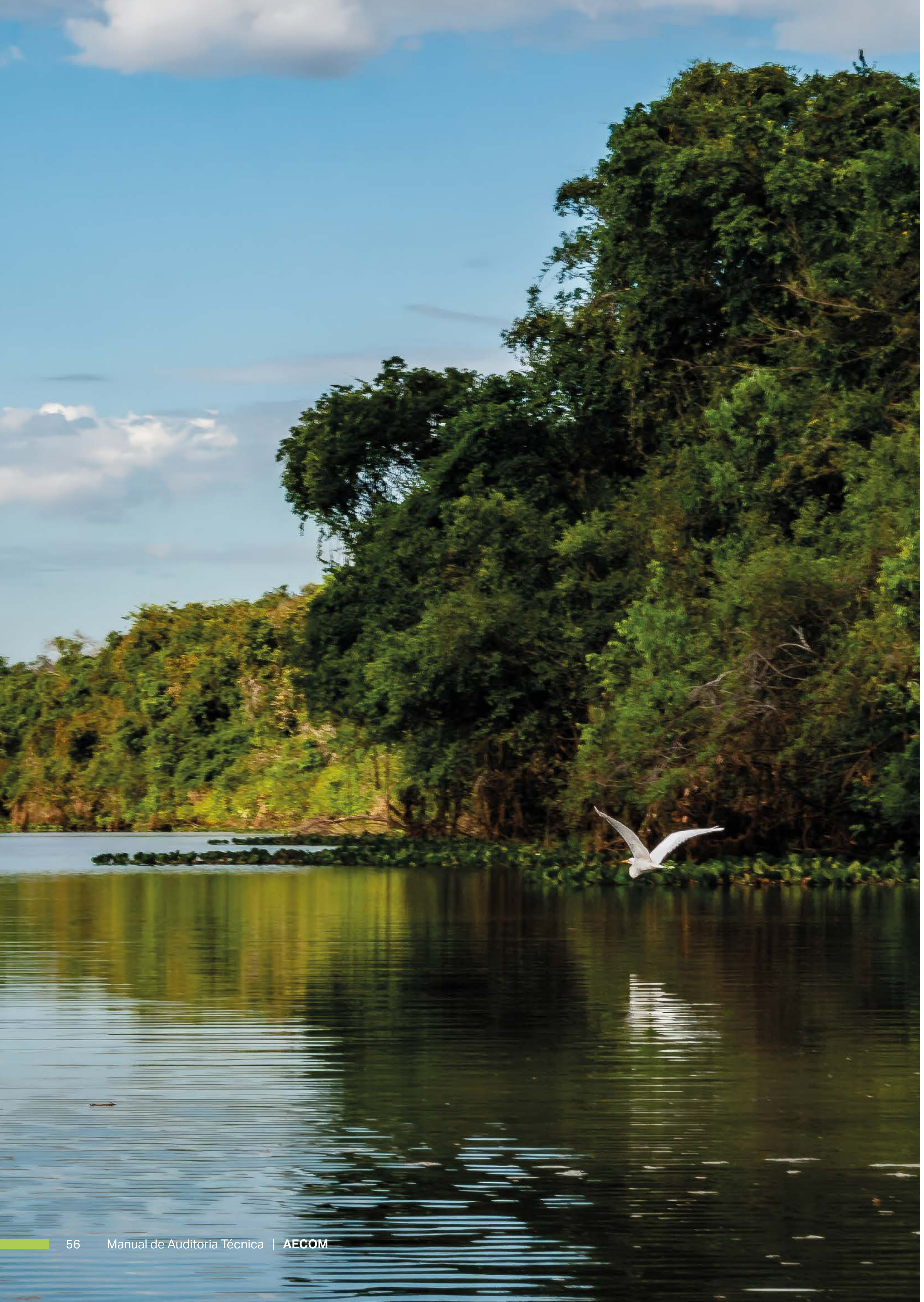

73. A organização auditada inclui espécies polinizadoras nos estudos de impacto da fauna atingida.

> Nota: Cabe ressaltar que para além dos insetos, como abelhas, borboletas, besouros, também são considerados polinizadores algumas espécies de aves, répteis e mamíferos.

74. A organização auditada implementa um programa de monitoramento dos impactos sobre a fauna silvestre terrestre e aquática.

> Nota: Recomenda-se que a organização auditada considere a inclusão de *táxons* específicos que respondam de modo previsível às alterações ambientais (bioindicadores).

75. A organização auditada possui programas de monitoramento específicos para as espécies-chave definidas através dos estudos de linha de base.

76. A organização auditada evidencia a realização de ações voltadas a mitigar os impactos aos animais domésticos, incluindo aspectos socioeconômicos.

> Nota: A organização auditada deve elaborar programas para avaliação e redução de impactos nas comunidades rurais.
>
> Nota: A auditada deve implementar programas de resiliência das comunidades e retomada das atividades do setor agropecuário.
>
> Nota: O Manual de Boas Práticas no Abrigamento de Cães e Gatos em Situações de Desastre (Gebara *et al.*, 2024) apresenta diretrizes fundamentais para a organização e gestão de abrigos.

77. A organização auditada implementa um programa com métricas para a valoração de danos aos serviços ecossistêmicos após desastre.

> Nota: Como danos aos serviços ecossistêmicos, podem ser considerados: redução da segurança alimentar; redução dos estoques pesqueiros; redução da polinização e dispersão de sementes; aumento da incidência de doenças relacionadas à fauna sinantrópica, entre outros.
>
> Nota: A valoração dos serviços ecossistêmicos deve considerar os costumes e valores culturais das comunidades atingidas (condições socioeconômicas, educacionais, ocupacionais, idade, gênero, cultura intragrupo, entre outros).
>
> Nota: A incorporação da comunidade durante o processo de coleta das informações confere melhor entendimento da tradição, do conhecimento ecológico e ajuda a manter o patrimônio cultural, sua identidade e seus valores.

6.6 Critério IX – Reabilitação e Reintrodução de Animais Silvestres

DEFINIÇÃO	A reabilitação da fauna silvestre visa o atendimento de animais com injúrias físicas, para a recuperação dos seus comportamentos naturais, seja para a soltura no ambiente ou para adaptação às suas novas condições de sobrevivência. Podem ocorrer três formas de reabilitação: física, fisiológica e comportamental.
IMPORTÂNCIA	No processo de reabilitação, são desenvolvidas ações que ajudam na preparação dos animais manejados para o retorno à vida livre. No caso de espécimes da fauna silvestre resgatados em situação emergencial, a reintrodução é uma ferramenta que permite devolver o animal à condição de vida livre, desde que o indivíduo se apresente saudável, que haja áreas naturais adequadas à espécie e aderentes à quantidade de animais resgatados.
ATRIBUIÇÕES DA AUDITORIA TÉCNICA	O objetivo geral é analisar a eficiência do processo de reabilitação, a infraestrutura, os equipamentos e a equipe da instituição responsável pelo processo.<br>A equipe de auditores deve ter conhecimentos gerais sobre a legislação ambiental pertinente, a clínica médica de animais silvestres, o comportamento de animais silvestres, técnicas empregadas no processo de reabilitação e da avaliação de aptidão comportamental.

A reintrodução é o tipo mais comum de translocação conservacionista e corresponde à soltura intencional de organismos na natureza com fins de conservação. Inclui ainda a colonização assistida e a substituição ecológica (quando a soltura não é realizada dentro da área de distribuição original da espécie) e o reforço (quando a soltura ocorre dentro da área de distribuição original, mas já existe uma população da espécie presente na área de soltura) (IUCN, 2013). Cabe ressaltar que as ações de reabilitação e reintrodução de animais silvestres estão condicionadas à deliberação dos órgãos ambientais responsáveis pela aprovação dos planos de reabilitação (a serem elaborados pelas organizações auditadas) e locais de recondicionamento físico e comportamental, área de soltura e periodicidade do monitoramento.

A manutenção dos estímulos de comportamentos naturais a uma espécie durante o período em cativeiro, como a busca por alimento em situações semelhantes à vida livre (por exemplo, caça, forrageamento, disputas com outros indivíduos) e fuga de predadores (incluindo a desassociação com humanos) são essenciais para a readaptação e sobrevivência desses indivíduos na natureza.

Ressalta-se que os Cinco Domínios do Bem-estar Animal também devem ser respeitados e implementados para o cativeiro de animais silvestres, mesmo que transitório. Portanto, os critérios do Princípio 4 – Bem-estar do Animal Abrigado, também são avaliados em conjunto com este Critério.

INDICADORES

78. A organização auditada possui Projeto de Reabilitação e Reintrodução protocolado junto ao órgão ambiental para cada animal silvestre sob sua tutela e que apresente avaliação de aptidão para soltura na natureza, considerando o menor tempo possível no cativeiro até a sua destinação para o Programa.

    Nota: Exemplos de condições comuns que demandam atividades de reabilitação prévia à soltura são a manutenção do animal em cativeiro por períodos prolongados; indivíduos resgatados ainda jovens/filhotes; restrições físicas, clínicas e comportamentais temporárias (exemplos são as aves jovens com penugem incompleta para o voo ou animais adultos com a capacidade de voo comprometida pela restrição do cativeiro); entre diversas outras condicionantes.

79. A organização auditada disponibiliza relatórios periódicos do Programa de Reabilitação e Reintrodução de animais silvestres, incluindo as etapas de monitoramento pós-soltura.

80. A organização auditada, especializada em reabilitação e reintrodução de animais, possui recintos com presença de barreira verde, distanciamento adequado de animais domésticos e design apropriado para a espécie abrigada.

    Nota: Os recintos devem estar distantes de áreas com fluxo de pessoas ou de automóveis.

81. A organização auditada registra, nos prontuários individuais dos animais silvestres abrigados, as avaliações etológicas periódicas.

82. A organização auditada deve apresentar parecer técnico oficial ou licença expedida pelos órgãos ambientais para transporte, translocação e soltura de espécies nativas.

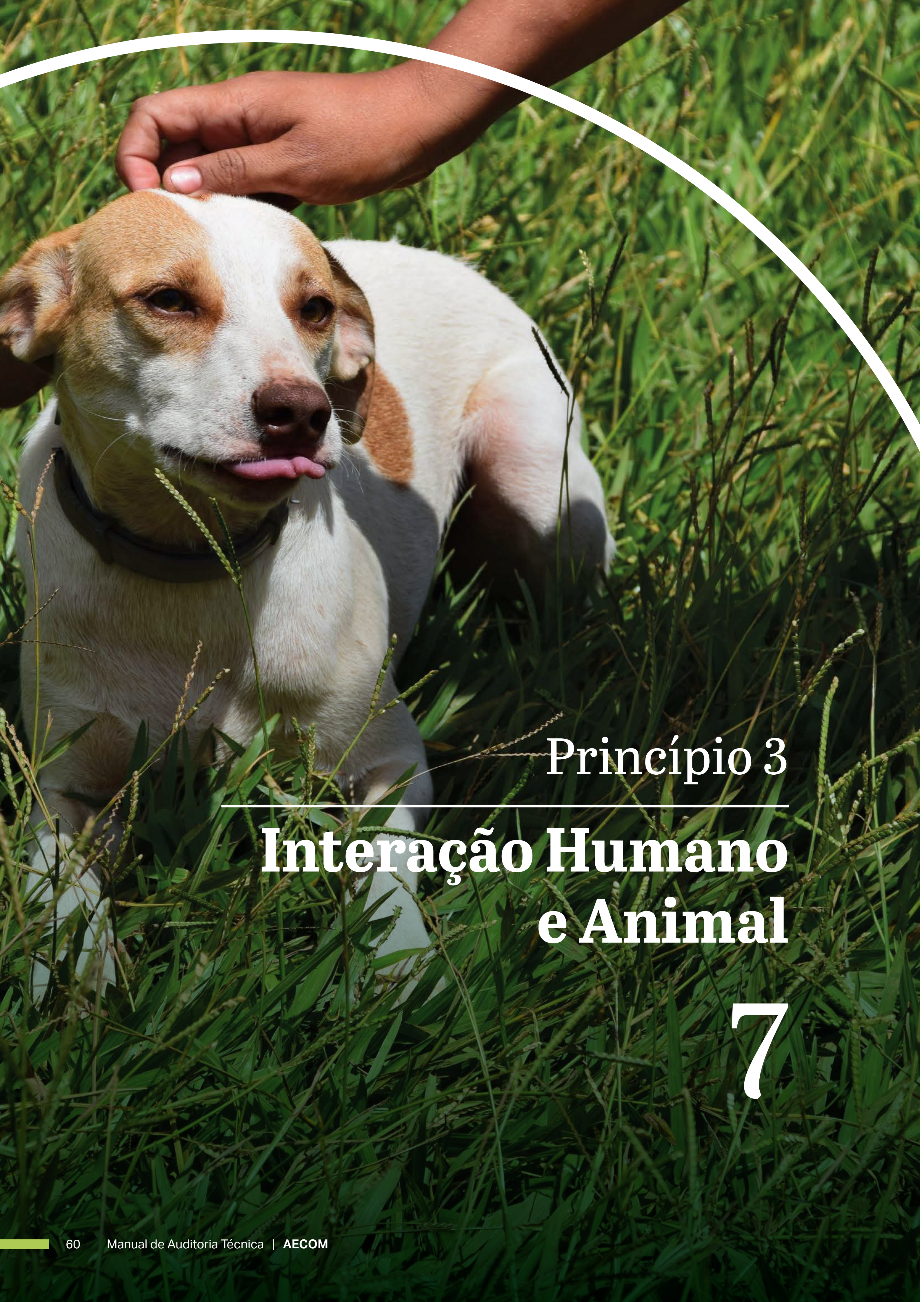

Princípio 3

Interação Humano e Animal

7

7. Princípio 3 Interação Humano e Animal

Parte-se da premissa que na sociedade contemporânea há indissociabilidade das relações com os animais de companhia, de produção e silvestres que participaram ativamente na organização e ocupação do território, na escolha e definição dos locais de implementação de empresas e indústrias, no estabelecimento de economias agro-pastoris, na consolidação de núcleos humanos ou mesmo em casos da promoção à sustentação de tradições culturais (Animal Ethics, 2024).

Nesse sentido, há a necessidade de assegurar que essa interação ocorra de forma equilibrada e voltada ao bem-estar das espécies. No Princípio Interação Humano e Animal, no contexto do processo de auditoria técnica voltado à proteção da fauna, são elencados três Critérios avaliativos, conforme a Figura 11.

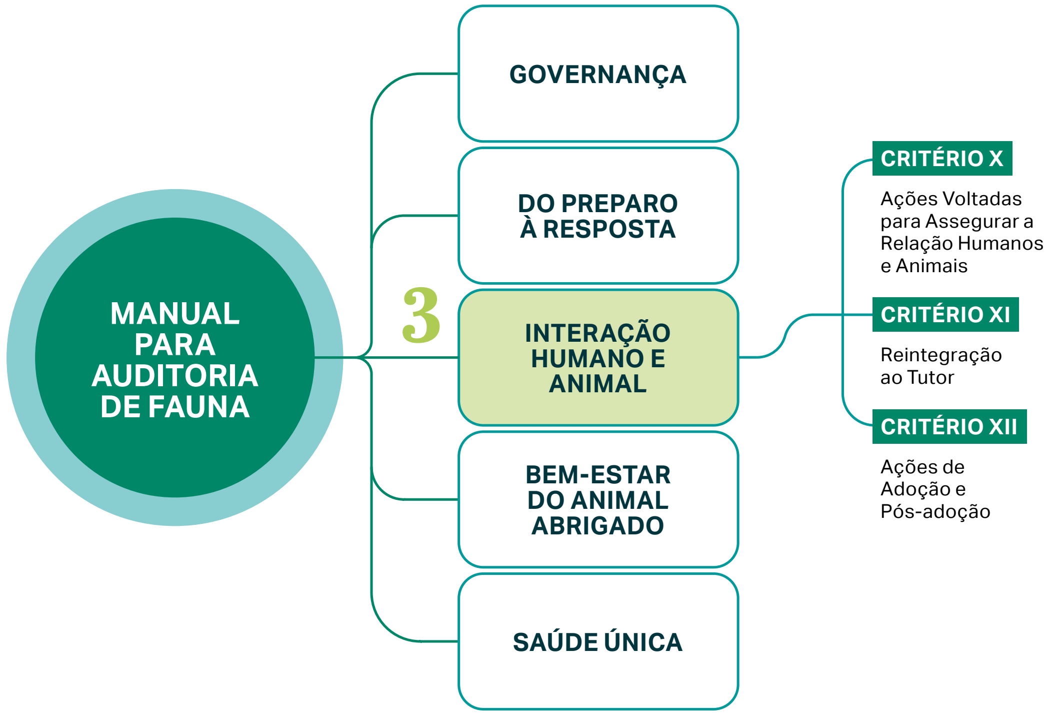

Figura 11 – Critérios avaliativos do Princípio Interação Humano e Animal.

7.1 Critério X – Ações Voltadas para Assegurar a Relação Humanos e Animais

DEFINIÇÃO	A relação entre o ser humano e outros animais envolve aspectos diversos tanto de relações comunitárias e interação social quanto de promover em seus tutores sentimentos de companheirismo e segurança (*zooeyia*), que devem ser consideradas nos eventos de evacuação e resgate de fauna.
IMPORTÂNCIA	Como forma de mitigação do impacto, assegurar que a relação tradicionalmente estabelecida entre os tutores originais e os animais seja respeitada é de suma importância para legitimar os cuidados e respeitar os direitos de todos atingidos, sejam eles humanos ou não humanos.
ATRIBUIÇÕES DA AUDITORIA TÉCNICA	Auditar as ações previstas que assegurem aos tutores seu direito de obter informações e participar das decisões que envolvam o manejo e cuidados com seus animais durante a permanência em abrigo.

INDICADORES

83. A organização auditada demonstra que prioriza, sempre que possível, a permanência dos animais de companhia junto aos seus tutores durante as ações de evacuação de território.

    Nota: No caso do animal ser mantido com o tutor, é necessário que a organização auditada assegure a identificação individual, de acordo com a permissão do tutor.

    Nota: Animal silvestre em cativeiro irregular, ainda que tido como animal de companhia por seu tutor, deverá ter sua destinação avaliada pelo órgão ambiental.

84. A organização auditada demonstra que promove a destinação final dos animais abrigados no menor tempo possível.

    Nota: Deve considerar a reabilitação rápida de animais silvestres como ponto crucial para garantir sua soltura e evitar o *imprinting*, preservando seu comportamento natural e aumentando suas chances de sobrevivência no *habitat* selvagem. Já para animais domésticos, encontrar o tutor de um animal resgatado o mais rápido possível é fundamental para assegurar os laços afetivos existentes e promover o bem-estar emocional do animal e de seu tutor.

85. A organização auditada possui meios de comunicação adequados à realidade dos tutores dos animais sob a sua tutela.

    Nota: É importante que o tutor seja informado de qualquer mudança de localização de seu animal, seja ela para outro abrigo ou clínica veterinária.

    Nota: Todos os procedimentos clínicos realizados com os animais devem ser informados aos tutores.

86. A organização auditada possui um programa de relacionamento com os tutores dos animais abrigados, permitindo que a relação tradicionalmente estabelecida entre tutores e seus animais seja respeitada.

87. A organização auditada promove ações que facilitam a visitação dos tutores aos seus animais abrigados.

88. A organização auditada possui áreas de visitação para momentos de recreação entre animais abrigados e seus tutores, de acordo com cada espécie animal.

    Nota: No caso de cavalos, as atividades de equitação e cavalgada com seus tutores devem ser estimuladas.

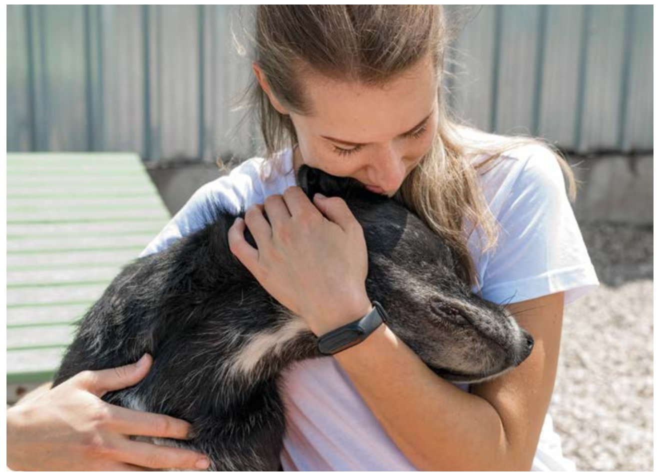

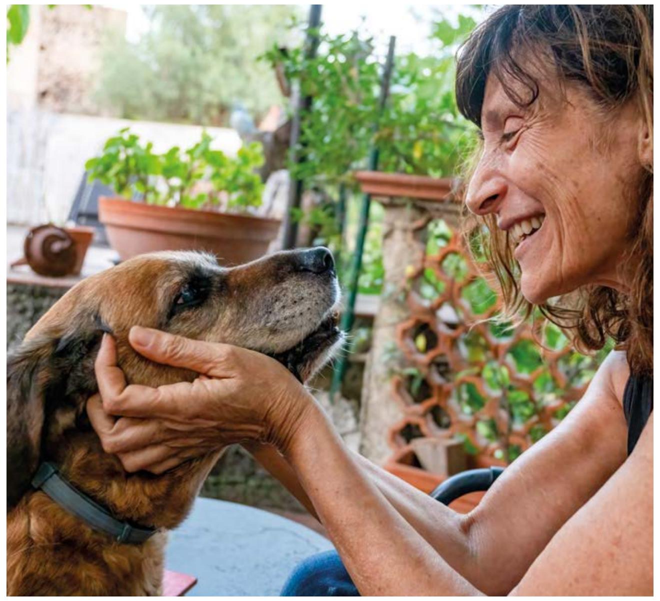

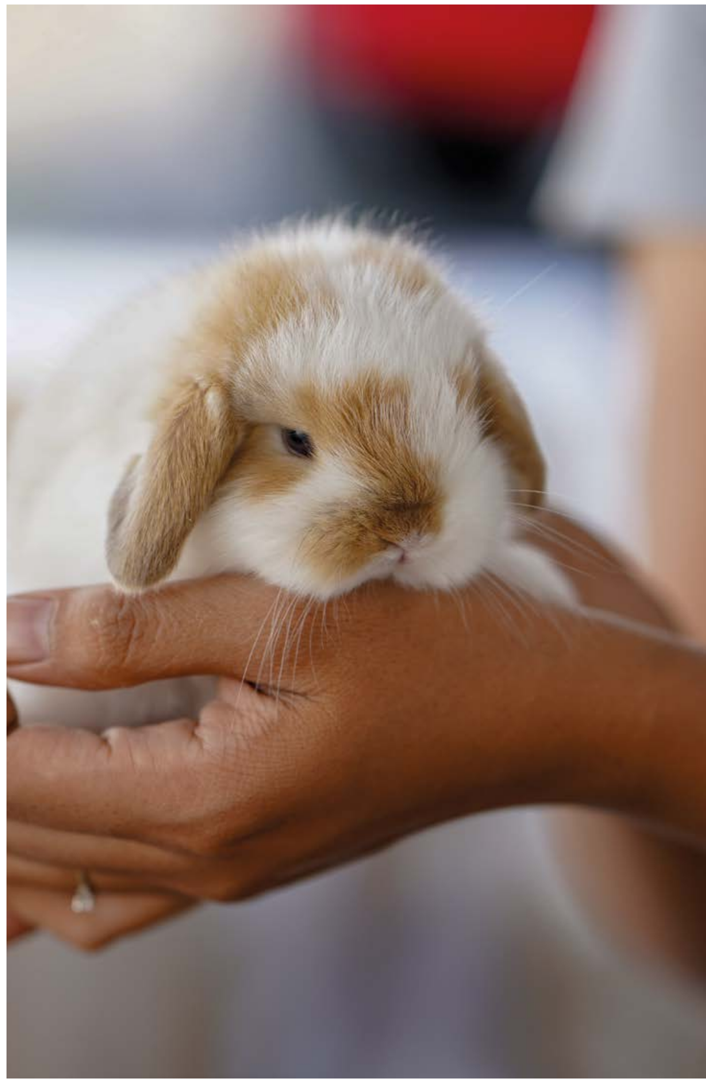

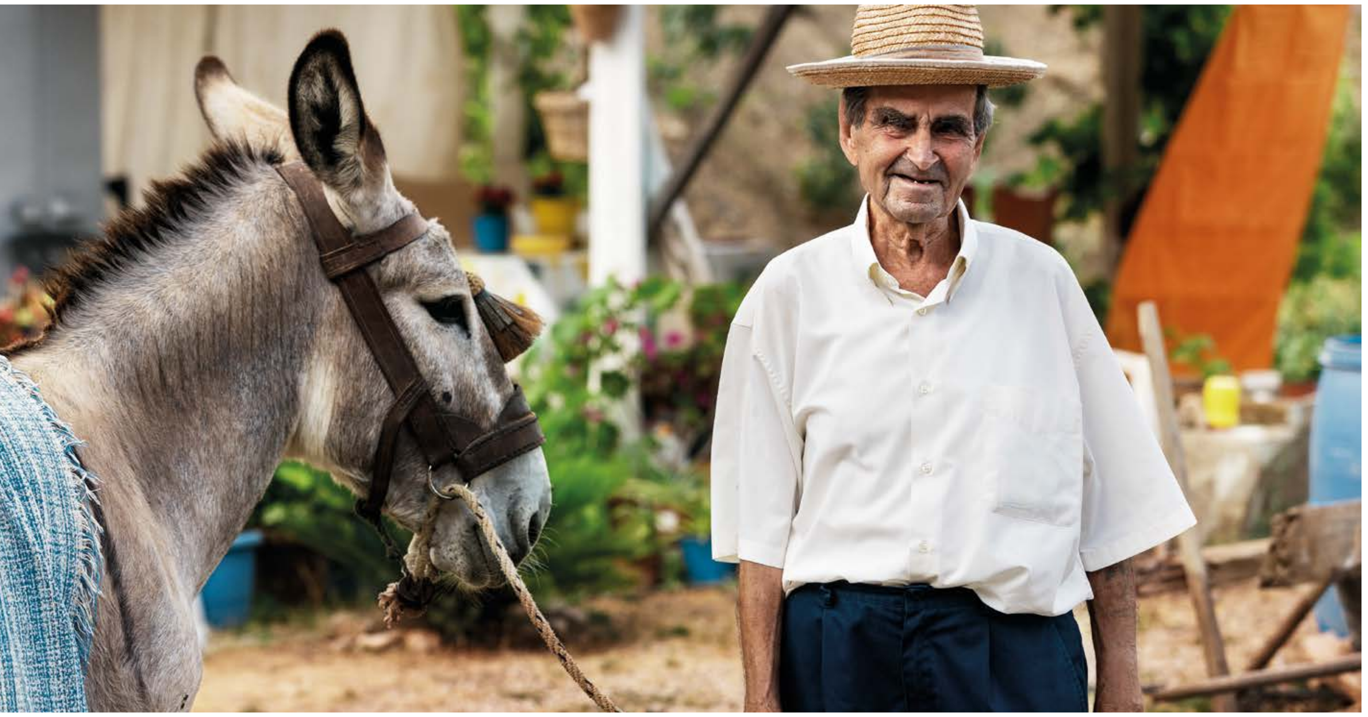

7.2 Critério XI – Reintegração ao Tutor

DEFINIÇÃO	A reintegração de posse aos tutores corresponde a um conjunto de atividades desenvolvidas pela organização auditada com o objetivo de realizar, de forma responsável, a devolução dos animais resgatados e evacuados aos seus tutores originais, após período vivendo em abrigos de fauna sob a tutela da organização auditada.
IMPORTÂNCIA	A reintegração permite que os tutores recebam seus animais saudáveis, de forma a restabelecer as relações de parceria humano e animal do modo mais aproximado de como era antes do resgate ou evacuação. Dessa maneira, o desenvolvimento e o acompanhamento de todas as suas etapas possibilitam a realização do processo de maneira célere e adequada.
ATRIBUIÇÕES DA AUDITORIA TÉCNICA	Auditar o processo de reintegração dos animais aos tutores, desde o correto levantamento e identificação dos responsáveis por cada animal, passando pela comunicação com a comunidade durante a fase de distanciamento de seus animais, até o momento de devolução, respeitando as particularidades de cada caso. O auditor deve conhecer a programação de devolução prevista pela organização auditada, bem como a situação dos animais, relacionada ao estado de saúde e bem-estar e à realidade dos tutores, incluindo as condições da área de destino e de manejo dos animais.

A reintegração é uma fase delicada em todo o processo, visto que essa atividade pode ocasionar impactos negativos tanto para os animais (afetando seu bem-estar e estado de saúde) como para os tutores. Há casos em que o tutor não consegue oferecer condições similares àquelas dispensadas pelos abrigos de fauna avaliados (alimentação especial, profissionais especialistas em comportamento animal), e em que as mudanças em seu modo de vida interferem no vínculo entre o animal e sua família.

INDICADORES

89. A organização auditada possui plano de comunicação diversificado com a finalidade de divulgar animais domésticos resgatados ou evacuados para que seus tutores possam identificá-los e comprovar vínculo.

90. A organização auditada reintegra os animais aos seus tutores originais estando devidamente identificados, vacinados, desparasitados e castrados (quando em anuência com o tutor), seguindo o protocolo sanitário adequado para a espécie.

91. A organização auditada reintegra os animais aos seus tutores originais após a verificação de condições adequadas no local de destino (com a anuência do tutor para essa avaliação).

    Nota: É importante que o tempo de distanciamento entre tutor e animal seja o menor possível, a fim de minimizar impactos psicológicos, fisiológicos e comportamentais.

    Nota: Os tutores devem ser informados da devolução com a maior antecedência possível, de modo que tenham tempo hábil de realizar as adequações necessárias, visando oferecer as melhores condições de manejo possíveis para os animais.

    Nota: A organização auditada deve ser responsável pela emissão da GTA, salvo casos em que seja definido em comum acordo que o tutor ficará responsável.

92. A organização auditada possui logística e transporte adequados para realizar a reintegração dos animais aos seus tutores originais, sem a incidência de cobrança e custos financeiros aos tutores.

93. A organização auditada possui e implementa um programa adequado de acompanhamento dos animais reintegrados aos seus tutores.

    Nota: O prazo de acompanhamento pós-reintegração deverá ser estipulado anteriormente entre as partes interessadas.

    Nota: Importante desenvolver e compartilhar materiais educativos sobre guarda responsável, bem-estar animal e doma gentil de equídeos e bovinos.

7.3 Critério XII – Ações de Adoção e Pós-adoção

DEFINIÇÃO	Consiste em destinar os animais resgatados sem tutor identificado, com o propósito de integrá-los a um núcleo familiar. Consoante ao avanço e à realização da adoção, o acompanhamento de pós-adoção tem a finalidade de verificar a adaptação aos tutores, a adequabilidade do local de moradia, atualizar os protocolos sanitários e orientar sobre a guarda responsável, incluindo necessidades nutricionais, comportamentais e de segurança; e a importância da castração, quando viável.
IMPORTÂNCIA	A adoção é considerada uma das ações fundamentais para o bem-estar animal, já que é uma das principais soluções para evitar abrigos permanentes. A sua realização de forma segura e responsável permite ao animal qualidade de vida junto a seus novos tutores. Dessa maneira, o acompanhamento de pós-adoção é uma ferramenta fundamental que assegura para ambos, tutor e animal, a compreensão de suas individualidades e necessidades.
ATRIBUIÇÕES DA AUDITORIA TÉCNICA	Auditar as ações de adoção e pós-adoção inclui verificar a aptidão dos animais para participar de programas de adoção, em termos de saúde e comportamento, o quantitativo de animais disponíveis para adoção, a eficiência das campanhas de adoção e o acompanhamento do pós-adoção.<br>A equipe de auditores deve procurar conhecer o histórico dos animais, a capacitação da equipe responsável pela realização de entrevistas e visitas aos adotantes, a utilização de Termos de Adoção e de Guarda Responsável com instruções específicas para cada espécie.

Previamente à adoção, qualquer espécie animal deve passar por anamnese e exames laboratoriais que assegurem o seu estado de saúde. É relevante que seja realizada uma análise comportamental do animal, a fim de se estabelecer seu perfil para o direcionamento a um lar compatível às suas necessidades. Para cães e gatos, recomenda-se a esterilização cirúrgica como um pré-requisito.

É importante que os futuros tutores sejam educados quanto à guarda responsável (Teixeira e Ferreira, 2013) e às legislações pertinentes, como o art. nº 32 da Lei de Crimes Ambientais – Lei nº 9.605/1998 e alterada pela Lei nº 14.064/2020 (Brasil, 2020). Nessa normativa, configura como crime a prática de ato de abuso, maus-tratos, ferir ou mutilar animais silvestres, domésticos ou domesticados, nativos ou exóticos, com pena de detenção de três meses a um ano e multa. Para cães e gatos, a Lei nº 14.064/2020 (Brasil, 2020) inclui um capítulo na Lei de Crimes Ambientais (Brasil, 1998) que não só condena como também aumenta a pena para maus tratos contra os animais de companhia.

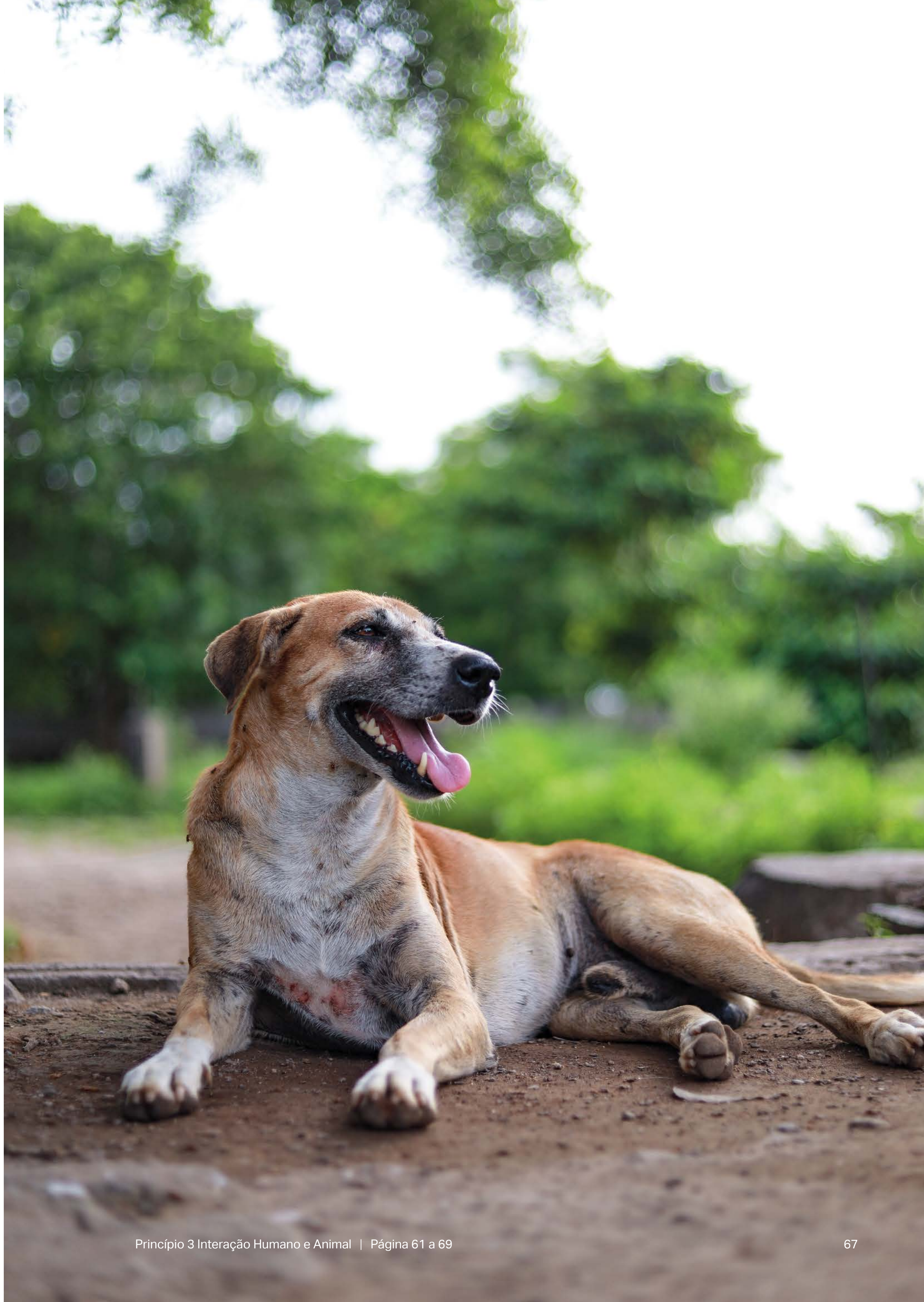

INDICADORES

94. A organização auditada possui programas de adoção, de acordo com a espécie animal disponível e com, métricas de avaliação de eficiência.

Nota: Não deve ser estimulado qualquer programa de adoção que ofereça recompensas, incentivos financeiros ou materiais para os futuros adotantes, pois essas práticas objetificam os animais e não fomentam a adoção responsável.

Nota: A organização auditada deve realizar campanhas de adoção em plataformas virtuais para alcançar um público amplo.

95. A organização auditada assegura a aptidão do animal para adoção, de acordo com as boas práticas vigentes (estar identificado, vacinado, desparasitado, com avaliação de perfil comportamental e castrado, no caso de cães e gatos).

96. A organização auditada apresenta as principais informações individuais (porte, idade, estado de saúde e perfil comportamental) com data da última atualização em seu material de divulgação dos animais disponíveis para adoção.

97. A organização auditada assegura condições de bem-estar e segurança sanitária adequadas para os animais, quando em eventos presenciais de adoção.

98. A organização auditada evidencia a avaliação de aptidão adequada dos futuros tutores, considerando o perfil familiar compatível ao do animal.

Nota: Esse indicador se refere a todos os tipos de animais (companhia ou de produção).

Nota: Deve ser avaliada a regularização da área, atendimento à legislação local de zoneamento urbano e incluir os prazos apropriados para a preparação das propriedades que receberão os animais de fazenda.

Nota: A formação de algumas áreas de capineiras ou pastagens pode requerer meses, dependendo da época do ano, e esse tempo de preparação deve estar incluído no planejamento de entrega do animal ao novo tutor.

99. A organização auditada possui um termo de adoção específico, assinado pelos adotantes, confirmando entendimento sobre a guarda responsável.

Nota: É fundamental incluir, no termo de adoção, cláusulas claras e objetivas relacionadas às legislações vigentes sobre Crimes Ambientais. Além disso, deve-se estabelecer que a adoção do animal não pode ser transferida a terceiros. Caso o tutor desista da adoção, o animal deve retornar à tutela da organização auditada. Também é importante que o futuro tutor se comprometa a fornecer informações sobre as condições do animal por meio de plataformas virtuais, caso a visita de acompanhamento pós-adoção não seja viável.

100. A organização auditada fornece ao novo tutor a documentação atinente ao animal adotado, como seu cartão de vacinação e histórico clínico.

Nota: No caso de adoção especial (animais com condições de saúde crônicas, idosos ou com perfil comportamental desafiador), a organização auditada deve fornecer aos tutores, informações do caso clínico e instruções para uma conduta de cuidados.

101. A organização auditada realiza o transporte dos animais adotados até seu destino e proporciona condições sanitárias e de bem-estar adequadas para cada espécie durante o trajeto.

Nota: Em casos de transportes programados, a organização auditada deve apresentar evidências de que o animal foi transportado com o Guia de Trânsito Animal (GTA) emitido para a data estabelecida.

102. A organização auditada implementa um programa adequado de pós-adoção, com métricas claras de bem-estar, para o acompanhamento do período de adaptação do animal ao novo lar.

Nota: O prazo de acompanhamento pós-adoção será estipulado anteriormente entre as partes interessadas. Porém, não deve ser inferior a 3 meses.

Nota: A organização auditada deve possuir um protocolo de recolhimento do animal caso o seu bem-estar esteja comprometido durante o acompanhamento.

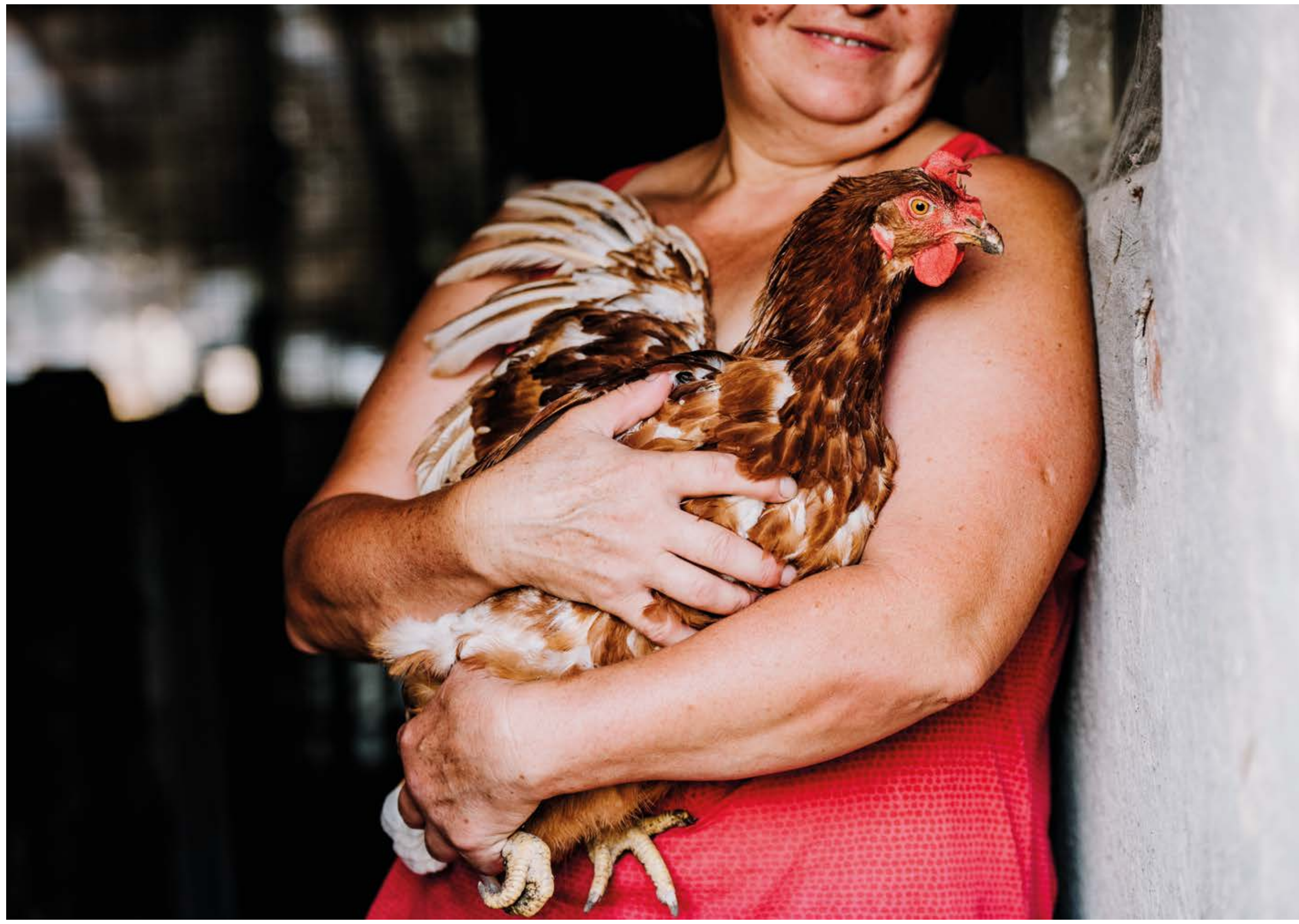

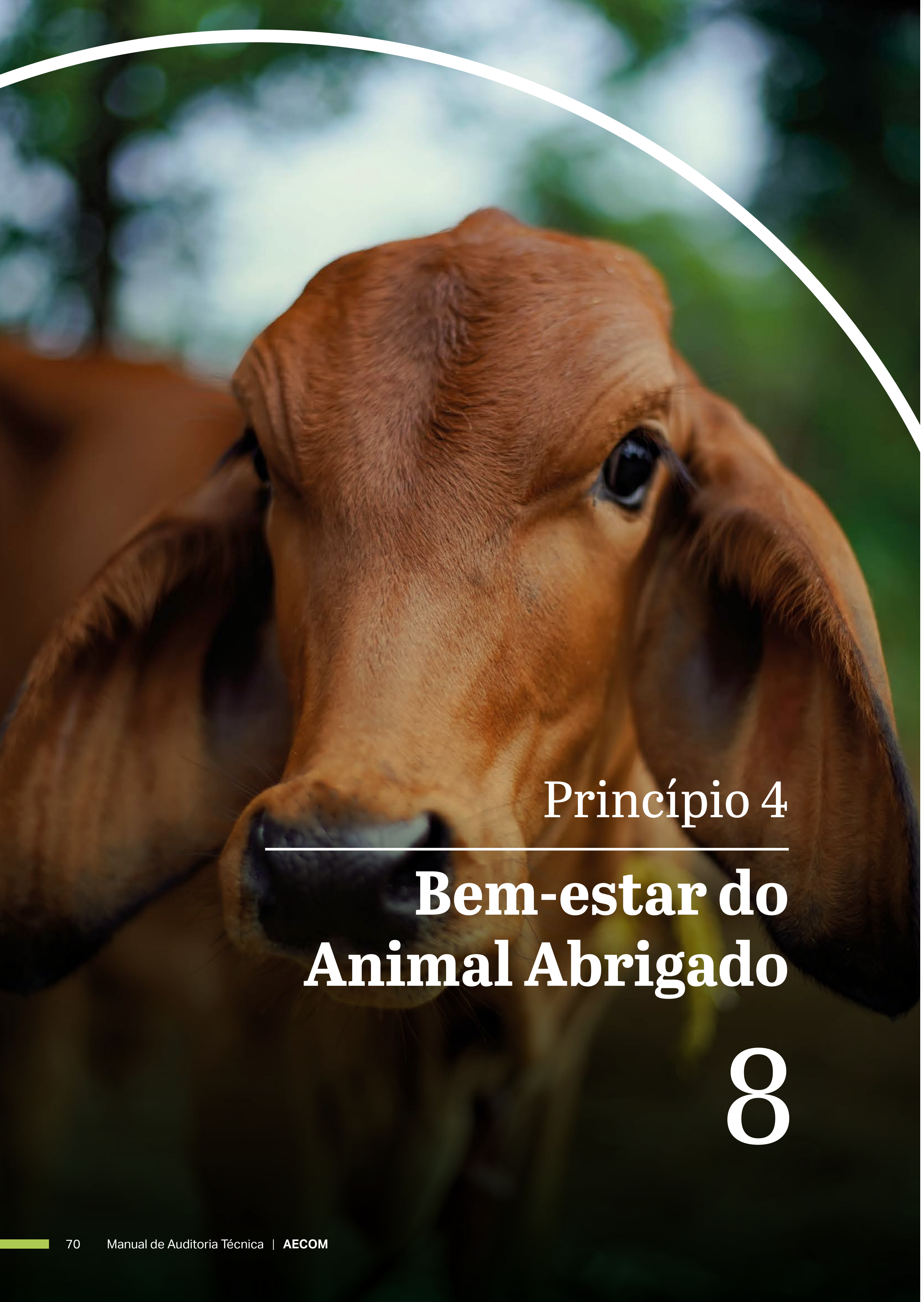

8. Princípio 4 Bem-estar do Animal Abrigado

Esse Princípio considera o bem-estar dos animais sob tutela da organização auditada, trazendo como fundamento os Cinco Domínios, que consideram os estados físicos e funcionais de nutrição, ambiente, saúde e comportamento que, por sua vez, afetam positiva ou negativamente o estado mental de cada indivíduo.

Os abrigos de fauna devem ser considerados como moradias temporárias onde animais em situação de risco em desastres (evacuados ou resgatados) são acolhidos, medicados e mantidos em boas condições de bem-estar até serem adotados ou retornarem aos seus tutores originais (Galdioli *et al.*, 2021; Galdioli e Garcia, 2022). Embora a grande maioria dos abrigos sejam voltados para cães e gatos, existem aqueles direcionados para equídeos, ruminantes, aves domésticas, peixes e animais silvestres, que devem atuar de acordo com as legislações vigentes.

Quando abrigos temporários estruturados para situações de emergência se tornam permanentes, há um impacto negativo crescente no bem-estar animal decorrente das altas taxas de ocupação, risco aumentado de transmissão de doenças infectocontagiosas e problemas comportamentais causados por ambientes com baixa complexidade estrutural e poucas atividades recreativas (Gebara *et al.*, 2024).

O controle de doenças infecciosas e zoonóticas é um grande problema em abrigos de animais por diversos fatores: animais recém-introduzidos podem carregar uma variedade de patógenos, animais estressados são mais vulneráveis à infecção, a superlotação predispõe a piores condições de higienização, dificulta a vistoria diária dos animais e promove a propagação da infecção. Enquanto algumas doenças infecciosas possuem protocolos bem estabelecidos para controle nesses locais, como a cinomose e a doença do aparelho respiratório felino, o risco de surto de outros patógenos é pouco conhecido, como leishmaniose (*Leishmania* spp.), leptospirose (*Leptospira* spp.) ou microrganismos resistentes a antibióticos (Foley e Bannasch, 2004; Bannasch e Foley, 2005; Aguiar *et al.*, 2017).

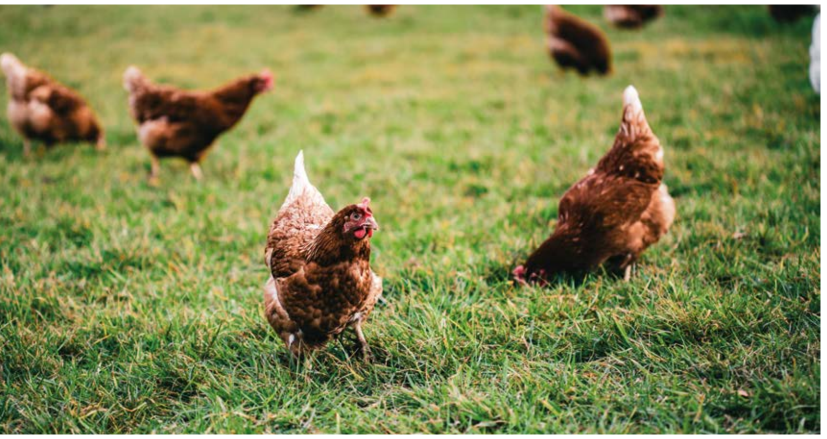

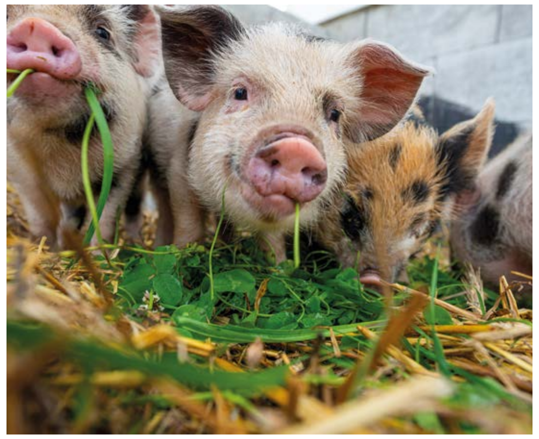

Ao se analisar o bem-estar como um todo, tão importante quanto as informações obtidas diretamente do animal (estado de saúde, comportamento natural da espécie, estado mental ou afetivo) é a avaliação da adequação do ambiente para cada espécie abrigada e o gerenciamento da estrutura física e humana envolvida, que precisa ser proativa e focada na prevenção (Figura 12). A métrica da Capacidade de Prover Cuidados (CPC) deve ser aplicada à toda instituição para reconhecer seu limite de atendimento, pois permite que os animais tenham qualidade de vida adequada enquanto abrigados (Rocha e Galdioli, 2022).

Capacidade de Prover Cuidados (CPC) em abrigos de fauna

RECURSOS DISPONÍVEIS

- Taxa de ocupação;

QUALIDADE DE VIDA DOS ANIMAIS

- Funcionários em quantidade suficiente;
- Recursos financeiros;
- Cinco domínios do bem-estar.

PROGRAMAS PREVENTIVOS

- Registro e identificação;
- Banco de dados;
- Estruturas físicas;
- Mapa de risco por setor;
- Limpeza e higienização;
- Imunização;
- Enriquecimento ambiental;
- Protocolos de tratamento;
- Tempo de permanência no abrigo;
- Ações de destinação.

Figura 12 – Capacidade de Prover Cuidados em abrigos de fauna (adaptado de Rocha e Galdioli, 2022).

No contexto do processo de auditoria técnica voltada à proteção da fauna para o Princípio Bem-estar do Animal Abrigado, são elencados cinco Critérios avaliativos, conforme a Figura 13.

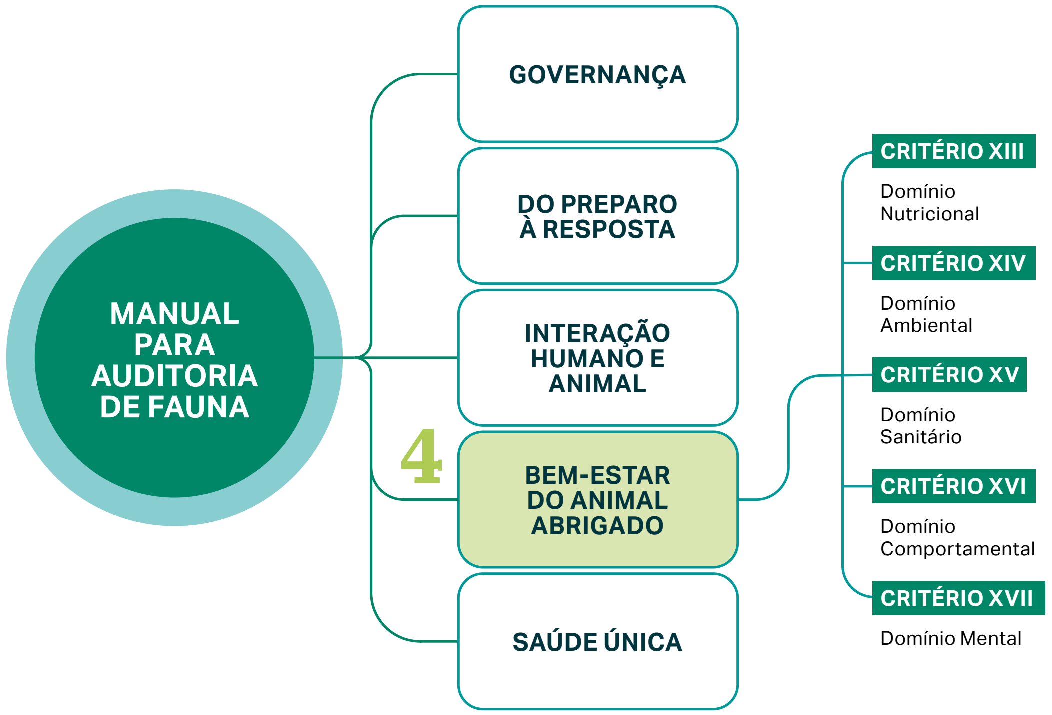

Figura 13 – Critérios avaliativos do Princípio Bem-estar do Animal Abrigado.

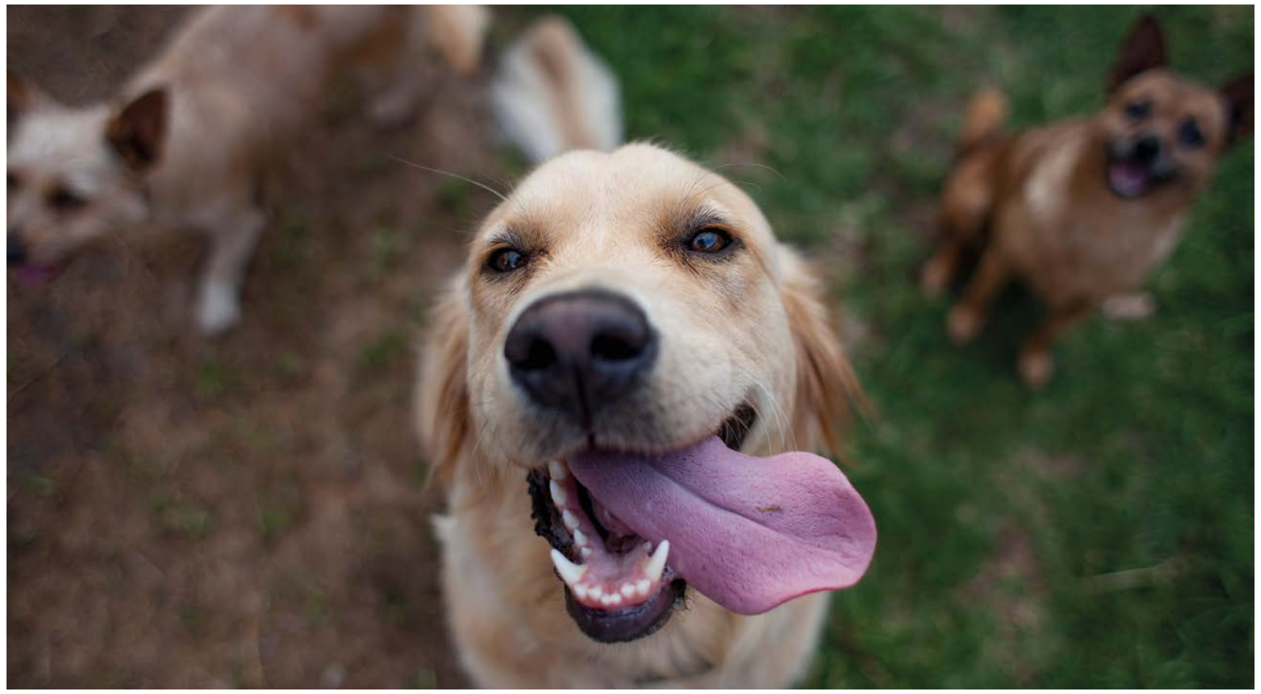

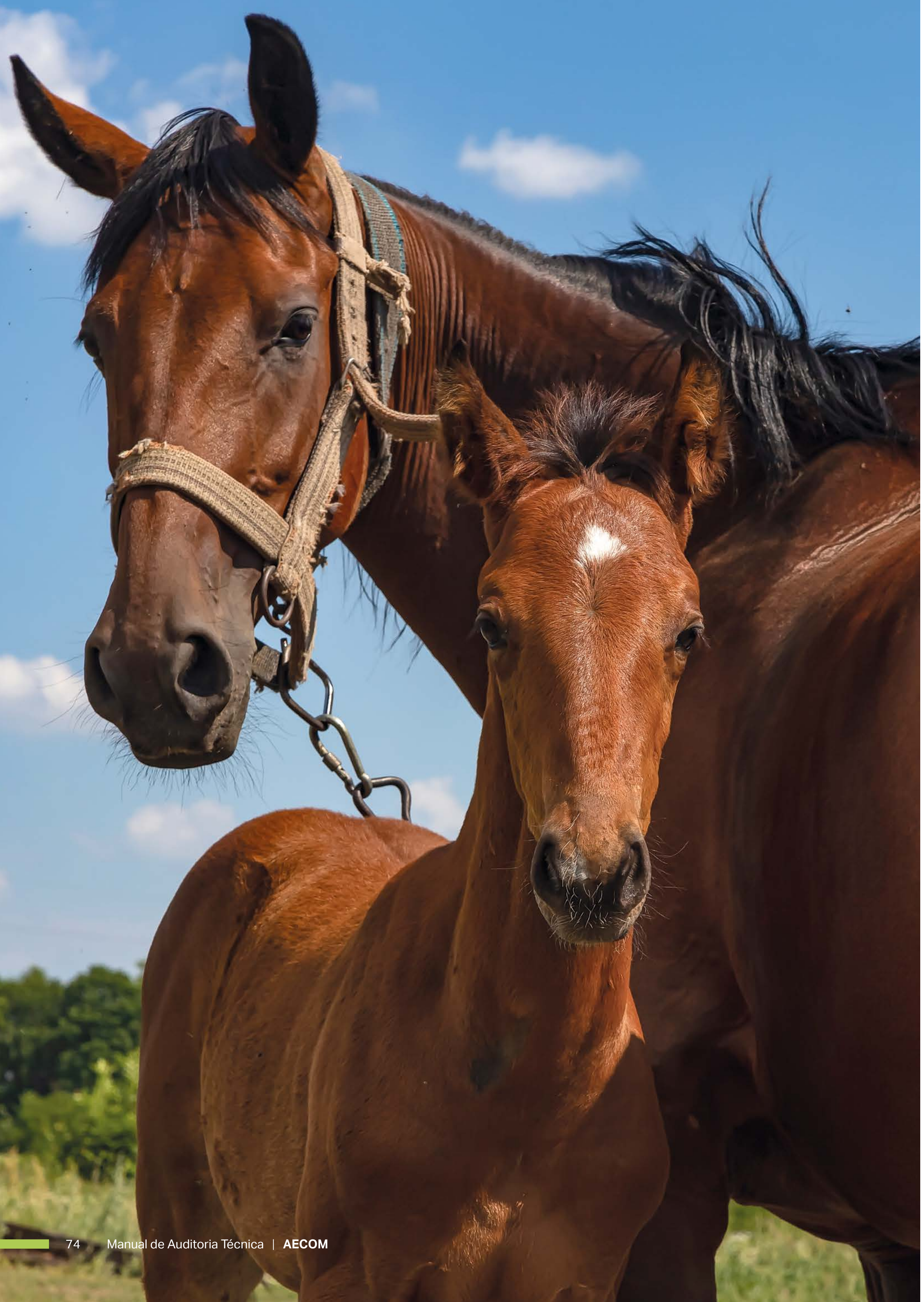

8.1 Critério XIII – Domínio Nutricional

DEFINIÇÃO	Considera que cada animal deve ter acesso a comida e a água em quantidade, frequência e qualidade ideais para consumo. Nesse contexto, a necessidade nutricional é individualizada e tende a mudar conforme espécie, idade, tamanho e condições fisiológicas.
IMPORTÂNCIA	Para o respeito às exigências nutricionais de cada espécie é fundamental que o animal tenha acesso a um plano alimentar balanceado. A alimentação adequada permite que o animal se desenvolva normalmente e de maneira saudável. Por outro lado, uma alimentação inadequada leva ao desequilíbrio físico e mental. Destaca-se também a importância do uso de alimentos de forma criativa como ferramentas de enriquecimento múltiplo em ações de bem-estar para todas as espécies.
ATRIBUIÇÕES DA AUDITORIA TÉCNICA	A auditoria técnica é responsável por avaliar o grau de conformidade da dieta fornecida considerando as peculiaridades de cada indivíduo.<br>Deve ainda verificar se há um controle interno de acompanhamento individual para confirmar a alimentação adequada de cada animal.

INDICADORES

103. A organização auditada fornece alimento de boa qualidade, em quantidade compatível à necessidade de cada indivíduo, disponibilizada em comedouros acessíveis e em quantidade proporcional ao número de animais no recinto e condizente com a espécie, idade e estado clínico de cada animal abrigado.

104. A organização auditada fornece água de boa qualidade, em temperatura adequada e quantidade suficiente, fornecida em bebedouros acessíveis e em quantidade proporcional ao número de animais abrigados, de forma condizente a cada espécie.

105. A organização auditada realiza observações diárias sobre o comportamento alimentar de cada indivíduo, sendo capaz de constatar mudanças no padrão de alimentação dos animais em recintos individuais ou coletivos.

106. A organização auditada realiza avaliação periódica do escore de condição corporal e intervém em casos de animais com ganho ou perda excessiva de peso corporal.

107. A organização auditada realiza o monitoramento dos efeitos e eficácia das rações medicamentosas quando necessárias.

8.2 Critério XIV – Domínio Ambiental

DEFINIÇÃO	É provido quando o local de abrigo do animal permite que ele possa viver livre de desconforto em um ambiente com dimensões, infraestrutura e conforto sensorial compatíveis com as necessidades de cada espécie.
IMPORTÂNCIA	A qualidade de vida do animal abrigado é diretamente influenciada pelas características de seu recinto e dos fatores ambientais do entorno. O local deve proporcionar condições de abrigo e descanso adequados, com conforto térmico, possibilidade de movimentação fácil e segura, para abrandar o estresse de confinamento e manter a saúde física e mental.
ATRIBUIÇÕES DA AUDITORIA TÉCNICA	Auditar uma instalação de fauna, que servirá como destinação de animais resgatados, consiste em verificar o planejamento e a gestão implementada para o recebimento desses animais. Dessa maneira, é importante que a taxa de ocupação, procedimentos operacionais, limpeza e organização da instalação estejam sistematizadas, que a infraestrutura dos abrigos seja adequada para cada espécie, incluindo as áreas de manejo, e que a equipe seja qualificada e em número suficiente para recebimento e manejo dos animais.

Os indicadores desse Critério devem ser ajustados de acordo com as normas e leis já existentes para cada espécie.

INDICADORES

108. A organização auditada possui recintos com espaço suficiente para o animal ficar em pé, explorar, urinar e defecar longe da comida, descansar em posição confortável, com substrato adequado para a espécie, tomar sol e se proteger da chuva, do calor e do frio.

Nota: O uso de correntes ou assemelhados para contenção do animal no recinto caracteriza maus-tratos e só deve ser utilizado temporariamente, em situações emergenciais.

Nota: Dentre as características associadas às necessidades de cada espécie, destacam-se solários e áreas de recreação. Área interna com espaços adequados (refúgio, ambientes aquáticos, poleiros) e protegidos contra condições climáticas adversas, incluindo proteção a ruídos externos.

109. A organização auditada possui recintos construídos com materiais não porosos de fácil higienização e que não apresentam arestas afiadas, desgastes no piso, infiltrações e outras imperfeições que podem causar injúrias aos animais abrigados.

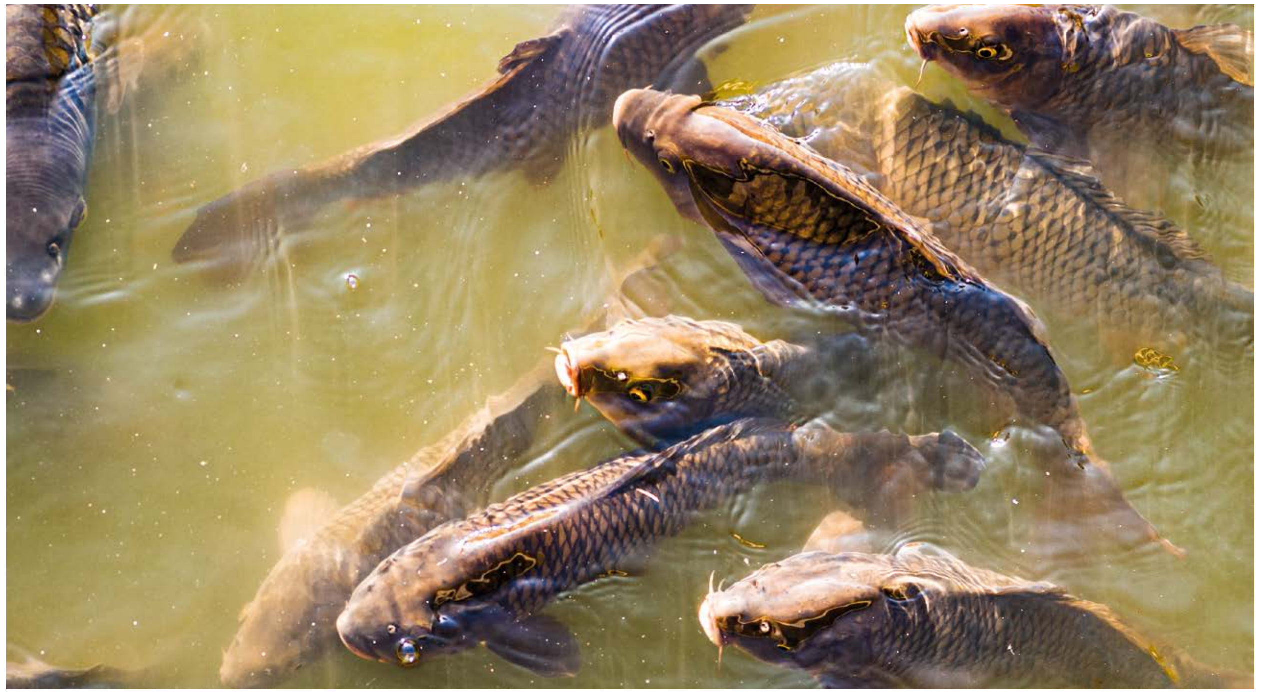

110. A organização auditada possui recintos com refúgios e estruturas como camas, caixas, ninhos e plataformas, permitindo aos animais se protegerem de situações negativas ou causadoras de estresse.

111. A organização auditada possui recintos adequados para animais com necessidades especiais, em recuperação clínica ou com restrições de convívio.

112. A organização auditada possui dispositivos de segurança para impossibilitar a fuga dos animais durante as ações de manejo.

113. A organização auditada que abriga espécies aquáticas realiza avaliação periódica adequada dos parâmetros de qualidade da água e os mantém dentro dos limites estabelecidos para o bem-estar dos animais.

Nota: Em caso de alteração nos valores limites dos parâmetros, as medidas de controle devem ser implementadas.

114. A organização auditada possui mecanismos de segurança para proteção dos animais abrigados, para que não sejam furtados e/ou de qualquer forma molestados por terceiros.

115. A organização auditada possui procedimentos voltados para preparação das instalações em função do período do ano (chuvoso ou seca).

116. A organização auditada fornece abrigos para a fauna silvestre com infraestrutura adequada ao comportamento natural de cada espécie.

Nota: Em caso de necessidade de criação de Centro de Triagem da Fauna Silvestre (CETAS), deve-se observar as diretrizes propostas nos art. 14º e 15º da Instrução Normativa IBAMA nº 146/2007 que detalham as características básicas de instalações dessa natureza, além de orientações do órgão responsável.

8.3 Critério XV – Domínio Sanitário

DEFINIÇÃO	É provido através de medidas de prevenção como triagem no momento da entrada, aplicação de vacinas e de antiparasitários, monitoramento clínico periódico para cada animal e populacional, tratamento de doenças, feridas e dores.
IMPORTÂNCIA	O domínio sanitário possui relevância do ponto de vista social e econômico, pois engloba prevenção de doenças infecciosas e zoonoses. Em abrigos de fauna, as ações desenvolvidas para controle sanitário têm a finalidade de minimizar os riscos de contágio de doenças e até mesmo sua erradicação, além de assegurar tratamento adequado para que os animais permaneçam com saúde e bem-estar.
ATRIBUIÇÕES DA AUDITORIA TÉCNICA	A auditoria técnica é responsável por avaliar as ações direcionadas à saúde dos animais abrigados por meio de evidências fotográficas, documentos (como laudos clínicos e laboratoriais) e durante as auditorias técnicas de campo. É importante que sejam consideradas as peculiaridades de cada indivíduo e espécie. Também cabe ao auditor verificar a existência de protocolos adequados de higienização de recintos e utensílios, controle interno de vacinação e de antiparasitários, além de demais cuidados necessários a cada espécie.

INDICADORES

117. A organização auditada realiza triagem clínica e epidemiológica prévia para os animais destinados aos abrigos de fauna.

118. A organização auditada possui e implementa protocolo de imunização e controle de parasitas dos animais abrigados.

> Nota: Considerar o mesmo protocolo para os animais que permanecem com o tutor e que recebem atendimento externo.
>
> Nota: Considerar protocolos alternativos para animais com sensibilidade aos compostos ativos dos medicamentos antiparasitários, especialmente para coleira repelente.

119. A organização auditada mantém um cronograma para avaliação clínica periódica dos animais e realiza exames laboratoriais quando necessários.

120. A organização auditada demonstra que utiliza procedimentos que asseguram confiabilidade das análises laboratoriais dos animais sob a sua tutela (coleta, armazenamento, transporte e processamento de amostras biológicas).

121. A organização auditada implementa mapeamento de risco ambiental e protocolos de limpeza e higienização adequados a cada setor de seu abrigo.

> Nota: É recomendável classificar áreas do abrigo em crítica, semicrítica e não crítica, de acordo com o grau de risco de biossegurança e fazer a separação de animais em tratamento para doenças infecciosas, animais recém-admitidos, filhotes e/ou fêmeas prenhes em recintos adequados de acordo com esta classificação.

122. A organização auditada assegura que os produtos violados e/ou vencidos são segregados em ambiente seguro e identificados quanto a sua condição e destino.

123. A organização auditada possui manejo individualizado preventivo para os animais com condições que aumentam sua susceptibilidade a doenças infectocontagiosas (gestantes, filhotes, geriátricos ou com doenças crônicas).

124. A organização auditada provê tratamentos adequados para os animais diagnosticados com doenças de potencial zoonótico, assim como implementa medidas preventivas para diminuir o risco de transmissão para pessoas e outros animais.

125. A organização auditada realiza procedimentos cirúrgicos de esterilização somente em animais aptos e mediante autorização do tutor, quando identificado.

126. A organização auditada certifica que todos os fômites que entram em contato com animais enfermos sejam desinfetados ou descartados após o uso.

127. A organização auditada possui procedimentos voltados para organização e limpeza das áreas comuns dentro dos abrigos de fauna.

128. A organização auditada apresenta um protocolo de avaliação de sanidade dos peixes abrigados, realizado de forma periódica para diagnosticar as principais doenças atinentes às espécies abrigadas.

> Nota: Boas práticas de coleta e armazenamento de amostras biológicas de fauna aquática podem ser consultadas em publicações técnicas do Ministério da Pesca e Aquicultura (RENAQUA, 2013).

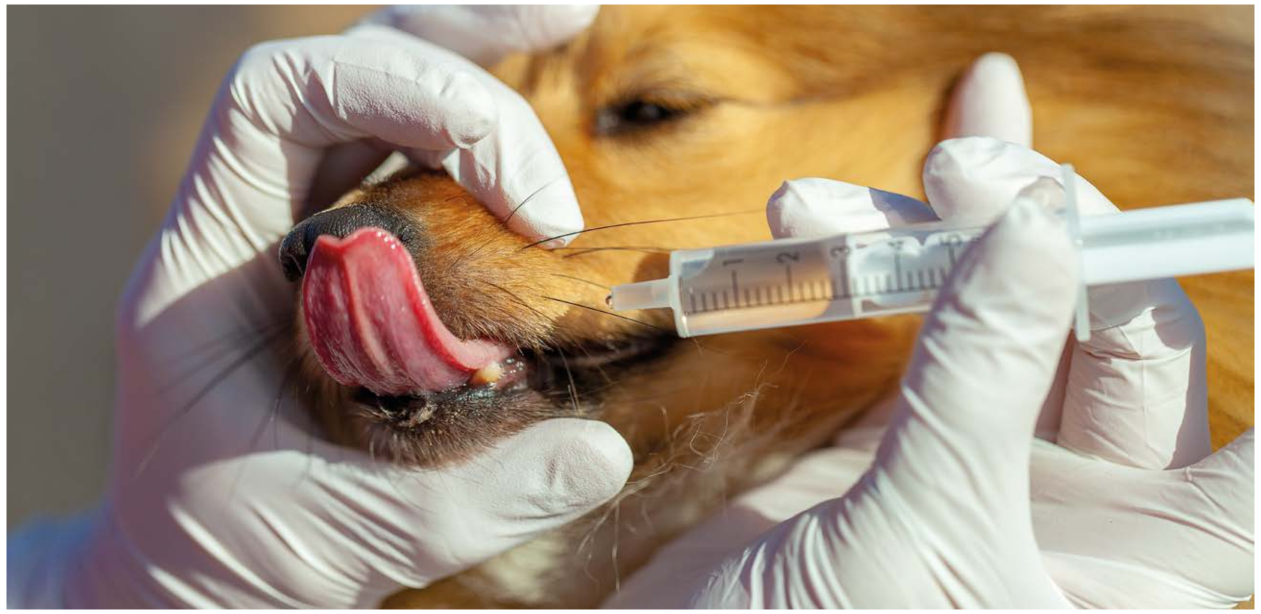

8.4 Critério XVI – Domínio Comportamental

DEFINIÇÃO	Considera que o animal abrigado deve exercer o seu comportamento natural. Por sua vez, o bem-estar comportamental está diretamente relacionado ao tempo de confinamento e à rotina do abrigo.
IMPORTÂNCIA	Cada espécie animal é dotada do seu próprio repertório de padrões de comportamento. Nesse sentido, o domínio comportamental reflete a necessidade de cada indivíduo ao seu comportamento natural, de acordo com suas características anatômicas de sua espécie. Os animais devem ter a liberdade de escolha, o que exige espaço suficiente, instalações adequadas e a companhia coespecífica, para aquelas espécies que são classificadas como sociais. Quando o animal não consegue manifestar seu comportamento em cativeiro, pode desenvolver estados mentais e emocionais negativos que levam a alterações etológicas como agressividade, apatia e estereotipias.
ATRIBUIÇÕES DA AUDITORIA TÉCNICA	A auditoria técnica deve verificar se as ações de enriquecimento ambiental implementadas estão de acordo com as necessidades de modulação comportamental de cada espécie abrigada e considerar as peculiaridades de cada indivíduo em um determinado grupo (Galdioli *et al.*, 2021). Deve ainda verificar se há um controle interno do histórico de monitoramento comportamental dos animais abrigados.

INDICADORES

129. A organização auditada promove a formação de grupo por características sociais compatíveis intra e interespécies em suas instalações destinadas à fauna.

130. A organização auditada realiza a avaliação comportamental individual dos animais e, em caso de desvios, determina ações para melhoria do bem-estar do animal.

> Nota: Deve-se avaliar se cada protocolo apresenta procedimentos padronizados, com metodologias preconizadas e aprovadas pelos conselhos de classe e organizações oficiais, possibilitando diagnosticar, monitorar e avaliar os resultados do manejo comportamental, de acordo com cada espécie.

131. A organização auditada realiza monitoramento comportamental periódico, tanto dos indivíduos como dos grupos formados.

132. A organização auditada aplica técnicas de enriquecimento multimodais espécie-específicas e direcionadas, conforme avaliação comportamental.

133. A organização auditada implementa ações de modulação comportamental com metodologias adequadas que consideram as características intrínsecas de aprendizado para cada espécie e indivíduo.

8.5 Critério XVII – Domínio Mental

DEFINIÇÃO	Este domínio está relacionado ao bem-estar físico e emocional do animal, sendo exteriorizado comportamentalmente através de suas capacidades cognitivas e na sua consciência, levando o animal a buscar ativamente estímulos para vivenciar emoções positivas.
IMPORTÂNCIA	As interações ambientais e sociais para solução de problemas, aprendizagem, expectativas, intenções e tomadas de decisões irão refletir na saúde mental do animal. O manejo do abrigo de fauna deve prevenir que o animal seja exposto a situações que causem sofrimento e sentimentos negativos, como frustração, tédio, medo, dor, estresse e, de forma complementar, deve oferecer opções para os animais vivenciarem situações positivas com a inclusão de programas que estimulam estados emocionais positivos (recreação, treinamentos por recompensa, dessensibilização, dentre outros).
ATRIBUIÇÕES DA AUDITORIA TÉCNICA	A auditoria técnica é responsável por avaliar se a organização auditada realiza atividades e possui equipe técnica especializada para a manutenção da saúde mental dos animais abrigados considerando as peculiaridades de cada indivíduo e a expressão de seu comportamento natural.

INDICADORES

134. A organização auditada considera as características comportamentais naturais das espécies (fossoriais, diurnas, noturnas, crepusculares) para o manejo dos animais nas instalações.

135. A organização auditada considera o comportamento natural das espécies (social ou solitária) e a relação antagonista entre predador e presa no planejamento de ocupação dos recintos.

136. A organização auditada inclui nos protocolos de manejo da fauna silvestre, atividades que reproduzam e estimulem os comportamentos naturais das espécies abrigadas.

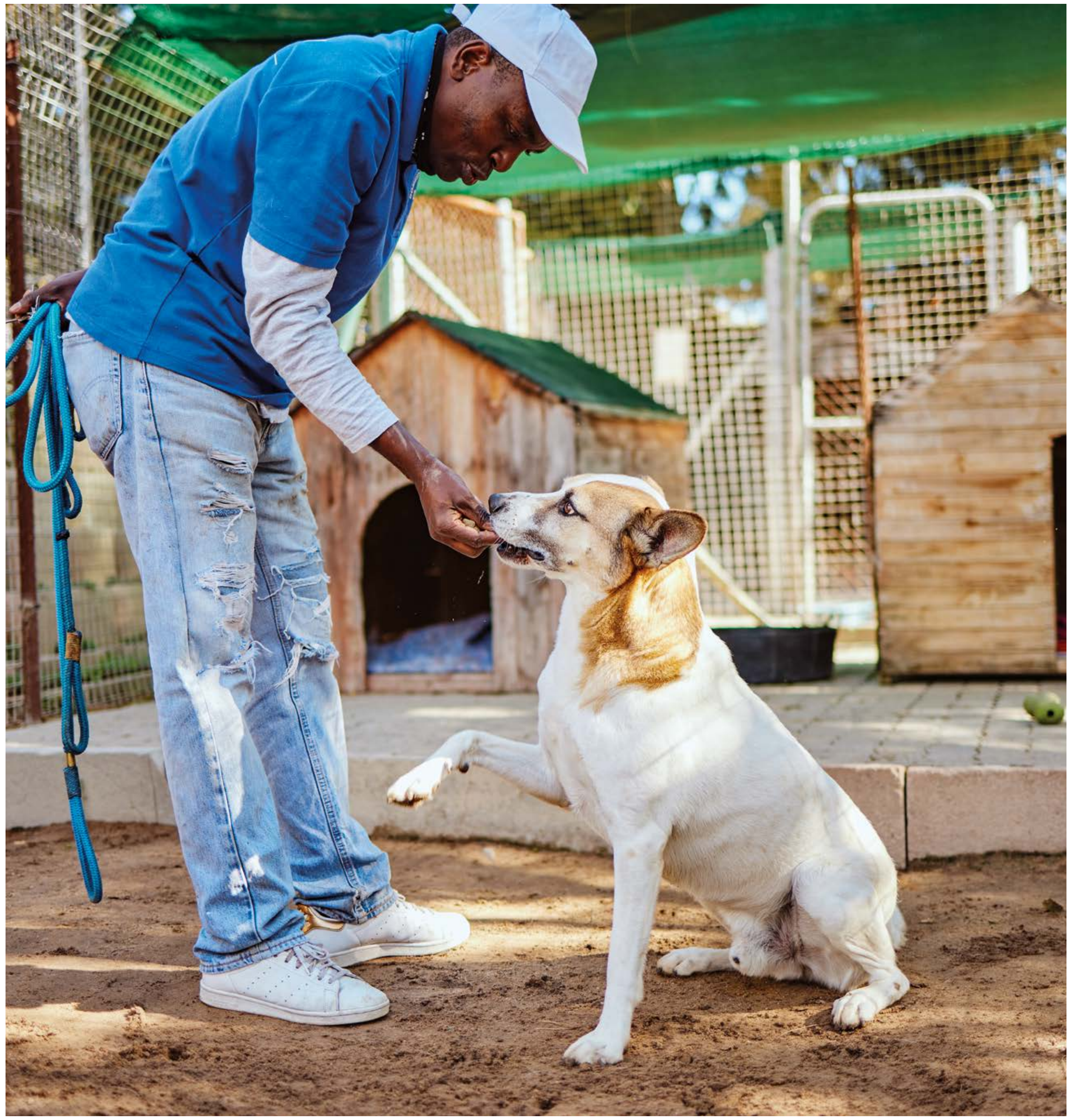

137. A organização auditada considera as características pretéritas de vida do animal (antes da evacuação ou resgate) como elementos essenciais para o processo de manejo comportamental.

138. A organização auditada adota procedimentos adequados de eutanásia de acordo com cada espécie.

> Nota: Em certos casos, ações de eutanásia de animais abrigados podem requerer anuência de órgãos públicos de defesa da fauna e não apenas da avaliação individualizada da equipe médica-veterinária responsável.
>
> Nota: Devem ser respeitados os requisitos básicos e normativas estabelecidas para o procedimento da eutanásia, que consistem em sedação, analgesia e relaxamento muscular.

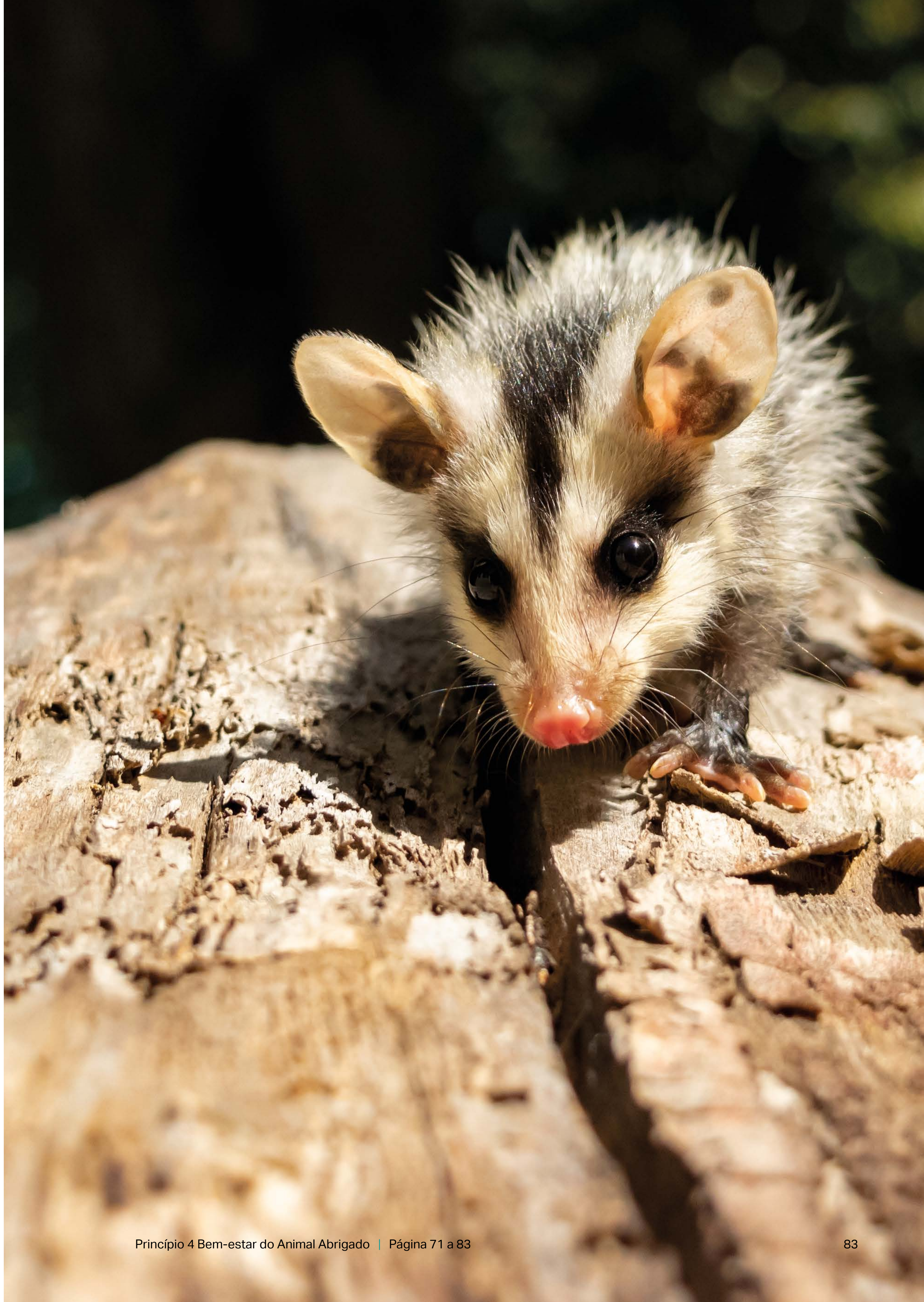

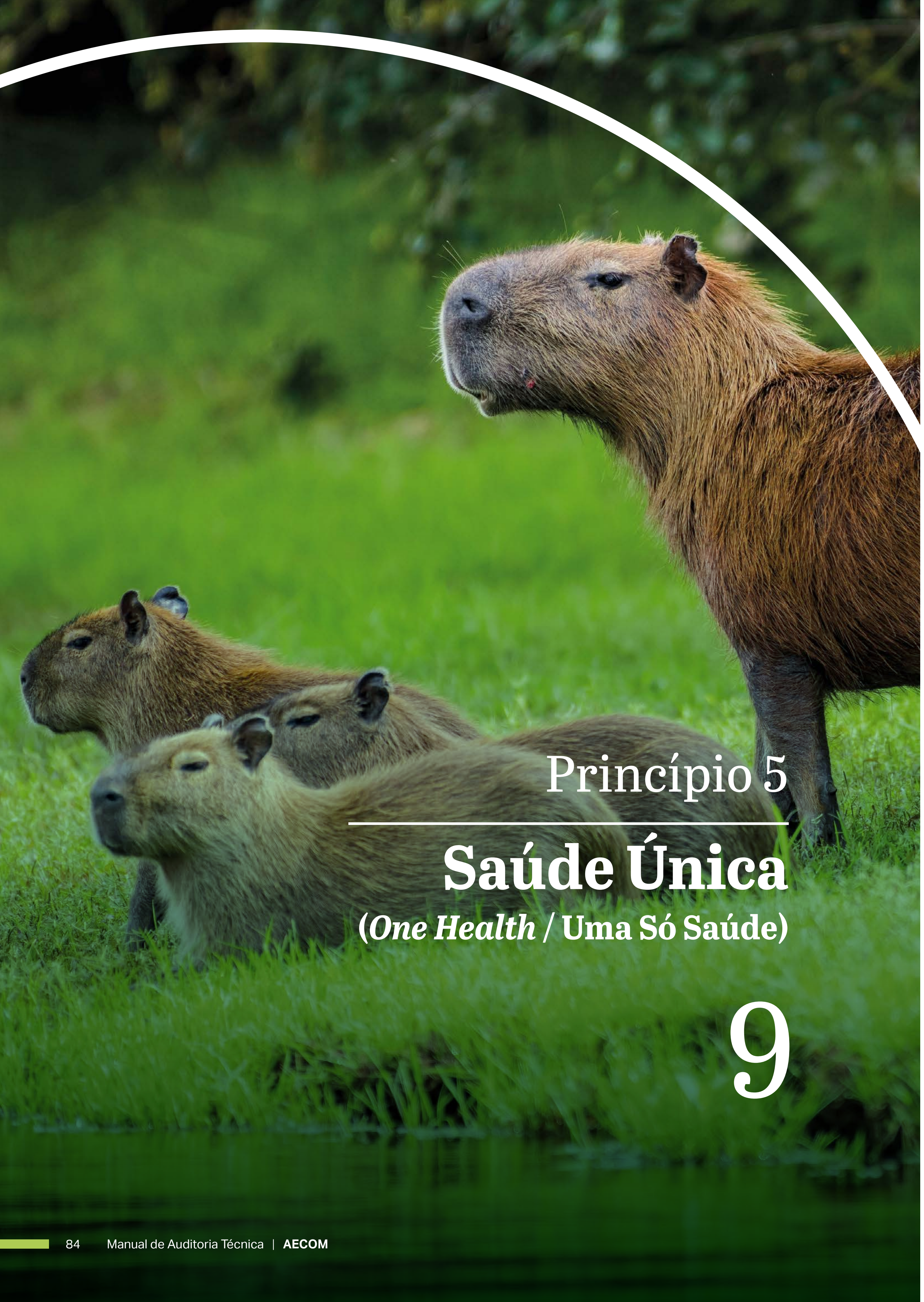

9. Princípio 5 Saúde Única (*One Health* / Uma Só Saúde)

A abordagem de Saúde Única, termo originário do inglês "*One Health*", também conhecida como "Uma Só Saúde", é uma proposta cientificamente estabelecida e validada, de grande importância social. Essa abordagem surgiu, inicialmente, do estudo integrado de zoonoses e das doenças emergentes. Atualmente, é definida como "*uma abordagem integrada e unificadora que visa equilibrar e otimizar de forma sustentável a saúde das pessoas, animais e ecossistemas. Reconhece a saúde dos seres humanos, dos animais domésticos e selvagens, das plantas e do ambiente mais amplo (incluindo ecossistemas) estão estreitamente ligados e interdependentes*" (OHHLEP *et al.*, 2022).

Sua aplicação estimula a mobilização de programas com governança intersetorial e interdisciplinar em vários níveis da sociedade para trabalharem juntos para promover o bem-estar e enfrentar ameaças à saúde e aos ecossistemas, ao mesmo tempo em que aborda a necessidade coletiva de acesso à água e a ar limpos, energias de baixo impacto, alimentos seguros e nutritivos e adaptação às mudanças climáticas, contribuindo para o desenvolvimento sustentável (Carneiro e Pettan-Brewer, 2021). O objetivo é a união de informações e decisões que costumam ser implementadas de forma segregada por limitações de gestão, métodos, financiamento ou comunicação.

No Brasil, país que se destaca mundialmente na aplicação e fortalecimento da abordagem Saúde Única, foi promulgado oficialmente o Dia Nacional da Saúde Única, que internacionalmente é comemorado em 3 de novembro a Lei nº 14.792/2024 (Brasil, 2024). Além disso, foi estabelecida, através do Decreto nº 12.007/2024 (Brasil, 2024), a criação do Comitê Uma Só Saúde, coordenado pelo Ministério da Saúde e composto por 20 entidades, que tem como objetivo central promover uma abordagem integrada e multisetorial entre diferentes setores da sociedade para prevenir e controlar ameaças à saúde.

Na prática do manejo de fauna em situação de desastres, os preceitos da saúde única são aplicados na avaliação combinada de riscos à saúde na interface ecológica (humano, animal, plantas e do ambiente físico) com a concepção e implementação de estratégias de intervenção e prevenção com o objetivo de produzir conhecimento integrado (WOAH, 2021). Essas avaliações são de natureza transdisciplinar, incluindo, mas não se limitando a epidemiologia, a medicina preventiva, à toxicologia, à ecologia, à agricultura, à sustentabilidade, à medicina da conservação, ao urbanismo, à economia, à comunicação, à psicologia, à antropologia, às ciências sociais, entre outros (Carneiro e Pettan-Brewer, 2021). Inclui-se também a prevenção e ao monitoramento de zoonoses que podem surgir em cenários pós-desastres, em que há um grande impacto nas

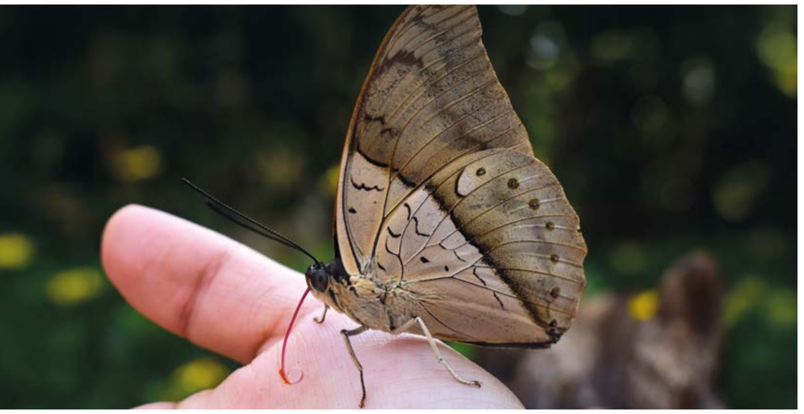

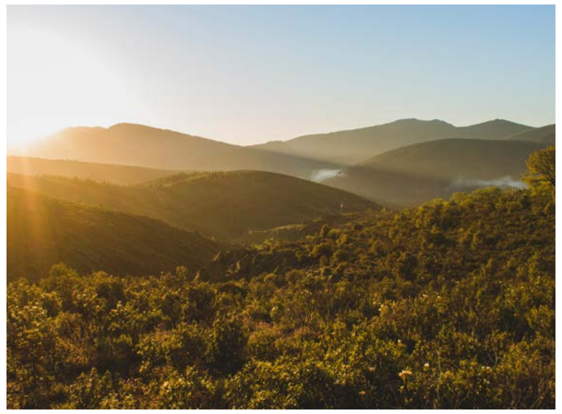

interações ecológicas, facilitando desequilíbrios ambientais e surgimento de epidemias. Também é importante a avaliação de riscos em saúde única nos ambientes onde há muitos animais domésticos confinados, como abrigos, fazendas, granjas e moradias.

Além desses, a resistência antimicrobiana (AMR, da sigla em inglês para *Antimicrobial Resistance*) é considerada um dos desafios aos sistemas de saúde contemporâneos. A OMS define AMR como a "*capacidade de um microrganismo impedir a atuação de um antimicrobiano*". Como resultado, os tratamentos de doenças virais, parasitárias ou fúngicas tornam-se ineficazes e as infecções persistentes ou até incuráveis. As bactérias resistentes aos antimicrobianos podem circular entre seres humanos e animais por meio da alimentação, da água e do meio ambiente, e sua transmissão é influenciada pelo comércio, pelas viagens e pelas migrações humana e animal. A abordagem de saúde única que se viu refletida na concepção do Plano de Ação Global estabelece a necessidade desse envolvimento multisetorial com o objetivo de assegurar o tratamento da questão sob as perspectivas conjugadas de saúde humana, animal e ambiental (WHO, 2019). Considerado um problema global para os sistemas de saúde, a resistência aos antimicrobianos mobiliza diversos organismos internacionais de vigilância à saúde, indústrias alimentícias, cientistas e governos. Em relação à ameaça AMR no Brasil, além do Ministério da Saúde, outros atores governamentais também estão envolvidos em seu enfrentamento e atuam na temática, como a ANVISA, a Fundação Oswaldo Cruz (FIOCRUZ), o Ministério da Agricultura e Pecuária (MAPA, 2020; 2022) e o Ministério da Ciência, Tecnologia, Inovações e Comunicações (MCTIC) (Portaria de Consolidação nº 5/2017).

Apesar de utilizar doenças infecciosas e resistência microbiana como ponto de partida, a abordagem é expansível para um escopo mais amplo, também conhecido como Planetary Health (poluição da água e do solo, segurança alimentar e mudanças climáticas). Dessa forma, a abordagem de Saúde Única ainda contribui para atingir diversos Objetivos de Desenvolvimento Sustentável das Nações Unidas (ODS), tais como Fome Zero e Agricultura Sustentável (ODS 2), Saúde e Bem-Estar (ODS 3), Trabalho Decente e Crescimento Econômico (ODS 8), Indústria Inovação e Infraestrutura (ODS 9) e Consumo e Produção Responsáveis (ODS 12).

A medicina veterinária foi inserida no escopo do trabalho da atenção básica do SUS, particularmente nos Núcleos de Apoio à Saúde da Família (NASF). As ações veterinárias que contribuem para a abordagem em saúde única incluem vigilância epidemiológica, maus-tratos a animais e teoria do elo, atendimento a pessoas em situação de acumulação de animais, prescrição de antibióticos, monitoramento e prevenção de doenças infecciosas em abrigos, vacinação correta, programas de castração, vigilância sanitária, entre outros (Garcia *et al.*, 2019; Mori *et al.*, 2019).

No contexto da auditoria técnica voltada à proteção da fauna, para o Princípio Saúde Única, são elencados três Critérios avaliativos, conforme a Figura 14.

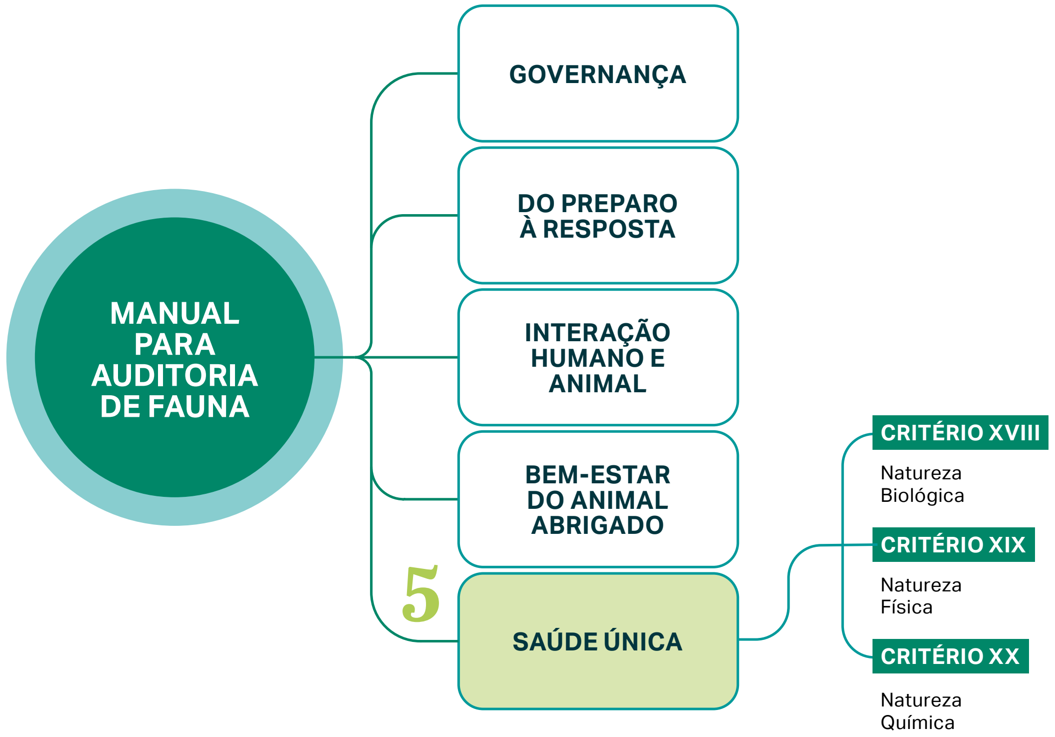

Figura 14 – Critérios avaliativos do Princípio Saúde Única.

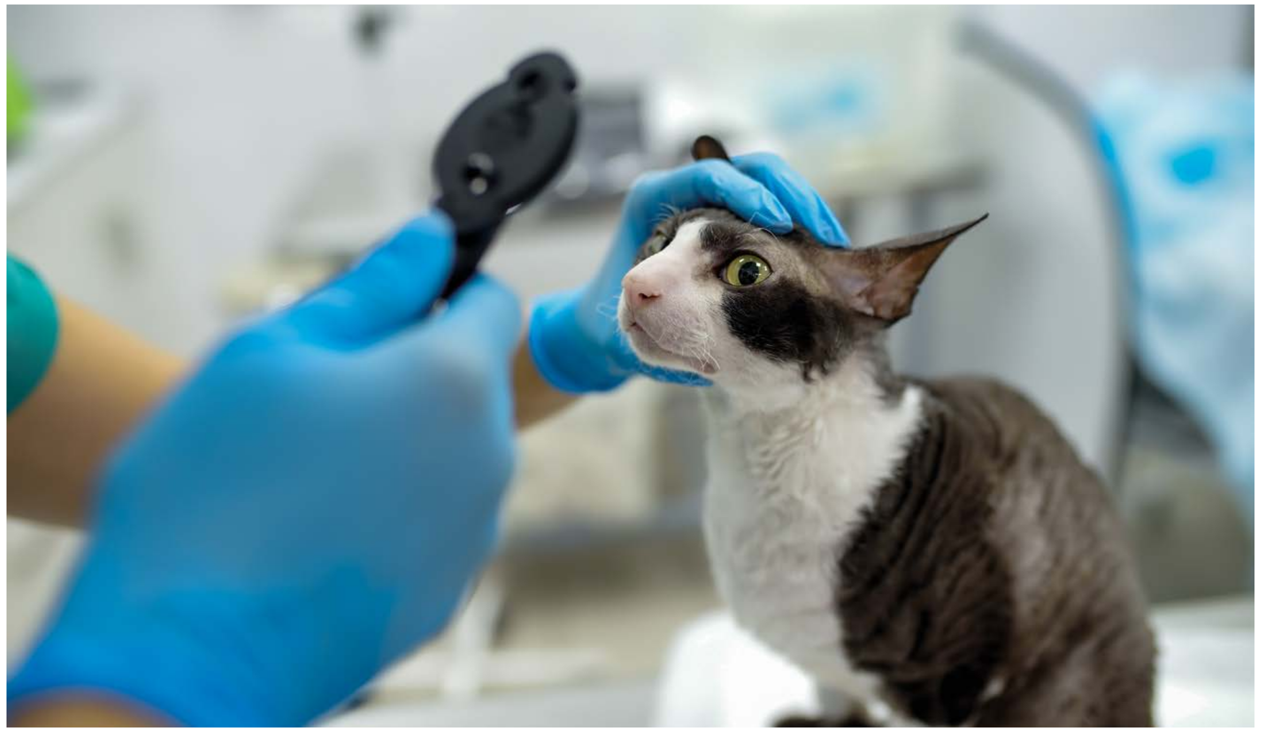

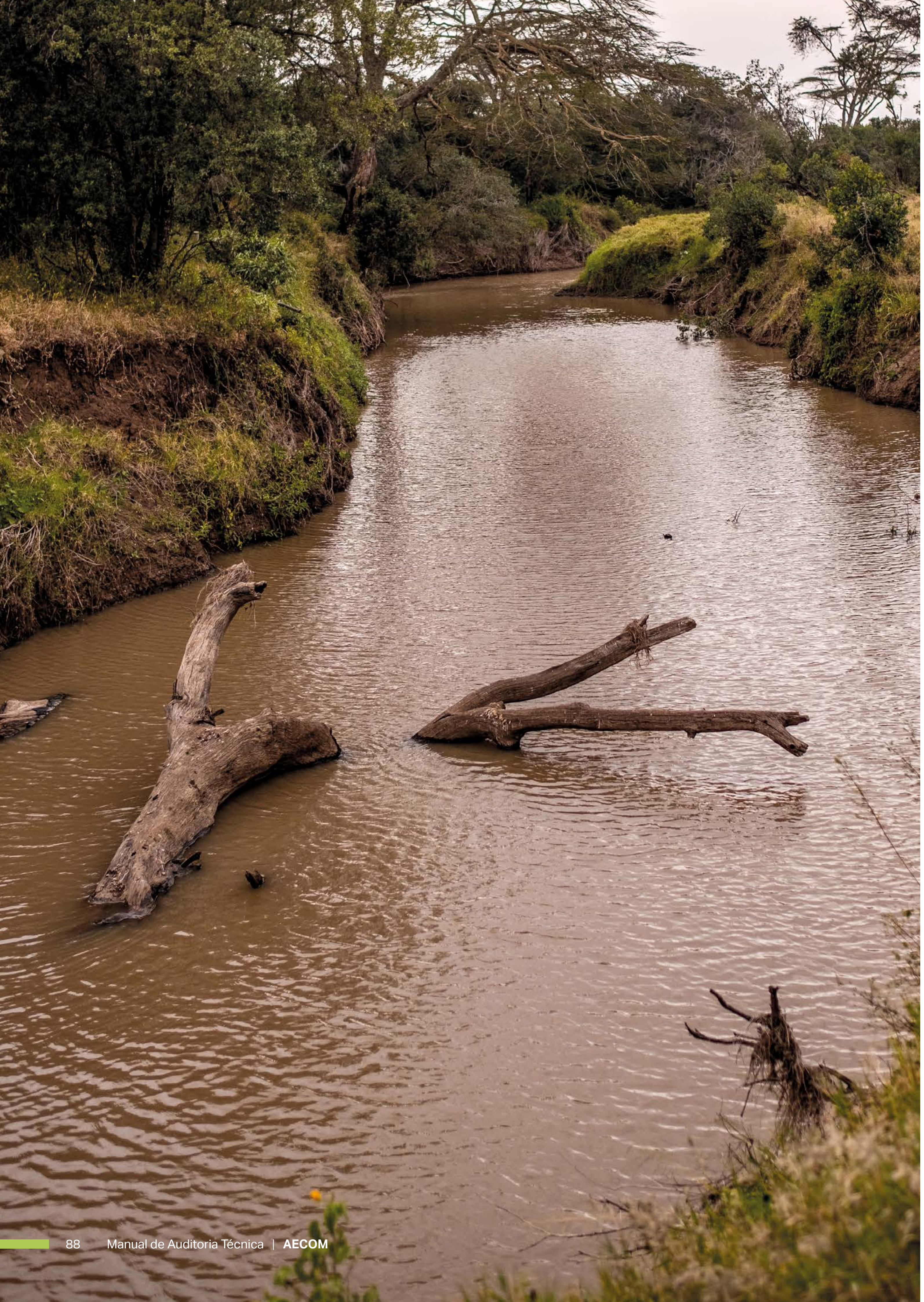

9.1 Critério XVIII – Natureza Biológica

DEFINIÇÃO	Relaciona-se às medidas para evitar a disseminação e contaminação de agentes biológicos potencialmente causadores de agravos à saúde das pessoas, animais e ao ambiente. Inclui a prevenção de doenças e de danos ambientais pelo manejo inadequado em abrigos de fauna, que podem levar ao escape de espécies exóticas invasoras, proliferação e dispersão de espécies vetores, contaminação do ambiente por resíduos biológicos, entre outros.
IMPORTÂNCIA	A saúde pode ser impactada, direta ou indiretamente, pelas mudanças nos ecossistemas que provocam o aumento nos riscos de transmissão de doenças, exposição a novos patógenos, e contaminação da água, solo e alimentos. A redução do risco de zoonoses pode ser alcançada através de programas de vacinação, vigilância epidemiológica, isolamento de áreas e dos animais afetados, além de educação para prevenção de mordidas e outros riscos de exposição a patógenos.<br>Por meio da avaliação dos riscos biológicos para a saúde única é possível elaborar protocolos sanitários, planos de controle de vetores e animais sinantrópicos, controle populacional das espécies abrigadas, entre outros.
ATRIBUIÇÕES DA AUDITORIA TÉCNICA	O auditor deve analisar a eficiência das ações da organização auditada envolvendo a aplicabilidade dos protocolos de biossegurança, bem como o controle de zoonoses, principalmente doenças endêmicas regionalmente como leishmaniose, febre maculosa, hantavirose, esporotricose, malária, entre outras.

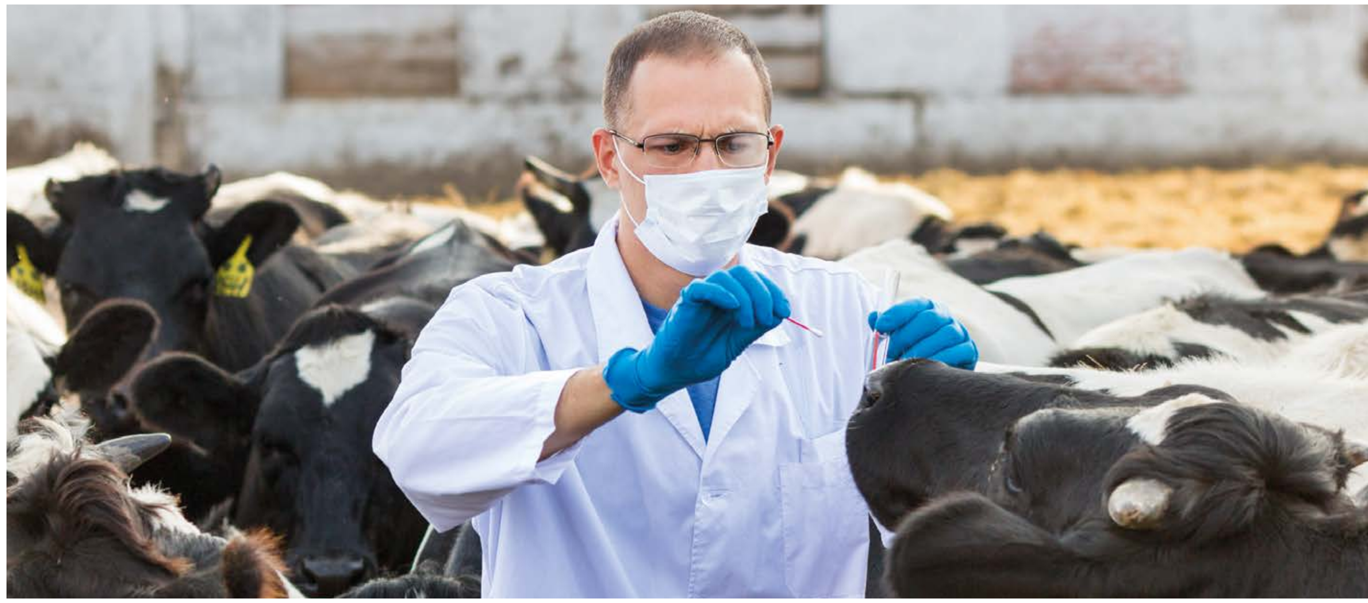

INDICADORES

139. A organização auditada promove a organização de comitês transdisciplinares para tomadas de decisões, dando igual importância para as disciplinas, visando a solução das problemáticas de saúde coletiva nas comunidades, assegurando a equidade entre os atores envolvidos.

140. A organização auditada evidencia que realiza a comunicação aos órgãos competentes quando há detecção de doenças de notificação compulsória nos animais tutelados ou assistidos.

Nota: Consultar a Portaria GM/MS nº 420/2022 (Brasil, 2022) para doenças e agravos de zoonoses detectadas em humanos e a Instrução Normativa MAPA nº 50/2013 (Brasil, 2013) para lista de doenças passíveis da aplicação de medidas de defesa sanitária animal.

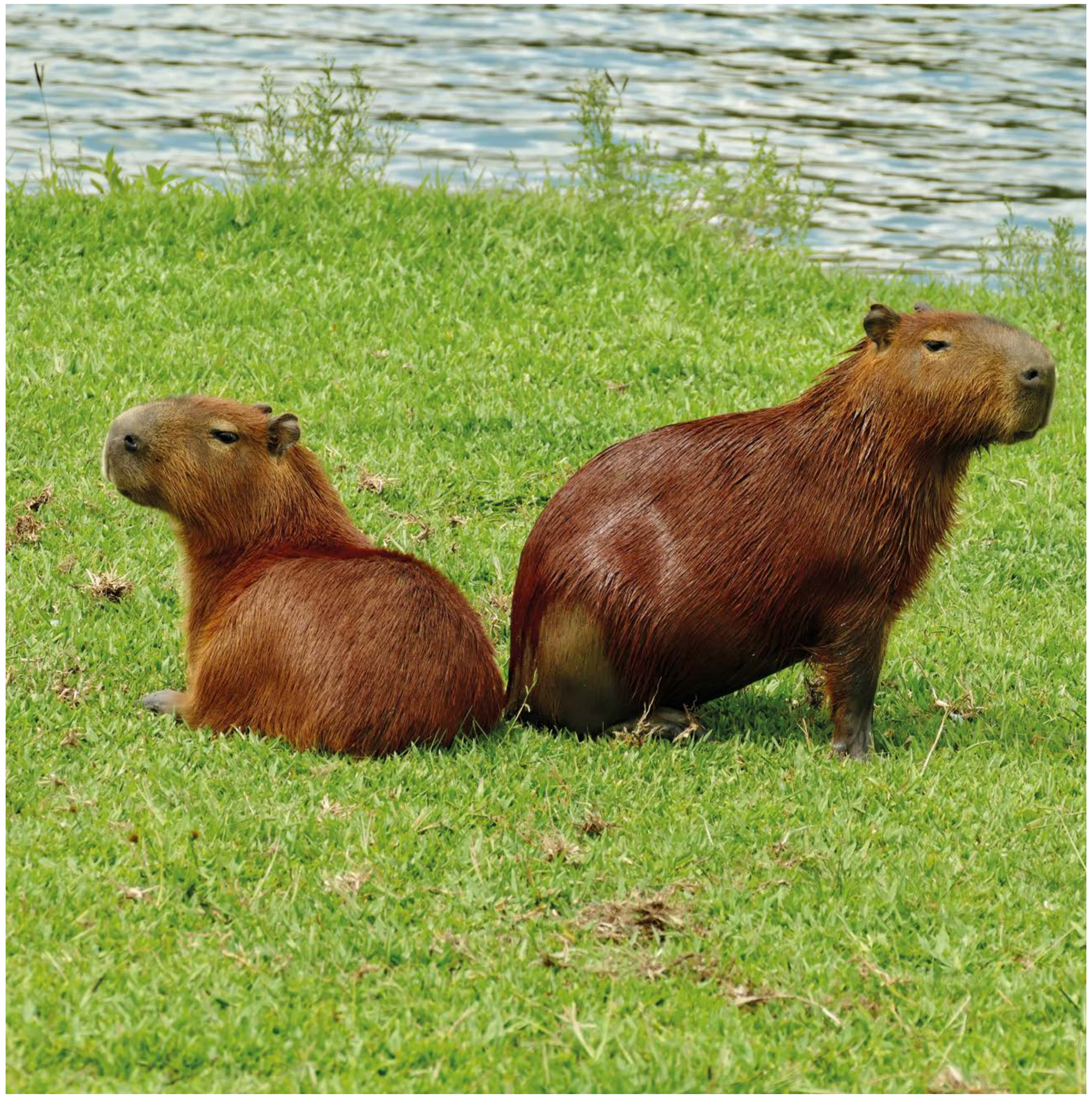

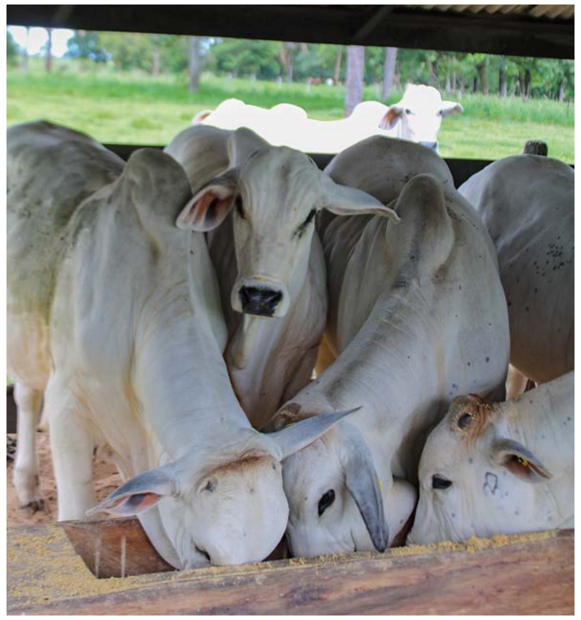

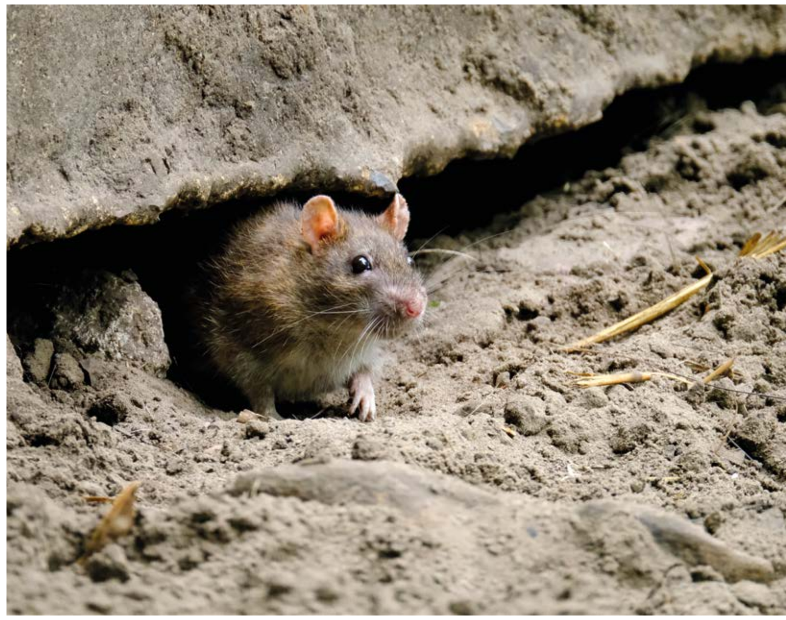

141. A organização auditada prevê o uso racional de antibióticos no tratamento dos animais atingidos pelo desastre.

Nota: Em caso de animais utilizados na produção, é importante que a organização auditada esteja ciente dos períodos de carência dos antimicrobianos utilizados anteriormente pelo tutor e assegure os protocolos pré-estabelecidos em caso de necessidade.

Nota: Para o uso de antimicrobianos, devem ser consideradas as especificidades de cada indivíduo como peso, espécie e resposta do indivíduo ao tratamento, conforme literatura de referência.

Nota: Bactérias de interesse na medicina veterinária como *Salmonella* sp., *Staphylococcus* sp., *Escherichia coli*, entre outras, podem apresentar resistência aos antimicrobianos comumente utilizados na rotina clínica.

A organização auditada demonstra a aplicação de antimicrobianos regulamentados para espécies aquáticas, tratando apenas as populações que necessitam do medicamento e de forma que não comprometa a qualidade hídrica.

142. A organização auditada demonstra realizar manejo integrado de animais sinantrópicos, vetores e reservatórios de patógenos nos abrigos ou nas comunidades afetadas pelo desastre.

143. A organização auditada demonstra que aplica medidas de biosseguridade que minimizam os riscos de dispersão de doenças nos abrigos, de acordo com cada espécie animal abrigada, ou nas comunidades afetadas.

Nota: Para avaliação de riscos epidemiológicos nas comunidades afetadas, deve-se instaurar monitoramento da fauna silvestre e doméstica de interesse da saúde pública.

Nota: Nos abrigos de fauna, é importante a implementação de protocolos preventivos voltados ao controle de zoonoses, como esporotricose, leishmaniose, raiva e doenças transmitidas por carrapatos (febre maculosa, erliquiose, babesiose).

Nota: A auditada deve promover a saúde da comunidade humana e animal, reforçando a importância dos programas de vacinação.

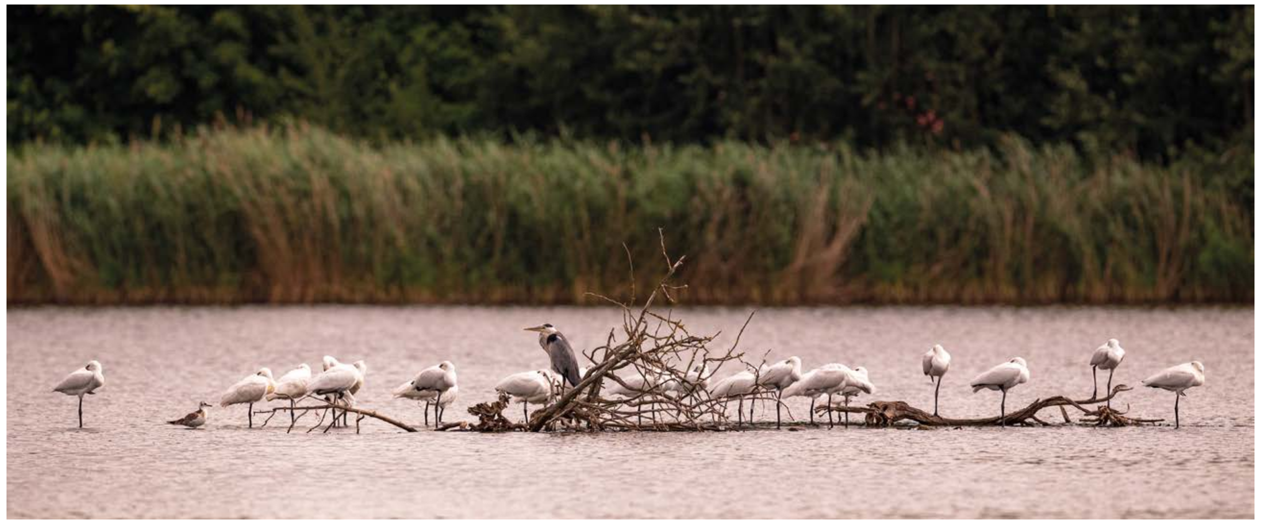

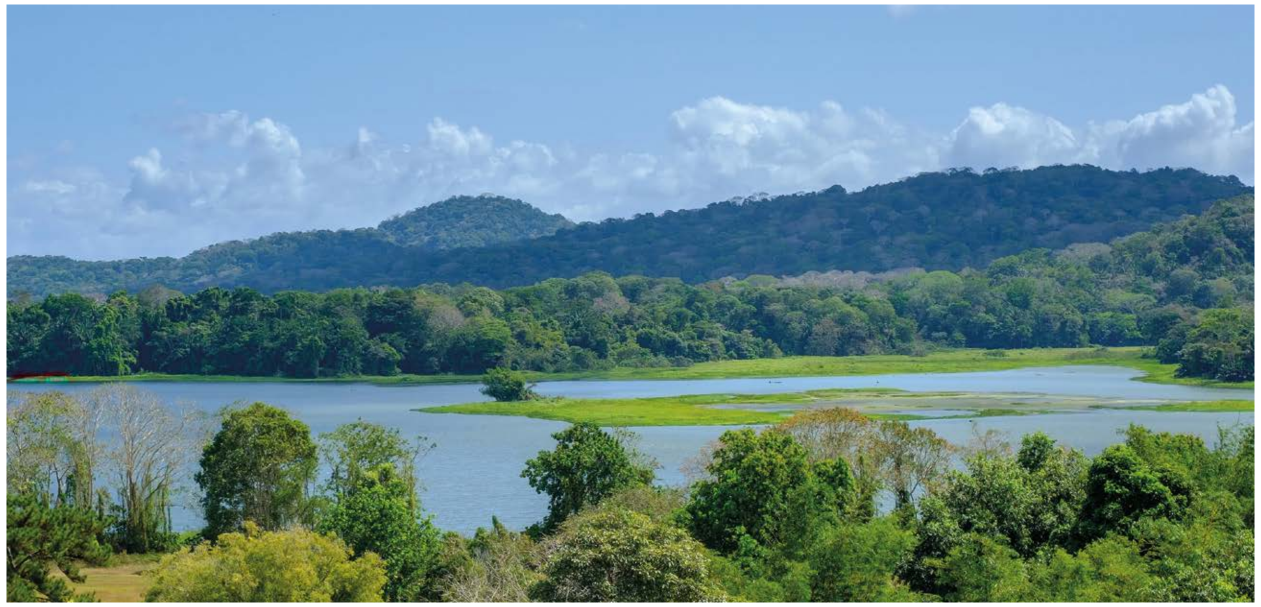

144. A organização auditada, que abriga espécies exóticas com potencial invasor, demonstra implementar medidas anti-fuga e de controle populacional específicas para cada espécie.

145. A organização auditada implementa, para espécies aquáticas, a instalação de barreiras de contenção evitando o escape de propágulos para corpos d'água adjacentes aos abrigos.

146. A organização auditada fornece alimentos e água livre de contaminantes às comunidades atingidas e aos seus animais.

147. A organização auditada disponibiliza e implementa procedimento adequado para coleta, armazenamento e destinação de carcaças e amostras biológicas durante as ações de campo ou resgate.

> Nota: Os procedimentos devem seguir a legislações sanitárias vigentes.
>
> Nota: Em caso de não identificação da espécie animal, faz-se necessária a coleta de material biológico de cada carcaça para futuras análises comparativas de material genético.

9.2 Critério XIX – Natureza Física

DEFINIÇÃO	Relaciona-se à infraestrutura, aos equipamentos e aos procedimentos voltados à segurança dos animais e das pessoas, nas áreas impactadas por desastres e nos abrigos de fauna, incluindo a segurança na gestão de resíduos químicos e biológicos nos abrigos de fauna.
IMPORTÂNCIA	Minimizar os riscos de acidentes de trabalho, doenças ocupacionais e proteger a capacidade de trabalho dos colaboradores.
ATRIBUIÇÕES DA AUDITORIA TÉCNICA	O auditor deve avaliar a infraestrutura local perante as normas de biossegurança de pessoas e animais, com ênfase na adequada localização dos equipamentos, utilização de EPI e EPC, higienização de setores, destinação de resíduos, entre outros.

INDICADORES

148. A organização auditada evidencia que orienta e fiscaliza o cumprimento de protocolos sanitários nos abrigos de fauna.

149. A organização auditada disponibiliza e fomenta a utilização de EPI e EPC pelos colaboradores para qualquer atividade que envolva manejo de animais, manuseio de amostras biológicas, dejetos ou substâncias químicas.

> Nota: O setor de saúde e segurança do trabalho poderá implantar um conjunto de medidas para minimizar os acidentes de trabalho, diminuir as doenças ocupacionais e proteger a capacidade de trabalho dos colaboradores.
>
> Nota: Em todas as atividades envolvendo animais, só deve ser permitido o acesso de pessoas autorizadas com o cumprimento de protocolos sanitários e àutilização de EPI.
>
> Nota: O manuseio de amostras biológicas, dejetos ou substâncias químicas só deve ser permitido por colaboradores utilizando EPI adequado para cada situação.

150. A organização auditada demonstra a qualidade e a disponibilidade dos equipamentos, materiais, insumos e medicamentos, de acordo com a complexidade dos serviços prestados, e assegura que sejam utilizados exclusivamente para os fins a que se destinam.

> Nota: No caso do uso de aparelhos radiográficos, a organização auditada segue as recomendações de segurança determinadas pela legislação vigente, incluindo treinamento dos colaboradores.
>
> Nota: Considerando os equipamentos e mobiliários nos ambientes da instalação utilizada, é importante que se tenha protocolos voltados à adequada utilização desses materiais.

151. A organização auditada possui localização adequada, conforme as necessidades das espécies abrigadas e de acordo com a legislação vigente.

> Nota: É importante que os abrigos de fauna estejam localizados distantes de centros urbanos, a fim de minimizar eventuais problemas de ruídos, odores e dispersão de zoonoses.

152. A organização auditada realiza a destinação dos resíduos, de acordo com seu Plano de Gestão de Resíduos Sólidos (PGRS).

> Nota: Para estabelecimentos voltados à saúde animal, diretrizes técnicas podem ser consultadas em Mori *et al.* (2019).

153. A organização auditada realiza a destinação dos resíduos líquidos, de acordo com o Plano de Tratamento de Efluentes (PTE).

154. A organização auditada assegura o assistencialismo médico para humanos e animais nos abrigos emergenciais para famílias multiespécie.

155. A organização auditada implementa a separação de espécies, quando necessário.

156. A organização auditada possui medidas de contenção ao acesso de animais de vida livre às áreas restritas.

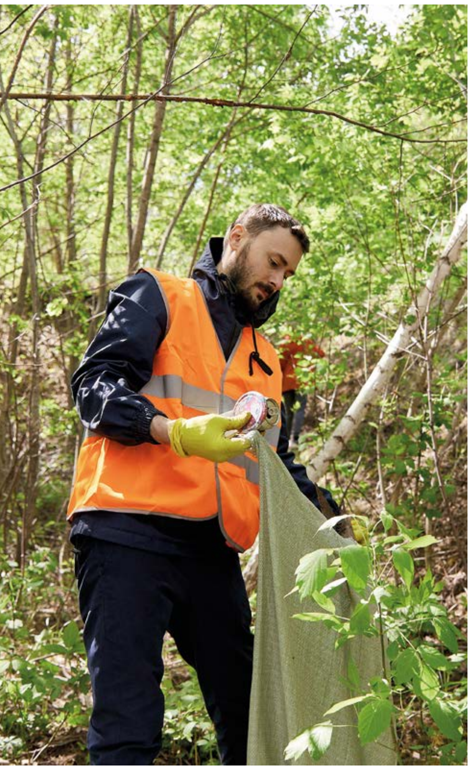

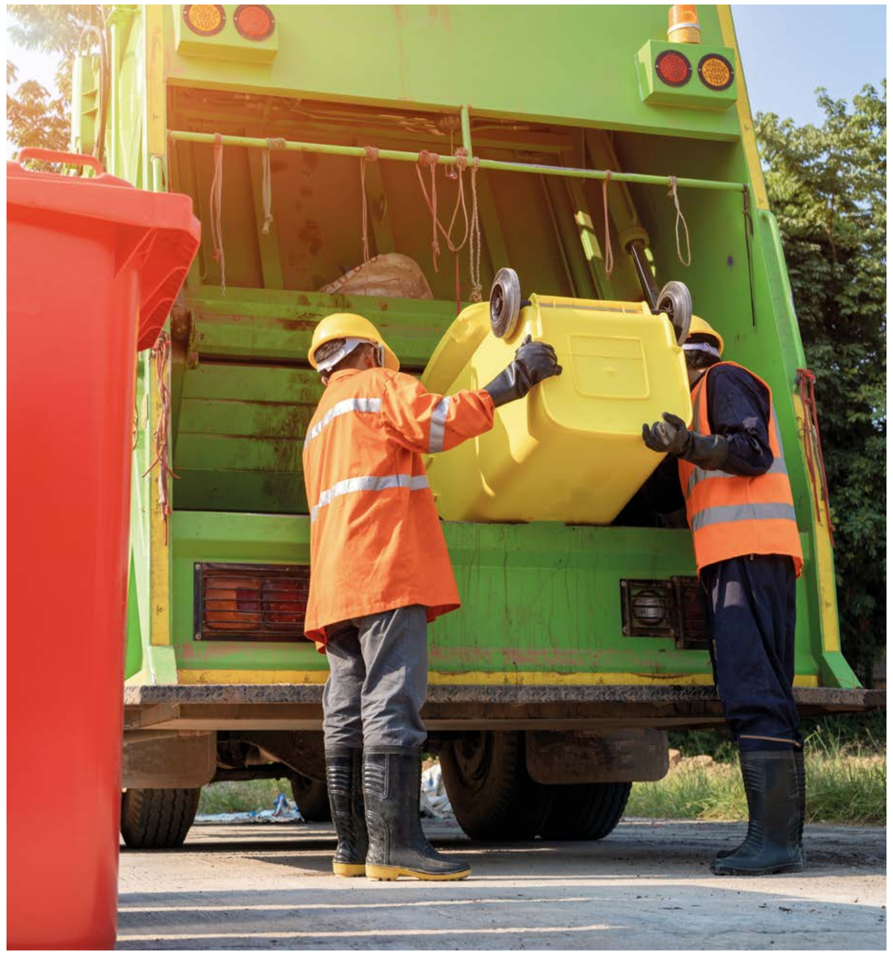

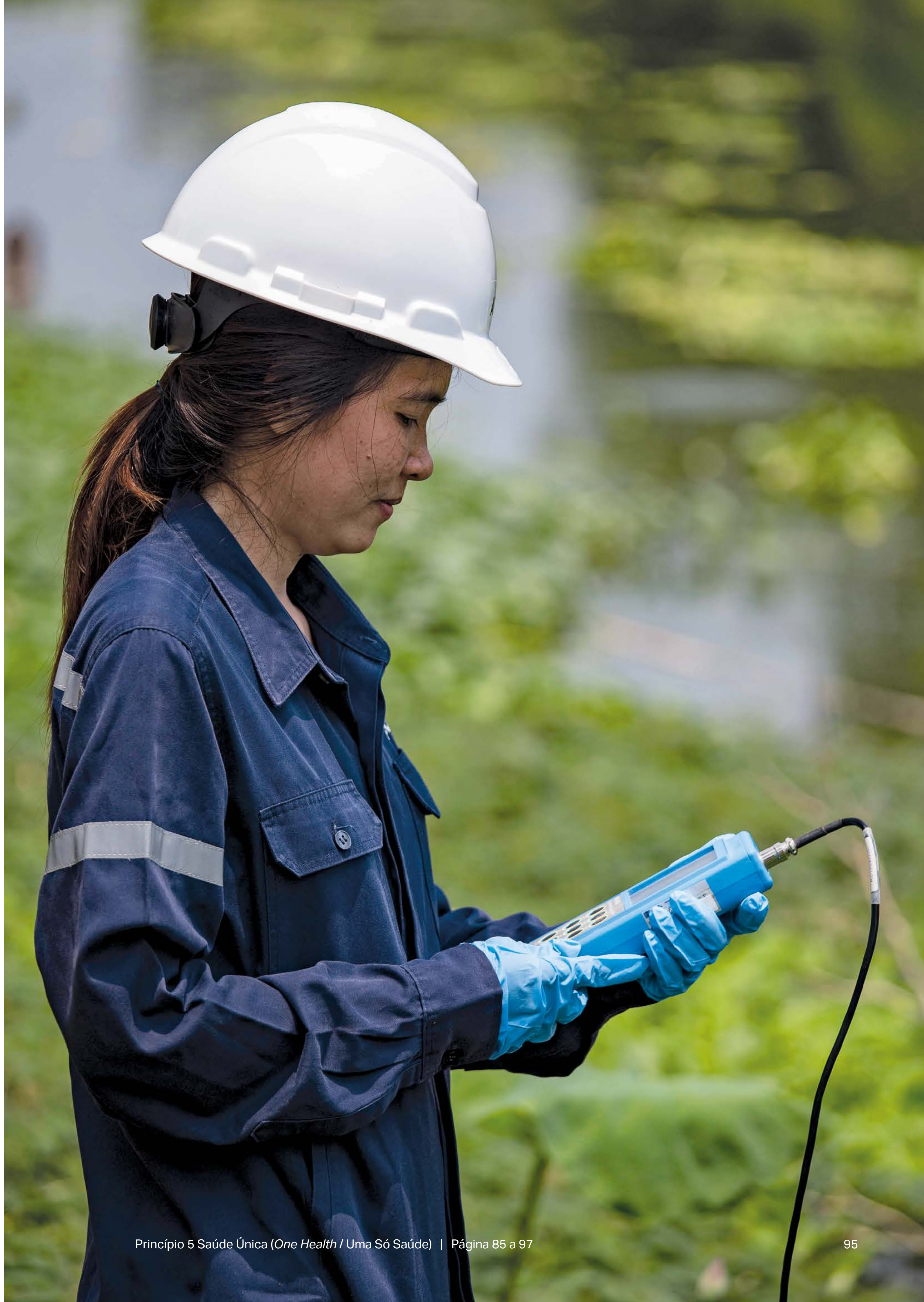

9.3 Critério XX – Natureza Química

DEFINIÇÃO	Sua avaliação no contexto da saúde única objetiva detectar a contaminação do meio ambiente e realizar análises toxicológicas para diagnosticar, controlar e prevenir fatores de riscos ambientais relacionados aos agravos à saúde e surgimento de doenças, considerando o impacto nas gerações do presente e do futuro.
IMPORTÂNCIA	Minimizar os impactos negativos causados pelo desastre e o controle de fatores de riscos ambientais para a saúde, especialmente quanto a ecotoxicidade.
ATRIBUIÇÕES DA AUDITORIA TÉCNICA	O auditor deve avaliar as infraestruturas, ambientes e procedimentos voltados à gestão de resíduos potencialmente negativos ao meio ambiente e à saúde, incluindo os processos de coleta de amostras em campo, os laboratórios.

INDICADORES

157. A organização auditada apresenta laudo técnico da análise da água das áreas atingidas pelo desastre.

158. A organização auditada apresenta laudo técnico da análise do solo das áreas atingidas pelo desastre.

159. A organização auditada possui protocolo de avaliação toxicológica voltado para as diferentes espécies animais resgatadas ou que tiveram contato com agentes contaminantes.

> Nota: As análises toxicológicas precisam manter sua periodicidade e devem ocorrer no ato do resgate durante sua triagem clínica, conforme o agente contaminante ao qual se quer avaliar e a prática laboratorial empregada, atendendo as diretrizes propostas na legislação e boas práticas laboratoriais.
>
> Nota: As matrizes (tecido, soro, urina, vísceras) precisam ser avaliadas de acordo com o biomarcador necessário determinado por um profissional capacitado e mantidas de forma correta sob responsabilidade da organização auditada.
>
> Nota: As análises toxicológicas devem ser realizadas em laboratórios acreditados com apresentação de evidências e da cadeia de custódia.
>
> Nota: É necessário ter um grupo controle como contraponto para análise dos resultados dos exames toxicológicos.
>
> Nota: Os resultados dos exames toxicológicos devem ser avaliados por, no mínimo, dois profissionais capacitados.
>
> Nota: É importante que a análise toxicológica seja realizada também nos animais atingidos que recebem atendimento assistencialista.
>
> Nota: Para os cães de trabalho (a exemplo, cães bombeiros), a avaliação toxicológica deve ocorrer por toda a vida do animal, incluindo análises *post mortem*, considerando a capacidade desses elementos de se ligarem aos tecidos e não serem eliminados pelo organismo.

160. A organização auditada possui protocolo de avaliação toxicológica voltado aos colaboradores que tiveram contato com agentes contaminantes.

161. A organização auditada possui protocolo de descontaminação para os colaboradores e animais que entrarem em contato com agentes contaminantes.

> Nota: A descontaminação deve ser realizada logo após o contato com o contaminante com a finalidade de reduzir os riscos à saúde.
>
> Nota: Exemplos de ações para descontaminação: remoção de roupas contaminadas, lavagem do corpo com substâncias desinfetantes, descontaminação de feridas etc.

162. A organização auditada assegura que animais destinados ao consumo alimentar (corte e leite) nas comunidades atingidas não tiveram contato com substâncias contaminantes e apresentam parâmetros dentro das normas estabelecidas.

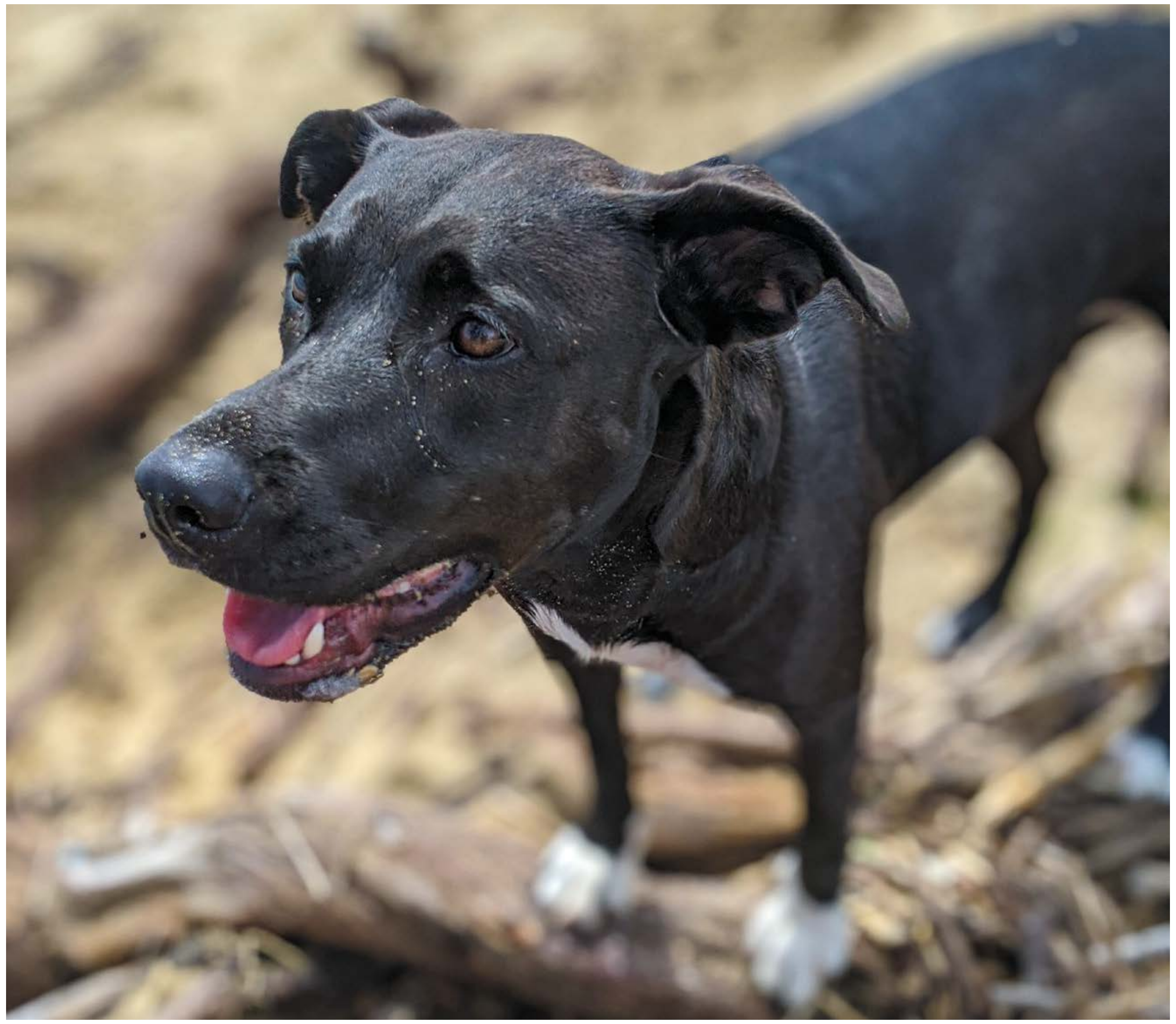

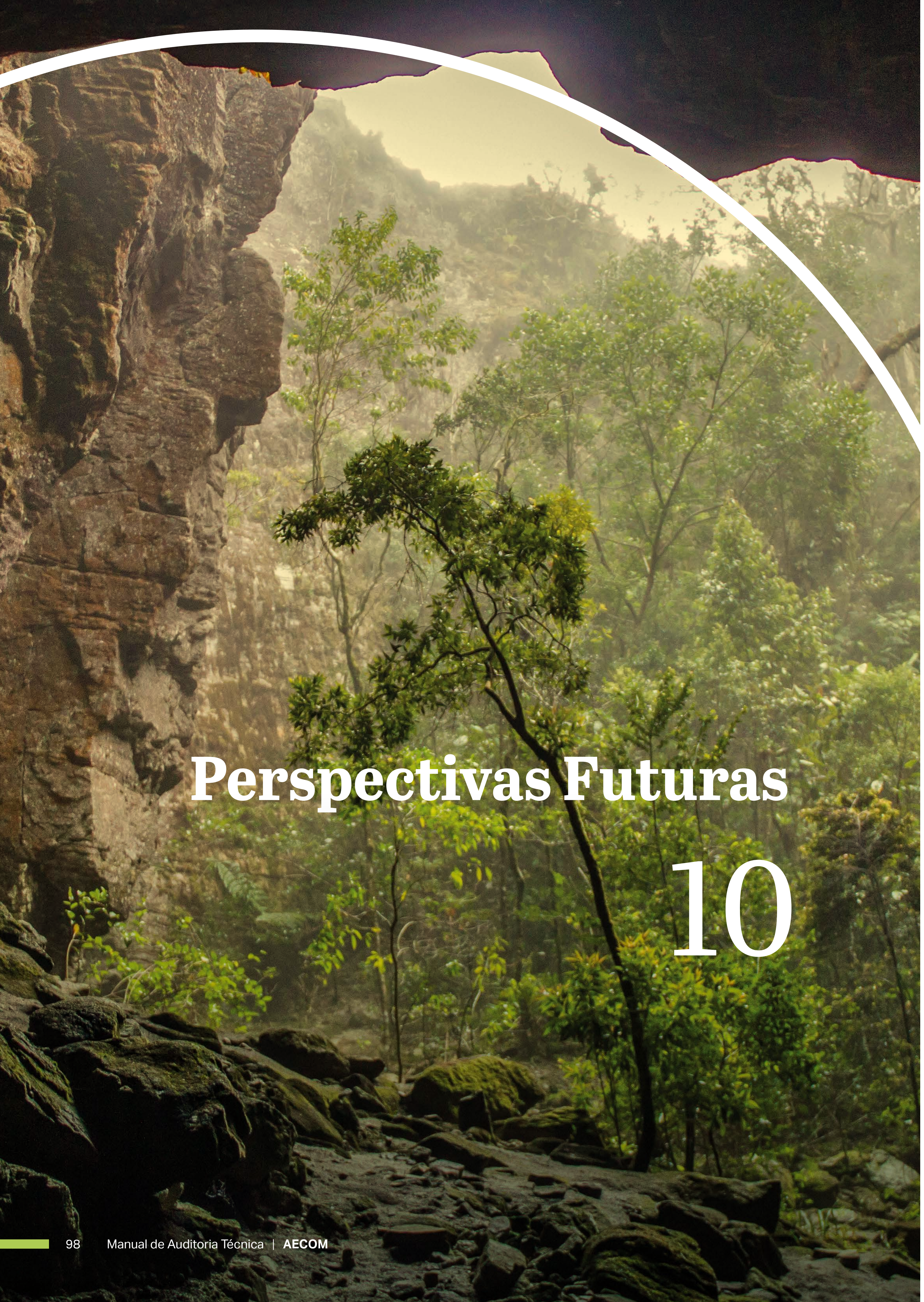

10. Perspectivas Futuras

Desastres em massa são eventos catastróficos e repentinos que requerem uma resposta rápida e eficiente, exigindo, para tanto e sempre que possível, um prévio planejamento por parte dos órgãos responsáveis. O aumento na ocorrência desses desastres em níveis globais é notório e o cenário brasileiro repercute essa tendência mundial (Figura 15). No país, aproximadamente 80% dos desastres são de origem climatológica e hidrológica, como a estiagem e seca (32,91%) e chuvas intensas (26,76%) (MIDR, 2023). Ao longo dos anos, foram acompanhados diversos casos de enchentes no Brasil, como em Nova Friburgo, RJ (2011), Rio de Janeiro, RJ (2012); Rio Branco, AC (2012), Rio Casca, MG (2017); municípios do ES e MG (2020); Petrópolis, RJ (2022), São Sebastião, SP (2024) e diferentes municípios do RS (2024).

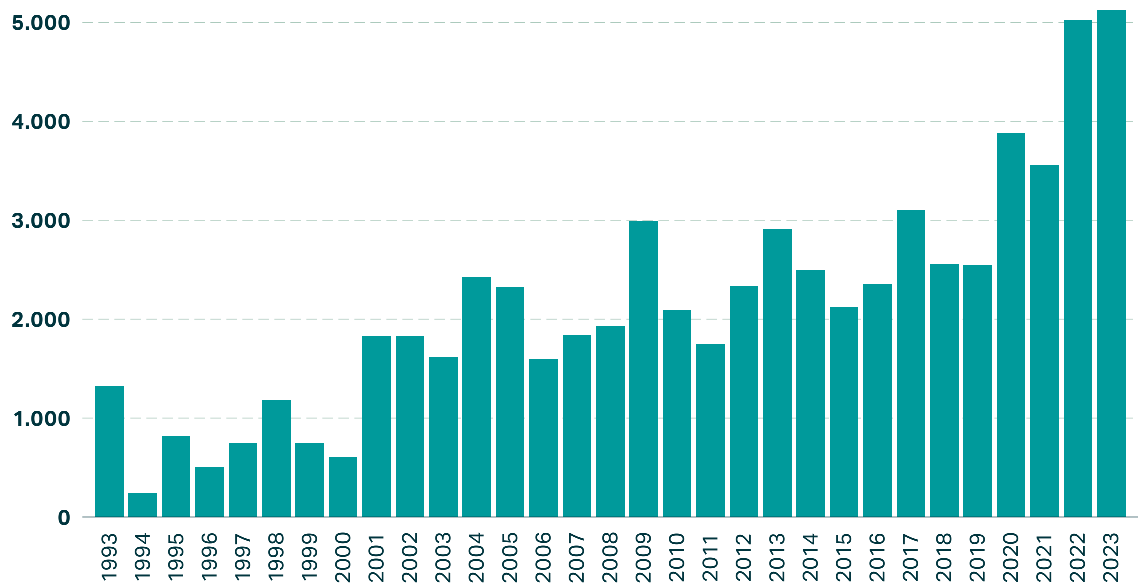

Figura 15 – Ocorrência de desastres no Brasil, 1993 – 2023 (MIDR, 2023).

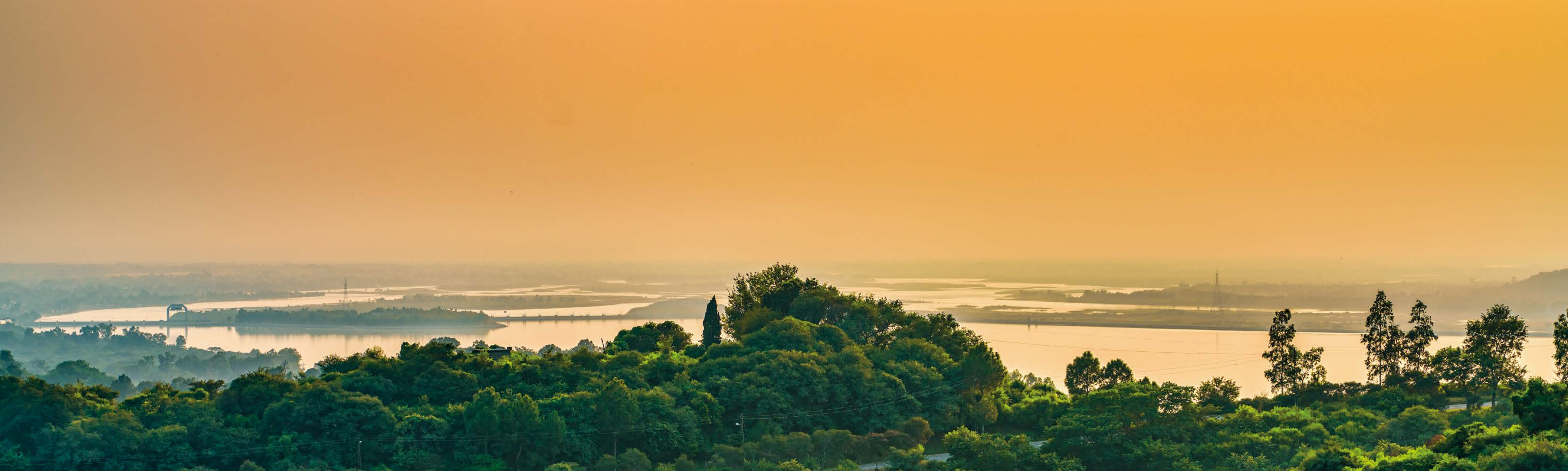

Relacionados às questões climáticas, 27 municípios brasileiros possuem leis e 4 apresentam decretos aprovados. Nas esferas federal e estadual, entraram em vigor até 2021, respectivamente 38 e 165 determinações legais sobre as mudanças no clima (Moreira *et al.*, 2021; Clima de Eleição, 2024). Além disso, 44 projetos de leis estaduais estão em tramitação (Clima de Eleição, 2024). Considerado um marco legal no país, há 15 anos, o governo federal criou a Política Nacional Sobre Mudança do Clima (PNMC), na qual o país se compromete a reduzir as emissões de gases de efeito estufa, fomentando atividades sustentáveis e adotando projetos para mitigar e preparar o país para as mudanças climáticas (Brasil, 2009).

Em 2012, foi instituída, por meio da Lei nº 12.608/2012 (Brasil, 2012), a Política Nacional de Proteção e Defesa Civil (PNPDEC) que estabelece diretrizes para o desenvolvimento de ações de redução de desastres em todo o país, para a prestação de socorro e assistência às populações afetadas e para a elaboração e execução do Plano de Contingência Municipal (PLANCON). Esse plano consiste em um documento de planejamento das ações dos órgãos de resposta do poder público municipal e parceiros para uma atuação em desastres naturais e humanos na cidade. O PLANCON deve ter o apoio do Estado e da União e estabelecer as ações de proteção e defesa civil, assim como prever a realização de audiências públicas e simulados.

O Decreto nº 10.593/2020 (Brasil, 2020) define o PLANCON como um planejamento da resposta, o qual deve conter, minimamente, o mapeamento dos riscos do município e quais as estruturas potencialmente afetadas, levantamento de infraestrutura disponível para atuação em resposta a esses cenários de emergência, contatos de equipe responsável, plano de comunicação com a população e planejamento de evacuação de residências em áreas críticas. A inserção de informações relacionadas à salvaguarda dos animais domésticos e de produção no PLANCON, apesar de ser uma diretriz, ainda é um ponto sensível para o plano elaborado por diferentes municípios. Para a salvaguarda dos animais, é relevante que o PLANCON de todos os municípios contemple a descrição dos procedimentos para evacuação e resgate dos animais, as rotas de fuga, pontos de encontro e de abrigo, considerando o cenário de risco e o mapeamento da população humana e animal atingida.

Um exemplo de plano de contingência que aborda a salvaguarda da fauna doméstica é o Plano de Contingências de Proteção e Defesa Civil – Porto Alegre/RS (CDC – Porto Alegre, 2022). Esse plano incluiu ações de resgate animal, instalação de hospital veterinário de campanha, assistência médico-veterinária, prevenção de acidentes com animais, inclusão dos animais em simulados e destinação dos animais após o resgate, além da criação de um Gabinete da Causa Animal para gerenciar as ações. Destaca-se que tais iniciativas se limitaram a animais domésticos, sem menção à fauna silvestre, exótica e de produção. Ainda para atender as demandas das ocorrências do Rio Grande do Sul, foi criado o Manual de Boas Práticas no Abrigamento de Cães e Gatos em Situações de Desastres, visando fornecer abrigo temporário para animais domésticos, como cães, gatos, pássaros e pequenos mamíferos (gerbils, hamsters, porquinhos-da-índia, coelhos etc.) durante emergências ou desastres (Gebara *et al.*, 2024).

Ao longo dos 5 anos de atuação como auditoria de fauna e acompanhando as implicações pós-desastres, principalmente relacionados às tratativas da salvaguarda animal, verifica-se um embasamento legislativo que permite traçar paralelos com os Princípios, Critérios e Indicadores descritos neste manual. Apesar de não existir um único responsável, poluidor-pagador, em casos de desastres causados por mudanças climáticas, a avaliação dos danos ocorridos, assim como o acompanhamento das ações de resposta, pode ser inspiradas pelos Princípios, Critérios e Indicadores propostos.

O manual em questão apresenta conteúdo estratégico e adaptável para auxiliar na verificação de ações, estruturação de documentos e elaboração de diretrizes que envolvem a salvaguarda animal em diferentes contextos de desastres, podendo ser subsídio para diferentes atores, como os órgãos municipais, estaduais e federais.

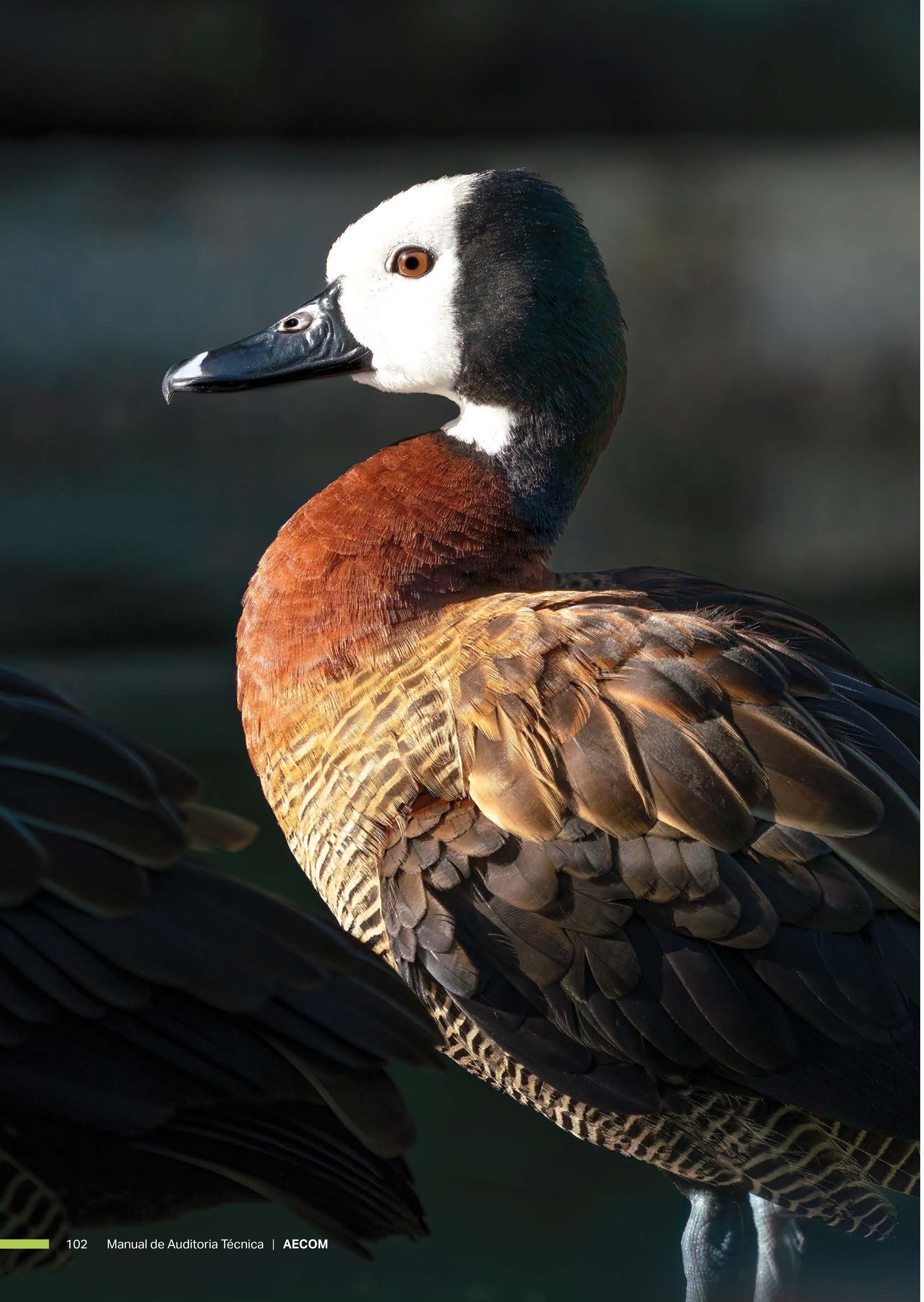

11. Considerações Finais

O Manual de Auditoria Técnica voltado à Proteção da Fauna em Caso de Desastres e Evacuações por Barragens é uma ferramenta para a realização de auditoria documental e *in loco*, visando assegurar o bem-estar da fauna doméstica, de produção e silvestre, considerando a proteção ambiental, em caso de desastres e evacuações. As diretrizes e procedimentos propostos são estabelecidos pela legislação e pelas boas práticas para o bem-estar animal, fundamentados nos Cinco Domínios (nutricional, ambiental, sanitária, comportamental e mental).

O método propõe procedimentos claros para avaliação em auditorias, de forma a facilitar a identificação das não conformidades em atividades destinadas aos cuidados com a fauna. Ele pode ser empregado para auditar ações de evacuação, remoção e abrigo de animais atingidos ou em situações em risco, resgate de animais em cenários de desastre e restauração ecológica; bem como ações de mitigação e compensação em áreas pós-desastre ambiental. Além das ações de resposta em emergência o, manual foi projetado para a avaliação das ações de preparo para a salvaguarda dos animais, garantindo sua proteção eficaz em situações de crise e minimizando riscos à sua segurança e ao seu bem-estar.

O processo avaliativo pode ser complementado com uma avaliação dos pesos associados aos Indicadores, cuja classificação ocorre em diferentes níveis de criticidade, definidos conforme a severidade, riscos associados, fundamentos legais e grau de reversibilidade. Essa proposta visa diminuir a subjetividade e realizar uma avaliação justa e transparente do cumprimento das ações englobadas pelos Princípios, Critérios e Indicadores do método.

Os Princípios e Critérios descritos pretendem ser suficientemente abrangentes como inspiração e referência para aplicação em qualquer ação que cause impacto sobre a fauna, considerando a versatilidade dessas ações nas esferas ambiental, social e econômica. Essas vantagens conferem a esse método a oportunidade de ser utilizado de imediato pela AECOM, ou qualquer outra instituição, em qualquer ação de auditoria técnica que tenha a fauna como interface. Sendo assim, é proposta uma ferramenta inédita e exclusiva que permite avaliar a atuação de uma organização auditada no panorama de pré e pós-desastre. O processo de avaliação proposto neste documento foi desenvolvido inicialmente para desastres de inundação por barragens, todavia, é capaz de ser adaptado e aplicável em diferentes cenários, uma vez que é alicerçado em grandes focos de ação: biodiversidade, medicina de abrigos e saúde única.

O bem-estar animal é um tema complexo e multifacetado com dimensões científicas, éticas, econômicas, jurídicas, religiosas e culturais, além de albergar importantes implicações em políticas comerciais. A colaboração com o poder público para uma educação continuada da comunidade, bem como de *stakeholders*, referente aos

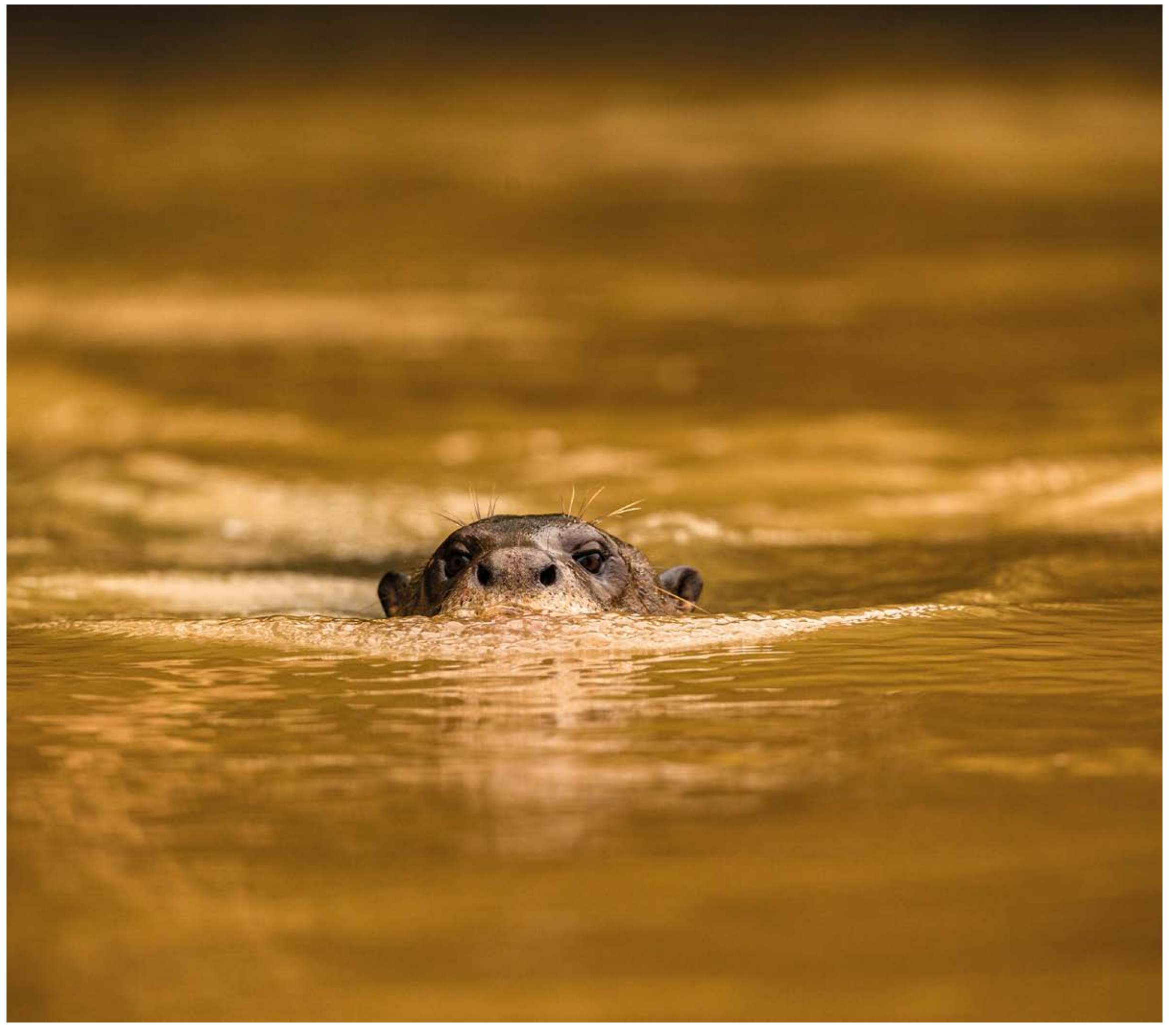

levantamentos de áreas de risco, aos treinamentos comunitários para evacuação preventiva, ao resgate de pessoas e animais atingidos e à disponibilidade de abrigos temporários multiespécie, auxiliaria na disseminação do conhecimento e tomada de decisão em casos de desastres e em pós-desastres. Nesse sentido, torna-se possível preparar comunidades, populações e construir cidades mais resilientes para lidar com eventos de evacuação preventiva e reconstrução. Contudo, esses temas ainda são incipientes no contexto nacional.

Considerando a contemporaneidade do objeto da auditoria técnica voltada à proteção da fauna, é pressuposta a revisão periódica dos Indicadores ou sempre que houver necessidade, visando que esse método continue com a abrangência que a complexidade do tema demanda. Dessa maneira, o Manual de Auditoria Técnica voltado à Proteção da Fauna em Caso de Desastres e Evacuações por Barragens é o início de um processo, almejando que, no futuro, o atendimento aos Princípios, Critérios e Indicadores estabelecidos nesse método sejam certificáveis e possa trazer visibilidade e vantagens às organizações que pratiquem, apoiem e disseminem os preceitos do bem-estar animal e da Saúde Única.

12. Bibliografia

1. | Aguiar, R. O. G.; Ribeiro, A. R. B.; Balda, A. C.; Ribeiro, P. M. T. O bem-estar de cães na prática hospitalar. **Enciclopédia Biosfera**. Goiânia, v.14, n.26, 446–461, 2017. Disponível em: https://www.conhecer.org.br/enciclop/2017b/agrar/o%20bem%20estar%20de%20caes.pdf. Acesso em: 23/07/2024.

2. | Allen, K.; Blascovich, J.; Mendes, W. B. Social interaction and cardiovascular reactivity within marriage: a focus on couples with and without pet cats and dogs. **Psychosomatic Medicine**. 60, 100, 1998. Disponível em: https://journals.lww.com/psychosomaticmedicine/pages/default.aspx. Acesso em: 23/07/2024.

3. | Andrews. K.; Birch, J.; Sebo, J. *et al.* **The New York Declaration on Animal Consciousness**. 2024. Disponível em: https://sites.google.com/nyu.edu/nydeclaration/declaration. Acesso em: 19/06/2024.

4. | Animal Ethics. **A Declaração de Nova York sobre a Consciência Animal enfatiza as implicações éticas da consciência animal**. 2024. Disponível em: https://www.animal-ethics.org/a-declaracao-de-nova-york-sobre-a--consciencia-animal-enfatiza-as-implicacoes-eticas-da--consciencia-animal/. Acesso em: 01/07/2024.

5. | Araújo, S. B. Administração de desastres – Conceitos e Tecnologias. **Sygma SMS**. Rio de Janeiro, 154 p., 2021. Disponível em: https://www.defesacivil.pr.gov.br/sites/defesa-civil/arquivos_restritos/files/documento/2018-12/AdministracaodeDesastres.pdf. Acesso em: 21/06/2024.

6. | Artaxo, P. As três emergências que nossa sociedade enfrenta: saúde, biodiversidade e mudanças climáticas. **Estudos avançados**. São Paulo, 34, 53–66, 2020. Disponível em: https://www.revistas.usp.br/eav/article/view/178752. Acesso em: 23/07/2024.

7. | Ataide Júnior, V. D. P. Capacidade Processual dos Animais: A Judicialização do Direito Animal no Brasil. **Editora Revista dos Tribunais**. São Paulo, Ed. 2022. Disponível em: https://www.jusbrasil.com.br/doutrina/capacidade-processual-dos-animais-ed-2022/1672936674. Acesso em: 20/06/2024.

8. | Ataide Júnior, V. D. P.; Lima, Y. F. Teoria das Capacidades Jurídicas Animais. **Revista Brasileira de Direito Animal**. 19 (2), V052403, 2024. DOI: https://doi.org/10.9771/rbda.v19i0.59041

9. | Bannasch, M. J.; Foley, J. E. Epidemiologic evaluation of multiple respiratory pathogens in cats in animal shelters. **Journal of Feline Medicine and Surgery**. 7, 109–119, 2005. DOI: https://doi.org/10.1016/j.jfms.2004.07.004

10. | Barnard, S.; Pedernera, C.; Candeloro, L.; Ferri, N.; Velarde, A.; Dalla Villa, P. Development of a new welfare assessment protocol for practical application in long-term dog shelters. **Veterinary Record**. v178, n.1, p.18-18, 2016. DOI: 10.1136/vr.103336

11. | Blokhuis, H. J.; Miele, M.; Veissier, I.; Jones, B. Improving farm animal welfare: science and society working together: the Welfare Quality approach. **Wageningen Academic Publishers**. The Netherlands. 229 p, 2013. https://edepot.wur.nl/255920

12. | Bonella, A. E. A ética no uso de animais. **Philósophos – Revista de Filosofia**, Goiânia, v. 17, n. 2, p. 11–41, 2012. DOI: 10.5216/phi.v17i2.15835. Disponível em: https://revistas.ufg.br/philosophos/article/view/15835. Acesso em: 14/08/2024.

13. | BRASIL. Agência Nacional de Mineração – ANM. Resolução da Agência Nacional de Mineração (ANM) nº 175 de 1 de agosto de 2024. **Diário Oficial da União**: seção 1, Brasília, DF, p 165. Altera a Resolução ANM nº 95, de 7 de fevereiro de 2022, que consolida os atos normativos que dispõem sobre segurança de barragens de mineração. Disponível em: https://www.in.gov.br/web/dou/-/resolucao-anm-n-175-de-1-de-agosto-de-2024-575984378.

14. | BRASIL. Conselho Federal de Biologia – CFBio. **Diário Oficial da União**: seção 1, Brasília, DF, 4 p. Resolução nº 538, de 06 de dezembro de 2019. Dispõe sobre a atuação do Biólogo na área de Análises Laboratoriais Animal e dá outras providências. Disponível em: https://cfbio.gov.br/2019/12/11/resolucao-no-538-de-06-de-dezembro-de-2019/. Acesso em: 18/07/2024.

15. | BRASIL. Conselho Federal de Biologia – CFBio. Resolução nº 300, de 7 de dezembro de 2012. **Diário Oficial da União**: seção 1, Brasília, DF, 18 p. Estabelece os requisitos mínimos para o Biólogo atuar em pesquisa, projetos, análises, perícias, fiscalização, emissão de laudos, pareceres e outras atividades profissionais nas áreas de Meio Ambiente e Biodiversidade, Saúde e, Biotecnologia e Produção. Disponível em: https://cfbio.gov.br/2012/12/27/resolucao-no-300-de-7-de-dezembro-de-2012/. Acesso em: 18/07/2024.

16. | BRASIL. Conselho Federal de Biologia – CFBio. Resolução nº 301, de 8 de dezembro de 2012. **Diário Oficial da União**: seção 1, Brasília, DF, 11 p. Dispõe sobre os procedimentos de captura, contenção, marcação, soltura e coleta de animais vertebrados in situ e ex situ, e dá outras providências. Disponível em: https://www.crbio01.gov.br/media/view/2016/06/resolu_o_cfbio_n_2012301_sobre_captura_de_vertebrados_362.pdf. Acesso em: 18/07/2024.

17. | BRASIL. Conselho Federal de Medicina Veterinária – CFMV. **Diário Oficial da União**: seção 1, edição 141, Brasília, DF, p.94. Resolução nº 1.275, de 25 de junho de 2019. Conceitua e estabelece condições para o funcionamento de Estabelecimentos Médico-Veterinários de atendimento a animais de estimação de pequeno porte e dá outras providências. Disponível em: https://www.in.gov.br/en/web/dou/-/resolucao-n-1.275-de-25-de-junho-de-2019-203419719. Acesso em: 18/07/2024.

18. | BRASIL. Conselho Federal de Medicina Veterinária – CFMV. **Diário Oficial da União**: seção 1, Brasília, DF, p.131. Resolução nº 1.284, de 19 de agosto de 2019. Altera o § 2o do Artigo 5o da Resolução CFMV no 1.236, de 26 de outubro de 2018. Disponível em: http://ts.cfmv.gov.br/manual/arquivos/resolucao/1284.pdf. Acesso em: 18/07/2024.

19. | BRASIL. Conselho Federal de Medicina Veterinária – CFMV. **Diário Oficial da União**: seção 1, Brasília, DF, p. 133-134.Resolução nº 1.236, de 26 de outubro de 2018. Define e caracteriza crueldade, abuso e maus-tratos contra animais vertebrados, dispõe sobre a conduta de médicos veterinários e zootecnistas e dá outras providências. Disponível em: https://manual.cfmv.gov.br/arquivos/resolucao/1236.pdf. Acesso em: 18/07/2024.

20. | BRASIL. Conselho Federal de Medicina Veterinária – CFMV. **Diário Oficial da União**: seção 1, Brasília, DF, p. 174-175. Resolução nº 1.374, de 02 de dezembro de 2020. Dispõe sobre a responsabilidade técnica, atividades clínico-laboratoriais, estrutura e funcionamento dos laboratórios clínicos de diagnóstico veterinário, postos de coleta, laboratórios de patologia veterinária e dá outras providências. Disponível em: https://manual.cfmv.gov.br/arquivos/resolucao/1374.pdf. Acesso em: 18/07/2024.

21. | BRASIL. Conselho Federal de Medicina Veterinária – CFMV. **Diário Oficial da União**: seção 1, Brasília, DF, p. 112. Resolução nº 1.321, de 24 de abril de 2020. Institui normas sobre os documentos no âmbito da clínica médico-veterinária e dá outras providências. Disponível em: https://manual.cfmv.gov.br/arquivos/resolucao/1321.pdf. Acesso em: 18/07/2024.

22. | BRASIL. Conselho Federal de Medicina Veterinária – CFMV. **Diário Oficial da União**: seção 1, Brasília, DF, p. 173-174. Resolução nº 887, de 15 de fevereiro de 2008. Dispõe sobre os procedimentos cirúrgicos em animais de produção e em animais silvestres; e cirurgias mutilantes em pequenos animais e dá outras providências. Disponível em: https://manual.cfmv.gov.br/arquivos/resolucao/877.pdf. Acesso em: 18/07/2024.

23. | BRASIL. Conselho Federal de Medicina Veterinária – CFMV. **Diário Oficial da União**: seção 1, Brasília, DF, p. 111-112. Resolução nº 1.511, de 28 de março de 2023. Institui diretrizes para a atuação de médicos-veterinários e zootecnistas em desastres em massa envolvendo animais domésticos e selvagens. Disponível em: http://ts.cfmv.gov.br/manual/arquivos/resolucao/1511.pdf. Acesso em: 12/08/2024.

24. | BRASIL. Conselho Federal de Medicina Veterinária – CFMV. **Nota Técnica**: Gabinete da Presidência do CFMV. Brasília, DF, 12 p. Resolução nº 1.000 de 11 de maio de 2012. Dispõe sobre procedimentos e métodos de eutanásia em animais e dá outras providências. Disponível em: https://www.cfmv.gov.br/wp-content/uploads/2022/06/NotaTecnicaCNSPV.pdf. Acesso em: 18/07/2024.

25. | BRASIL. Conselho Federal de Medicina Veterinária – CFMV. Resolução nº 1.069, de 27 de outubro de 2014. **Diário Oficial da União**: seção 1, Brasília, DF, 56 p. Dispõe sobre diretrizes gerais de responsabilidade técnica em estabelecimentos comerciais de exposição, manutenção, higiene estética e venda ou doação de animais, e dá outras providências. Disponível em: https://manual.cfmv.gov.br/arquivos/resolucao/1069.pdf. Acesso em: 18/07/2024.

26. | BRASIL. Constituição da República Federativa do Brasil de 1988. **Diário Oficial da União**: seção 1, Brasília, DF, p. 202. Presidência da República. Casa Civil. Subchefia de recursos jurídicos. Disponível em: https://www2.camara.leg.br/legin/fed/consti/1988/constituicao-1988-5-outubro-1988-322142-publicacaooriginal-1-pl.html. Acesso em: 18/07/2024.

27. | BRASIL. Decreto Federal nº 12.007 de 25 de abril de 2024. **Diário Oficial da União**: seção 1, Brasília, DF, p. 3. Institui o Comitê Técnico Interinstitucional de Uma Só Saúde. Presidência da República. Casa Civil. Secretaria Especial para Assuntos Jurídicos. Disponível em: https://www2.camara.leg.br/legin/fed/decret/2024/decreto-12007-25-abril-2024-795542-publicacaooriginal-171631-pe.html. Acesso em: 18/07/2024.

28. | BRASIL. Decreto Federal nº 4.339 de 22 de agosto de 2002. **Diário Oficial da União**: seção 1, Brasília, DF, p. 2. Institui princípios e diretrizes para a implementação da Política Nacional da Biodiversidade. Presidência da República. Casa Civil. Subchefia para assuntos jurídicos. Disponível em: https://pesquisa.in.gov.br/imprensa/jsp/visualiza/index.jsp?data=23/08/2002&jornal=1&pagina=2&totalArquivos=72. Acesso em: 18/07/2024.

29. | BRASIL. Decreto Federal nº 6.514, de 22 de julho de 2008. **Diário Oficial da União**: seção 1, Brasília, DF, p. 1. Dispõe sobre as infrações e sanções administrativas ao meio ambiente, estabelece o processo administrativo federal para apuração destas infrações, e dá outras providências. Presidência da República. Casa Civil. Subchefia para assuntos jurídicos. Disponível em: https://legislacao.presidencia.gov.br/atos/?tipo=DEC&numero=6514&ano=2008&ato=a87cXRE50dVpWTdf. Acesso em: 18/07/2024.

30. | BRASIL. Decreto Federal nº 9.571 de 21 de novembro de 2018. **Diário Oficial da União**: seção 1, Brasília, DF, p. 1. Estabelece as diretrizes nacionais sobre empresas e direitos humanos. Presidência da República. Secretaria Geral. Subchefia para Assuntos Jurídicos. Disponível em: https://pesquisa.in.gov.br/imprensa/jsp/visualiza/index.jsp?jornal=515&pagina=1&data=22/11/2018&totalArquivos=99. Acesso em: 18/07/2024.

31. BRASIL. Lei Complementar nº 140 de 8 de dezembro de 2011. **Diário Oficial da União**: seção 1, Brasília, DF, p. 1. Fixa normas, nos termos dos incisos III, VI e VII do caput e do parágrafo único do art. 23 da Constituição Federal, para a cooperação entre a União, os Estados, o Distrito Federal e os Municípios nas ações administrativas decorrentes do exercício da competência comum relativas à proteção das paisagens naturais notáveis, à proteção do meio ambiente, ao combate à poluição em qualquer de suas formas e à preservação das florestas, da fauna e da flora; e altera a Lei no 6.938, de 31 de agosto de 1981. Presidência da República Casa Civil Subchefia para Assuntos Jurídicos. Disponível em: https://legislacao.presidencia.gov.br/atos/?tipo=LCP&numero=140&ano=2011&ato=bbdITSq1UMVpWTdf5. Acesso em: 18/07/2024.

32. | BRASIL. Lei Federal nº 12.187 de 29 de dezembro de 2009. **Diário Oficial da União**: seção 1, Brasília, DF, p. 109. Institui a Política Nacional sobre Mudança do Clima - PNMC e dá outras providências. Presidência da República. Secretaria-Geral. Subchefia para Assuntos Jurídicos. Disponível em: https://www.planalto.gov.br/ccivil_03/_ato2007-2010/2009/lei/l12187.htm. Acesso em: 23/07/2024.

33. | BRASIL. Lei Federal nº 14.064 de 29 de setembro de 2020. **Diário Oficial da União**: seção 1, Brasília, DF, p. 4. Altera a Lei nº 9.605, de 12 de fevereiro de 1998, para aumentar as penas cominadas ao crime de maus-tratos aos animais quando se tratar de cão ou gato. Presidência da República. Secretaria-Geral. Subchefia para Assuntos Jurídicos. Disponível em: https://www.planalto.gov.br/ccivil_03/_ato2019-2022/2020/lei/l14064.htm. Acesso em: 23/07/2024.

34. | BRASIL. Lei Federal nº 9.966 de 28 de abril de 2000 de 28 de abril de 2000. **Diário Oficial da União**: seção 1, Brasília, DF, p. 1. Dispõe sobre a prevenção, o controle e a fiscalização da poluição causada por lançamento de óleo e outras substâncias nocivas ou perigosas em águas sob jurisdição nacional e dá outras providências. Presidência da República. Casa Civil. Subchefia para Assuntos Jurídicos. Disponível em: https://www.planalto.gov.br/ccivil_03/leis/l9966.htm. Acesso em: 23/07/2024.

35. | BRASIL. Lei Federal nº 12.305 de 2 de agosto de 2010. **Diário Oficial da União**: seção 1, Brasília, DF, p. 3. Institui a Política Nacional de Resíduos Sólidos; altera a Lei nº 9.605, de 12 de fevereiro de 1998; e dá outras providências. Presidência da República. Casa Civil. Subchefia para Assuntos Jurídicos. Disponível em: https://pesquisa.in.gov.br/imprensa/jsp/visualiza/index.jsp?data=03/08/2010&jornal=1&pagina=3&totalArquivos=84. Acesso em: 18/07/2024.

36. | BRASIL. Lei Federal nº 12.334, de 20 de setembro de 2010. **Diário Oficial da União**: seção 1, Brasília, DF, p. 1. Estabelece a Política Nacional de Segurança de Barragens destinadas à acumulação de água para quaisquer usos, à disposição final ou temporária de rejeitos e à acumulação de resíduos industriais, cria o Sistema Nacional de Informações sobre Segurança de Barragens e altera a redação do art. 35 da Lei nº 9.433, de 8 de janeiro de 1997, e do art. 4º da Lei nº 9.984, de 17 de julho de 2000. Presidência da República. Casa Civil. Subchefia para assuntos jurídicos. Disponível em: https://pesquisa.in.gov.br/imprensa/jsp/visualiza/index.jsp?jornal=1&pagina=1&data=21/09/2010&totalArquivos=120. Acesso em: 18/07/2024.

37. | BRASIL. Lei Federal nº 12.340 de 1 de dezembro de 2010. **Diário Oficial da União**: seção 1, Brasília, DF, p. 1. Dispõe sobre as transferências de recursos da União aos órgãos e entidades dos Estados, Distrito Federal e Municípios para a execução de ações de prevenção em áreas de risco de desastres e de resposta e de recuperação em áreas atingidas por desastres e sobre o Fundo Nacional para Calamidades Públicas, Proteção e Defesa Civil; e dá outras providências. Presidência da República. Casa Civil. Subchefia para Assuntos Jurídicos. Disponível em: https://www.planalto.gov.br/ccivil_03/_ato2007-2010/2010/lei/l12340.htm. Acesso em: 23/07/2024.

38. | BRASIL. Lei Federal nº 12.608 de 10 de abril de 2012. Institui a Política Nacional de Proteção e Defesa Civil – PNPDEC. **Diário Oficial da União**: seção 1, Brasília, DF, p. 1. Dispõe sobre o Sistema Nacional de Proteção e Defesa Civil - SINPDEC e o Conselho Nacional de Proteção e Defesa Civil - CONPDEC; autoriza a criação de sistema de informações e monitoramento de desastres; altera as Leis nºs 12.340, de 1º de dezembro de 2010, 10.257, de 10 de julho de 2001, 6.766, de 19 de dezembro de 1979, 8.239, de 4 de outubro de 1991, e 9.394, de 20 de dezembro de 1996; e dá outras providências. Presidência da República. Casa Civil. Subchefia para Assuntos Jurídicos. Disponível em: https://www.planalto.gov.br/ccivil_03/_ato2011-2014/2012/lei/l12608.htm. Acesso em: 23/07/2024.

39. | BRASIL. Lei Federal nº 12.651, de 25 de maio de 2012. **Diário Oficial da União**: seção 1, Brasília, DF, p. 1. Dispõe sobre a proteção da vegetação nativa; altera as Leis nos 6.938, de 31 de agosto de 1981, 9.393, de 19 de dezembro de 1996, e 11.428, de 22 de dezembro de 2006; revoga as Leis nos 4.771, de 15 de setembro de 1965, e 7.754, de 14 de abril de 1989, e a Medida Provisória nº 2.166-67, de 24 de agosto de 2001; e dá outras providências. Presidência da República. Casa Civil. Subchefia para Assuntos Jurídicos. Disponível em: https://pesquisa.in.gov.br/imprensa/jsp/visualiza/index.jsp?jornal=1&pagina=1&data=28/05/2012&totalArquivos=168. Acesso em: 18/07/2024.

40. | BRASIL. Lei Federal nº 13.426, de 30 de março de 2017. **Diário Oficial da União**: seção 1, Brasília, DF, p. 3. Dispõe sobre a política de controle da natalidade de cães e gatos e dá outras providências. Presidência da República. Secretaria-Geral. Subchefia para Assuntos Jurídicos. Disponível em: https://pesquisa.in.gov.br/imprensa/jsp/visualiza/index.jsp?data=31/03/2017&jornal=1&pagina=3&totalArquivos=136. Acesso em: 18/07/2024.

41. | BRASIL. Lei Federal nº 14.066, de 30 de setembro de 2020. **Diário Oficial da União**: seção 1, Brasília, DF, p. 3. Altera a Lei nº 12.334, de 20 de setembro de 2010, que estabelece a Política Nacional de Segurança de Barragens (PNSB), a Lei nº 7.797, de 10 de julho de 1989, que cria o Fundo Nacional do Meio Ambiente (FNMA), a Lei nº 9.433, de 8 de janeiro de 1997, que institui a Política Nacional de Recursos Hídricos, e o Decreto-Lei nº 227, de 28 de fevereiro de 1967 (Código de Mineração). Presidência da República. Secretaria-Geral. Subchefia para Assuntos Jurídicos. Disponível em: https://pesquisa.in.gov.br/imprensa/jsp/visualiza/index.jsp?data=01/10/2020&jornal=515&pagina=3&totalArquivos=886. Acesso em: 18/07/2024.

42. | BRASIL. Lei Federal nº 14.119 de 13 de janeiro de 2021. **Diário Oficial da União**: seção 1, Brasília, DF, p. 7. Institui a Política Nacional de Pagamento por Serviços Ambientais; e altera as Leis nº 8.212, de 24 de julho de 1991, 8.629, de 25 de fevereiro de 1993, e 6.015, de 31 de dezembro de 1973, para adequá-las à nova política. Diário Oficial da União. Atos do Poder Legislativo. Disponível em: https://pesquisa.in.gov.br/imprensa/jsp/visualiza/index.jsp?data=14/01/2021&jornal=515&pagina=7&totalArquivos=278. Acesso em: 18/07/2024.

43. | BRASIL. Lei Federal nº 5.197, de 3 de janeiro de 1967. **Diário Oficial da União**: seção 1, Brasília, DF, p. 177. Dispõe sobre a proteção à fauna e dá outras providências. Legislação citada anexada pela Coordenação de estudos legislativos – CEDI. Presidência da República. Casa Civil. Subchefia para Assuntos Jurídicos. Disponível em: https://www.planalto.gov.br/ccivil_03/leis/l5197.htm. Acesso em: 18/07/2024.

44. | BRASIL. Lei Federal nº 5.517, de 23 de outubro de 1968. **Diário Oficial da União**: seção 1, Brasília, DF, p. 9401. Dispõe sobre o exercício da profissão de médico- veterinário e cria os Conselhos Federal e Regionais de Medicina Veterinária. Presidência da República. Casa Civil. Subchefia para assuntos jurídicos. Disponível em: https://www.planalto.gov.br/ccivil_03/leis/l5517.htm. Acesso em: 18/07/2024.

45. | BRASIL. Lei Federal nº 6.938, de 31 de agosto de 1981. **Diário Oficial da União**: seção 1, Brasília, DF, p. 16509. Dispõe sobre a Política Nacional do Meio Ambiente, seus fins e mecanismos de formulação e aplicação, e dá outras providências. Presidência da República. Casa Civil, Subchefia para assuntos jurídicos. Disponível em: https://www.planalto.gov.br/ccivil_03/leis/l6938.htm. Acesso em: 18/07/2024.

46. | BRASIL. Lei Federal nº 9.605, de 12 de fevereiro de 1998. **Diário Oficial da União**: seção 1, Brasília, DF, p. 1. Dispõe sobre as sanções penais e administrativas derivadas de condutas e atividades lesivas ao meio ambiente, e dá outras providências. Presidência da República. Casa Civil. Subchefia para assuntos jurídicos. Disponível em: https://www.planalto.gov.br/ccivil_03/leis/l9605.htm. Acesso em: 23/07/2024.

47. | BRASIL. Lei nº 13.709 de 14 de agosto de 2018. **Diário Oficial da União**: seção 1, Brasília, DF, p. 59. Lei Geral de Proteção de Dados Pessoais (LGPD). Esta Lei dispõe sobre o tratamento de dados pessoais, inclusive nos meios digitais, por pessoa natural ou por pessoa jurídica de direito público ou privado, com o objetivo de proteger os direitos fundamentais de liberdade e de privacidade e o livre desenvolvimento da personalidade da pessoa natural. Disponível em: https://www.planalto.gov.br/ccivil_03/_ato2015-2018/2018/lei/L13709compilado.htm. Acesso em: 14/08/2024.

48. | BRASIL. Lei nº 14.792 de 5 de janeiro de 2024. **Diário Oficial da União**: seção 1, Brasília, DF, p. 4. Institui o Dia Nacional da Saúde Única. Presidência da República. Casa Civil. Secretaria Especial para Assuntos Jurídicos. Disponível em: https://www2.camara.leg.br/legin/fed/lei/2024/lei-14792-5-janeiro-2024-795214-publicacao-original-170767-pl.html. Acesso em: 18/07/2024.

49. | BRASIL. Ministério da Agricultura e Pecuária – MAPA. **Diário Oficial da União**: seção 1, Brasília, DF, p. 4. Gabinete da Ministra. Diário Oficial da União. Instrução Normativa nº 22 de 20 de junho de 2013. Define as normas para habilitação de médico veterinário privado para emissão de Guia de Trânsito Animal – GTA e aprovar na forma dos Anexos de I a V da presente Instrução Normativa. Disponível em: https://www.gov.br/agricultura/pt-br/assuntos/sanidade-animal-e-vegetal/saude-animal/transito-animal/cgtqa-legis/in-mapa-no-22-20-06-2013-habilitar-mv-gta.pdf. Acesso em: 18/07/2024.

50. | BRASIL. Ministério da Agricultura e Pecuária – MAPA. **Diário Oficial da União**: seção 1, edição 134, Brasília, DF, p. 2. Gabinete da Ministra. Diário oficial da união. Instrução Normativa nº 48, de 14 de julho de 2020. Aprova as diretrizes gerais para a vigilância da febre aftosa com vistas à execução do Programa Nacional de Vigilância para a Febre Aftosa (PNEFA). Disponível em: https://www.gov.br/agricultura/pt-br/assuntos/sanidade-animal-e-vegetal/saude-animal/programas-de-saude-animal/febre-aftosa/planocontingenciaparafebreaftosa.pdf. Acesso em: 18/07/2024.

51. | Brasil. Ministério da Agricultura e Pecuária – MAPA. **Diário Oficial da União**: seção 1, Brasília, DF, p. 2. Gabinete da Ministra – GM. Instrução Normativa n° 4, de 28 de fevereiro de 2019. Altera a IN nº 4/2015 que instituiu o Programa Nacional de Sanidade de Animais Aquáticos de Cultivo – Aquicultura com Sanidade. Disponível em: https://www.gov.br/agricultura/pt-br/assuntos/sanidade-animal-e-vegetal/saude-animal/programas-de-saude-animal/arquivos-programas-sanitarios/INMAPAn-4de28.02.2019AlteraaINMPA042015.pdf. Acesso em: 18/07/2024.

52. | BRASIL. Ministério da Agricultura e Pecuária – MAPA. **Diário Oficial da União**: seção 2, Brasília, DF, 5. p. Instrução Normativa nº 5, de 1º de março de 2002. Aprova Normas Técnicas para o controle da raiva dos herbívoros domésticos. Disponível em: https://sistemasweb.agricultura.gov.br/sislegis/action/detalhaAto.do?method=visualizarAtoPortalMapa&chave=377658578. Acesso em: 22/07/2024.

53. | BRASIL. Ministério da Agricultura e Pecuária – MAPA. Gabinete do Ministro. Diário oficial da união. **Diário Oficial da União**: seção 1, edição 186, Brasília, DF, p. 47. Instrução Normativa nº 50, de 24 de setembro de 2013. Altera a lista de doenças passíveis da aplicação de medidas de defesa sanitária animal, previstas no art. 61 do Regulamento do Serviço de Defesa Sanitária Animal. Disponível em: https://www.in.gov.br/materia/-/asset_publisher/Kujrw0TZC2Mb/content/id/31061237/do1-2013-09-25-instrucao-normativa-n-50-de-24-de-setembro-de-2013-31061233. Acesso em: 18/07/2024.

54. | BRASIL. Ministério da Agricultura e Pecuária – MAPA. Instrução Normativa nº 5, de 1º de março de 2002. **Diário Oficial da União**: seção 1, Brasília, DF, p. 4. Aprova as Normas Técnicas para o controle da raiva dos herbívoros domésticos. Disponível em: https://www.gov.br/agricultura/pt-br/assuntos/sanidade-animal-e-vegetal/saude-animal/programa-nacional-de-prevencao-e-vigilancia-da-encefalopatia-espongiforme-bovina-pneeb/principais-legislacoes-do-pneeb/instrucao-normativa-mapa-no-5-de-1o-03.2002/view. Acesso em: 18/07/2024.

55. | BRASIL. Ministério da Agricultura e Pecuária – MAPA. Secretária de Defesa Agropecuária. Instrução Normativa nº 10, de 3 de março de 2017. **Diário Oficial da União**: seção 1, Brasília, DF, p. 4-8. Institui o grau de risco para as doenças brucelose e tuberculose, assim como a definição de procedimentos de defesa sanitária animal a serem adotados de acordo com a classificação, na forma desta Instrução Normativa. Disponível em: https://www.agricultura.rs.gov.br/upload/arquivos/201709/01101230-pnce-bt-in-10-2017.pdf. Acesso em: 18/07/2024.

56. | BRASIL. Ministério da Ciência, Tecnologia, Inovações e Comunicações – MCTIC. **Diário Oficial da União**: seção 1, Brasília, DF, p. 5. Comissão Nacional de energia nuclear – CNEN. Resolução nº 164, de 07 de março de 2014. Dispõe sobre a alteração do item 5.4.3.1 da Norma CNEN NN 3.01 Diretrizes Básicas de Proteção Radiológica, que define a otimização médica da proteção radiológica aplicável à área de medicina nuclear. Disponível em: https://www.gov.br/cnen/pt-br/acesso-a-informacao/atos-normativos-cnen/comissao_deliberativa/resolucoes/2014/rs_cnencd_164_2014.pdf. Acesso em: 18/07/2024.

57. | BRASIL. Ministério da Economia – ME. **Diário Oficial da União**: seção 1, Brasília, DF, p. 4. Secretaria Especial da Fazenda. Secretaria de Orçamento Federal. Diário oficial da união. Instrução Normativa nº 41, de 29 de maio de 2020. Dispõe sobre orientações técnicas para a identificação das autorizações de despesas relacionadas ao enfrentamento de calamidade pública nacional decorrente de pandemia, e de seus efeitos sociais e econômicos, de que trata o Decreto nº 10.360, de 21 de maio de 2020. Disponível em: https://pesquisa.in.gov.br/imprensa/jsp/visualiza/index.jsp?data=22/05/2020&jornal=515&pagina=4&totalArquivos=210. Acesso em: 18/07/2024.

58. | BRASIL. Ministério da Infraestrutura – MINFRA. Conselho Nacional de Trânsito – CONTRAN. **Diário Oficial da União**: seção 1, Brasília, DF, p. 99-100. Resolução nº 791, de 18 de junho de 2020. Consolida as normas sobre o transporte de animais de produção, de interesse econômico, de esporte, de lazer ou de exposição. Diário Oficial da União. Disponível em: https://www.gov.br/transportes/pt-br/assuntos/transito/conteudo-contran/resolucoes/resolucao791-2020.pdf. Acesso em: 19/07/2024.

59. | BRASIL. Ministério da Integração e do Desenvolvimento Regional – MIDR. Secretaria de Proteção e Defesa Civil. Universidade Federal de Santa Catarina – UFSC. Centro de Estudos e Pesquisas em Engenharia e Defesa Civil – CEPED. **Atlas Digital de Desastres no Brasil**. Brasília: MIDR, 2023. Disponível em: https://atlasdigital.mdr.gov.br/. Acesso em: 19/07/2024.

60. | BRASIL. Ministério da Integração e do Desenvolvimento Regional. Secretaria de Proteção e Defesa Civil. Universidade Federal de Santa Catarina – UFSC. Centro de Estudos e Pesquisas em Engenharia e Defesa Civil – CEPED. **Política Nacional de Defesa Civil. Brasília**: PNDEC, 2012. Disponível em: https://www.ceped.ufsc.br/wp-content/uploads/2014/09/Pol%C3%ADtica-Nacional-de-Defesa-Civil.pdf. Acesso em: 22/07/2024.

61. | BRASIL. Ministério da Pesca e Aquicultura – MPA/CGSAP/DEMOC/SEMOC. **Manual de coleta e remessa de amostras para diagnóstico de enfermidades de animais aquáticos na rede nacional de laboratórios do Ministério da Pesca e Aquicultura – RENAQUA**. Coordenação-Geral de Sanidade Pesqueira. Departamento de Monitoramento e Controle. Secretaria de Monitoramento e Controle da Pesca e Aquicultura. Brasília, 1a edição, 67 p, 2013. Disponível em: https://www.idaron.ro.gov.br/wp-content/uploads/2021/07/manual_coleta_remessa.pdf. Acesso em: 23/07/2024.

62. | BRASIL. Ministério da Saúde – MS. Agência Nacional de Vigilância Sanitária – ANVISA. **Diário Oficial da União**: seção 1, Brasília, DF, 61 p. Resolução RDC nº 222, de 28 de março de 2018. Regulamenta as Boas Práticas de Gerenciamento dos Resíduos de Serviços de Saúde e dá outras providências. Disponível em: https://antigo.anvisa.gov.br/documents/33852/271855/RDC+222+de+Mar%C3%A7o+de+2018+COMENTADA/edd85795-17a2-4e1e-99ac-df6bad1e00ce?version=1.0 https://antigo.anvisa.gov.br/documents/33852/271855/RDC+222+de+Mar%C3%A7o+de+2018+COMENTADA/edd85795-17a2-4e1e-99ac-df6bad1e00ce?version=1.0. Acesso em: 14/08/2024.

63. | BRASIL. Ministério da Saúde – MS. Agência Nacional de Vigilância Sanitária – ANVISA. **Diário Oficial da União**: seção 1, Brasília, DF, 7 p. Resolução R nº 20, de 5 de maio de 2011. Dispõe sobre o controle de medicamentos à base de substâncias classificadas como antimicrobianos, de uso sob prescrição, isoladas ou em associação. Disponível em: https://www.gov.br/anvisa/pt-br/assuntos/fiscalizacao-e-monitoramento/sngpc/legislacao/arquivos/9170json-file-1. Acesso em: 14/08/2024.

64. | BRASIL. Ministério da Saúde – MS. Agência Nacional de Vigilância Sanitária – ANVISA. **Diário Oficial da União**: seção 1, Brasília, DF, p. 92. Diretoria Colegiada. Resolução CNE nº 330, de 20 de dezembro de 2019. Estabelece os requisitos sanitários para a organização e o funcionamento de serviços de radiologia diagnóstica ou intervencionista. Disponível em: https://cvs.saude.sp.gov.br/zip/U_RS-MS-ANVISA-RDC-330_201219.pdf. Acesso em: 19/07/2024.

65. | BRASIL. Ministério da Saúde – MS. Agência Nacional de Vigilância Sanitária – ANVISA. **Referência técnica para o funcionamento dos serviços veterinários**. Brasília, DF, 47 p. Agência Nacional de Vigilância Sanitária. Disponível em: https://www.crmv-pr.org.br/uploads/pagina/arquivos/Referencia_tecnica_funcionamento_servicos_veterinarios.pdf. Acesso em: 12/08/2024.

66. | BRASIL. Ministério da Saúde – MS. Gabinete do Ministro. Portaria nº 1.138, de 23 de maio de 2014. Define as ações e os serviços de saúde voltados para vigilância, prevenção e controle de zoonoses e de acidentes causados por animais peçonhentos e venenosos, de relevância para a saúde pública. Disponível em: https://bvsms.saude.gov.br/bvs/saudelegis/gm/2014/prt1138_23_05_2014.htmlhttps://bvsms.saude.gov.br/bvs/saudelegis/gm/2014/prt1138_23_05_2014.html. Acesso em: 12/08/2024.

67. | BRASIL. Ministério da Saúde – MS. Gabinete do Ministro. Portaria nº 5, de 28 de setembro de 2017. **Diário Oficial da União**: seção 1, Brasília, DF de 03 de outubro de 2017, p. 360-568. Consolidação das normas sobre as ações e os serviços de saúde do Sistema Único de Saúde. Disponível em: https://www.gov.br/saude/pt-br/composicao/sectics/pnpmf/orientacao-ao-prescritor/Publicacoes/portaria-de-consolidacao-no-5-de-28-de-setembro-de-2017.pdf/view. Acesso em: 14/08/2024.

68. | BRASIL. Ministério da Saúde – MS. Gabinete do Ministro. Portaria nº 1.929, de 11 de agosto de 2021. **Diário Oficial da União**: seção 1, Brasília, DF, p. 66. Altera a Portaria de Consolidação GM/MS nº 5 de 28 de setembro de 2017, para instituir Grupo Técnico de Resistência Antimicrobiana (GTAMR) de acompanhamento da implementação do Plano de Ação Nacional de Prevenção e Controle da Resistência aos Antimicrobianos, no âmbito do Ministério da Saúde. Disponível em: https://ses.sp.bvs.br/wp-content/uploads/2021/08/U_PT-MS-GM-1929_110821.pdf. Acesso em: 14/08/2024.

69. | BRASIL. Ministério da Saúde – MS. Gabinete do Ministro. Portaria nº 888, de 4 de maio de 2021. **Diário Oficial da União**: seção 1, Brasília, DF, p. 127. Altera o Anexo XX da Portaria de Consolidação GM/MS nº 5, de 28 de setembro de 2017, para dispor sobre os procedimentos de controle e de vigilância da qualidade da água para consumo humano e seu padrão de potabilidade. Disponível em: https://www.in.gov.br/en/web/dou/-/portaria-gm/ms-n-888-de-4-de-maio-de-2021-318461562. Acesso em: 14/08/2024.

70. | BRASIL. Ministério do Meio Ambiente e Mudança do Clima – MMA. Conselho Nacional do Meio Ambiente – CONAMA. **Diário Oficial da União**: seção 1, Brasília, DF, p.89. Resolução nº 430, 13 de maio de 2011. Dispõe sobre as condições e padrões de lançamento de efluentes, complementa e altera a Resolução no 357, de 17 de março de 2005, do Conselho Nacional do Meio Ambiente-CONAMA. Disponível em: https://www.saude.mg.gov.br/images/documentos/Resolucao%20CONAMA%20430%20de%2013%20de%20maio%20de%202011.pdfhttps://www.saude.mg.gov.br/images/documentos/Resolucao%20CONAMA%20430%20de%2013%20de%20maio%20de%202011.pdf. Acesso em: 14/08/2024.

71. | BRASIL. Ministério do Meio Ambiente e Mudança do Clima – MMA. Conselho Nacional do Meio Ambiente – CONAMA. **Diário Oficial da União**: seção 1, Brasília, DF, p. 2548-2549. Resolução nº 1, de 23 de janeiro de 1986. Dispõe sobre critérios básicos e diretrizes gerais para a avaliação de impacto ambiental. Disponível em: https://conama.mma.gov.br/?option=com_sisconama&task=arquivo.download&id=745. Acesso em: 22/07/2024.

72. | BRASIL. Ministério do Meio Ambiente e Mudança do Clima – MMA. Conselho Nacional do Meio Ambiente – CONAMA. **Diário Oficial da União**: seção 1, Brasília, DF, p. 10845. Resolução nº 1, de 13 de junho de 1988. Dispõe sobre o Cadastro Técnico Federal de atividades e instrumentos de defesa ambiental. Disponível em: http://www.areaseg.com/conama/1988/001-1988.pdf. Acesso em: 22/07/2024.

73. | BRASIL. Ministério do Meio Ambiente e Mudança do Clima – MMA. Conselho Nacional do Meio Ambiente – CONAMA. **Diário Oficial da União**: seção 2, Brasília, DF, p. 18046. Resolução nº 2, de 18 de abril de 1996. Dispõe sobre a implantação de Unidades de Conservação como critério compensatório para reparação de danos ambientais. Disponível em: https://www.ibama.gov.br/sophia/cnia/legislacao/MMA/RE0002-180496.PDF. Acesso em: 22/07/2024.

74. | BRASIL. Ministério do Meio Ambiente e Mudança do Clima – MMA. Conselho Nacional do Meio Ambiente – CONAMA. **Diário Oficial da União**: seção 1, Brasília, DF, p. 30841-30843. Resolução nº 237, de 19 de dezembro de 1997. Considerando a necessidade de revisão dos procedimentos e critérios utilizados no licenciamento ambiental, de forma a efetivar a utilização do sistema de licenciamento como instrumento de gestão ambiental, instituído pela Política Nacional do Meio Ambiente. Disponível em: https://conama.mma.gov.br/?option=com_sisconama&task=arquivo.download&id=237. Acesso em: 22/07/2024.

75. | BRASIL. Ministério do Meio Ambiente e Mudança do Clima – MMA. Conselho Nacional do Meio Ambiente – CONAMA. **Diário Oficial da União**: seção 1, Brasília, DF, p. 63-65. Resolução nº 358, de 29 de abril de 2005. Dispõe sobre o tratamento e a disposição final dos resíduos dos serviços de saúde e dá outras providências. Disponível em: https://www.ibama.gov.br/sophia/cnia/legislacao/MMA/RE0358-290405.PDF. Acesso em: 22/07/2024.

76. | BRASIL. Ministério do Meio Ambiente e Mudança do Clima – MMA. Conselho Nacional do Meio Ambiente – CONAMA. **Diário Oficial da União**: seção 1, Brasília, DF, p. 81-84. Resolução nº 420, de 30 de dezembro de 2009. Dispõe sobre critérios e valores orientadores de qualidade do solo quanto à presença de substâncias químicas e estabelece diretrizes para o gerenciamento ambiental de áreas contaminadas por essas substâncias em decorrência de atividades antrópicas. Disponível em: https://www.ibama.gov.br/sophia/cnia/legislacao/CONAMA/REA0420-281209.PDF. Acesso em: 22/07/2024.

77. | BRASIL. Ministério do Meio Ambiente e Mudança do Clima – MMA. Gabinete do Ministro. **Diário Oficial da União**: seção 1, Brasília, DF, p. 74. Portaria nº 148, de 7 de junho de 2022. Altera os Anexos da Portaria nº 443, de 17 de dezembro de 2014, da Portaria nº 444, de 17 de dezembro de 2014, e da Portaria nº 445, de 17 de dezembro de 2014, referentes à atualização da Lista Nacional de Espécies Ameaçadas de Extinção. Disponível em: https://www.in.gov.br/en/web/dou/-/portaria-mma-n-148-de-7-de-junho-de-2022-406272733. Acesso em: 22/07/2024.

78. | BRASIL. Ministério do Meio Ambiente e Mudança do Clima – MMA. Instituto Brasileiro do Meio Ambiente e Recursos Naturais Renováveis – IBAMA. **Diário Oficial da União**: seção 1, Brasília, DF, 27. p. Instrução Normativa nº 5 de 13 de maio de 2021. Dispõe sobre as diretrizes, prazos e os procedimentos para a operacionalização dos Centros de Triagem de Animais Silvestres (CETAS) do Ibama, bem como para a destinação de animais silvestres apreendidos, resgatados ou entregues espontaneamente a esses centros. Disponível em: https://www.icmbio.gov.br/cepsul/images/stories/legislacao/Instrucao_normativa/2021/IN_ibama_05_2021_diretrizes_operacionalizacao_cetas.pdf. Acesso em: 22/07/2024.

79. | BRASIL. Ministério do Meio Ambiente e Mudança do Clima – MMA. Instituto Brasileiro do Meio Ambiente e dos Recursos Naturais Renováveis – IBAMA. **Diário Oficial da União**: seção 1, Brasília, DF, 66. p. Instrução Normativa nº 7, de 30 de abril de 2015. Institui e normatiza as categorias de uso e manejo da fauna silvestre em cativeiro, e define, no âmbito do IBAMA, os procedimentos autorizativos para as categorias estabelecidas. Disponível em: https://www.icmbio.gov.br/cepsul/images/stories/legislacao/Instrucao_normativa/2015/in_ibama_07_2015_institui_categorias_uso_manejo_fauna_silvestre_cativeiro.pdf. Acesso em: 22/07/2024.

80. | BRASIL. Ministério do Meio Ambiente e Mudança do Clima – MMA. Instituto Brasileiro do Meio Ambiente e dos Recursos renováveis – IBAMA. **Diário Oficial da União**: seção 1, Brasília, DF, p. 115. Instrução Normativa nº 23 de 31 de dezembro de 2014. Esta Instrução Normativa – IN define as diretrizes e os procedimentos para a destinação de animais silvestres apreendidos, resgatados por autoridade competente ou entregues voluntariamente pela população, bem como para o funcionamento dos Centros de Triagem de Animais Silvestres do IBAMA – CETAS. Disponível em: https://www.in.gov.br/materia/-/asset_publisher/Kujrw0TZC2Mb/content/id/32094529/do1-2015-01-02-instrucao-normativa-n-23-de-31-de-dezembro-de-2014-32094512. Acesso em: 22/07/2024.

81. | BRASIL. Ministério do Meio Ambiente e Mudança do Clima – MMA. Instituto Brasileiro do Meio Ambiente e dos recursos naturais renováveis – IBAMA. **Diário Oficial da União**: seção 1, Brasília, DF, p. 71. Instrução normativa nº 8, de 14 de julho de 2017. Dispõe sobre estabelecer os procedimentos para a solicitação e emissão de Autorização para Captura, Coleta e Transporte de Material Biológico (Abio) no âmbito dos processos de licenciamento ambiental federal. Disponível em: https://crmvba.org.br/wp-content/uploads/2018/02/crmvba.org.br-medicos-veterinarios-discutem-legislacao-dos-trabalhos-de-licenciamento-ambiental-in-08-2017-meio-ambiente.pdf. Acesso em: 22/07/2024.

82. | BRASIL. Ministério do Meio Ambiente e Mudança do Clima – MMA. Instituto Brasileiro do Meio Ambiente e dos Recursos Naturais Renováveis – IBAMA. **Diário Oficial da União**: seção 1, Brasília, DF, p. 114. Portaria Normativa nº 10 de 22 de maio de 2009. Restringe a aplicação da Instrução Normativa nº 146, de 10 janeiro de 2007 quanto ao licenciamento de empreendimentos de aproveitamento hidrelétrico. Disponível em: https://urbanismoemeioambiente.fortaleza.ce.gov.br/images/urbanismo-e-meio-ambiente/legislacao-especifica/portaria_normativa_n%C2%BA_10_de_22_de_maio_de_2009_-_ibama.pdf. Acesso em: 22/07/2024.

83. | BRASIL. Ministério do Meio Ambiente e Mudança do Clima – MMA. Instituto Brasileiro do Meio Ambiente e dos Recursos Naturais Renováveis – IBAMA. **Diário Oficial da União**: seção 1, Brasília, DF, 22. p. Instrução Normativa nº 10, de 27 de maio de 2013. Regulamenta o Cadastro Técnico Federal de atividades e instrumentos de defesa animal – CTF/AIDA. Disponível em: https://www.icmbio.gov.br/cepsul/images/stories/legislacao/Instrucao_normativa/2013/in_ibama_10_2013_regulamentar_cadastrot%C3%A9cnicofederal_atividades_instrumentos_defesaambiental_ctf_aida.pdf. Acesso em: 22/07/2024.

84. | BRASIL. Ministério do Meio Ambiente e Mudança do Clima – MMA. Instituto Brasileiro do Meio Ambiente e dos Recursos Naturais Renováveis – IBAMA. **Diário Oficial da União**: seção 1, Brasília, DF, p. 29. Instrução Normativa nº 12, de 25 de março de 2019. Institui o Sistema Integrado de Manejo de Fauna – SIMAF, como sistema eletrônico para recebimento de declarações e relatórios de manejo da espécie exótica invasora javali – Sus scrofa. Disponível em: https://www.in.gov.br/materia/-/asset_publisher/Kujrw0TZC2Mb/content/id/70006375. Acesso em: 22/07/2024.

85. | BRASIL. Ministério do Meio Ambiente e Mudança do Clima – MMA. Instituto Brasileiro do Meio Ambiente e dos Recursos Naturais Renováveis – IBAMA. **Diário Oficial da União**: seção 1, Brasília, DF, 13. p. Portaria nº 145, de 29 de outubro de 1998. Estabelece normas para a introdução, reintrodução e transferência de peixes, crustáceos, moluscos, e macrófitas aquáticas para fins de aquicultura, excluindo-se as espécies animais ornamentais. Disponível em: https://www.icmbio.gov.br/cepsul/images/stories/legislacao/Portaria/1998/p_ibama_145_n_1998_introducaoespeciesnaaquicultura_revoga_p_ibama_119_1997_alterada_p_ibama_105_n_1999_27_2003_04_2005.pdf. Acesso em: 22/07/2024.

86. | BRASIL. Ministério do Meio Ambiente e Mudança do Clima – MMA. Instituto Brasileiro do Meio Ambiente e dos Recursos Naturais Renováveis – IBAMA. **Diário Oficial da União**: seção 1, Brasília, DF, 8. p. Instrução Normativa nº 146, de 10 de janeiro de 2007. Estabelece os critérios para procedimentos relativos ao manejo de fauna silvestre (levantamento, monitoramento, salvamento, resgate e destinação) em áreas de influência de empreendimentos e atividades consideradas efetiva ou potencialmente causadoras de impactos à fauna, sujeitas ao licenciamento ambiental. Disponível em: https://www.ipaam.am.gov.br/wp-content/uploads/2021/01/IN-146-07-IBAMA-Fauna.pdf. Acesso em: 22/07/2024.

87. | BRASIL. Ministério do Meio Ambiente e Mudança do Clima – MMA. Instituto Brasileiro Do Meio Ambiente e dos Recursos Naturais Renováveis – IBAMA. **Diário Oficial da União**: seção 1, Brasília, DF, 30. p. Instrução normativa nº 169 de 20 de fevereiro de 2008. Institui e normatiza as categorias de uso e manejo da fauna silvestre em cativeiro em território brasileiro e dá outras providências (Revogado pela Instrução Normativa IBAMA nº 7/2015). Disponível em: https://www.gov.br/ibama/pt-br/assuntos/biodiversidade/fauna-silvestre/arquivos/faunasilvestre/2008_ibama_in_169-2008_anexo.pdf. Acesso em: 22/07/2024.

88. | BRASIL. Ministério do Meio Ambiente e Mudança do Clima – MMA. Instituto Brasileiro do Meio Ambiente e dos Recursos Naturais Renováveis – IBAMA. **Diário Oficial da União**: seção 1, Brasília, DF, p. 60-63. Instrução Normativa nº 179, de 25 de junho de 2008. Estabelece os critérios para a destinação da fauna apreendida. Disponível em: https://www.icmbio.gov.br/cma/images/stories/Legislacao/Instru%C3%A7%C3%B5es_normativas/Instrucao_normativa_179_25062008_-_Normatiza%C3%A7%C3%A3o_do_CETAS.pdf. Acesso em: 22/07/2024.

89. | BRASIL. Ministério do Meio Ambiente e Mudança do Clima – MMA. Instituto Brasileiro do Meio Ambiente e dos Recursos Naturais Renováveis – IBAMA. **Diário Oficial da União**: seção 1, Brasília, DF, p. 74-77. Portaria nº 93, de 07 de julho de 1998. Dispõe sobre a importação e exportação de fauna silvestre (destaque para o Anexo I – Listagem de fauna considerada doméstica para fins de operacionalização do IBAMA. Disponível em: https://www.gov.br/ibama/pt-br/assuntos/biodiversidade/cites-e-comercio-exterior/arquivos/legislacao/1998_ibama_portaria_93_1998_importacao_exportacao_fauna_silvestre__lista_fauna_domestica.pdf. Acesso em: 22/07/2024.

90. | BRASIL. Ministério do Meio Ambiente e Mudança do Clima – MMA. Instituto Brasileiro do Meio Ambiente e dos Recursos Naturais Renováveis – IBAMA. **Diário Oficial da União**: seção 1, Brasília, DF, p. 50. Portaria nº 2.489, de 9 de julho de 2019. Apresenta a lista de espécies isentas de controle para fins de operacionalização do IBAMA. Disponível em: https://www.in.gov.br/web/dou/-/portaria-n-2489-de-9-de-julho-de-2019-191677320. Acesso em: 22/07/2024.

91. | BRASIL. Ministério do Meio Ambiente e Mudança do Clima – MMA. Instituto Chico Mendes de Conservação da Biodiversidade – ICMBIO. **Diário Oficial da União**: seção 1, Brasília, DF, p. 51. Instrução Normativa nº 5, de 28 de junho de 2021. Estabelece os procedimentos para criação e implementação dos Programas de Manejo Populacional de Espécies Ameaçadas da Fauna Brasileira. Disponível em: https://www.gov.br/icmbio/pt-br/assuntos/programas-e-projetos/programa-de-manejo-populacional/arquivos/in_5_de_28_de_junho_de_2021.pdf. Acesso em: 22/07/2024.

92. | BRASIL. Ministério do Trabalho e do Emprego – MTE. **Diário Oficial da União**: seção 1, Brasília, DF, 2. p. Portaria nº 485, de 11 de novembro de 2005. Aprova a Norma Regulamentadora nº 32 (Segurança e Saúde no Trabalho em Estabelecimentos de Saúde). Disponível em: https://www.camara.leg.br/proposicoesWeb/prop_mostrarintegra?codteor=726447. Acesso em: 22/07/2024.

93. | BRASIL. Resolução CONAMA nº 01 de 17 de fevereiro de 1986. **Diário Oficial da União**: seção 1, Brasília, DF, p. 2548-2549. Dispõe sobre critérios básicos e diretrizes gerais para a avaliação de impacto ambiental. Disponível em: https://conama.mma.gov.br/?option=com_sisconama&task=arquivo.download&id=745. Acesso em: 18/07/2024.

94. | Broom, D. M. A History of Animal Welfare Science. **Acta Biotheor**. 59, 121–137, 2011. DOI: 10.1007/s10441-011-9123-3

95. | Carneiro, J. M. E. A.; Botelho, D. G.; Resgala Júnior, R. M. Princípio da Dignidade Animal e o Dever Fundamental de Proteção. **Revista Ibero-Americana de Humanidades, Ciências e Educação**. 9, 2098–2109, 2023. DOI: https://doi.org/10.51891/rease.v9i9.11339.

96. | Carneiro, L. A.; Pettan-Brewer, C. *One Health*: Conceito, história e questões relacionadas – revisão e reflexão. In: Pesquisa em Saúde & Ambiente na Amazônia: perspectivas para sustentabilidade humana e ambiental na região. **Editora Científica Digital**. p. 219–240, 2021. DOI: 10.37885/978-65-89826-36-1

97. | Carver, L. F. When pets are family, the benefits extend into Society. **The conversation**. Queen's University, Ontario, 2019. Disponível em: https://theconversation.com/when-pets-are-family-the-benefits-extend-into-society-109179. Acesso em 22/02/2022.

98. | Clima de eleição. **GT Clima da Frente Ambientalista**. 2024. Disponível em: https://linktr.ee/climadeeleicao. Acesso em: 23/07/2024.

99. | Defesa Civil de Porto Alegre – CDC. **Plano de Contingências de Proteção e Defesa Civil**. Processo Sistema Eletrônico Informação - SEI nº 22.0.000036187-2. Porto Alegre/RS, 2022. Disponível em: https://dopaonlineupload.procempa.com.br/dopaonlineupload/4419_ce_366883_2.pdf. Acesso em: 23/07/2024.

100. | Defesa Civil de Porto Alegre – CDC. Plano de Contingências de Proteção e Defesa Civil -: Processo Sistema Eletrônico Informação - SEI nº 22.0.000036187-2. Porto Alegre/RS, 2022. Disponível em: https://dopaonlineupload.procempa.com.br/dopaonlineupload/4419_ce_366883_2.pdf. Acesso em: 23/07/2024.

101. | Domingos, R. O. C.; Souza, E. L. D. O critério de senciência dos animais humanos e não humanos e sua condição como "sujeito de direito". In: Anais do IV Colóquio Estadual de Pesquisa Multidisciplinar do II Congresso Nacional de Pesquisa Multidisciplinar. **Unifimes**. Centro Universitário de Mineiros, 2019. Disponível em: https://publicacoes.unifimes.edu.br/index.php/coloquio/article/view/624. Acesso em: 23/07/2024.

102. | Foley, J.; Bannasch, M. Infectious diseases of dogs and cats. In Shelter Medicine for Veterinarians and Staf (ed. by Miller, L.; S Zawitowski). **Blackwell Publishing Ltd**. Ames, Iowa, p. 235–284, 2004.

103. | Galdioli, L.; Garcia R. **Medicina de Abrigos**: Princípios e Diretrizes. 1ª ed. Curitiba: Instituto de Medicina Veterinária do Coletivo. 2022. 1140 p.

104. | Galdioli, L.; Polato, H. Z.; Mausson, L. F. T.; Ferraz, C. P.; Garcia, R. C. M. **Guia introdutório de bem-estar e comportamento de cães e gatos para gestores e funcionários de abrigos**. 1ª ed. Paraná: Universidade Federal do Paraná, 2021. Disponível em: https://www.premierpet.com.br/wp-content/uploads/2023/02/GUIA-INTRODUTORIO-DE-BEM-ESTAR-E-COMPORTAMENTO-DE-CAES-E-GATOS-PARA-GESTORES-E-FUNCIONARIOS-DE-ABRIGOS-DIGITAL.pdf. Acesso em: 09/08/2024.

105. | Garcia, R. C. M.; Calderón, N.; Brandespim, D. F. **Medicina Veterinária do Coletivo: Fundamentos e Práticas**. São Paulo: Integrativa Vet, 2019.

106. | Gebara, R. R. Registro e identificação. In: Garcia, R. C. M.; Calderón, N.; Brandespim, D. F. **Medicina Veterinária do Coletivo: Fundamentos e Práticas**. São Paulo: Integrativa Vet, 2019. Sessão 2. Item 2.5.5 p. 209–217.

107. | Gebara, R.; Galdioli, L.; Bastos, P.; Bastos, A. L.; Garcia, R.; Bonela, L.; Rocha, Y. S. G.; Zacchi, F. **Manual de Boas-práticas no Abrigamento de Cães e Gatos em Situações de Desastres**. 1ª ed. Instituto de Medicina Veterinária do Coletivo. 2024. Disponível em: https://institutomvc.org.br/site/index.php/manual/. Acesso em: 09/08/2024.

108. | Genaro, G. Aplicação de conceitos básicos em etologia na clínica médica veterinária felina. **Revista de Educação Continuada em Medicina Veterinária e Zootecnia do CRMV-SP**, v. 11, n.1. p. 32–37. 2013. Disponível em: https://www.revistamvez-crmvsp.com.br/index.php/recmvz/issue/view/427/20. Acesso em: 09/08/2024.

109. | Global Veterinary Community. **Directrizes para Identificação Electrónica**. The world small animal veterinary association – WSAVA 2020. Disponível em: https://wsava.org/wp-content/uploads/2020/01/WSAVA-Microchip-Identification-Guidelines-Portuguese.pdf. Acesso em: 08/08/2024.

110. | Gomes, L. B.; Reis, S. T.; Atayde, I. B.; Bastos, A. L. F.; Miranda, C. M. S. **Plano Nacional de Contingência de Desastres em Massa Envolvendo Animais**. 1ª ed. Conselho Federal de Medicina Veterinária. 2020. Disponível em: https://crmvsp.gov.br/wp-content/uploads/2021/02/Plano-Nacional-de-Conting%C3%AAncia-de-Desastres-em-Massa-Envolvendo-Animais-CFMV.pdf. Acesso em: 09/08/2024.

111. | Gomes, N. S.C. Ética e Dignidade Animal: Uma Abordagem da Constituição Brasileira, da Lei De Crimes Contra a Natureza e do Decreto de Proteção aos Animais Sob a Ótica da Declaração Universal dos Direitos dos Animais. *In*: XIX Encontro Nacional do CONPEDI, 2010. Fortaleza, CE. **Anais**. Disponível em: http://www.publicadireito.com.br/conpedi/manaus/arquivos/anais/fortaleza/3375.pdf. Acessado em: 09/08/2024.

112. | Hodgson, K.; Barton, L.; Darling, M.; Antao, V.; Kim, F. A.; Monavvari, A. Pets' impact on your patients' health: leveraging benefits and mitigating risk. **The Journal of the American Board of Family Medicine**. v. 28, n.4, p. 526–534. 2015. DOI: https://doi.org/10.3122/jabfm.2015.04.140254. Disponível em: https://www.jabfm.org/content/jabfp/28/4/526.full.pdf. Acesso em: 09/08/2024.

113. | Hodgson, K.; Darling, M. Zooeyia: An essential component of "One Health". **Canadian Veterinary Journal**, v. 52, 189–191. 2011. Disponível em: https://www.ncbi.nlm.nih.gov/pmc/articles/PMC3022463/pdf/cvj_02_189.pdf. Acesso em: 09/08/2024.

114. | International Union for Conservation of Nature – IUCN. **Guidelines for reintroductions and other conservation translocations. version 1.0**. Gland, Switzerland: IUCN Species Survival Commission, 72 p., 2013. Disponível em: https://portals.iucn.org/library/efiles/documents/2013-009.pdf. Acesso em: 13/08/2024.

115. | Leira, M. H.; Santos, J. R. C.; Reghim, L. S.; Ciacci, L. S.; Barreto, B. B.; Cunha, L. T.; Amorim, D. R. C.; Belato, B. C. A.; Bueno, M. R.; Silva, D. A.; Dias, N. T. Zooantropologia: O novo conceito dentro do velho e bem-estar animal. **Pubvet**. v.11, n.8, p.754–760, 2017. DOI: http://dx.doi.org/10.22256/PUBVET.V11N8.754-760.

116. | Mellor, D. J., & Beausoleil, N. J. Extending the 'Five Domains' model for animal welfare assessment to incorporate positive welfare states. Herfordshire: **Universities Feeration for Animal Welfare**. v. 24, p. 241–253 2015. DOI: https://doi.org/10.7120/09627286.24.3.241. Disponível em: https://www.researchgate.net/profile/NgaioBeausoleil/publication/280631950_Extending_the_%27Five_Domains%27_model_for_animal_welfare_assessment_to_incorporate_positive_welfare_states/links/5b0641d1aca2725783d8a31c/Extending-the-Five-Domains-model-for-animal-welfare-assessment-to-incorporate-positive-welfare-states.pdf. Acesso em: 09/08/2024.

117. | Mellor, D. J.; Hunt, S.; Gusset, M. Caring for Wildlife: **The World Zoo and Aquarium Animal Welfare Strategy**. Gland, Switzerland: World Association of Zoos and Aquariums – WAZA, Executive Office, 87 p. 2015. Disponível em: <https://www.waza.org/wp-content/uploads/2019/03/WAZA-Animal-Welfare-Strategy-2015_Portuguese.pdf>. Acessado em: 09/08/2024.

118. | Minas Gerais. Conselho Estadual de Política Ambiental – COPAM. Deliberação Normativa COPAM nº 238, de 26 de agosto de 2020. **Diário do Executivo**: Belo Horizonte, MG, 7 p. Altera a Deliberação Normativa Copam nº 214, de 26 de abril de 2017, que estabelece as diretrizes para a elaboração e a execução dos Programas de Educação Ambiental no âmbito dos processos de licenciamento ambiental no Estado de Minas Gerais. Disponível em: processo-1370010021922202079 (meioambiente.mg.gov.br)processo-1370010021922202079 (meioambiente.mg.gov.br). Acesso em: 14/08/2024.

119. | Minas Gerais. Conselho Estadual de Recursos Hídricos – CERH e o Conselho Estadual de Política Ambiental – COPAM. Deliberação Normativa no 26, de 18 de dezembro de 2008. **Diário do Executivo**: Belo Horizonte, MG, 2 p. Dispõe sobre procedimentos gerais de natureza técnica e administrativa a serem observados no exame de pedidos de outorga para o lançamento de efluentes em corpos de água superficiais no domínio do Estado de Minas Gerais. Disponível em: https://www.cbhdoce.org.br/wp-content/uploads/2018/01/DN-CERH-26.pdf. Acesso em: 07/08/2024.

120. | Minas Gerais. Instituto Estadual de Florestas – IEF. Diretoria de Biodiversidade. Resolução Conjunta SEMAD/FEAM/IEF/IGAM nº 3.181 de 11 de novembro de 2022. **Termo de Referência para metodologia de análise de conectividade – Plano de Ação de Emergência (PAE)**. Governo do Estado de Minas. Disponível em: http://www.feam.br/gestao-de-barragens/-plano-de-acao-de-emergencia--pae. Acesso em: 12/08/2024.

121. | Minas Gerais. Instituto Estadual de Florestas – IEF. Diretoria de Biodiversidade. Resolução Conjunta SEMAD/FEAM/IEF/IGAM nº 3.181 de 11 de novembro de 2022. **Termo de Referência para caracterização de base quanto a fauna silvestre e serviços ecossistêmicos associados e impactos toxicológicos e ecotoxicológicos relacionados – Plano de Ação de Emergência (PAE)**. Governo do Estado de Minas. Disponível em: http://www.feam.br/gestao-de-barragens/-plano-de-acao-de-emergencia--pae. Acesso em: 12/08/2024.

122. | Minas Gerais. Instituto Estadual de Florestas – IEF. Resolução Conjunta SEMAD/FEAM/IEF/IGAM nº 3.181 de 11 de novembro de 2022. **Termo de referência da avaliação de impactos ambientais decorrente da ruptura sobre a fauna silvestre terrestre e biodiversidade aquática – Plano de Ação de Emergência (PAE)**. Governo do Estado de Minas. Disponível em: http://www.feam.br/gestao-de-barragens/-plano-de-acao-de-emergencia--pae. Acesso em: 12/08/2024.

123. | Minas Gerais. Instituto Estadual de Florestas – IEF. Resolução Conjunta SEMAD/FEAM/IEF/IGAM nº 3.181 de 11 de novembro de 2022. **Termo de referência do plano de resgate, salvamento e destinação de fauna silvestre de vida livre em caso de ruptura – Plano de Ação de Emergência (PAE)**. Governo do Estado de Minas. Disponível em: http://www.feam.br/gestao-de-barragens/-plano-de-acao-de-emergencia--pae. Acesso em: 12/08/2024.

124. | Minas Gerais. Instituto Estadual de Florestas – IEF. **Termo de referência para a caracterização de linha de base quanto a fauna silvestre e serviços ecossistêmicos associados – Plano de Ação de Emergência (PAE)**. Resolução Conjunta SEMAD/FEAM/IEF/IGAM nº 3.181 de 11 de novembro de 2022. Governo do Estado de Minas. Disponível em: http://www.feam.br/gestao-de-barragens/-plano-de-acao-de-emergencia--pae. Acesso em: 12/08/2024.

125. | Minas Gerais. Instituto Mineiro de Agropecuária – IMA. **Portaria nº 2.047, de 31 de março de 2021**. Estabelece diretrizes, exigências e ações para a apresentação e aprovação do Plano de Ação de Emergência – PAE, para as barragens abrangidas pela Lei nº 23.291, de 25 de janeiro de 2019, no âmbito das competências do Instituto Mineiro de Agropecuária definidas pelo Decreto nº 48.078, de 5 de novembro de 2020, e determina procedimentos a serem adotados pelos responsáveis destas barragens quando estiverem em situação de emergência. Governo do Estado de Minas. Disponível em: http://www.ima.mg.gov.br/files/1739/Ano-2021/18555/Portaria-n%C2%BA-2047,-de-31-de-marco-de-2021.pdf?preview=1. Acesso em: 12/08/2024.

126. | Minas Gerais. Lei Estadual nº 14.181, de 17 de janeiro de 2002. **Diário do Executivo**: Belo Horizonte, MG. Dispõe sobre a política de proteção à fauna e à flora aquáticas e de desenvolvimento da pesca e da aquicultura no Estado e dá outras providências. Governo do Estado de Minas. Instituto Mineiro de Agropecuária – IMA. Disponível em: https://www.almg.gov.br/legislacao-mineira/texto/LEI/14181/2002/?cons=1. Acesso em: 07/08/2024.

127. | Minas Gerais. Lei Estadual nº 14.309 de 19 de junho de 2002. **Diário do Executivo**: Belo Horizonte, MG. Dispõe sobre as políticas florestal e de proteção à biodiversidade no Estado. Governo do Estado de Minas. Disponível em: https://www.lexml.gov.br/urn/urn:lex:br;minas.gerais:estadual:lei:2002-06-19;14309. Acesso em: 07/08/2024.

128. | Minas Gerais. Lei Estadual nº 20.922 de 16 de outubro de 2013. **Diário do Executivo**: Belo Horizonte, MG. Dispõe sobre as políticas florestal e de proteção à biodiversidade no Estado. Governo do Estado de Minas. Disponível em: https://www.almg.gov.br/legislacao-mineira/texto/LEI/20922/2013/?cons=1. Acesso em: 07/08/2024.

129. | Minas Gerais. Lei Estadual nº 23.291, de 25 de fevereiro de 2019. **Diário do Executivo**: Belo Horizonte, MG. Institui a política estadual de segurança de barragens. Governo do Estado de Minas. Disponível em: https://www.almg.gov.br/legislacao-mineira/texto/LEI/20922/2013/?cons=1. Acesso em: 07/08/2024.

130. | Minas Gerais. Resolução Conjunta SEMAD/FEAM/IEF/IGAM nº 3.181 de 11 de novembro de 2022. **Diário do Executivo**: Belo Horizonte, MG, 11 p. Estabelece diretrizes para a apresentação do Plano de Ação de Emergência, para as barragens abrangidas pela Lei nº 23.291, de 25 de janeiro de 2019. Governo do Estado de Minas. Disponível em: https://www.siam.mg.gov.br/sla/download.pdf?idNorma=56489. Acesso em: 07/08/2024.

131. | Minas Gerais. Secretaria de Estado de Saúde – SES. **Resolução SES/MG nº 7.921 de 09 de dezembro de 2021**. Dispõe sobre o licenciamento sanitário de atividades veterinárias e aprova o Regulamento Técnico que estabelece as condições higiênico-sanitárias e as Boas Práticas de Funcionamento para os estabelecimentos que exercem atividades veterinárias que utilizam produtos de uso humano sujeitos ao controle sanitário do Sistema Nacional de Vigilância Sanitária e/ou realizam serviços de radiologia diagnóstica veterinária e dá outras providências. Governo do Estado de Minas. Disponível em: https://www.saude.mg.gov.br/images/documentos/RESOLU%C3%87%C3%83O%20SES%207921%20de%2009%20de%20Dezembro%20.pdf. Acesso em: 12/08/2024.

132. | Ministério da Agricultura e Pecuária – MAPA. **Aquicultura com sanidade. Programa Nacional de sanidade de animais aquáticos de cultivo. Manual orientado aos produtores**. 1ªed. Brasília: Ministério da Agricultura, Pecuária e Abastecimento. 2020. Processo Sistema Eletrônico Informação – SEI nº 21000.050206/2020-11/. Disponível em: https://www.gov.br/agricultura/pt-br/assuntos/sanidade-animal-e-vegetal/saude-animal/programas-de-saude-animal/arquivos-programas-sanitarios/AquiculturacomsanidadeManualorientadoaosprodutores.pdf. Acesso em: 09/08/2024.

133. | Ministério da Agricultura e Pecuária – MAPA. **Guia de Uso Racional de Antimicrobianos para Cães e Gatos**. 1ª ed. Brasília: Ministério de Agricultura, Pecuária e Abastecimento. 2022. Disponível em: https://www.gov.br/agricultura/pt-br/assuntos/insumos-agropecuarios/insumos-pecuarios/resistencia-aos-antimicrobianos/publicacoes/livroantimicrobianosv22.pdf. Acesso em: 09/08/2024.

134. | Ministério do Meio Ambiente e Mudança do Clima – MMA. **Convenção sobre Diversidade Biológica. Série Biodiversidade nº 1**. Disponível em: https://www.planalto.gov.br/ccivil_03/decreto/1998/anexos/and2519-98.pdf. Acesso em: 07/08/2024.

135. | Ministério Público de Minas Gerais – MPMG. Termo de Compromisso (TC) que celebram o Ministério Público de Minas Gerais assinado entre o Ministério Público de Minas Gerais e a VALE S.A, versando sobre medidas de proteção dos animais domésticos e silvestres que se encontrem nas manchas de inundação das estruturas da compromissária em Minas Gerais. Processo Sistema Eletrônico Informação - SEI nº 19.16.2372.0076772/2021-56. Belo Horizonte/MG, 2021.

136. | Ministério Público de Minas Gerais – MPMG. Termo de Compromisso (TC) que celebram o Ministério Público de Minas Gerais assinado entre o Ministério Público de Minas Gerais e a VALE S.A, versando sobre medidas de proteção dos animais domésticos e silvestres que se encontrem nas manchas de inundação das estruturas da compromissária em Minas Gerais. 2019.

137. | Mori, A. C. F. P.; Santos, E. P.; Veiga, M. C. M.; Lobão, R. A. **Plano de Gerenciamento de resíduos de serviços de saúde animal simplificado**. São Paulo: Conselho Regional de Medicina Veterinária do Estado de São Paulo. 2019. Disponível em: https://crmvsp.gov.br/3d-flip-book/plano-de-gerenciamento-de-residuos/. Acesso em: 08/08/2024.

138. | Movimento dos Atingidos por Barragens – MAB, **Crise climática aumenta os casos de rompimento por barragens**. 2024. Disponível em: https://mab.org.br/2024/05/03/nota-crise-climatica-aumenta-os-casos-de-rompimento-de-barragens/. Acesso em: 09/08/2024.

139. | Nagasawa, M.; Mitsui, S.; En, S.; Ohtani, N.; Ohta, M.; Sakuma, Y.; Onaka, T.; Mogi, K.; Kikusui, T. Social evolution. Oxytocin-gaze positive loop and the coevolution of human-dog bonds. **Science**. v. 348, n. 6232. p. 333- 336. 2015. DOI: 10.1126/science.1261022. Disponível em: https://www.science.org/doi/10.1126/science.1261022. Acesso em: 08/08/2024.

140. | Oliveira, K. S. **Manual de boas práticas na criação de animais de estimação: cães e gatos**. Goiânia: Cir Gráfica e Editora, 2019, 98 p. Disponível em: https://abinpet.org.br/wp-content/uploads/2021/04/Manual_de_Boas_Praticas_online.pdf. Acesso em: 08/08/2024.

141. | Oliveira, L. B. S.; Bastos, A. L. F.; Gomes, L. B.; Melo, M. D. V.; Reis, S. T. J.; Zacchi, F. R.; Snitovsky, L. G. **Manual de resgate e assistência à ictiofauna em situações de desastres ambientais**. Conselho Federal de Medicina Veterinária, 1a ed. 2020. 36 p. Disponível em: https://sis.univs.edu.br/uploads/12/Manual_de_resgate_e_assist__ncia____Ictiofauna_em_situa____es_de_desastres_compressed__1_.pdf. Acesso em: 08/08/2024.

142. | *One Health* High-Level Expert Panel – OHHLEP,; Adisasmito W. B.; Almuhairi S.; Behravesh C. B.; Bilivogui, P.; Bukachi S. A., *et al. One Health*: A new definition for a sustainable and healthy future. **PLoS Pathog**. v. 18, n. 6, e1010537. 2022. DOI: https://doi.org/10.1371/journal.ppat.1010537.Disponível em: https://journals.plos.org/plospathogens/article?id=10.1371/journal.ppat.1010537. Acesso em: 08/08/2024.

143. | Organização Mundial de Saúde Animal – OIE. **Global Animal Welfare Strategy**. Paris, França: OIE World Organization of Animal Health. 2017. Disponível em: https://www.woah.org/app/uploads/2021/03/en-oie--aw-strategy.pdf. Acesso em: 08/08/2024.

144. | Organização Mundial de Saúde Animal – OIE. **Guidelines on disaster management and risk reduction in relation to animal health and welfare and veterinary public health**. Paris, França: OIE. World organisation for animal health. Protecting animals, preserving our future. 2016. Disponível em: https://www.woah.org/app/uploads/2021/03/disastermanagement-ang.pdf. Acesso em: 08/08/2024.

145. | Paraíba. Lei Estadual nº 11.140, de 08 de junho de 2018. Diário Oficial: Paraíba, PB. **Institui o Código de Direito e Bem-estar animal do Estado da Paraíba**. Disponível em:http://sapl.al.pb.leg.br/sapl/sapl_documentos/norma_juridica/13183_texto_integral. Acesso em: 08/08/2024.

146. | Pavanelli, G. C.; Eiras, J. C.; Takemoto, R. M. **Doenças de Peixes. Profilaxia, Diagnóstico e Tratamento**. 3ª ed. Maringá: EDUEM. 2008.

147. | Pinto, A. J. W. **Manual Técnico de Socorrismo e Resgate Animal**. São Paulo: Pimenta Cultural. 2021

148. | Ramos, E. C. B.; Leite, H. P. S.; Simoes, K. C. C.; Viana, M. B.; Schneider M. **Meio Ambiente, Mudanças Climáticas e Rompimento de Barragens**. Brasília, DF: Observatório Parlamentar da Revisão Periódica Universal. 2021. Disponível em: https://bd.camara.leg.br/bd/items/66b500f6-c38d-47f3-8aa9-45933e3a7da7. Acesso em: 07/08/2024.

149. | Rault, JL.; Waiblinger, S.; Boivin, X.; Hemsworth, P. The Power of a positive human-animal relationship for animal welfare. **Front Vet Sci**. v.9, n.7, 13 p., 2020. DOI: https://doi.org/10.3389/fvets.2020.590867.

150. | Rocha, Y. S. G.; Galdioli, L. Métricas em Abrigos: Capacidade de Prover Cuidados. *In*: Galdioli L. e Garcia R. **Medicina de Abrigos: Princípios e Diretrizes**. 1ª ed. Curitiba: Instituto de Medicina Veterinária do Coletivo. 2022. Cap 5, p. 64-81.

151. | Rodrigues, N. T. D.; Rehbein, K. D. S.; Diotto, N. **Direito Animal**. Santa Maria: Arco Editores. 2022. Disponível em: https://www.researchgate.net/publication/359263704_DIREITO_ANIMAL. Acesso em: 07/08/2024.

152. | Silva, T. C. P. C. D.; Salomão, K.; Neves, A. M. A ética animal em Peter Singer e Tom Regan em virtude da problemática dos direitos universalizáveis dos animais. **Revista DIAPHONÍA**. Toledo, PR. v. 6, n. 1, p. 253–262. 2020. DOI: 10.48075/rd.v6i1.25085. Disponível em: https://e-revista.unioeste.br/index.php/diaphonia/article/view/25085. Acesso em: 07/08/2024.

153. | Souza, D. D. B. **Mudanças climáticas e um novo paradigma para as barragens**. Correio Braziliense. 2023. Disponível em: https://www.correiobraziliense.com.br/opiniao/2023/10/5129971-artigo-mudancas-climaticas-e-um-novo-paradigma-para-as-barragens.html. Acesso em: 07/08/2024.

154. | Souza. A. A. S. **Animais têm Direitos? Entendendo a Ética Animal à Luz da Filosofia**. Trabalho de Conclusão de Curso, Escola de Humanidades, Pontifícia Universidade Católica do Rio Grande do Sul, Porto Alegre, 2018. Disponível em: <file:///C:/Users/gislene.fournier/Downloads/ ANIMAIS_TEM_DIREITOS_Entendendo_a_Etica.pdf>. Acesso em: 01/07/2024.

155. | Teixeira, A. H. L. **Animal não humano: presente! Reflexões sobre a educação e a relação entre animais e não humanos**. 1 ed. Belo Horizonte: Ministério Público do Estado de Minas Gerais. 2020. Disponível em: https://ecologiaintegral.santoagostinho.com.br/wp-content/uploads/2022/09/Animal-nao-humano-presente-DIGITAL.pdf. Acesso em: 08/08/2024.

156. | Teixeira, A. H. L.; Ferreira, F. Q. C. **Cartilha Guarda responsável: que bicho é esse? Ensinando o respeito à vida e aos direitos dos animais**. Belo Horizonte: Ministério Público do Estado de Minas Gerais. 2013. Disponível em: https://www.mpdft.mp.br/portal/pdf/unidades/promotorias/prodema/defesa_animal/guardaresponsavel_MPMG.pdf. Acesso em: 08/08/2024.

157. | The United Nation for Disaster Risk Reduction – UNISDR. **Poverty & Death: disaster mortality 1996-2015**. Geneva, Switzerland. 2016. Disponível em: https://www.preventionweb.net/files/50589_creddisastermortalityallfinalpdf.pdf. Acesso em: 08/08/2024.

158. | Valencio, N. Processos de vulnerabilização envolvendo comunidades pesqueiras no Brasil: dos desastres recentes aos riscos relacionados às mudanças climáticas. **Revista de Educomunicação Ambiental**. São Carlos v. 3, n. 1, p. 106-120, 2013. DOI: 10.18248/1982-6389/edu-ambiental.v3n1p106-120. Disponível em: https://www.researchgate.net/publication/277884678_Processos_de_Vulnerabilizacao_Envolvendo_Comunidades_Pesqueiras_no_Brasil_Dos_Desastres_Recentes_aos_Riscos_Relacionados_as_Mudancas_Climaticas. Acesso em: 12/08/2024.

159. | Williamson, L. E.; Dell, C. A.; Chalmers, D.; Cruz, M.; De Groot, P. Strengthening zooeyia: Understanding the human-animal bond between veterans living with comorbid substance use and posttraumatic stress disorder and their service dogs. **Humam Animal Interaction Bulletin**. v.10, n.2, p.20–47, 2022. Disponível em: https://cabidigitallibrary.org by 34.100.51.37.

160. | World Health Organization – WHO; Food and Agriculture Organization of the United Nations – FAO; World Organization for Animal Health – WOAH. **Monitoring and evaluation of the global action plan on antimicrobial resistance: framework and recommended indicators**. 43 p.‚ 2019‚. Disponível em: https://iris.who.int/bitstream/handle/10665/325006/9789241515665-eng.pdf?sequence=1&isAllowed=y. Acesso em: 08/08/2024.

161. | World Organization for Animal Health – WOAH. **The OIE Terrestrial Animal Health Code (the Terrestrial Code)**. Online version, 29th Edition, v. 1, 2021. Disponível em: https://www.woah.org/en/what-we-do/standards/codes-and-manuals/previous-editions-of-the-terrestrial-code/. Acesso em 12/08/2024.

162. | Xie, Z.; Zhao, D.; Chen, B.; Wang, Y.; Ma, Y.; Shi, H.; Yang, Y.; Wang, Z.; Wang, L.Association between pet ownership and coronary artery disease in a Chinese population. Baltimore: **Medicine**. v. 96, n. 13, e6466. 2017. DOI: 10.1097/MD.0000000000006466. Disponível em: https://journals.lww.com/md-journal/fulltext/2017/03310/association_between_pet_ownership_and_coronary.28.aspx. Acesso em: 08/08/2024.

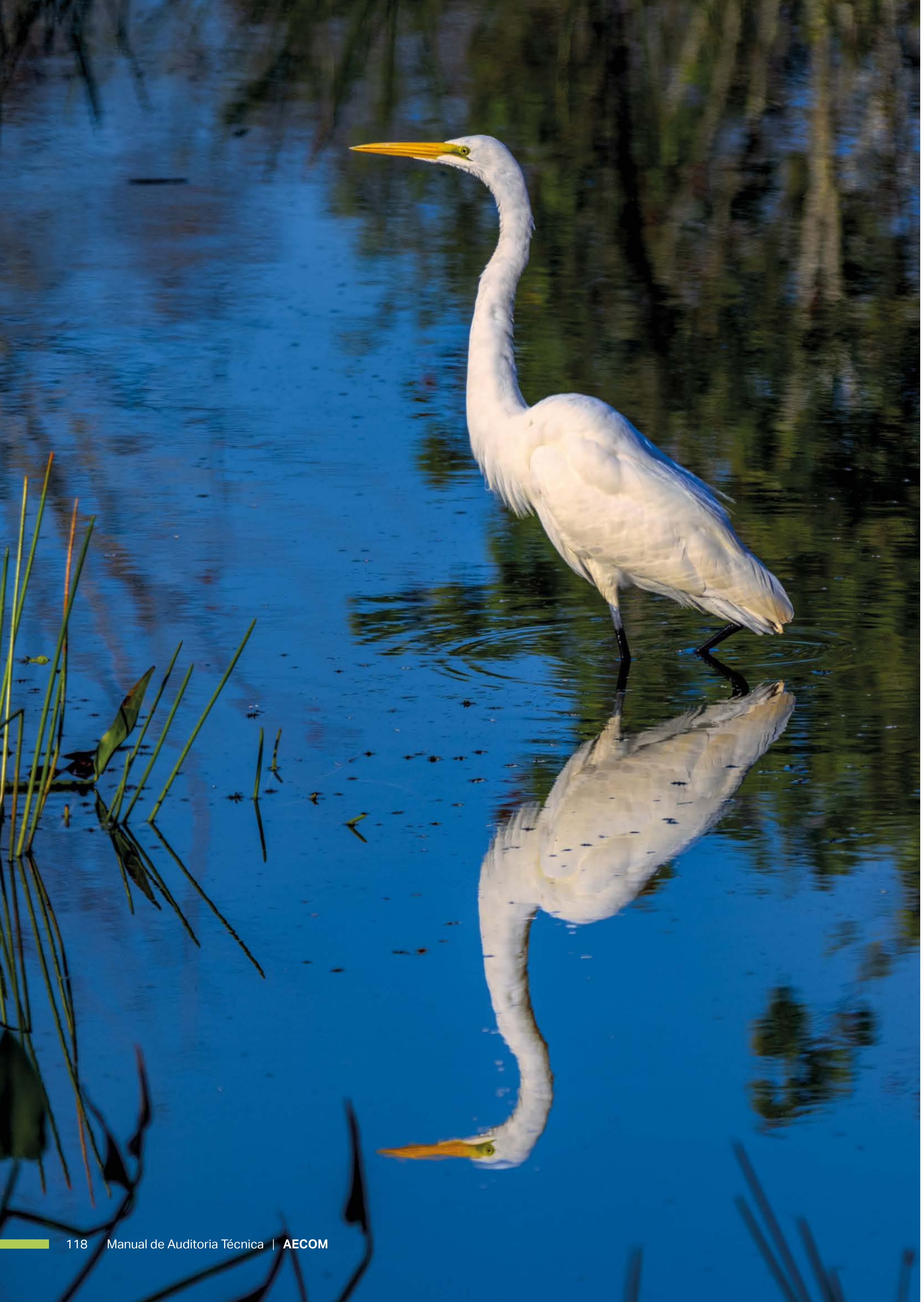

13. Glossário

Abrigo de fauna

Moradias temporárias ou permanentes, onde animais resgatados em situação emergencial, domésticos, de produção, exóticos e silvestres cativos ou silvestres resgatados em área de risco são acolhidos, medicados e mantidos em boas condições de bem-estar até serem destinados. Embora a grande maioria seja voltada para cães e gatos, há abrigos para as mais variadas espécies, incluindo cavalos, ruminantes, aves domésticas e animais silvestres (que devem estar de acordo com as legislações vigentes). Sinônimo para Instalação de fauna.

Abrigo multiespécie

Abrigos que contém mais de uma espécie.

Ações reparatórias

Atreladas ao princípio poluidor-pagador, as ações que visam a obrigatoriedade da organização auditada em reparar a gravidade ou a relevância dos impactos ambientais, o ressarcimento de algum prejuízo ou danos causados pela instalação/operação de empreendimentos ou pela ocorrência de desastres antropogênicos.

Adoção

Destinação de animais sem tutor identificado que foram resgatados de áreas afetadas ou evacuadas das áreas impactadas pelo desastre, com o propósito de integrar um animal a um núcleo familiar.

Ambiente

Tudo que rodeia ou envolve os seres vivos e/ou as coisas. Meio ambiente é o conjunto de condições físicas, químicas e biológicas que permite, abriga, afeta e rege a vida e os serviços do ecossistema que os sustentam (por exemplo o ciclo hidrológico e a fertilidade do solo).

Animal

Um animal pertence ao reino Metazoa, sendo definido por diversas características únicas, como seres eucariotos (células com núcleo verdadeiro separado do citoplasma), pluricelulares, sexuados e heterotróficos (ingerem alimentos) e passam pela formação de tecidos embrionários.

Animal de companhia

É um animal domesticado que é mantido principalmente para proporcionar companhia ou prazer ao seu tutor, ao invés de fins produtivos ou utilitários. Esses animais são frequentemente considerados membros da família e recebem cuidados e atenção especiais. Exemplos comuns de animais de companhia incluem cães, gatos, pássaros, peixes, e pequenos mamíferos como coelhos e *hamsters*.

Animal de produção

Refere-se a qualquer animal que seja criado e utilizado para a produção de bens ou serviços de interesse econômico. Esses animais são geralmente criados em ambientes agrícolas ou industriais para fornecer alimentos, como carne, leite, ovos, ou outros produtos como lã, couro, e fibras. Exemplos comuns de animais de produção incluem bovinos, suínos, aves, ovinos e peixes. Além disso, esses animais podem ser usados para trabalho, como em atividades de tração ou transporte.

Animal reservatório

É uma espécie de animal que hospeda um patógeno, onde este patógeno cresce e se multiplica. O agente infeccioso depende do reservatório para sua sobrevivência, para que possa ser transmitido a um hospedeiro susceptível.

Animal sinantrópico

Animais silvestres (mamíferos, aves, insetos) capazes de circular entre os ambientes silvestres, rural e urbano, com o potencial de veicular patógenos.

Animal sob tutela
Animal evacuado ou resgatado em situação de risco ou após desastre que está sob cuidados e responsabilidade da organização auditada, em moradia temporária nas instalações de fauna com tutor diferente do seu com seu tutor originário.

Aptidão comportamental
Status obtido através da avaliação realizada em um animal silvestre após reabilitação para verificar se apresenta comportamento natural de sua espécie e está apto a retornar à natureza. Para animais domésticos, está relacionada ao papel desempenhado por cada indivíduo no contexto em que está inserido.

Área de Influência (AI)
Áreas circunvizinhas à ADA e que podem sofrer impactos diretos ou indiretos à biodiversidade.

Área de Referência
Áreas que não sofreram o impacto e permitem, desta forma, a comparação com os ambientes atingidos para o diagnóstico dos danos ambientais decorrentes de um desastre.

Áreas de manejo
Espaços utilizados para o trato de animais com segurança em abrigos/cativeiros, adaptados às necessidades de cada espécie. São exemplos de áreas de manejo: recinto de cambeamento, brete, curral, embarcadouro, redondel, caixas de contenção, gaiolas, baias, piquetes, transfish etc.

Áreas de preservação permanente (APP)
Áreas protegidas por lei, com cobertura ou não por vegetação nativa, com a função ambiental de preservar os recursos hídricos, a paisagem, a estabilidade geológica, a biodiversidade, o fluxo gênico de fauna e flora, proteger o solo e assegurar o bem-estar das populações humanas.

Área Diretamente Afetada (ADA)
Áreas e corpos hídricos cujos impactos são diretos à biodiversidade.

Auditoria
Exame sistemático das atividades desenvolvidas em determinada empresa ou setor, que tem o objetivo de averiguar se estão de acordo com as disposições planejadas e/ou estabelecidas previamente, se foram implementadas com eficácia e se estão adequadas.

Banco de dados para fauna
Constitui quaisquer coleções organizadas de informações sobre um grupo de animais.

Barragem
Qualquer barreira ou parede construída transversalmente a um curso d’água ou vale para alcançar um ou mais dos seguintes objetivos: criar um reservatório (tanque ou lago) para o armazenamento de água, criar uma coluna hidrostática que possa ser usada para gerar energia, controlar enchentes e contenção de resíduos de qualquer natureza (inclusive rejeitos de mineração).

Biodiversidade
É a variabilidade entre organismos vivos de diversas regiões, incluindo, entre outros, ecossistemas terrestres, aquáticos e os complexos ecológicos dos quais fazem parte. Isso inclui diversidade dentro das espécies, entre espécies e entre ecossistemas.

Biodiversidade aquática
Refere-se à variedade de vida encontrada nos ecossistemas aquáticos, incluindo não apenas vertebrados e invertebrados, mas também plantas aquáticas, algas, fungos, bactérias, protozoários e outros microrganismos que habitam ambientes de água doce, salobra e marinha.

Bioma
É um conjunto de vida vegetal e animal, constituído pelo agrupamento de tipos de vegetação que são próximos e que podem ser identificados em nível regional, com condições de geologia e clima semelhantes e que, historicamente, sofreram os mesmos processos de formação da paisagem, resultando em uma diversidade de flora e fauna própria. O Brasil possui sete biomas reconhecidos: Amazônia, Cerrado, Pantanal, Mata Atlântica, Caatinga, Pampa e Sistema Costeiro-Marinho.

Biossegurança
Uma abordagem estratégica e integrada que abrange a política e a regulamentação de estruturas administrativas (incluindo instrumentos e atividades) que analisam e gerenciam os riscos nos setores de segurança alimentar, saúde animal e saúde vegetal, incluindo risco ambiental associado. A biossegurança é um conceito holístico de relevância direta para a sustentabilidade da agricultura, segurança alimentar e proteção do meio ambiente, incluindo a biodiversidade.

Campo
Fruto da observação de dados *in natura*, ação de entrar em contato físico com determinada atividade no local onde o evento estudado ocorre naturalmente. Engloba a coleta e/ou registro de dados, caracteres, informações relativas ao fenômeno ou objeto de estudo.

Capacidade de Prover Cuidados (CPC)
É a capacidade de atuação com o número adequado de animais, de acordo com os recursos materiais, físicos e humanos disponíveis na instalação de fauna. O CPC é baseado nas avaliações não só da estrutura física e ambiente, como também diz respeito aos procedimentos internos de organização de documentações e banco de dados, protocolos de admissão e saída de animais, procedimentos de higienização e destinação de resíduos, manejo nutricional, sanitário, comportamental, dentre outros.

Capineira
Área cultivada com gramíneas de elevado potencial de produção de forragem que serão cortadas e picadas para fornecimento como verde picado aos animais durante o período seco do ano.

Cativeiro
Atividade humana de manter preso, em área determinada, espécies não domésticas, em geral com propósitos de manutenção, crescimento e reprodução de espécies.

Causa mortis
Expressão em latim que significa causa da morte, utilizada após a submissão de procedimentos de necropsia para determinar a causa da morte em um documento.

Centro de Triagem de Animais Silvestres (CETAS)
São unidades responsáveis pelo recebimento e manejo dos animais silvestres oriundos de ação fiscalizatória, resgate ou entrega voluntária de particulares.

Dados qualitativos
Informações não numéricas.

Dados quantitativos
Informações numéricas.

Desastre
Evento, acontecimento que causa sofrimento e grande prejuízo.

Desastre ambiental
Evento ou acontecimento que afeta negativamente o meio ambiente, causando danos incalculáveis e de difícil restituição. Pode ser de origem natural, antropogênica ou mista.

Desenvolvimento sustentável
Desenvolvimento capaz de suprir as necessidades da geração atual, sem comprometer a capacidade de atender as necessidades das futuras gerações. É o desenvolvimento que não esgota os recursos para o futuro.

Dessedentação
Método para suprir necessidades de água para contingentes animais.

Diagnóstico ambiental
Levantamento de informações sobre diferentes componentes ambientais da área alvo, de forma a conhecer e contextualizar o território para a avaliação dos riscos e danos potenciais em um desastre ambiental e, principalmente, o planejamento de ações para a proteção da fauna em uma emergência. É composto por uma caracterização da linha de base quanto à biodiversidade e aos serviços ecossistêmicos.

Doenças endêmicas
São aquelas que se manifestam com frequência em determinadas regiões, provocadas por circunstâncias ou causas locais, como varicela, malária, dengue, febre amarela, esquistossomose, leishmaniose, doença de Chagas, entre outras.

Doenças infecciosas
Doenças causadas por um agente infeccioso específico ou por seu produto tóxico.

Doenças infectocontagiosas
Doença de fácil e rápida transmissão, provocada por agentes patogênicos.

Doenças ocupacionais
Doenças diretamente relacionadas ao processo do trabalho, ou seja, às condições de trabalho e ao ofício com o qual o trabalhador está envolvido.

Ecossistema
Complexo dinâmico de comunidades de plantas, animais e microrganismos e seu ambiente abiótico, interagindo como uma unidade funcional.

Ectoparasitas
Parasitas que se fixam no exterior do corpo do hospedeiro, como pulgas e carrapatos.

Educação ambiental
São os processos por meio dos quais o indivíduo e a coletividade constroem valores sociais, conhecimentos, habilidades, atitudes e competências voltadas para a conservação do meio ambiente, bem de uso comum do povo, essencial à sadia qualidade de vida e sua sustentabilidade (Política Nacional de Educação Ambiental – Lei Federal nº 9.795/1999, art. 1º).

Elementos bióticos e abióticos
Bióticos são os organismos vivos e abióticos são os componentes físicos, químicos ou físico-químicos do ambiente que influênciam os seres vivos em um ecossistema, como a luz, a temperatura, o vento.

Enchente
É um processo natural ou potencializado pela ação antrópica que acontece por meio do aumento do volume de água nos rios, córregos ou canal, alcançando seu ponto máximo sem transbordar.

Endêmica
Ocorre somente em uma determinada área ou região geográfica.

Endoparasitas
Parasitas que se instalam no interior do corpo do hospedeiro, como vermes e protozoários.

Enriquecimento ambiental
Processo dinâmico no qual mudanças na estrutura e nas implantações de práticas de manejo com estratégias temporais, físicas, sociais e sensoriais visam oferecer uma série de estímulos que possam aumentar o conforto e a capacidade de adaptação do animal ao abrigo e cativeiro.

Enriquecimento comportamental
Inserção de objetos e atividades que modifiquem o ambiente físico ou social dos animais, visando modulação comportamental do animal para melhoria da qualidade de vida do animal abrigado, proporcionando condições mais próximas das necessidades etológicas de cada espécie.

Epidemia
Quando novos casos de uma doença, em uma determinada população humana e durante um determinado período, excedem substancialmente o que se espera com base na experiência recente. A doença não precisa ser transmissível.

Epidemiologia
Ciência básica para a saúde coletiva que estuda a distribuição e os determinantes dos problemas de saúde (fenômenos e processos associados) em populações humanas ou não humanas. Seu objeto são as relações de ocorrência de saúde-doença em massa. As relações são referidas e analisadas mediante o conceito de risco.

Equipamento de proteção coletiva (EPC)
Todo dispositivo ou produto utilizado para proteção contra riscos fornecidos pelo ambiente de trabalho, de maneira coletiva.

Equipamento de proteção individual (EPI)
Todo dispositivo ou produto, de uso individual utilizado pelo trabalhador, destinado à proteção contra riscos capazes de ameaçar a sua segurança e a sua saúde.

Espécie
Conjunto de indivíduos semelhantes e com potencial reprodutivo entre si, capazes de originar descendentes férteis, incluindo aqueles que se reproduzem por meios assexuados.

Espécime
Indivíduo vivo ou morto, de uma espécie, em qualquer fase de seu desenvolvimento.

Etologia
Ciência que estuda o comportamento animal.

Evacuação
Consiste em sair ou ser retirado de uma área de risco, deixando-a vazia, de forma emergencial ou preventiva.

Evidência
Registro que não dá margem a dúvida em que se baseia o processo de auditoria técnica independente.

Extrativismo
Não se baseia somente na coleta em si de produtos da natureza, mas em um modo de vida e cultura de comunidades tradicionais, por exemplo, atividades extrativistas de consumo de estoques pesqueiros, piscicultura, meliponicultura (abelhas nativas) e caça.

Família multiespécie
É conceituada como aquela lastreada essencialmente na afetividade inerente à relação humano-animal.

Fauna doméstica de companhia
Conjunto de todas as espécies de animais mantidas principalmente para proporcionar companhia ou prazer ao seu tutor, ao invés de fins produtivos ou utilitários. São dotadas de mansidão hereditária e podem ser encontradas em sistema produtivo com fins de venda como animais de companhia.

Fauna doméstica de produção
Conjunto de todas as espécies de animais criadas em sistemas produtivos projetados para a produção de alimentos, fibras ou outros produtos de origem animal. Esses animais foram submetidos historicamente aos processos tradicionais e sistematizados de manejo e/ou melhoramento zootécnico. Eles se tornaram domésticos, apresentando características biológicas e comportamentais em estreita dependência do homem, podendo apresentar fenótipo variável, diferente da espécie silvestre que os originou.

Fauna edáfica
Comunidade de invertebrados que vivem permanentemente no solo ou que passa um ou mais ciclos de vida no solo.

Fauna invertebrada
São os animais que não possuem coluna vertebral, mais 31 filos animais, entre eles: poríferos (esponjas), cnidários (por exemplo, medusas, águas-vivas e corais), platelmintos (por exemplo, planária), anelídeos (por exemplo, minhocas, sanguessugas) e artrópodes (por exemplo, insetos, aranhas, escorpiões, caranguejos, camarões, lagosta, centopeias, gongolos).

Fauna silvestre exótica
Conjunto de espécies cuja distribuição geográfica ocorre em território distinto daquele tomado por referência, ainda que introduzidas, pelo homem ou espontaneamente, em ambiente natural, inclusive as espécies asselvajadas e excetuadas as migratórias.

Fauna silvestre nativa
Todo animal pertencente à espécie nativa, migratória e a qualquer outra não exótica, que tenha todo ou parte do seu ciclo de vida ocorrendo dentro dos limites do território brasileiro ou águas jurisdicionais brasileiras.

Fauna vertebrada
São animais que possuem coluna vertebral, ou seja, pertencem ao filo Chordata, subfilo Vertebrata.

Fitofisionomia
Aspecto da vegetação que se encontra em determinado lugar.

Fômites
Objetos e superfícies inanimadas que podem levar e espalhar uma doença e agentes infecciosos.

Função ecológica
Simplificadamente, pode-se dizer que é o papel que cada espécie desempenha em seu ecossistema, incluindo predação, aeração do solo, forrageamento etc.

Guarda responsável
Condição na qual o guardião de um animal aceita e se compromete a assumir uma série de deveres centrados no atendimento das necessidades físicas, psicológicas e ambientais de seu animal, assim como prevenir os riscos (potencial de agressão, transmissão de doenças ou danos a terceiros) que seu animal possa causar à comunidade ou ao ambiente, como interpretado pela legislação vigente.

Guia de Transporte Animal (GTA)
Documento oficial para transporte animal que contém informações essenciais sobre a rastreabilidade (origem, destino, finalidade, espécie, vacinações, entre outros).

Guilda ecológica
Como qualquer conjunto de espécies que subsistem de um mesmo tipo de recurso, explorando-o de forma similar.

Habitat
Conjunto de circunstâncias físicas e geográficas que oferece condições favoráveis à vida e ao desenvolvimento de determinada espécie animal ou vegetal.

Impacto ambiental
Alteração de condições do meio ambiente e/ou dos elementos inseridos nele em consequência de atividades humanas.

Imunização
Aquisição de proteção imunológica contra uma doença infecciosa.

Instalação de fauna
Sinônimo para Abrigo de Fauna.

Interações ecológicas
Interações que ocorrem entre os seres vivos que vivem em um determinado ambiente, podendo ser benéficas ou prejudiciais.

Inundações
É o processo em que ocorre submersão de áreas fora dos limites normais de um curso de água em zonas que normalmente não se encontram submersas. O transbordamento ocorre de modo gradual em áreas de planície, geralmente ocasionado por chuvas distribuídas e alto volume acumulado na bacia de contribuição.

Inventário de fauna
Estudo que busca conhecer a fauna presente na área e sua relação com o ambiente no entorno. Em áreas de risco para desastres ambientais permite determinar o número de pessoas e animais domésticos do local, além das condições de moradia.

Leishmaniose
Doença infecciosa causada por um protozoário do gênero Leishmania, considerado um parasita.

Leptospirose
Doença de caráter infeccioso que é causada por uma bactéria, a *Leptospira interrogans*. Essa bactéria vive nos rins de diversos animais, como os roedores, sendo eliminada na urina desses animais. O contato com essa secreção faz a transmissão da leptospirose para os seres humanos.

Levantamento de fauna silvestre
Objetiva caracterizar a fauna presente na área de interesse, com vistas a orientar o planejamento de ações para a proteção dos animais em um cenário de desastre ambiental, bem como obter uma linha de base da biodiversidade local no cenário anterior ao desastre. Nesse contexto, o objetivo principal é conhecer as espécies da fauna que potencialmente serão afetadas, com especial atenção às ameaçadas de extinção, raras, endêmicas, de distribuição restrita, migratórias, invasoras ou de relevância ecológica/econômica.

Licenciamento ambiental
Instrumento de prevenção e fiscalização, instituído pela Política Nacional do Meio Ambiente (Lei Federal nº 6.938/1981) que consiste em um procedimento administrativo pelo qual o órgão competente licencia as atividades do empreendedor que potencialmente podem causar danos ambientais.

Linha de base da biodiversidade
Trabalho de coletar e interpretar informações sobre os valores de biodiversidade presentes em um local, a sua condição atual e tendências anteriores ao início de um projeto. O estudo de linha de base da biodiversidade desempenha funções importantes de apoio à avaliação dos impactos e riscos de um projeto, apoiando a aplicação da hierarquia de mitigação para a biodiversidade e delineando o programa de monitoramento da biodiversidade de longo prazo.

Malha hídrica
Conjunto de infraestrutura e instalações para extração, transporte, armazenamento e distribuição de água.

Manejo
Tipo de intervenção humana que ocorre de forma ocasional ou sistemática, em cativeiro ou na natureza, visando manter, recuperar ou controlar populações silvestres, domésticas, domesticadas ou asselvajadas para assegurar a estabilidade dos ecossistemas, dos processos ecológicos ou dos sistemas produtivos. Atualmente, existem três concepções para a palavra manejo, o manejo para conservação, o manejo de animais de companhia e o manejo produtivo. O manejo ético de animais deve combinar técnica e responsabilidade através da aplicação de conhecimentos e habilidades nas áreas de etologia, fisiologia, bem-estar e direito animal.

Manejo comportamental
Consiste no monitoramento, diagnóstico, compreensão e tratamento de desvios comportamentais apresentados pelos animais abrigados de quaisquer espécies, sejam eles ocasionados por estresse pela alteração de rotina ou remoção de seu ambiente habitual, ausência dos tutores, confinamento incorreto, ou ainda por doenças subdiagnosticadas.

Medidas profiláticas
Conjunto de precauções que tem como objetivo evitar ou reduzir o risco de transmissão de uma doença.

Mitigação
Intervenções visando reduzir ou remediar os impactos ambientais nocivos da atividade humana. Também se refere ao ato de suavizar os efeitos de um evento.

Necropsia
Procedimentos e observações, organizada e hierarquizada, realizada ao cadáver com o objetivo de determinar o que provocou o óbito.

Paisagem
Um conjunto de formas naturais, rurais, industriais modificadas culturalmente e associadas a uma dada escala de tempo.

Patógenos
Organismos que podem provocar uma doença.

Pet Alert
Adesivo de segurança utilizado nas residências com o objetivo de sinalizar as propriedades que possuem animais a serem resgatados ou evacuados em caso de emergência.

Plano de Gerenciamento de Resíduos Sólidos (PGRS)
Documento técnico que identifica os tipos e a quantidade de geração de cada tipo de resíduos sólidos e indica formas ecologicamente corretas para manejo, transporte e disposição final, entre outros, baseado na legislação vigente (âmbito Federal na Lei nº 12.305/2010).

Pós-adoção
É o acompanhamento por uma equipe especializada de cada animal adotado por um período de tempo, com a finalidade de avaliar sua adaptação aos novos tutores, permanência do bem-estar e do ambiente na nova moradia, atualização dos protocolos sanitários das necessidades nutricionais, comportamentais, de segurança e controle reprodutivo.

Procedimento Operacional Padrão (POP)
Documento que estabelece de forma minuciosa os detalhes de um processo, elencando aspectos como sequência de procedimentos, materiais utilizados, cuidados a serem observados, responsáveis por cada etapa.

Programa
Conjunto de projetos planejados e organizados para um objetivo em comum.

Projeto
Descrição escrita e detalhada de um empreendimento a ser realizado; plano, delineamento, esquema.

Propágulos
Qualquer parte de um organismo que possa dar origem a novos indivíduos da mesma espécie e que, portanto, possibilite o estabelecimento de uma nova população.

Prospecção
A prospecção de carcaças envolve a busca sistemática por restos mortais de animais vítimas de desastres para entender as causas de mortalidade, avaliar impactos ambientais e fornecer dados valiosos para a conservação da biodiversidade.

Quarentena
É um tipo de reclusão aplicada a determinado grupo de animais sadios, mas que podem ter sido contaminados por algum agente causador de alguma doença, a fim de evitar que ela se espalhe. Setor destinado para a reclusão desses indivíduos recém-chegados em uma instalação de fauna com o intuito de aguardar o período de incubação de uma doença, protegendo, assim, os outros animais abrigados.

Rastreabilidade
Sistema de controle de animais que permite sua identificação individual desde o nascimento até o momento atual, registrando todas as ocorrências relevantes ao longo de sua vida.

Reabilitação de animais silvestres
Consiste em recuperar indivíduos com injúrias físicas, fisiológicas ou comportamentais, seja para a devolução ao seu *habitat* original ou para adaptação ao cativeiro.

Recomendação
Instrumento pelo qual este expõe, em ato formal, razões fáticas sobre determinada questão, com o objetivo de persuadir o destinatário a praticar ou deixar de praticar determinados atos em benefício da melhoria dos serviços e de relevância ou do respeito aos interesses, direitos e bens defendidos pela instituição, atuando, assim, como instrumento de prevenção de responsabilidades ou correção de condutas.

Reintegração ao tutor
Conjunto de atividades desenvolvidas com o objetivo de realizar, de forma idônea, a devolução dos animais, que se encontram sob tutela provisória do empreendimento responsável pela evacuação ou resgate, aos seus tutores originais.

Reintrodução
Ato de soltar indivíduos de uma determinada espécie em um local de ocorrência histórica, distante da região considerada como possivelmente afetada pelo desastre.

Resgate de fauna
Consiste na retirada ativa de animais das áreas de risco ou afetadas pelo desastre.

Responsável Comunitário
Pessoa designada ou voluntária que atua como um ponto de contato entre uma organização, entidade ou grupo e a comunidade local.

Risco
É a probabilidade de ocorrer consequências danosas ou perdas esperadas (mortos, feridos, edificações destruídas e danificadas etc.), como resultado de interações entre um perigo natural e as condições de vulnerabilidade local.

Rompimento de barragem
Falha na contenção dos resíduos armazenados em barragens, causando o lançamento do material com potenciais danos ambientais, econômicos e sociais. Neste manual, o rompimento abrange eventos de transbordamento e vazamento.

Saúde Única
Do termo *One Health*, compreende abordagens com enfoque intersetorial e transdisciplinar de iniciativas necessárias para promover e proteger a saúde das pessoas, animais, plantas e ambiente de maneira integrada. A inseparabilidade da saúde humana, animal e ambiental também é promovida pelos movimentos como Uma só Saúde, *Planetary Health*, *Ecohealth*, *One Welfare*, entre outros.

Serviços ecossistêmicos
Os benefícios que as pessoas obtêm dos ecossistemas, que podem ser de bens incluindo serviços de abastecimento, como alimentos e água; serviços reguladores, como controle de enchentes e doenças; serviços culturais, como benefícios espirituais, recreativos e culturais; e serviços de apoio, como ciclagem de nutrientes, que mantêm as condições de vida na Terra.

Simulados de evacuação
Exercícios práticos que implicam na mobilização de recursos e pessoas para avaliar, em tempo real, o processo de remoção de pessoas e animais de áreas com risco de desastres.

Táxon
Unidade taxonômica, essencialmente associada a um sistema de classificação científica. O táxon pode indicar uma unidade em qualquer nível de um sistema de classificação: um reino, gênero e uma espécie são taxas assim como qualquer outra unidade de um sistema de classificação dos seres vivos.

Termo de adoção
Documento em que o adotante se declara apto a assumir a guarda e a responsabilidade sobre a saúde e integridade do animal, e de estar ciente dos cuidados e manutenção, riscos inerentes à espécie no convívio humano e se compromete a proporcionar boas condições de bem-estar.

Termo de Ajustamento de Conduta (TAC)
É um acordo que o Ministério Público celebra com o violador de determinado direito coletivo. Esse instrumento tem a finalidade de impedir a continuidade da situação de ilegalidade, reparar o dano ao direito coletivo e evitar a ação judicial.

Termo de Conduta (TC)
É um instrumento jurídico que formaliza o acordo de vontades entre duas ou mais partes, onde uma ou ambas se comprometem a cumprir determinadas obrigações ou responsabilidades.

Toxicológica
Análise para determinação dos limites apresentados de metais e compostos orgânicos em diversas matrizes no organismo dos animais, auxiliando o diagnóstico e monitoramento dos casos de contaminação.

Translocação
A movimentação de organismos vivos para posterior soltura em outra área previamente determinada conforme a distribuição geográfica da espécie.

Vetor
É todo ser vivo invertebrado capaz de transmitir um agente infectante (vírus, bactéria, protozoário ou parasita) de forma ativa (o vetor é infectado) ou passiva (o vetor não se infecta).

Visão ecossistêmica
Estratégia para a gestão integrada dos sistemas naturais e recursos vivos que promova a conservação e o uso sustentável de forma equitativa.

Vulnerabilidade
Série de características socialmente construídas que tornam a sociedade suscetível a danos e perdas e que enfrentam dificuldades para se recuperar de forma autônoma após desastres.

Zoneamento urbano
Plano que divide um determinado espaço (cidade) em zonas territoriais e determina, para cada uma delas, as regulamentações pertinentes quanto ao uso e à ocupação do solo.

Zoonose
Doença ou infecção que é naturalmente transmitida entre pessoas e animais.

AECOM

É com grande satisfação que apresentamos o Manual de Auditoria Técnica para a Proteção da Fauna em Desastres e Evacuações por Barragens. Desenvolvido pela Equipe de fauna da AECOM do Brasil, este manual compartilha as lições aprendidas durante o processo de auditoria técnica independente focado no bem-estar de animais domésticos e silvestres.

Ao explorar este manual, você terá acesso à perspectiva do auditor de fauna e aos conhecimentos adquiridos por meio da experiência com animais em cenários de desastre e emergência. Com um conteúdo estratégico e adaptável, que reflete a complexidade do tema, este manual foi criado para avaliar ações de resposta em emergências e de salvaguarda animal, promovendo ações de proteção eficazes em situações de crise. Cada capítulo foi elaborado para orientar auditores, gestores e demais interessados em um processo de avaliação de conformidade, baseado no princípio da dignidade animal, no conceito dos cinco domínios e diversas temáticas de vanguarda relacionadas à senciência animal.

Ainda há um longo caminho a percorrer na construção de uma sociedade que interaja de forma mais saudável e ética com o ambiente e todos os seres vivos. Esperamos que este manual seja uma fonte de aprendizado e inspiração, incentivando a adoção de práticas mais responsáveis e eficazes na proteção animal.

ISBN: 978-65-985342-0-2
CBL
9 786598 534202

AECOM Delivering a better world
